# HOXC9 accelerates esophageal squamous cell carcinoma progression via OTUD1-FABP5-mediated lipid metabolic reprogramming

**DOI:** 10.1038/s41419-026-09013-4

**Published:** 2026-06-30

**Authors:** Cai Zhang, Yangyang Ji, Yilu Tong, Yue Du, Mingyuan Zhang, Yongfeng Wang

**Affiliations:** 1 Department of Clinical Laboratory, The First Affiliated Hospital of Zhengzhou University, Zhengzhou, China; 2 Key Clinical Laboratory of Henan Province, Zhengzhou, China

**Keywords:** Cancer, Oncogenes, Cancer metabolism

## Abstract

Homeobox C9 (HOXC9) plays a critical role in tumor progression. However, its function and regulatory mechanisms in esophageal squamous cell carcinoma (ESCC) remain unclear. Here, we found that HOXC9 expression was significantly upregulated in ESCC (|log_2_FC| ≥ 2, *p* < 0.05) and was positively associated with poor prognosis in ESCC patients (*p* = 0.032). HOXC9 promoted ESCC progression in vitro and in vivo. Mechanistically, HOXC9 directly activated *ovarian tumor deubiquitinase 1* (*OTUD1*) transcription by binding to its promoter region. This activation enhanced OTUD1-mediated fatty acid binding protein 5 (FABP5) deubiquitination, increasing FABP5 protein stability, reducing lipid droplet accumulation, and elevating glycerol and free fatty acid levels (*p* < 0.05), thereby accelerating ESCC cell proliferation and migration. In addition, HOXC9-OTUD1-FABP5 signaling was closely linked to the clinicopathological grade of ESCC patients. Our study comprehensively reveals the mechanism by which HOXC9 accelerates ESCC progression, and identifies potential biomarkers and therapeutic targets for the pathogenesis and clinical treatment of ESCC.

**Figure:**
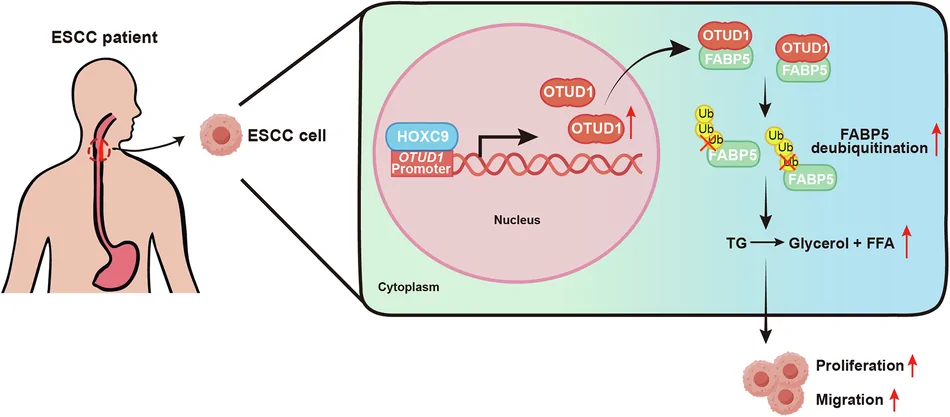

## Introduction

Esophageal cancer (EC) is one of the most common malignant tumors and ranks seventh among the leading causes of cancer-related deaths worldwide [1, 2]. EC can be classified as esophageal squamous cell carcinoma (ESCC) or esophageal adenocarcinoma (EAC) based on histological subtype, with ESCC accounting for approximately 90% of cases [3]. Despite significant advancements in the diagnosis and treatment of ESCC, the mortality rate remains high [4, 5]. Therefore, investigations into the molecular mechanisms underlying ESCC progression are urgently needed to identify promising therapeutic targets.

The homeobox (HOX) family encodes evolutionarily conserved transcription factors and was initially found to play critical roles in embryonic development [6–8] and adipogenesis [9, 10]. To date, the role of the HOX family in tumor occurrence and development has been increasingly elucidated. The HOX family is divided into four gene clusters: HOXA, HOXB, HOXC, and HOXD [11]. Homeobox C9 (HOXC9), a member of the HOXC family, has been reported to be a biomarker of lipolysis [12], while its paralogue HOXA9 can inhibit adipogenesis [13]. In addition, HOXC9 is aberrantly expressed and contributes to tumor progression in several types of cancers, including breast cancer [14], gastric cancer [15, 16], and lung cancer [17]. For example, HOXC9 increases tumor invasiveness and promotes a phenotypic transition from a proliferative to an invasive state in breast cancer [14]. HOXC9 also confers resistance to interferon-gamma treatment in gastric cancer by inhibiting the DAPK1/RIG1/STAT1 axis [15], which may be a novel prognostic and diagnostic marker [16]. Liu et al. reported that HOXC9 serves as a biomarker to enhance the predictive efficacy of immune checkpoint inhibitors combined with chemotherapy and is a viable target for oncogenes and immunotherapy in lung adenocarcinoma [17]. However, the molecular mechanisms and clinical value of HOXC9 in ESCC remain unclear.

Accumulating evidence indicates that deubiquitinating enzymes (DUBs) can regulate protein stability, localization, and activity in diverse cellular processes and contribute to cancer development [18–20]. Ovarian tumor deubiquitinase 1 (OTUD1), a member of the OTU domain-containing family, has been gradually recognized as a critical regulator of tumorigenesis through its deubiquitinating activity [21, 22]. For instance, OTUD1 is highly expressed in ovarian cancer and is positively associated with poor prognosis. Mechanistically, OTUD1 interacts with apoptosis signal-regulating kinase-1 (ASK1) to maintain its protein stability, which, in turn, activates the downstream JNK signaling pathway to sustain ovarian cancer stem cell properties [22]. Liu et al. reported that OTUD1 expression is downregulated in renal cell carcinoma, where it interacts with phosphatase and tensin homolog (PTEN) to inhibit the PI3K/AKT and TNF-alpha/NF-κB signaling pathways, thereby increasing renal cancer cell sensitivity to tyrosine kinase inhibitors (TKIs) [23]. Understanding the potential mechanisms underlying OTUD1 dysregulation in ESCC progression will aid in the development of more effective treatment strategies.

Lipid metabolism reprogramming, including lipid uptake, de novo lipogenesis, storage, and degradation, is an essential indicator of tumor progression [24–26]. Fatty acid binding protein 5 (FABP5), a member of the fatty acid-binding protein family, plays vital roles in regulating lipolysis and the synthesis of glycerol and free fatty acids in tumor cells [27, 28], thereby accelerating the progression of cervical cancer [29], prostate cancer, and breast cancer [30]. Pre-mRNA processing factor 19 (PRP19) promotes fatty acid synthesis by enhancing *sterol regulatory element-binding protein 1* (*SREBF1*) mRNA stability in an N^6^-methyladenosine-dependent manner and by accelerating ESCC progression [31]. In addition, Tian et al. reported that carnitine O-palmitoyl transferase 1 (CPT1A), a key enzyme involved in fatty acid oxidation, maintains redox homeostasis by supplying GSH and NADPH, thereby protecting ESCC cells from apoptosis [32]. However, research on the potential significance of FABP5-mediated lipid metabolism reprogramming in ESCC progression is limited, which makes it a promising direction for future ESCC-targeted therapy research.

In this study, we demonstrate that HOXC9 is highly expressed in ESCC and positively correlates with poor prognosis in ESCC patients. HOXC9 activates *OTUD1* transcription by directly binding to its promoter region, thereby upregulating FABP5 expression via OTUD1-mediated deubiquitination. This reduces lipid droplet accumulation and increases glycerol and free fatty acid levels, thereby accelerating ESCC cell proliferation and migration. Our findings identify potential biomarkers and therapeutic targets for the pathogenesis and clinical treatment of ESCC.

## Results

### HOXC9 is upregulated in ESCC and is positively associated with poor prognosis in ESCC patients

**Fig. 1:**
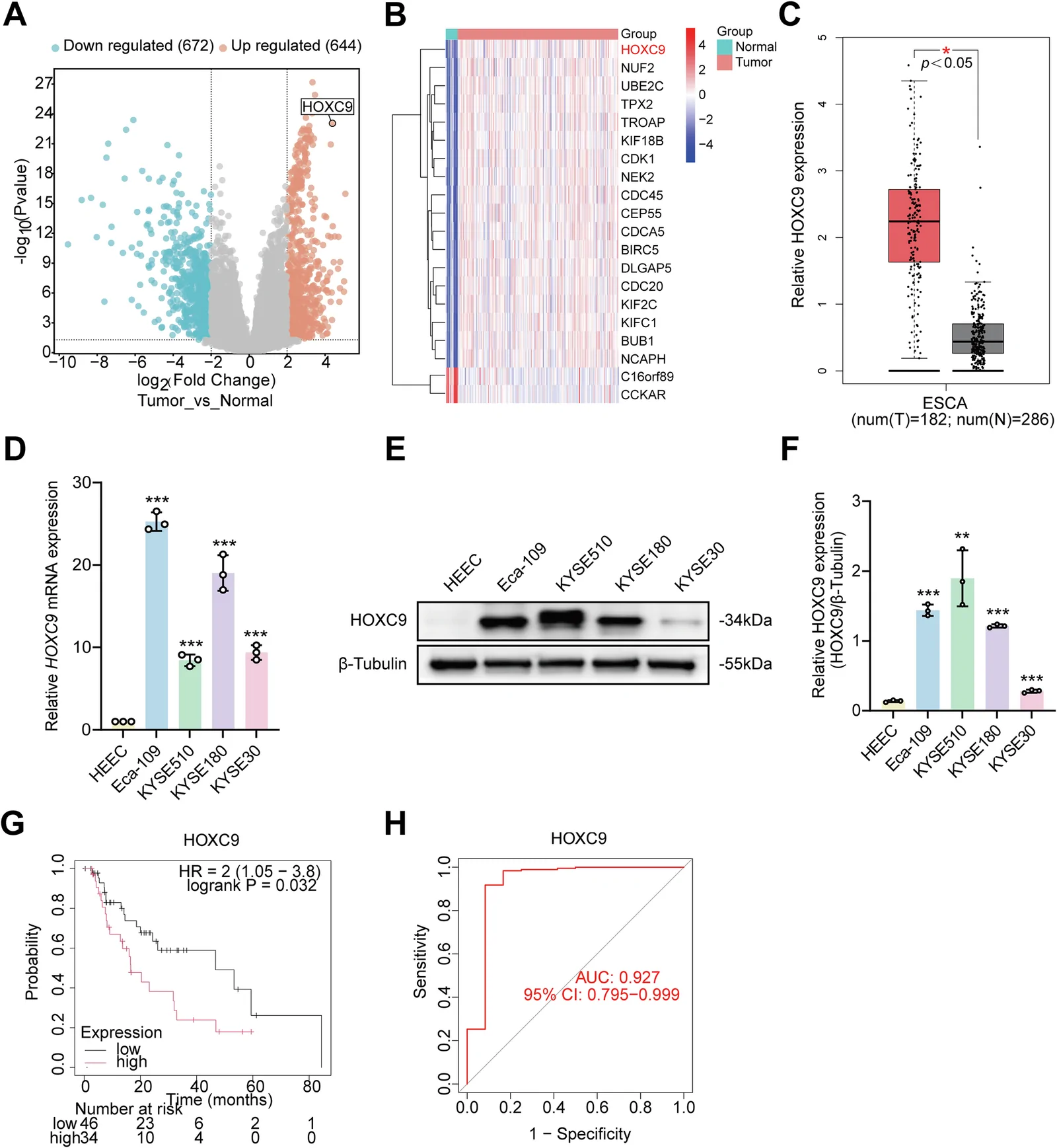
HOXC9 is upregulated in ESCC and is positively associated with poor prognosis in ESCC patients. **A** Volcano plot of differentially expressed genes in ESCC obtained from the TCGA database (|log_2_FC| ≥ 2, *p* < 0.05). **B** Heatmap of top 20 differentially expressed genes in ESCC obtained from the TCGA database (|log_2_FC| ≥ 2, *p* < 0.05). **C** GEPIA analysis of HOXC9 expression in normal and ESCC tissues (T tumor tissues; N normal tissues). HOXC9 mRNA and protein expression in HEEC and four ESCC cells (Eca-109, KYSE510, KYSE180, KYSE30) were detected by RT-qPCR (**D**) and western blot (**E**, **F**). **G** Kaplan–Meier plotter analysis of ESCC patients with different HOXC9 expression. HR hazard ratio. **H** ROC curve of HOXC9 expression in patients with ESCC obtained from TCGA. The experiments (**D**–**F**) were repeated three times. Data are shown as mean ± SD and determined by one-way ANOVA (**D**, **F**). **p < 0.01; ***p < 0.001.

To identify novel targets associated with ESCC progression, we extracted 19 527 genes that were differentially expressed in ESCC (|log_2_FC| ≥ 2, *p* < 0.05) from the TCGA database (Fig. 1A), among which HOXC9 was the top-ranked expressed gene in ESCC (Fig. 1B). Consistently, GEPIA analysis revealed that HOXC9 expression was significantly upregulated in ESCC tissues compared with adjacent normal esophageal tissues (Fig. 1C). We also detected elevated HOXC9 expression in ESCC cell lines compared with HEECs (Fig. 1D–F). Furthermore, Kaplan-Meier plotter analysis demonstrated that ESCC patients with high HOXC9 expression had a poorer prognosis (Fig. 1G). More importantly, receiver operating characteristic (ROC) curve analysis revealed that HOXC9 exhibited excellent diagnostic performance for ESCC patients (AUC = 0.927, 95% CI: 0.795–0.999; Fig. 1H).

Collectively, these data suggest that HOXC9 is upregulated in ESCC and is positively associated with poor prognosis in ESCC patients.

### HOXC9 accelerates ESCC progression in vitro and in vivo

**Fig. 2:**
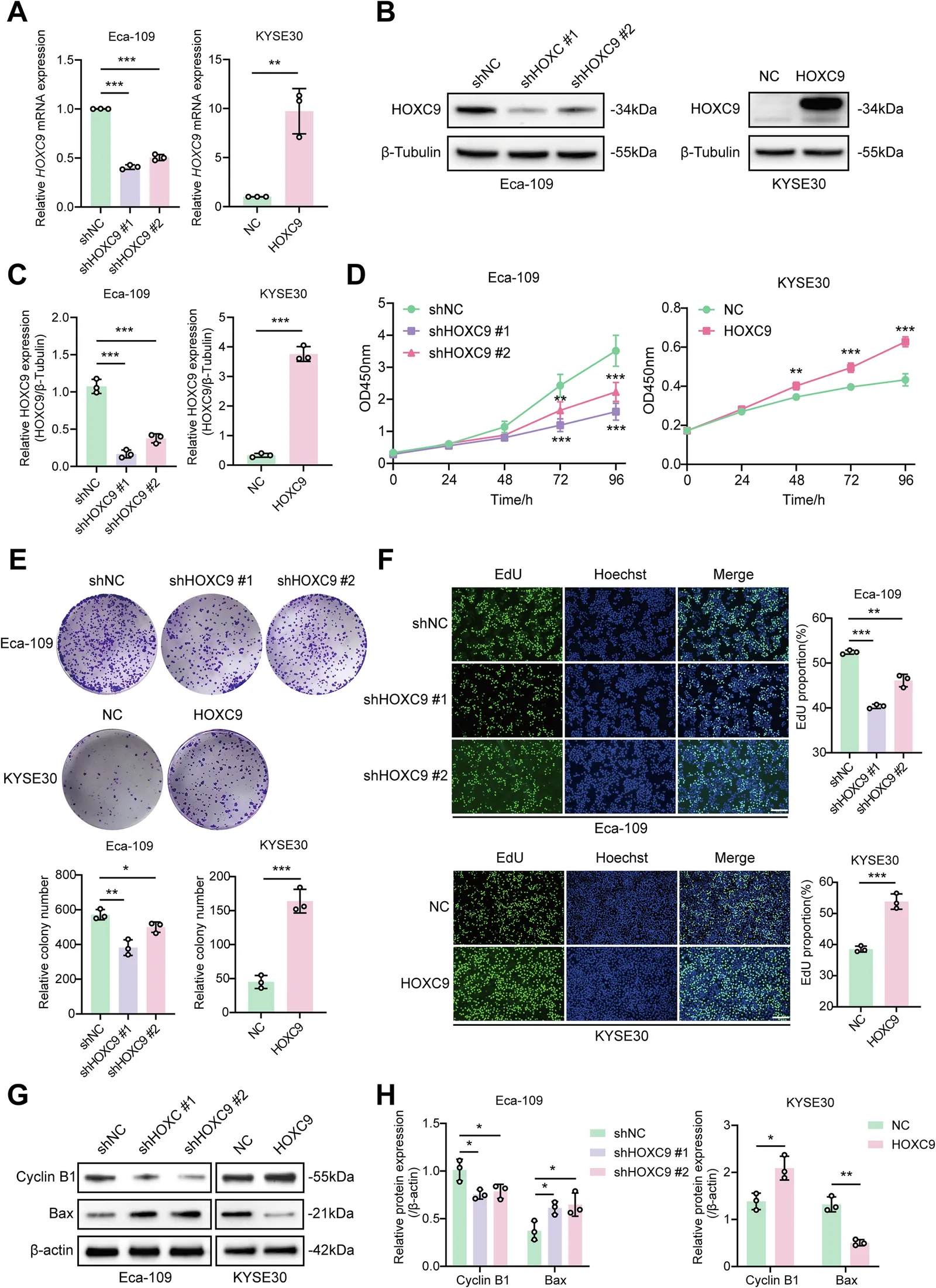
HOXC9 promotes the proliferation of ESCC cells in vitro. Eca-109 cells (with higher endogenous HOXC9 levels) were transduced with control lentivirus (shNC) and HOXC9 shRNA lentivirus (shHOXC9#1, shHOXC9#2). KYSE30 cells (with low baseline endogenous HOXC9 expression) were transduced with empty lentivirus (NC) and HOXC9-overexpressing lentivirus (HOXC9). HOXC9 expression in Eca-109 (shNC, shHOXC9#1, shHOXC9#2) and KYSE30 cells (NC, HOXC9) was detected by RT-qPCR (**A**) and western blot (**B**, **C**). The proliferation of Eca-109 (shNC, shHOXC9#1, shHOXC9#2) and KYSE30 cells (NC, HOXC9) was detected by CCK-8 assays (**D**), colony-formation assays (**E**) and EdU assays (**F**). Scale bar, 200 μm (EdU assays). **G**, **H** Cyclin B1 and Bax protein expression in Eca-109 (shNC, shHOXC9#1, shHOXC9#2) and KYSE30 cells (NC, HOXC9) was detected by western blot. All experiments were repeated three times. Data are shown as the mean ± SD; statistical significance was determined by one-way ANOVA in Eca-109 cells and unpaired t-test in KYSE30 cells for (**A**, **C**–**F**, **H**). *p < 0.05; **p < 0.01; ***p < 0.001.

Next, we investigated the role of HOXC9 in ESCC. Eca-109 and KYSE510 cells were transduced with control or HOXC9 shRNA lentivirus, whereas KYSE30 and Eca-109 cells were transduced with empty lentivirus or HOXC9-overexpressing lentivirus (Figs. 2A–C, and S1A–C). CCK-8 assays showed that HOXC9 knockdown reduced the cell viability in Eca-109 and KYSE510 cells, whereas HOXC9 overexpression markedly increased the viability of KYSE30 and Eca-109 cells (Fig. 2D and S1D). Colony formation assays demonstrated that HOXC9 knockdown significantly impaired cluster formation capacity of Eca-109 and KYSE510 cells, while HOXC9 overexpression enhanced colony formation of KYSE30 and Eca-109 cells (Figs. 2E and S1E). Similarly, EdU assays revealed that HOXC9 knockdown substantially decreased the number of EdU-positive cells, whereas HOXC9 overexpression significantly promoted cell proliferation (Figs. 2F and S1F). HOXC9 knockdown reduced Cyclin B1 expression and increased the apoptosis-related protein Bax, whereas HOXC9 overexpression promoted Cyclin B1 but inhibited Bax expression in ESCC cells (Fig. 2G, H). Additionally, wound-healing and Transwell assays showed that HOXC9 knockdown inhibited the migration of Eca-109 and KYSE510 cells, whereas HOXC9 overexpression promoted ESCC cell migration (Figs. S1G, H and S2A–D).

**Fig. 3:**
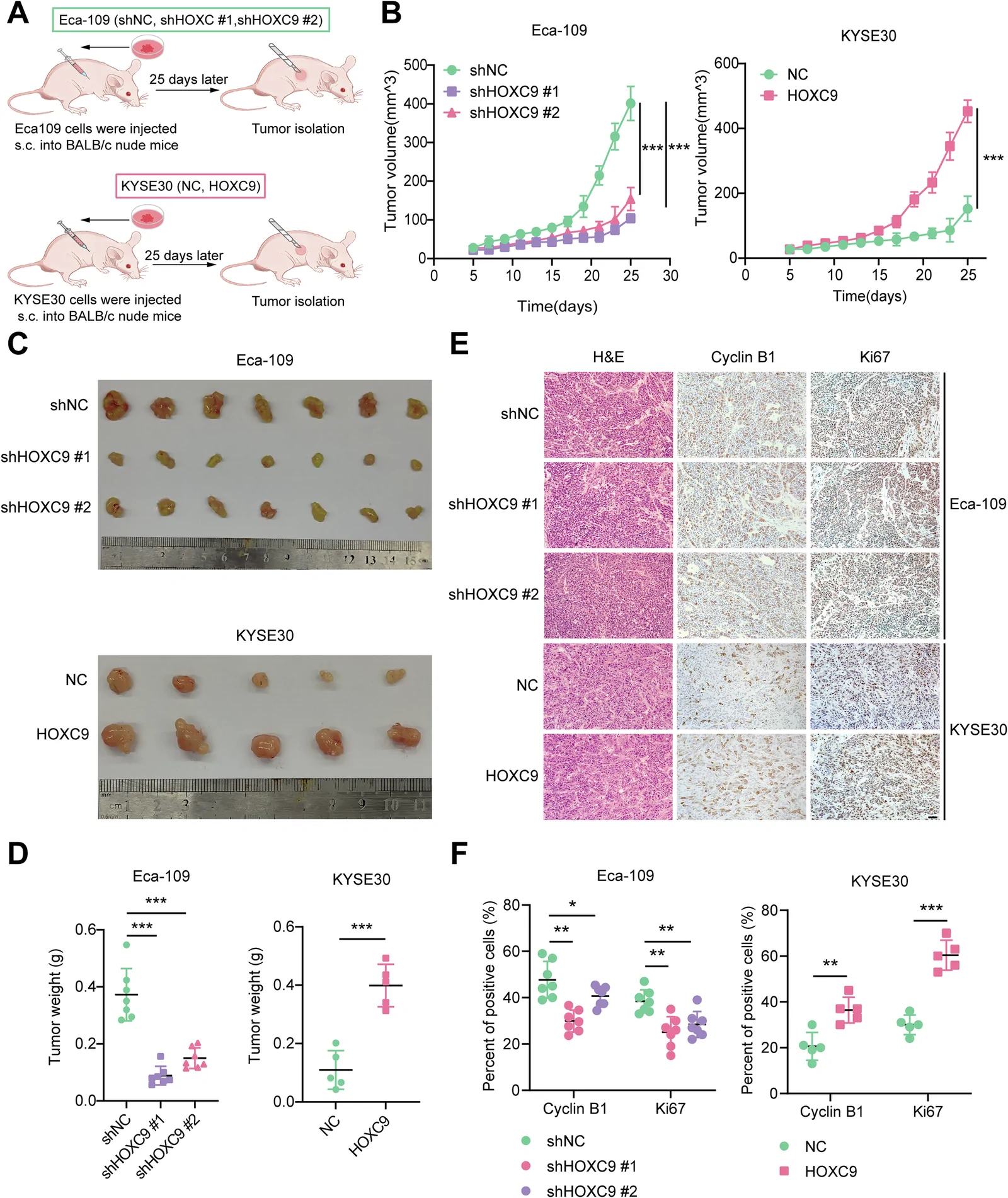
HOXC9 accelerates the growth of ESCC in vivo. **A** Eca-109 cells were transduced with control lentivirus (shNC) and HOXC9 shRNA lentivirus (shHOXC9#1, shHOXC9#2). KYSE30 cells were transduced with empty lentivirus (NC) and HOXC9-overexpressing lentivirus (HOXC9). 5 × 10^6^ Eca-109 cells (shNC, shHOXC9#1, shHOXC9#2, *n* = 7) and KYSE30 cells (NC, HOXC9, *n* = 5) were injected subcutaneously into BALB/c nude mice. Tumor volume was measured every two days. At day 25, the subcutaneous xenograft tumors were isolated for further analysis. Tumor growth curves (**B**), representative tumor images and tumor weights (**C**, **D**) were shown. **E, F** Representative images of H&E and IHC staining of Cyclin B1 and Ki-67 in different group of xenograft tumors were shown. Scale bar, 100 μm. Data are shown as means ± SD and statistical significance was determined by one-way ANOVA in Eca-109 cells and unpaired t-test in KYSE30 cells for (**B**, **D**, **F**). *p < 0.05; **p < 0.01; ***p < 0.001.

To further evaluate the function of HOXC9 in ESCC progression in vivo, Eca-109 cells (shNC, shHOXC9#1, and shHOXC9#2) and KYSE30 cells (NC and HOXC9) were subcutaneously inoculated into BALB/c nude mice to establish xenograft models (Fig. 3A). Compared with shNC group, HOXC9 knockdown significantly reduced tumor volume and tumor weight in Eca-109 cells (Fig. 3B–D). Moreover, IHC analysis revealed decreased Cyclin B1 and Ki67 expression in tumors derived from shHOXC9#1 and shHOXC9#2 groups compared with the shNC group (Fig. 3E, F). In contrast, HOXC9 overexpression in KYSE30 cells markedly accelerated tumor growth (Fig. 3B–D). Correspondingly, HOXC9 overexpression was associated with higher Cyclin B1 and Ki67 levels than the NC group (Fig. 3E, F).

Collectively, these findings demonstrate that HOXC9 plays a vital role in promoting ESCC progression.

### HOXC9 transcriptionally activates OTUD1 in ESCC cells

**Fig. 4:**
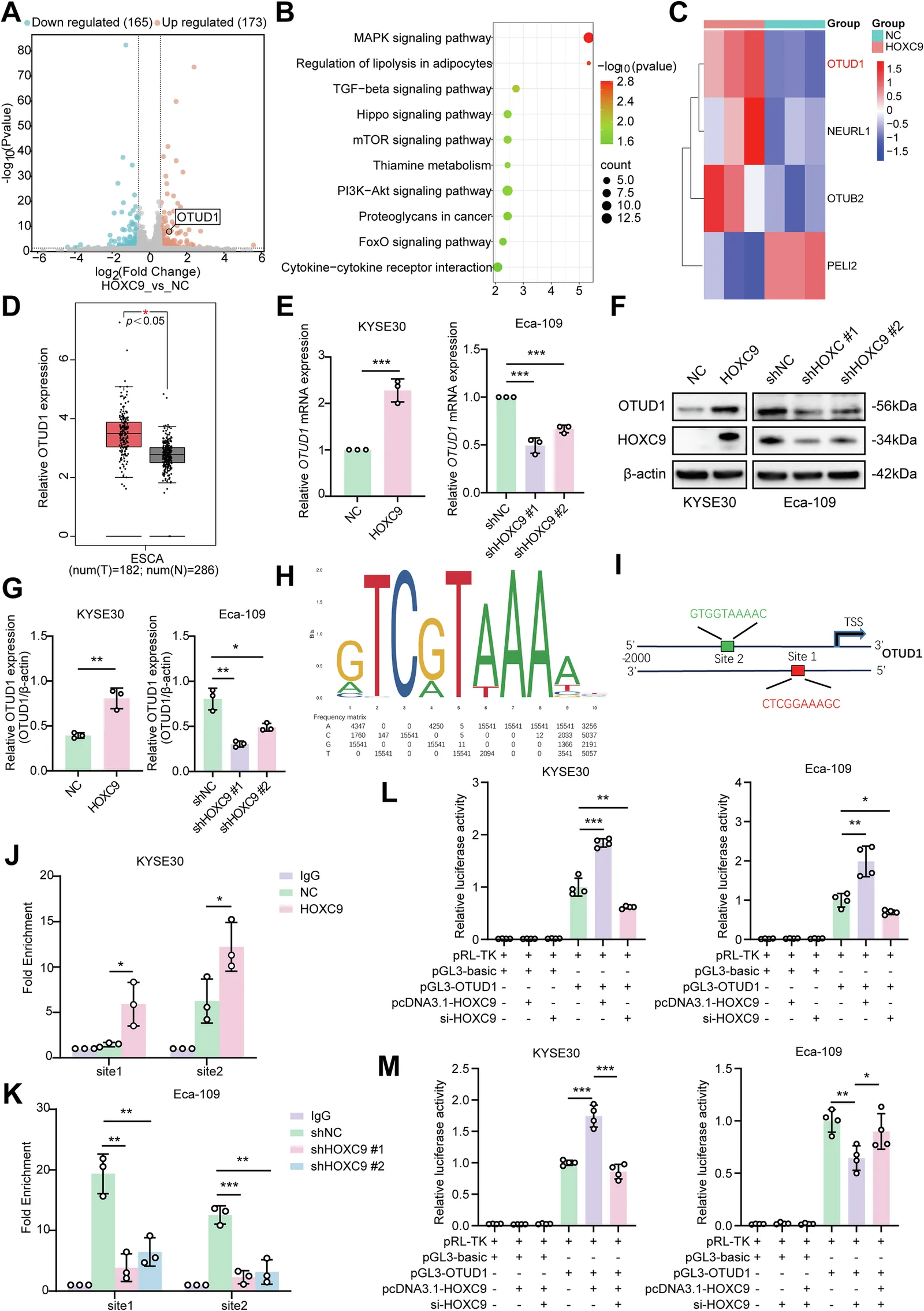
HOXC9 directly binds to the *OTUD1* promoter to upregulate OTUD1 expression. **A**–**C** RNA-seq (GSE301253) was conducted in KYSE30 cells transduced with empty lentivirus (NC) and HOXC9-overexpressing lentivirus (HOXC9). Volcano plot (**A**) and KEGG analysis (**B**) of differentially expressed genes in KYSE30 (NC, HOXC9) cells (|FC| ≥ 1.5, *p* < 0.05). **C** Heatmap of ubiquitin-associated genes in KYSE30 (NC, HOXC9) cells (|FC | ≥ 1.5, *p* < 0.05). **D** GEPIA analysis of OTUD1 expression in normal and ESCC tissues (T tumor tissues, N normal tissues). **E**–**G** Eca-109 cells were transduced with control lentivirus (shNC) and HOXC9 shRNA lentivirus (shHOXC9#1, shHOXC9#2). KYSE30 cells were transduced with empty lentivirus (NC) and HOXC9-overexpressing lentivirus (HOXC9). OTUD1 expression in Eca-109 (shNC, shHOXC9#1, shHOXC9#2) and KYSE30 (NC, HOXC9) cells was tested by RT-qPCR (**E**) and western blot (**F**, **G**). **H**, **I** The binding site of HOXC9 on the *OTUD1* promoter was predicted using the JASPAR database. **J**, **K** The enrichment of HOXC9 in the *OTUD1* promoter region in Eca-109 (shNC, shHOXC9#1, shHOXC9#2) and KYSE30 (NC, HOXC9) cells was determined by ChIP-PCR. **L**, **M** KYSE30 and Eca-109 cells were co-transfected with pGL3-basic or pGL3-*OTUD1* plasmids, HOXC9-overexpressing pcDNA3.1 plasmid (pcDNA3.1-HOXC9) or si-HOXC9 together with pRL-TK for 2 days, and luciferase activities were measured. The experiments were repeated three times (**E**–**G**, **J**, **K**) or twice in duplicates (**L**, **M**). Data are shown as the mean ± SD. For **E**, **G**, statistical significance was determined by unpaired t‑test in KYSE30 cells and one‑way ANOVA in Eca‑109 cells. For **J**–**M**, statistical comparisons were performed using one‑way ANOVA. *p < 0.05; **p < 0.01; ***p < 0.001.

To elucidate the molecular mechanism underlying how HOXC9 promotes ESCC development, RNA sequencing (GSE301253) was performed in KYSE30 cells (NC, HOXC9) and 173 upregulated genes and 165 downregulated genes were identified (Fig. 4A). KEGG analysis revealed that the differentially expressed genes were associated with tumor progression pathways, including the MAPK signaling pathway, lipolysis and the TGF-beta signaling pathway, further indicating that HOXC9 plays a critical role in ESCC development (Fig. 4B). Accumulating evidence indicates that ubiquitinating enzymes and DUBs are closely associated with cancer development because they regulate protein stability, localization and activity [18–20]. We further analyzed the RNA sequencing data (GSE301253) to screen ubiquitin-associated genes, including OTUD1, neuralized homolog 1(NEURL1), ovarian tumor deubiquitinase 2 (OTUB2), and pellino E3 ubiquitin protein ligase 2 (PELI2), among which OTUD1 was the top-ranked differentially expressed HOXC9 target in ESCC (Fig. 4C). Furthermore, GEPIA analysis showed that OTUD1 expression was significantly upregulated in ESCC tissues compared with adjacent normal tissues (Fig. 4D). OTUD1 regulates tumor cell migration and stemness through ubiquitination in various types of cancer, such as ovarian [22] and breast cancer [21]. To explore the function of OTUD1 in ESCC, we knocked down OTUD1 in Eca-109 cells and overexpressed OTUD1 in KYSE30 cells (Fig. S3A, B). CCK-8 (Fig. S3C), EdU (Fig. S3D), and colony formation (Fig. S3E) assays demonstrated that OTUD1 knockdown significantly suppressed cell proliferation, whereas OTUD1 overexpression markedly enhanced ESCC cell growth. Additionally, wound-healing assays revealed that OTUD1 knockdown inhibited Eca-109 cell migration, whereas OTUD1 overexpression significantly promoted KYSE30 cell migration (Fig. S3F).

To investigate the underlying mechanism through which HOXC9 regulates OTUD1, we first conducted RT-qPCR and western blot in KYSE30 cells (NC, HOXC9) and Eca-109 cells (shNC, shHOXC9#1, shHOXC9#2). HOXC9 overexpression significantly increased OTUD1 mRNA and protein expression, whereas HOXC9 knockdown decreased OTUD1 levels (Fig. 4E–G). As a transcription factor, HOXC9 can regulate the transcription of downstream genes. Analysis using the JASPAR database and ChIP-qPCR demonstrated that HOXC9 overexpression significantly increased its binding affinity to the *OTUD1* promoter in KYSE30 cells, whereas knockdown of HOXC9 markedly reduced the enrichment of HOXC9 in the *OTUD1* promoter (Fig. 4H–K). In addition, dual-luciferase reporter assays showed that luciferase activity driven by the *OTUD1* promoter was enhanced in HOXC9-overexpressing KYSE30 and Eca-109 cells compared with control cells, whereas HOXC9 silencing markedly suppressed the luciferase activity driven by the *OTUD1* promoter in ESCC cells (Fig. 4L). Moreover, the enhanced luciferase activity driven by the OTUD1 promoter in HOXC9-overexpressing KYSE30 cells was attenuated by HOXC9 silencing, and HOXC9 overexpression reversed the suppressed luciferase activity in HOXC9-silenced Eca-109 cells (Fig. 4M).

Taken together, these findings indicate that *HOXC9* transcriptionally regulates *OTUD1*, promoting ESCC cell growth.

### OTUD1 mediates FABP5 deubiquitination to stabilize FABP5 in ESCC cells

**Fig. 5:**
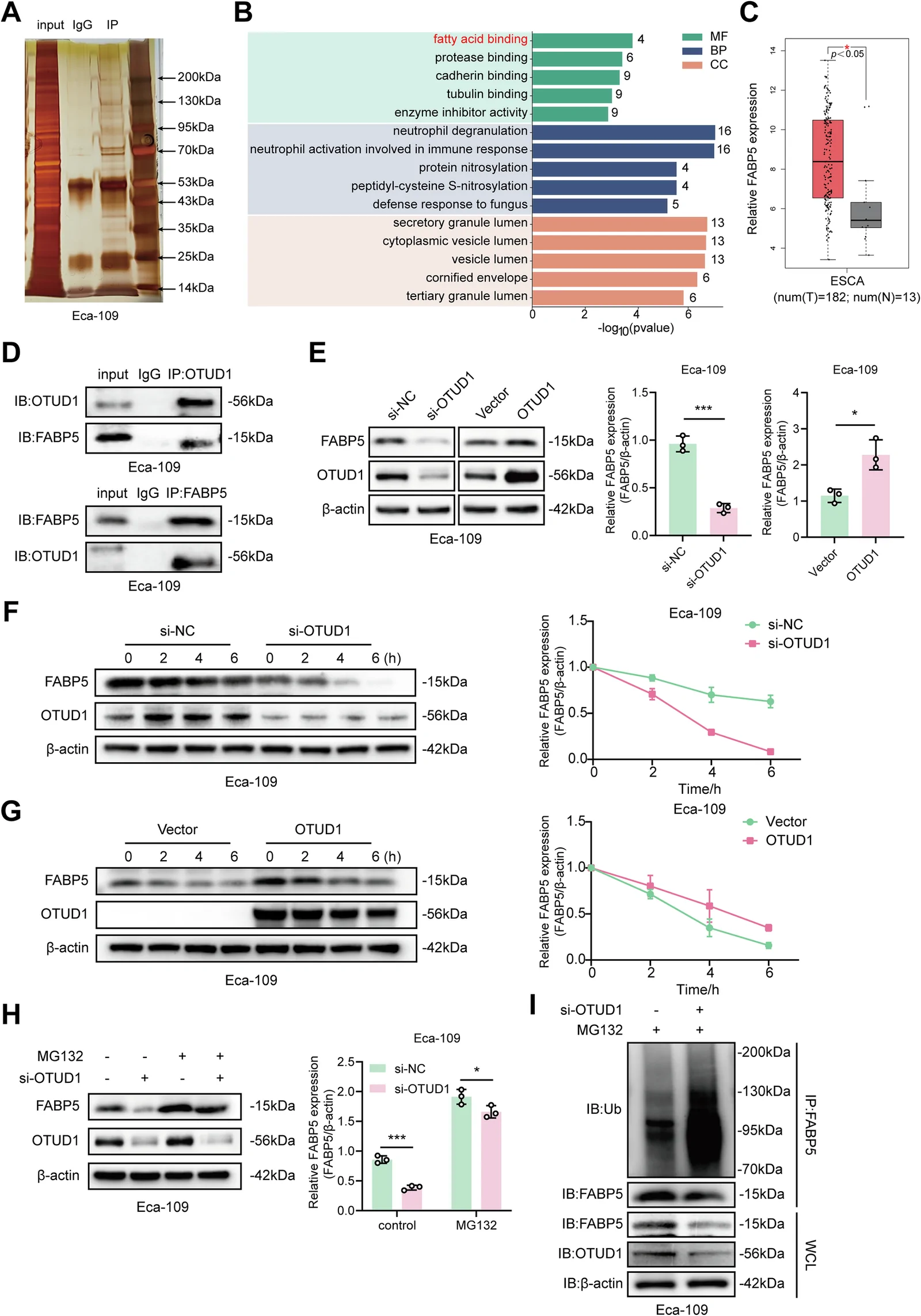
OTUD1 interacts with FABP5 to stabilize FABP5 protein via deubiquitination. **A** Cell lysates were immunoprecipitated using anti-OTUD1 and IgG antibody, and the precipitated proteins were visualized using silver staining. **B** GO analysis of the potential OTUD1-binding protein obtained from LC-MS/MS. **C** GEPIA analysis of FABP5 expression in normal and ESCC tissues (T tumor tissues, N normal tissues). **D** The interaction of OTUD1 and FABP5 in Eca-109 cells was determined by Co-IP. **E** Eca-109 cells were transfected with control siRNA (si-NC) and OTUD1 siRNA (si-OTUD1), as well as empty plasmid (Vector) and OTUD1-overexpressing plasmid (OTUD1). OTUD1 and FABP5 protein expression in Eca-109 cells (si-NC and si-OTUD1, Vector and OTUD1) was analyzed by western blot. **F**, **G** OTUD1 and FABP5 expression in Eca-109 cells (si-NC and si-OTUD1, Vector and OTUD1) treated with cycloheximide (CHX, 400 µg/ml) for the indicated time was measured by western blot. **H** FABP5 expression in Eca-109 cells (si-NC, si-OTUD1) treated with or without MG132 (20 µM, 8 h) were analyzed by western blot. **I** FABP5 ubiquitination in Eca-109 cells (si-NC, si-OTUD1) treated with MG132 (20 µM, 8 h) was determined by Co-IP assays. The experiments (**D**–**I**) were repeated three times. Data are shown as the mean ± SD and determined by unpaired t-test (**E**) or two-way ANOVA (**H**). *p < 0.05; ***p < 0.001.

To further investigate the mechanism by which OTUD1 regulates ESCC cells, we performed IP and LC-MS/MS in Eca-109 cells (Fig. 5A). GO analysis of the 151 identified potential OTUD1-interacting proteins revealed that fatty acid binding was highly enriched (Fig. 5B). Among these, FABP5 was positively correlated with fatty acid synthase (FASN) and acetyl-CoA carboxylase 1 (ACACA) and negatively correlated with carnitine palmitoyltransferase-1A (CPT1A), further suggesting the potential role of FABP5 in ESCC lipid metabolism (Fig. S4A–C). Furthermore, FABP5 expression was significantly upregulated in ESCC tissues compared with adjacent normal tissues (Fig. 5C). Kaplan–Meier plotter analysis showed that ESCC patients with higher FABP5 expression exhibited a poorer prognosis (Fig. S4D). ROC curve analysis further indicated excellent diagnostic efficacy of FABP5 for ESCC patients (AUC = 0.737; 95% CI: 0.560–0.881; Fig. S4E).

Next, we explored how OTUD1 regulates FABP5. Co-IP experiments using anti-OTUD1 and anti-FABP5 antibodies showed that OTUD1 could interact with FABP5 in Eca-109 cells (Fig. 5D). Western blot analysis revealed that compared with the si-NC group, the OTUD1 knockdown significantly shortened the half-life of FABP5, leading to decreased FABP5 protein levels in Eca-109 cells (Fig. 5E, F). Conversely, OTUD1 overexpression inhibited FABP5 degradation and upregulated FABP5 expression comparing with Vector group (Fig. 5E, G). OTUD1 can recognize and remove ubiquitin molecules conjugated to proteins, thereby regulating protein stability and function [21, 22]. Notably, compared with the untreated group, treatment with the proteasome inhibitor MG132 led to an accumulation of FABP5 protein in Eca-109 cells, while the increase in FABP5 protein levels induced by proteasome inhibition was only partially reduced by OTUD1 knockdown (Fig. 5H). We subsequently performed IP assays and found that OTUD1 knockdown significantly promoted FABP5 ubiquitination in OTUD1-knockdown cells (Fig. 5I).

Taken together, our data suggest that OTUD1 upregulates FABP5 expression by deubiquitinating FABP5 protein.

### HOXC9 upregulates FABP5 expression via OTUD1 to remodel lipid metabolism and promote ESCC progression

**Fig. 6:**
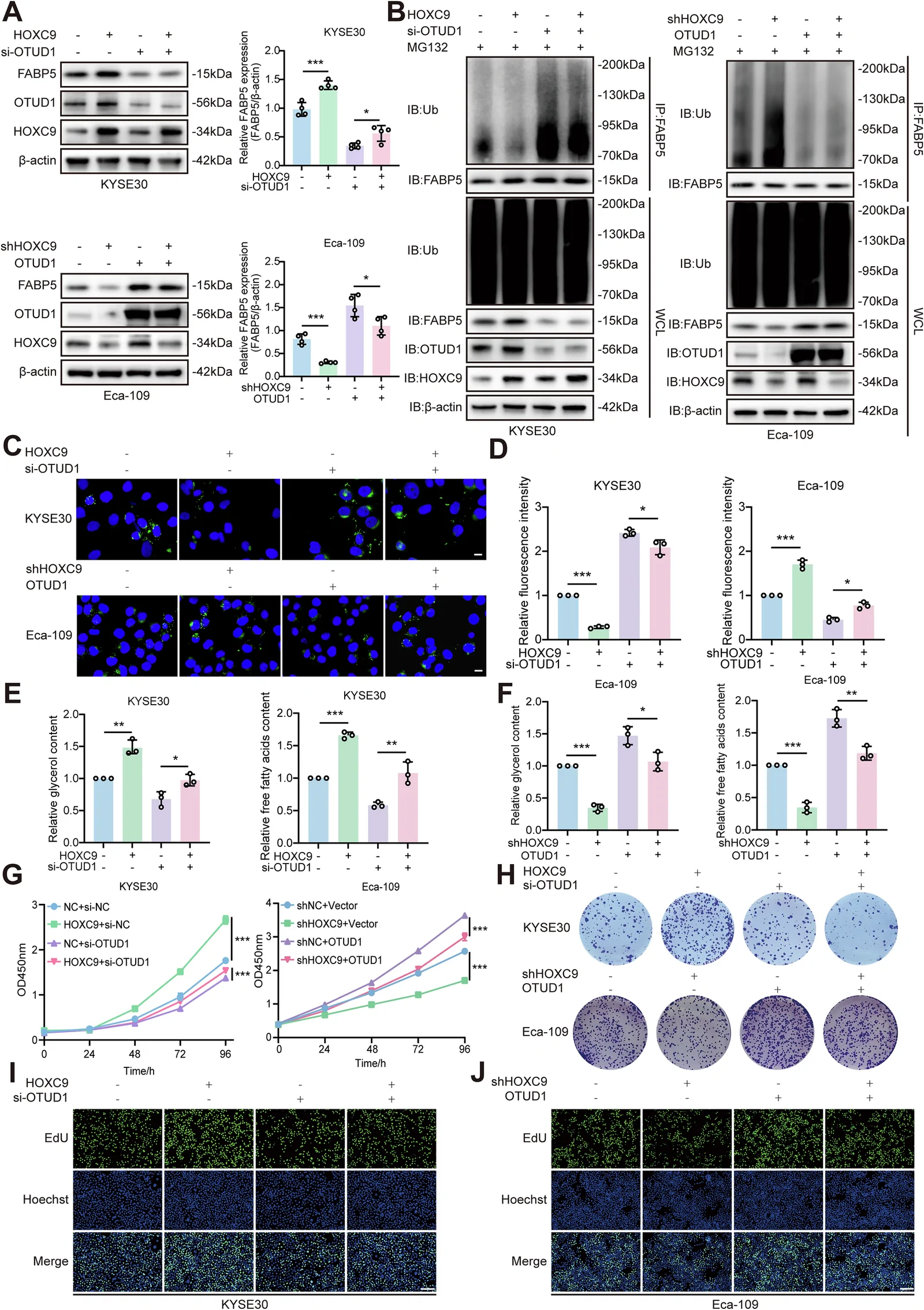
HOXC9 upregulates FABP5 expression via OTUD1 to remodel lipid metabolism and promote ESCC cell proliferation. KYSE30 (NC, HOXC9) cells were transfected with control siRNA (si-NC) and OTUD1 siRNA (si-OTUD1). Eca-109 (shNC, shHOXC9) cells were transfected with empty plasmid (Vector) and OTUD1-overexpressing plasmid (OTUD1). **A** HOXC9, OTUD1, and FABP5 protein expression in different groups of KYSE30 and Eca-109 cells was detected by western blot. **B** FABP5 ubiquitination in different groups of KYSE30 and Eca-109 cells treated with MG132 (20 µM, 8 h) was determined by Co-IP assays. Representative BODIPY 493/503 staining images (**C**) and relative BODIPY 493/503 fluorescence intensity (**D**) in different groups of KYSE30 and Eca-109 cells are shown. Green, BODIPY 493/503; Blue, DAPI. Scale bar, 25 μm. **E**, **F** Glycerol and free fatty acid levels in different groups of KYSE30 and Eca-109 cells was determined. The proliferation in different groups of KYSE30 and Eca-109 cells was detected by CCK-8 assays (**G**), colony-formation assays (**H**) and EdU assays (**I**, **J**). Scale bar, 200 μm (EdU assays). The experiments were repeated three (**B**–**J**) or four times (**A**). Data are shown as the mean ± SD and determined by two-way ANOVA. *p < 0.05; **p < 0.01; ***p < 0.001.

OTUD1 has been increasingly recognized as a critical regulator of tumorigenesis through its deubiquitinating activity [21, 22]. To determine whether HOXC9 upregulates FABP5 expression via OTUD1, we conducted cycloheximide (CHX) chase assays. We found that compared with shNC group, HOXC9 knockdown significantly shortened the half-life of FABP5 protein in Eca-109 cells, leading to decreased FABP5 protein levels without affecting *FABP5* mRNA expression (Fig. S5A–C). Consistent with these findings, HOXC9 overexpression enhanced FABP5 protein stability and increased FABP5 expression in KYSE30 cells (Fig. S5B, D). Co-transfection of HOXC9-overexpressing lentivirus and siRNA-OTUD1 in KYSE30 and Eca-109 cells, as well as HOXC9 shRNA and OTUD1-overexpressing plasmid in Eca-109 and KYSE510 cells, showed that OTUD1 knockdown significantly inhibited HOXC9-mediated upregulation of FABP5 by inducing FABP5 ubiquitination (Figs. 6A, B and S6A). Moreover, overexpression of OTUD1 markedly reversed the reduction in FABP5 expression caused by HOXC9 knockdown by enhancing FABP5 deubiquitination in ESCC cells (Figs. 6A, B and S7A). In addition, near-complete inhibition of OTUD1 using shRNA blocked the effect of HOXC9 overexpression and knockdown on FABP5 protein levels (Fig. S8). These data suggest that HOXC9 upregulates FABP5 expression via OTUD1.

**Fig. 7:**
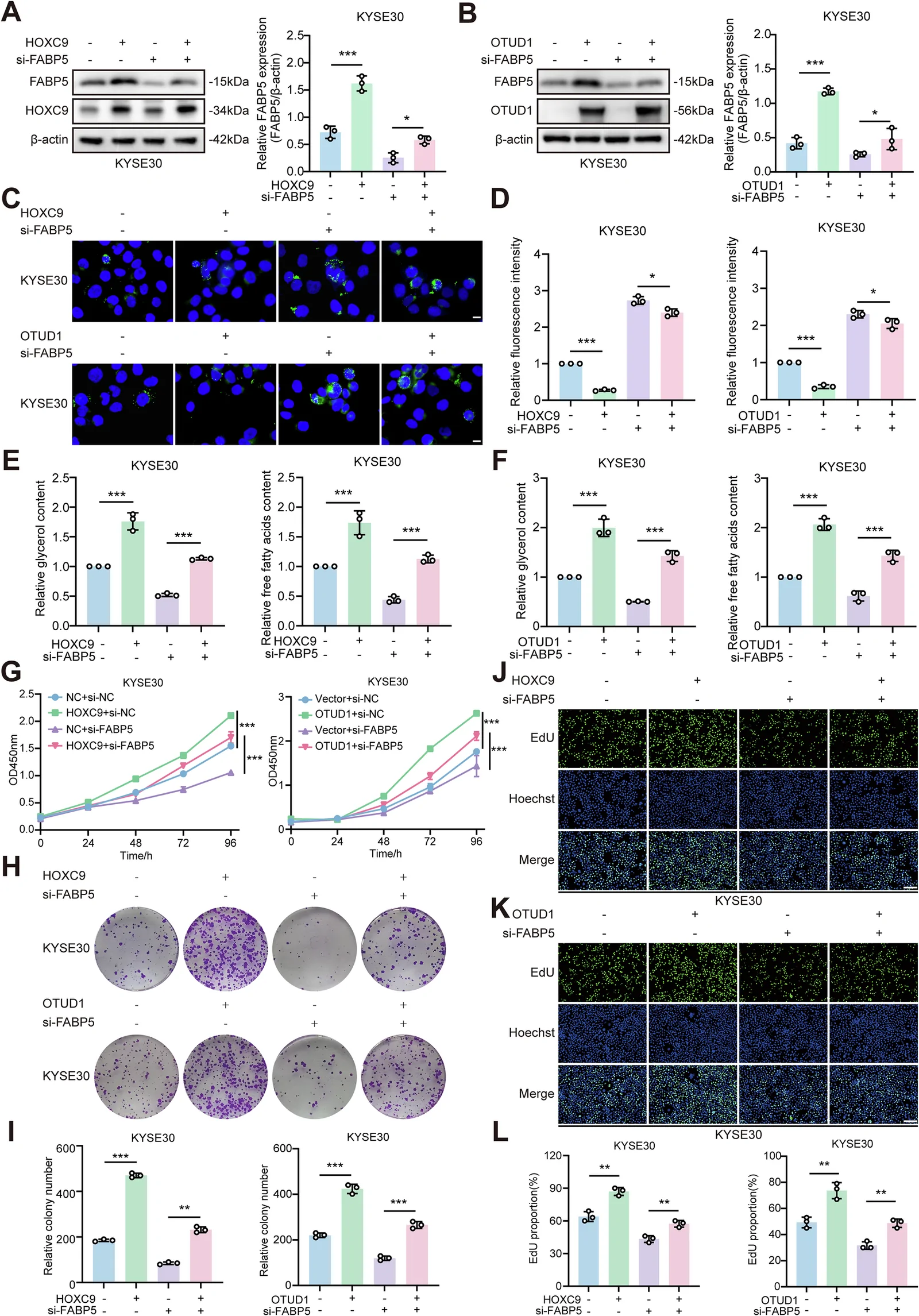
Inhibition of FABP5 restrains the tumor-promoting effects of HOXC9 and OTUD1 in ESCC cells. **A** KYSE30 (NC, HOXC9) cells were transfected with control siRNA (si-NC) and FABP5 siRNA (si-FABP5). HOXC9 and FABP5 protein expression in different groups of KYSE30 cells was tested by western blot. **B** KYSE30 (Vector, OTUD1) cells were transfected with control siRNA (si-NC) and FABP5 siRNA (si-FABP5). OTUD1 and FABP5 protein levels in different groups of KYSE30 cells were tested by western blot. Representative BODIPY 493/503 staining images (**C**) and relative BODIPY 493/503 fluorescence intensity (**D**) in different groups of KYSE30 cells are shown. Green, BODIPY 493/503; Blue, DAPI. Scale bar, 25 μm. **E**, **F** Glycerol and free fatty acid levels in different groups of KYSE30 cells. The proliferation in different groups of KYSE30 cells was tested by CCK-8 assays (**G**), colony formation assays (**H**, **I**) and EdU assays (**J**–**L**). Scale bar, 200 μm (EdU assays). All experiments were repeated three times. Data are shown as the mean ± SD and determined by two-way ANOVA. *p < 0.05; **p < 0.01; ***p < 0.001.

We next investigated the effect of the HOXC9-OTUD1-FABP5 axis on lipid metabolism in ESCC cells. BODIPY 493/503 and Oil Red O staining revealed that HOXC9 overexpression reduced lipid droplet accumulation and increased glycerol and free fatty acid levels, whereas OTUD1 knockdown significantly restrained the increased level of lipolysis caused by HOXC9 overexpression (Figs. 6C–F, S6B–D, and S9A, B). On the other hand, HOXC9 knockdown markedly increased lipid droplet accumulation and reduced glycerol and free fatty acid levels, effects that were partially reversed by OTUD1 overexpression (Figs. 6C–F, S7B–D, and S9A, B). Lipid metabolism is a crucial indicator of tumor progression [24, 26]. Next, we aimed to identify the role of the HOXC9-OTUD1-FABP5 axis in ESCC progression. CCK-8, colony formation, EdU, and wound-healing assays comprehensively indicated that OTUD1 knockdown significantly suppressed the proliferative and migratory ability induced by HOXC9 overexpression (Figs. 6G–J, S6E–K, and S9C–F). Conversely, OTUD1 overexpression could also rescue impaired cell viability and migration ability in HOXC9-silenced ESCC cells (Figs. 6G–J, S7E–K, and S9C–F). Furthermore, we transfected FABP5 siRNA into HOXC9- or OTUD1-overexpressing KYSE30 cells (Fig. 7A, B). Compared with the control group, silencing FABP5 significantly blocked the increase in lipolysis and enhanced proliferative ability in both HOXC9- and OTUD1-overexpressing KYSE30 cells (Figs. 7C–L and S10A, B), further supporting the notion that FABP5 is a necessary and sufficient downstream effector linking HOXC9-OTUD1 signaling to lipid metabolism and growth phenotypes.

Taken together, these data indicate that HOXC9 upregulates FABP5 expression via OTUD1 to remodel lipid metabolism, thereby accelerating ESCC progression.

### HOXC9, OTUD1, and FABP5 are positively correlated with pathological grade in ESCC patients

**Fig. 8:**
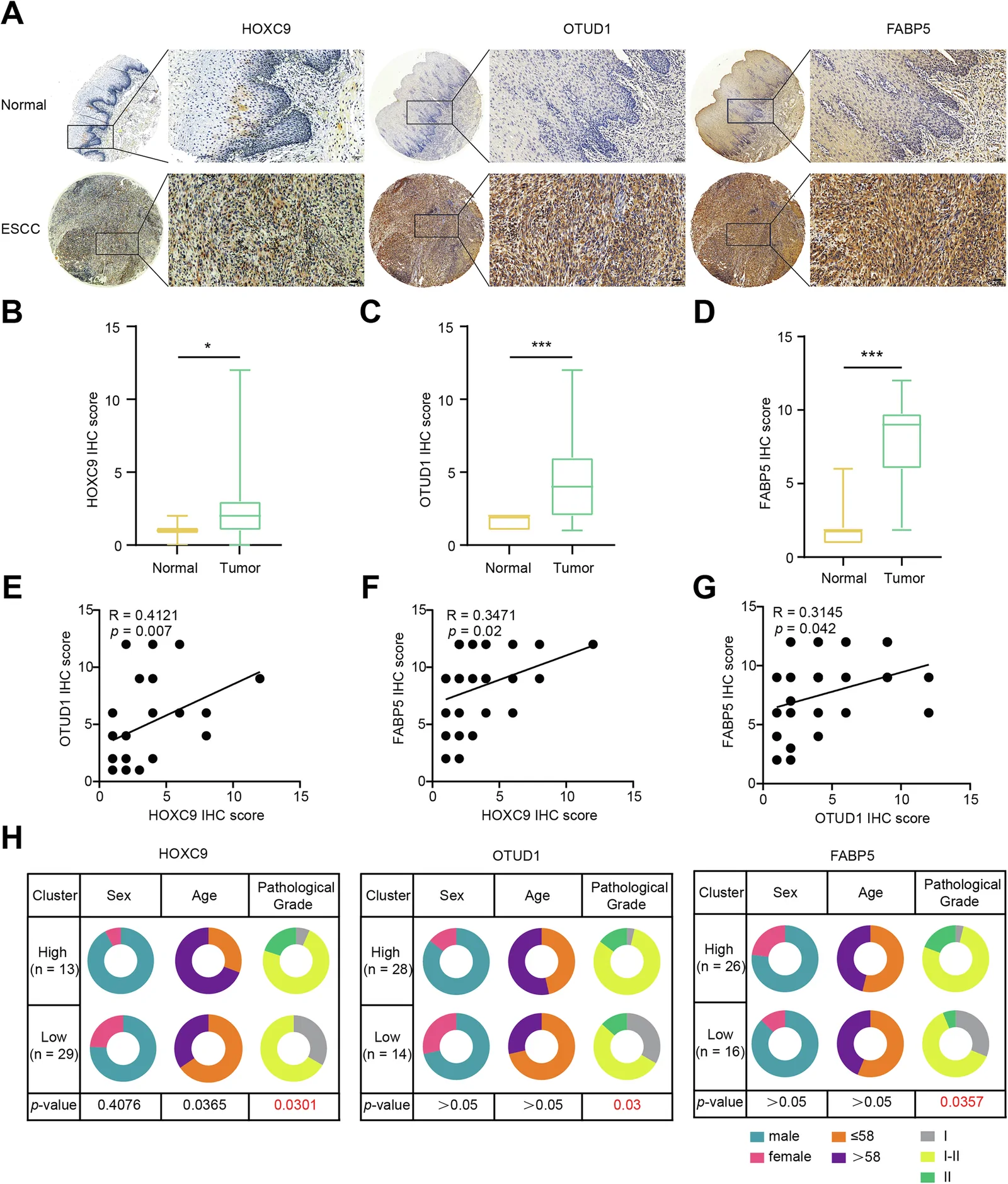
HOXC9, OTUD1, and FABP5 are positively correlated with pathological grade in ESCC patients. **A**–**D** Representative images of IHC staining of HOXC9, OTUD1, and FABP5 in tissue microarrays containing ESCC tissues (*n* = 42) and adjacent tissues (*n* = 15). Scale bar, 50 μm. **E**–**G** Correlation analysis of HOXC9, OTUD1 and FABP5 IHC scores in ESCC tissues (*n* = 42). **H** Correlation of HOXC9, OTUD1, and FABP5 IHC scores with clinicopathological features of ESCC patients (*n* = 42). Data are shown as the mean ± SD and determined by unpaired t-test (**B**–**D**), Pearson correlation analysis (**E**–**G**) and chi-square test (**H**). *p < 0.05; ***p < 0.001.

To further evaluate the clinical relevance of HOXC9, OTUD1, and FABP5 in ESCC, IHC staining was performed on ESCC tissue microarrays containing ESCC tissues (*n* = 42) and adjacent normal tissues (*n* = 15). HOXC9, OTUD1, and FABP5 were expressed at significantly higher levels in ESCC tissues than in adjacent normal tissues (Fig. 8A–D), with a positive correlation observed among them (Fig. 8E–G). More importantly, elevated tumor HOXC9, OTUD1, and FABP5 expression was positively associated with higher pathological grade in ESCC patients (*p* = 0.0301 for HOXC9; *p* = 0.0300 for OTUD1; *p* = 0.0357 for FABP5**;** Fig. 8H). Collectively, these findings indicate that HOXC9/OTUD1/FABP5 signaling holds considerable promise as a diagnostic biomarker for ESCC patients.

## Discussion

ESCC remains a lethal disease with unsatisfactory clinical outcomes. Intensive investigations of the molecular mechanisms underlying ESCC progression are urgently needed to develop accurate diagnostic markers and promising therapeutic strategies for better clinical efficacy. In this study, we demonstrated that elevated HOXC9 expression is positively associated with poor prognosis in ESCC patients. Mechanistically, HOXC9 directly binds to *OTUD1* promoter regions to activate OTUD1 transcription, which subsequently deubiquitinates FABP5 to promote lipid metabolism and thus accelerates the malignant phenotype of ESCC. Our findings identify potential biomarkers and therapeutic targets for the pathogenesis and clinical treatment of ESCC.

As a member of the HOX family, HOXC9 has been increasingly recognized as a diagnostic biomarker and contributes to poor prognosis in patients with various malignancies, including colorectal cancer [33], gastric cancer [15], and lung adenocarcinoma [17]. Here, we report that HOXC9 is overexpressed in ESCC and positively associated with higher pathological grade and shorter survival in ESCC patients, further supporting the clinical diagnostic value of HOXC9. Transcription factors are key regulators of intrinsic cellular processes, such as differentiation and development. Recent studies have provided novel insights into the tumor-promoting effects of HOXC9. For example, HOXC9 knockdown relieved its transcriptional inhibition of DAPK1, thereby activating the DAPK1-Beclin1 pathway, which induces autophagy in glioblastoma cells [34]. Our work revealed that HOXC9 transcriptionally activates its downstream target OTUD1, which stabilizes FABP5, thereby enhancing lipid metabolism and thus promoting ESCC cell proliferation and migration. These findings further illustrate the vital role of HOXC9 as an oncogene in tumor progression. Although we clarified that HOXC9 directly binds to the *OTUD1* promoter to upregulate its expression using ChIP-qPCR and dual luciferase reporter assay, reporting the promoter coordinates using site-directed mutant constructs in the *OTUD1* promoter would provide sequence-specific validation complementary to the ChIP-qPCR results and would require further investigation.

Deubiquitinating enzymes can regulate cellular metabolism [35, 36], proliferation [37–39], and differentiation [40] by modulating protein degradation and stabilization and are closely associated with the occurrence and development of various tumors. For example, ubiquitin-specific peptidase 14 (USP14) promotes tryptophan metabolism and T-cell dysfunction by stabilizing indoleamine 2,3-dioxygenase 1 (IDO1), thereby accelerating colorectal cancer progression [36]. Tripartite motif-containing 47 (TRIM47) facilitates K48-linked ubiquitination of cylindromatosis (CYLD) to induce the degradation of CYLD, thereby activating the NF-κB pathway and accelerating the tumor growth of gastric cancer [38]. Ubiquitin-specific peptidase 10 (USP10) interacts with anilin (ANLN) and removes K11- and K63-linked ubiquitin chains of ANLN, preventing ANLN ubiquitin-mediated degradation to drive ESCC progression [39]. Zhang et al. reported that OTUD1 directly deubiquitinates SMAD family member 7 (SMAD7) to prevent its degradation and alleviate the TGF-β-induced pro-oncogenic response, thereby inhibiting breast cancer metastasis [21]. In our study, we identified OTUD1 as an oncogenic factor in ESCC that accelerates ESCC cell proliferation and migration by deubiquitinating FABP5, thereby enhancing lipid metabolism. To more conclusively support the tumor-promoting role of the OTUD1-FABP5 axis, we need to further apply OTUD1 catalytic-dead mutants (e.g., Cys-to-Ala) that cannot stabilize FABP5 for tumor proliferation assays in ESCC cells. On the other hand, OTUB1, another DUB of the OTU subfamily, can deubiquitinate MYC, thereby blocking MYC protein degradation and inducing hexokinase 2 (HK2), which leads to enhanced aerobic glycolysis and promotes breast tumorigenesis [41]. Metabolic intermediates play critical roles in modulating signal transduction and gene expression by chemically modifying proteins. In the future, identifying the direct roles of OTUD1 in regulating metabolic rewiring and related chemical modifications in different tumor cells will provide a comprehensive understanding of the relationships among OTUD1, posttranslational modification, and tumor progression.

Lipid metabolism generally provides the energy and cellular metabolites necessary for the proliferation and invasion of cancer cells [24–26]. As a member of the fatty acid-binding protein (FABP) family, FABP5 mediates progression of various tumors by regulating lipid metabolism [27–29, 42]. FABP5 expression is downregulated in colorectal cancer (CRC), where it interacts with fatty acid synthase (FASN) and activates the ubiquitin-proteasome pathway, leading to decreased FASN expression and lipid accumulation, thereby inhibiting CRC [42]. In contrast, FABP5 promotes lipolysis and the synthesis of glycerol and free fatty acids, thereby promoting lymph node metastasis in patients with cervical cancer [29]. Here, we report that elevated tumor FABP5 levels are positively correlated with poor prognosis in ESCC patients, underscoring its role in regulating ESCC progression. Given the key metabolic checkpoint role of lipids, multiple strategies, such as FASN inhibitors [43] and immunotherapy [44], have been combined to modulate lipid metabolism/signaling within tumors to improve various therapeutic strategies. More importantly, HOXC9 serves as a biomarker to amplify the predictive efficacy of immune checkpoint inhibitors plus chemotherapy in lung adenocarcinoma [17]. OTUD1 blockade has been shown to overcome tumor immune checkpoint resistance and to provide synergy with immune checkpoint blockade (ICB) therapy [45, 46]. Our work provides proof of concept that blockade of the HOXC9-OTUD1-FABP5 axis may be a potentially effective therapeutic approach for ESCC. However, the limited detailed follow-up data for ESCC tissue microarrays were insufficient to conduct Kaplan-Meier survival analysis and multivariable Cox regression analysis. In the future, we need to collect more ESCC tissue samples and follow-up data to supplement and conduct relevant diagnostic and prognostic analyses.

In summary, we identified HOXC9 as a potential oncogenic factor that promotes OTUD1-mediated lipid metabolism by deubiquitinating FABP5, thereby accelerating ESCC cell proliferation and migration. This study provides a strong scientific rationale for the HOXC9-OTUD1-FABP5 axis as a prospective diagnostic biomarker for patients with ESCC.

## Materials and methods

### Human tissue microarray

Tissue microarrays (HEso-Squ030PG-01 and HEsoS030PG02) containing 42 primary ESCC tissues and 15 adjacent normal tissues were obtained from Shanghai OUTDO BIOTECH Co., Ltd (Shanghai, China).

### Cell culture

The human ESCC cell lines Eca-109, KYSE30, KYSE510, and KYSE180, and human normal esophageal epithelial cells (HEECs) were obtained from the Institute of Cells, Shanghai Academy of Life Sciences. All cell lines were authenticated by short tandem repeat (STR) profiling and tested to be free of mycoplasma contamination. All cell lines were maintained in RPMI-1640 medium (L110KJ; BasalMedia, China) supplemented with 10% fetal bovine serum (FBS; AUS-01S-02; Cell-Box, China) and 1% penicillin-streptomycin (P1400; Solarbio, China) in a humidified atmosphere containing 5% CO_2_ at 37 °C.

### Lentivirus infection, small interfering RNAs (siRNAs), and plasmid transfection

Eca-109, KYSE510, and KYSE30 cells were transduced with lentiviral particles encoding HOXC9 or OTUD1 (GeneChem, China) and selected with puromycin (P8230; Solarbio) for 4 weeks to generate stable cell lines. Eca-109, KYSE510, and KYSE30 cells were transfected with HOXC9- and OTUD1-overexpressing pcDNA3.1 plasmids (GeneChem, China) or siRNAs targeting HOXC9, OTUD1, and FABP5 using Lipofectamine 2000 (11668019; Invitrogen, USA) or RNATransMate (E607402; Sangon, China), respectively, as described by the manufacturer. The sequences of the shRNAs and siRNAs are presented in Table S1.

### RT-qPCR

Total RNA was extracted using TRIzol reagent (CW0580; CWBIO, China). Reverse transcription to synthesize cDNA was performed with the PrimeScript™ RT reagent Kit (RR037A; TaKaRa, Japan) following the manufacturer’s instructions. RT-qPCR was conducted on a LightCycler 480 system (Roche, Switzerland) using 2× SYBR Green qPCR Premix (KS0601; KEMIX, China) and the corresponding primers (Table S2). Gene expression levels were normalized to *ACTB* and analyzed by the 2^−ΔΔCt^ method.

### Western blot

Cells and tissues were lysed using RIPA lysis buffer (G2002; Servicebio, China) supplemented with protease inhibitors (P1045; Beyotime, China). Protein lysates were separated by sodium dodecyl sulfate-polyacrylamide gel electrophoresis and subsequently transferred onto polyvinylidene fluoride membranes. The membranes were blocked with 5% milk at room temperature for 2 h with gentle agitation, followed by incubation with primary antibodies against HOXC9 (sc-81100; Santa Cruz, USA), OTUD1 (29921-1-AP; Proteintech, China), FABP5 (12348-1-AP; Proteintech), Ubiquitin (80992-1-RR; Proteintech), Bax (50599-2-Ig; Proteintech), Cyclin B1 (CY5378; Abways, China), β-actin (AB0035; Abways), and β-tubulin (M20005; Abmart) at 4 °C overnight and the corresponding secondary antibodies at room temperature for 1 h. Protein signals were visualized using ECL (PK10001; Proteintech). Details of the antibodies used in this study are presented in Table S3. Band intensities were quantified using ImageJ and normalized to those of β-tubulin or β-actin.

### Tumor proliferation assays

For the CCK-8 assays, tumor cells were subjected to various treatments and seeded in 96-well plates at a density of 1500 cells per well. At each time point, the culture medium was removed and replaced with 100 μL of fresh medium containing 10 μL of CCK-8 reagent (C0037; Beyotime), followed by a 2 h incubation at 37 °C. Afterwards, the absorbance (450 nm) was measured.

For the EdU assays, equal numbers of tumor cells from different groups were seeded in 96-well plates and cultured for 24 h. After removing the medium, fresh culture medium containing 10 μM EdU (C0071S; Beyotime) was added to each well, and the cells were incubated for 2 h. The cells were then fixed with 4% paraformaldehyde and treated with 0.5% Triton X-100 for 15 min, followed by green fluorescence labeling according to the manufacturer’s instructions. Nuclei were stained with Hoechst, and images were captured using an inverted fluorescence microscope (Leica DMI3000 B), and the number of EdU-positive cells was quantified with ImageJ.

For colony formation assays, tumor cells were seeded in 12-well plates at a density of 800 cells per well and cultured for 10–14 days. The colonies were fixed with paraformaldehyde, stained with crystal violet (DZ0055; LEAGENE), and quantified using ImageJ.

### Wound healing and Transwell assays

For the wound healing assays, ESCC cells were seeded into 12-well plates. Scratch wounds were created using a pipette tip after the cells reached 90% confluence. The cells were then cultured in serum-free medium and imaged at 0 h and 24 h.

For Transwell assays, 5 × 10^4^ tumor cells were seeded into the upper Transwell chamber (3422; Corning) with 200 μL of serum-free medium, while 600 μL of complete medium containing 20% FBS was added to the lower chamber. After 48 h, the upper chamber was fixed with 4% paraformaldehyde and stained with crystal violet (DZ0055; LEAGENE). Migrated cells were photographed under a microscope and analyzed using ImageJ.

### RNA sequencing

KYSE30 cells were transduced with empty lentivirus (NC group) or HOXC9-overexpressing lentivirus (HOXC9 group). Total RNA was isolated from KYSE30 cells (NC and HOXC9) using TRIzol reagent (CW0580; CWBIO). RNA sequencing (GSE301253) was conducted by Majorbio Biopharm Biotechnology Co., Ltd (Shanghai, China) according to the manufacturer’s guidelines. The data were analyzed using the Majorbio Cloud Platform.

### Chromatin Immunoprecipitation (ChIP)

KYSE30 (NC, HOXC9) and Eca-109 (shNC, shHOXC9#1, shHOXC9#2) cells were cross-linked, lysed, sonicated, and immunoprecipitated overnight with anti-HOXC9 (sc-81100; Santa Cruz) or IgG antibodies (B900620; Proteintech) at 4 °C, followed by washing, elution, and cross-link reversal using a ChIP Assay Kit (P2083S, Beyotime). Finally, the purified DNA was analyzed using a LightCycler 480 system (Roche, Switzerland) with the corresponding primers listed in Table S2.

### Dual luciferase reporter assay

*OTUD1* promoter fragments (−471 bp to −1120 bp relative to the transcription start site) were synthesized by Sangon and then inserted into the pGL3-basic vector (PGL3-*OTUD1*). KYSE30 and Eca-109 cells were cotransfected with pGL3-*OTUD1* or pGL3-basic vector, pRL-TK, and either HOXC9-overexpressing pcDNA3.1 plasmid (pcDNA3.1-HOXC9) or HOXC9 siRNA (si-HOXC9) for 2 days. Luciferase activity was measured using the Dual Luciferase Reporter Gene Assay Kit II (RG029M; Beyotime) with a BioTek Synergy H1 microplate reader, and the firefly/Renilla ratio was quantified.

### Immunoprecipitation

Immunoprecipitation (IP) was performed as follows using Protein A + G Agarose (P2055; Beyotime). Briefly, cells were lysed in ice-cold IP lysis buffer (P0013; Beyotime) containing protease inhibitors (P1045; Beyotime). After preclearing with control IgG-bound beads, lysates were incubated with antibody-conjugated beads overnight at 4 °C. Beads were washed three times with IP lysis buffer, and bound proteins were eluted in 2× SDS-PAGE buffer at 95 °C for 6 min before western blot. The antibodies used in this study are listed in Table S3.

### LC-MS/MS

To identify OTUD1-interacting proteins, total protein extracts from Eca-109 cells were subjected to immunoprecipitation with anti-OTUD1 antibody (29921-1-AP; Proteintech) or rabbit IgG (30,000-0-AP; Proteintech), followed by SDS-PAGE. Gels were stained with a rapid silver staining kit (P0017S; Beyotime Biotechnology) according to the manufacturer’s instructions. Bands of interest were excised, and chromatographic separation was performed using an HPLC system (Easy nLC-1200, Thermo Scientific, USA). Mass spectrometry was performed using a Q-Exactive HF (Thermo Scientific, USA) at Shanghai Bioprofile Biotechnology Co., Ltd.

### Quantification of lipid droplets, glycerol, and free fatty acids

For BODIPY 493/503 staining, cells were seeded on glass coverslips and cultured to approximately 50% confluence. Cells were fixed with 4% paraformaldehyde for 15 min, stained with BODIPY 493/503 (20 μM; HY-W090090; MedChemExpress, USA) for 30 min, and counterstained with 4′,6-diamidino-2-phenylindole (DAPI; 2 μg/mL; HY-D0814, MedChemExpress) for 10 min in the dark. Fluorescence images were captured using a confocal microscope (Zeiss, Germany), and lipid droplet intensity was quantified using ImageJ.

For Oil Red O staining, Oil Red O powder (O0625; Sigma-Aldrich, Germany) was dissolved in isopropanol to prepare the stock solution, and the working solution (stock solution/distilled water = 3:2) was filtered through a 0.22 μm membrane. When the cells reached 90% confluence, they were fixed with 4% paraformaldehyde, stained with working solution for 30 min at room temperature, washed with distilled water, and imaged under an optical microscope.

For measurement of glycerol and free fatty acids, 5 × 10⁶ cells were incubated with detection buffer and homogenized thoroughly on ice. The supernatant was collected by centrifugation (4 °C, 12,000 × *g*, 5 min) for subsequent analyses. Glycerol and free fatty acids were quantified using Amplex Red Glycerol Assay Kit (S0223; Beyotime) and Amplex Red Free Fatty Acid Assay Kit (S0215; Beyotime), respectively, according to the manufacturer’s protocol. Afterwards, the absorbance (570 nm) was measured.

### Xenograft models

5-week-old female BALB/c nude mice were randomly assigned to different experimental groups. Subsequently, Eca-109 cells (shNC, shHOXC9#1, shHOXC9#2; *n* = 7) and KYSE30 cells (NC, HOXC9; *n* = 5) were subcutaneously injected into the dorsal flanks of BALB/c nude mice (5 × 10^6^/mouse). Tumor volumes were measured every two days with Vernier callipers. Twenty-five days later, mice were euthanized for tumor weight measurement. Paraffin-embedded sections were subjected to hematoxylin and eosin (H&E) staining and immunohistochemistry (IHC) analysis. This study was approved by the Ethics Committee of the First Affiliated Hospital of Zhengzhou University (Zhengzhou, China).

### H&E and IHC

Tissue microarrays (HEso-Squ030PG-01 and HEsoS030PG02) were stained with anti-HOXC9 (sc-81100; Santa Cruz), anti-OTUD1 (29921-1-AP; Proteintech), and anti-FABP5 (12348-1-AP; Proteintech) antibodies. IHC scores were independently assessed by two researchers. Briefly, IHC scores were calculated by multiplying the positive staining density score by the range score. The density score was assigned according to the following criteria: 0 for negative; 1 for weak (light brown); 2 for moderate (yellow-brown); and 3 for strong (brown). The range score was determined by the percentage of positively stained cells: 1 for less than 25%; 2 for 26–50%; 3 for 51–75%; and 4 for 76–100%. Median values were used as cutoffs for high and low expression of target proteins. H&E and IHC staining were conducted on tumor tissues isolated from xenograft models that were incubated with anti-Ki67 (28074-1-AP; Proteintech) and anti-Cyclin B1 (CY5378; Abways) antibodies.

### Bioinformatics analysis

ESCC data from the Cancer Genome Atlas (TCGA) were analyzed using R software (version 4.3.1). Heatmaps, volcano plots, Gene Ontology (GO) and Kyoto Encyclopedia of Genes and Genomes (KEGG) analyses were generated by Shanghai NewCore Biotechnology Co., Ltd. HOXC9, OTUD1, and FABP5 expression, as well as the correlation of FABP5 with lipid metabolism-related genes in ESCC, were analyzed using Gene Expression Profiling Interactive Analysis (GEPIA). Kaplan-Meier survival curves were generated using the Kaplan-Meier plotter.

### Statistical analysis

Statistical analyses were performed using GraphPad Prism 9. All data were normally distributed according to the Shapiro-Wilk normality test. Statistical comparisons between two independent groups were analyzed using unpaired t-test, while ANOVA was used for multigroup comparisons. Correlation analysis was performed using Pearson correlation analysis. The correlation between gene expression and clinicopathological features was assessed using the chi-square test. Statistical significance was defined as *p* < 0.05.

## Supplementary information

Supplementary Figure

Supplementary Tables

Original western blots

## Data Availability

All data generated or analyzed during this study are included either in this article or GEO (Series GSE301253). Data will be made available on request.
