# Supplementary material for: HOXC9 accelerates esophageal squamous cell carcinoma progression via OTUD1-FABP5-mediated lipid metabolic reprogramming
Source: Cell Death Dis. 2026 Jun 30;17(1):809. doi: 10.1038/s41419-026-09013-4 (PMC13586250; doi:10.1038/s41419-026-09013-4)
Supplement: Supplementary file 1 [file 41419_2026_9013_MOESM1_ESM.pdf]

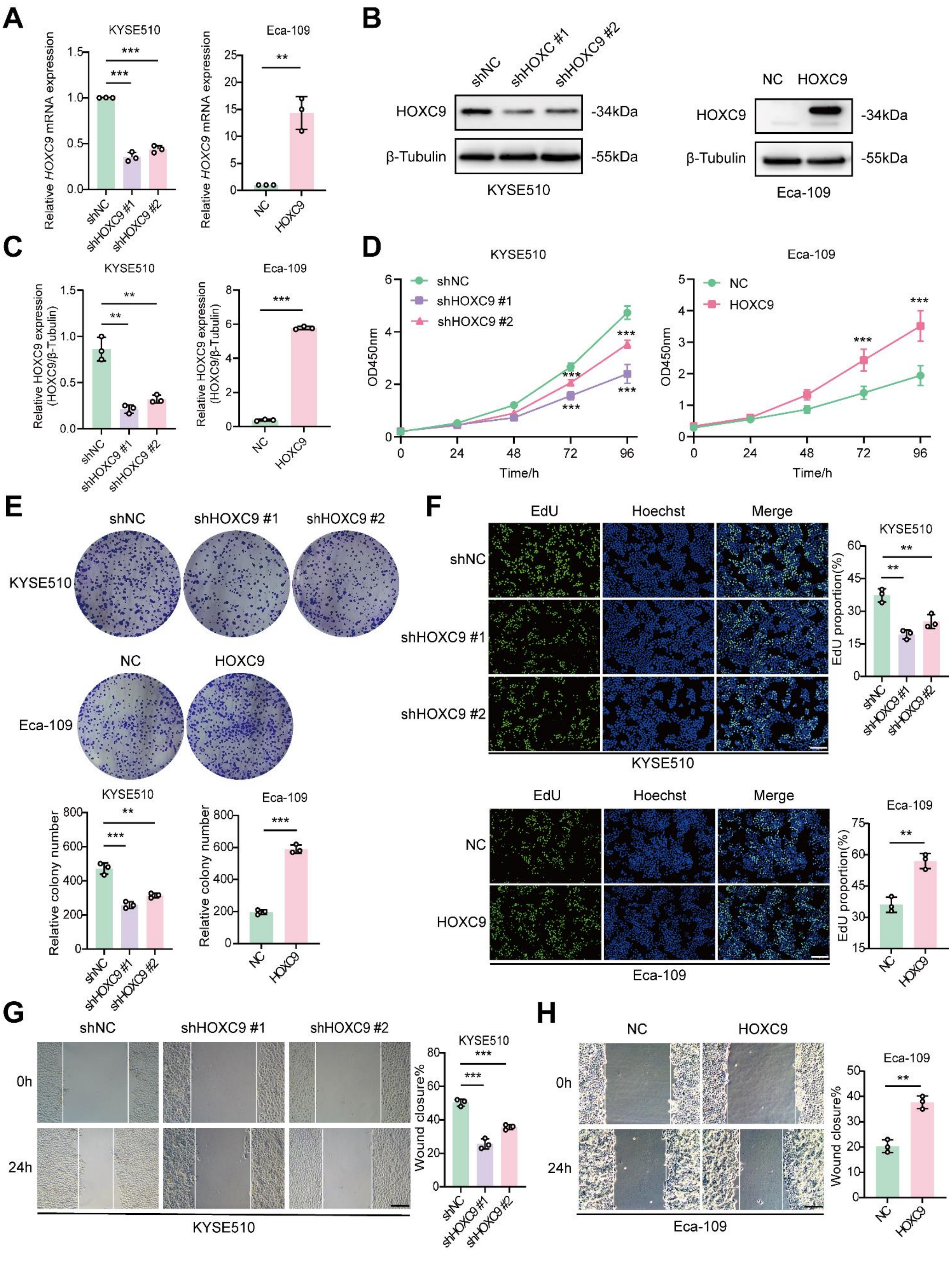

Supplementary Figure 1. HOXC9 promotes the proliferation and migration of KYSE510 and

Eca-109 cells. KYSE510 cells were transduced with control lentivirus (shNC) and HOXC9 shRNA lentivirus (shHOXC9#1, shHOXC9#2). Eca-109 cells were transduced with empty lentivirus (NC) and HOXC9-overexpressing lentivirus (HOXC9). **A-C** The expression of HOXC9 in KYSE510 cells (shNC, shHOXC9#1, shHOXC9#2) and Eca-109 cells (NC, HOXC9) was detected by RT-qPCR (A) and western blot (B-C). **D-F** The proliferation of KYSE510 cells (shNC, shHOXC9#1, shHOXC9#2) and Eca-109 cells (NC, HOXC9) was detected by CCK-8 assays (D), colony-formation assays (E) and EdU assays (F). Scale bar, 200 μm (EdU assays). **G-H** The migration and invasion of KYSE510 cells (shNC, shHOXC9#1, shHOXC9#2) and Eca-109 cells (NC, HOXC9) were detected by wound healing assays. Scale bar, 200 μm. All experiments were repeated three times. Data are shown as the mean ± SD and determined by one-way ANOVA in KYSE510 cells for panels A, C-G and by unpaired t-test in Eca-109 cells for panels A, C-F, and H. $^{**}p < 0.01$; $^{***}p < 0.001$.

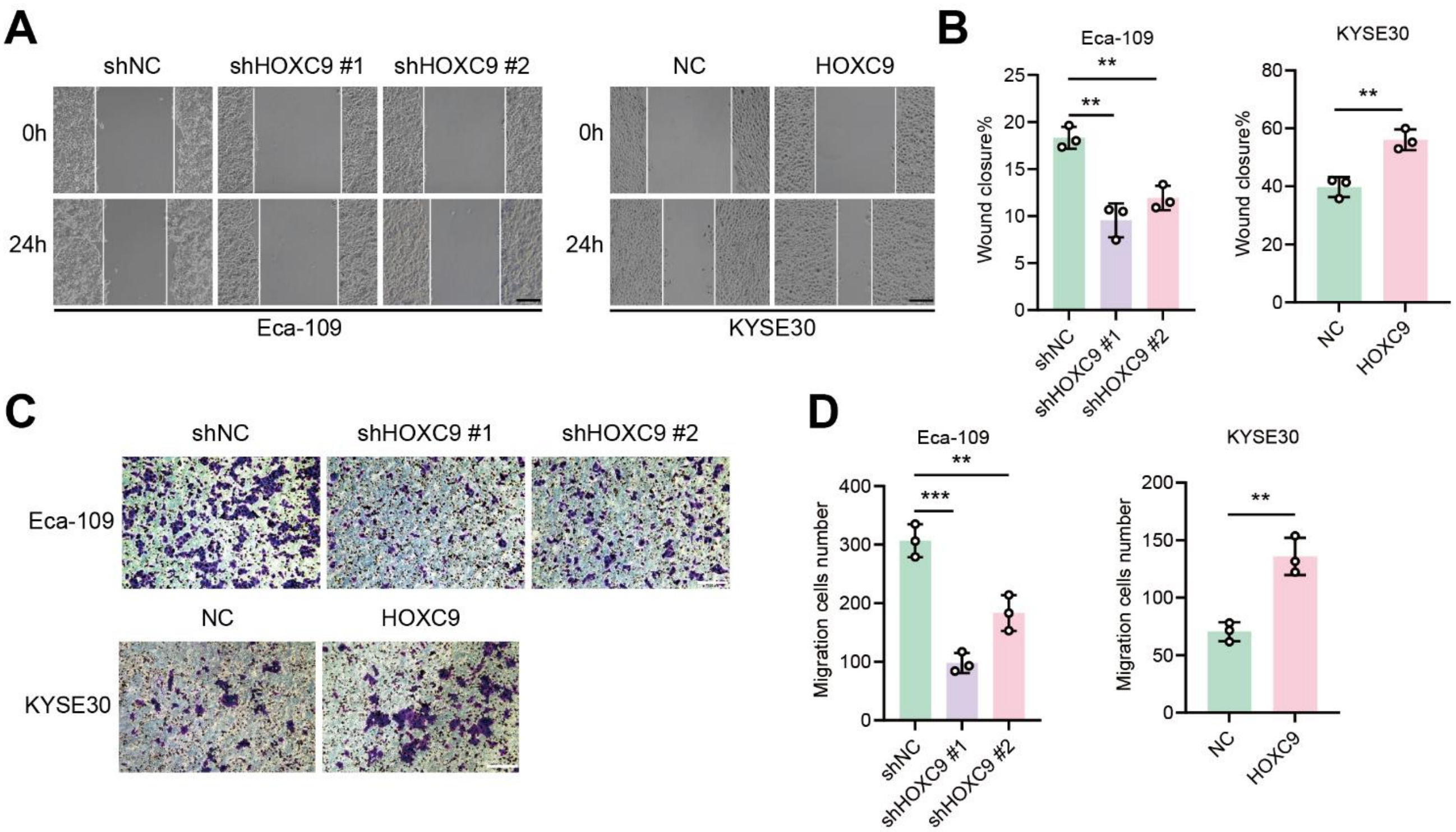

Supplementary Figure 2. Knockdown of HOXC9 inhibits the migration of Eca-109 and KYSE30 cells. Eca-109 cells were transduced with control lentivirus (shNC) and HOXC9 shRNA lentivirus (shHOXC9#1, shHOXC9#2). KYSE30 cells were transduced with empty lentivirus (NC) and HOXC9-overexpressing lentivirus (HOXC9). **A-D** The migration of Eca-109 and KYSE30 cells was detected by wound healing assays (A-B) and Transwell assays (C-D). Scale bar, 200 μm (wound healing assays); Scale bar, 100 μm (Transwell assays). All experiments were repeated three times. Data are shown as the mean ± SD and statistical significance was determined by one-way ANOVA in Eca-109 cells and unpaired t-test in KYSE30 cells for panels B and D. ***p* < 0.01; ****p* < 0.001.

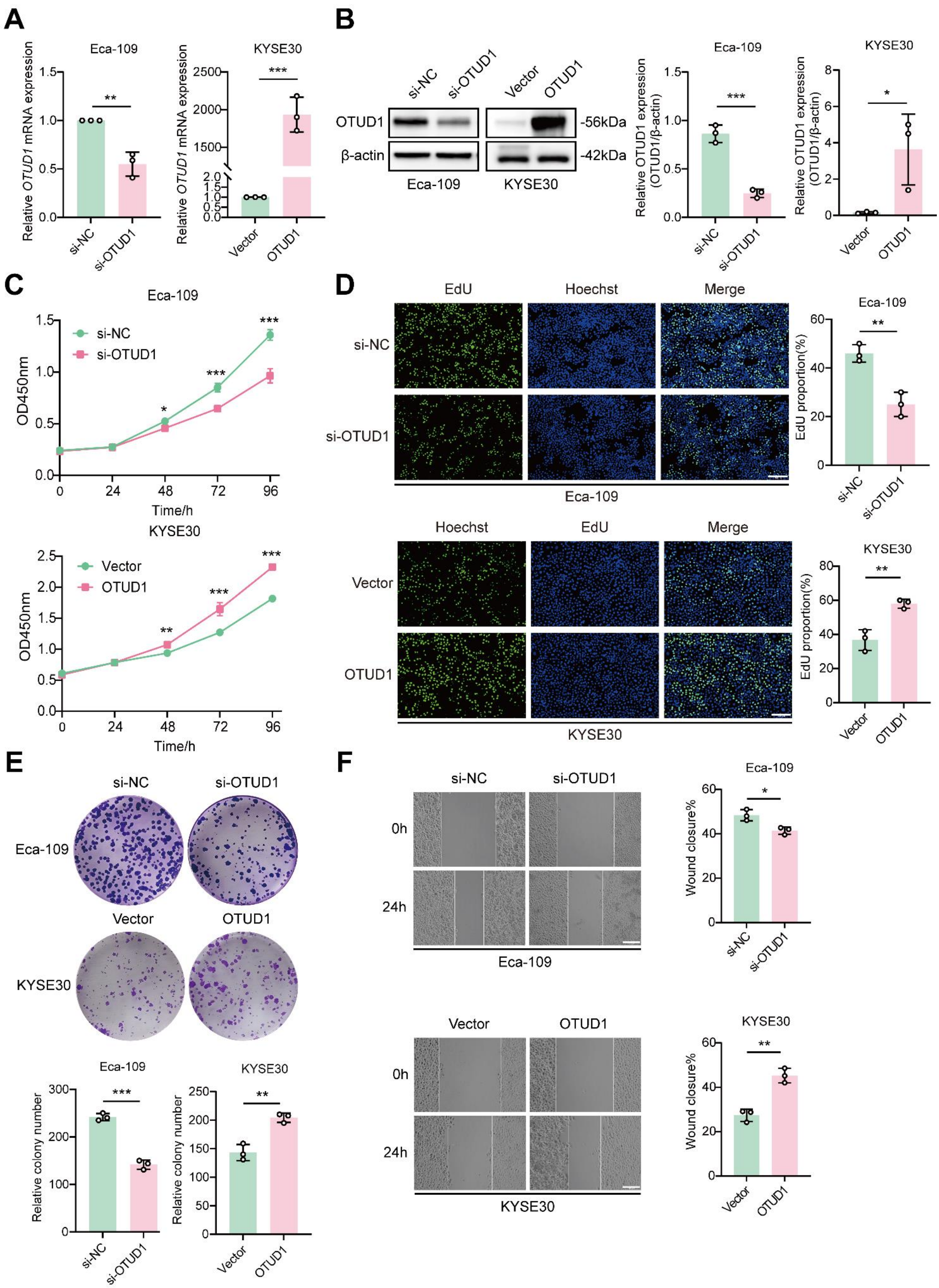

Supplementary Figure 3. OTUD1 promotes the proliferation and migration of ESCC cells. Eca-

109 cells were transfected with control siRNA (si-NC) and OTUD1 siRNA (si-OTUD1). KYSE30 cells were transfected with empty plasmid (Vector) and OTUD1-overexpressing plasmid (OTUD1). **A-B** The expression of OTUD1 in Eca-109 cells (si-NC, si-OTUD1) and KYSE30 cells (Vector, OTUD1) was tested by RT-qPCR (A) and western blot (B). **C-E** The proliferation of Eca-109 cells (si-NC, si-OTUD1) and KYSE30 cells (Vector, OTUD1) was detected by CCK-8 assays (C), EdU assays (D) and colony-formation assays (E). Scale bar, 200 μm (EdU assays). **F** The migration of Eca-109 cells (si-NC, si-OTUD1) and KYSE30 cells (Vector, OTUD1) was detected by wound healing assays. Scale bar, 200 μm. All experiments were repeated three times. Data are shown as the mean ± SD and determined by unpaired t-test. *$p < 0.05$; **$p < 0.01$; ***$p < 0.001$.

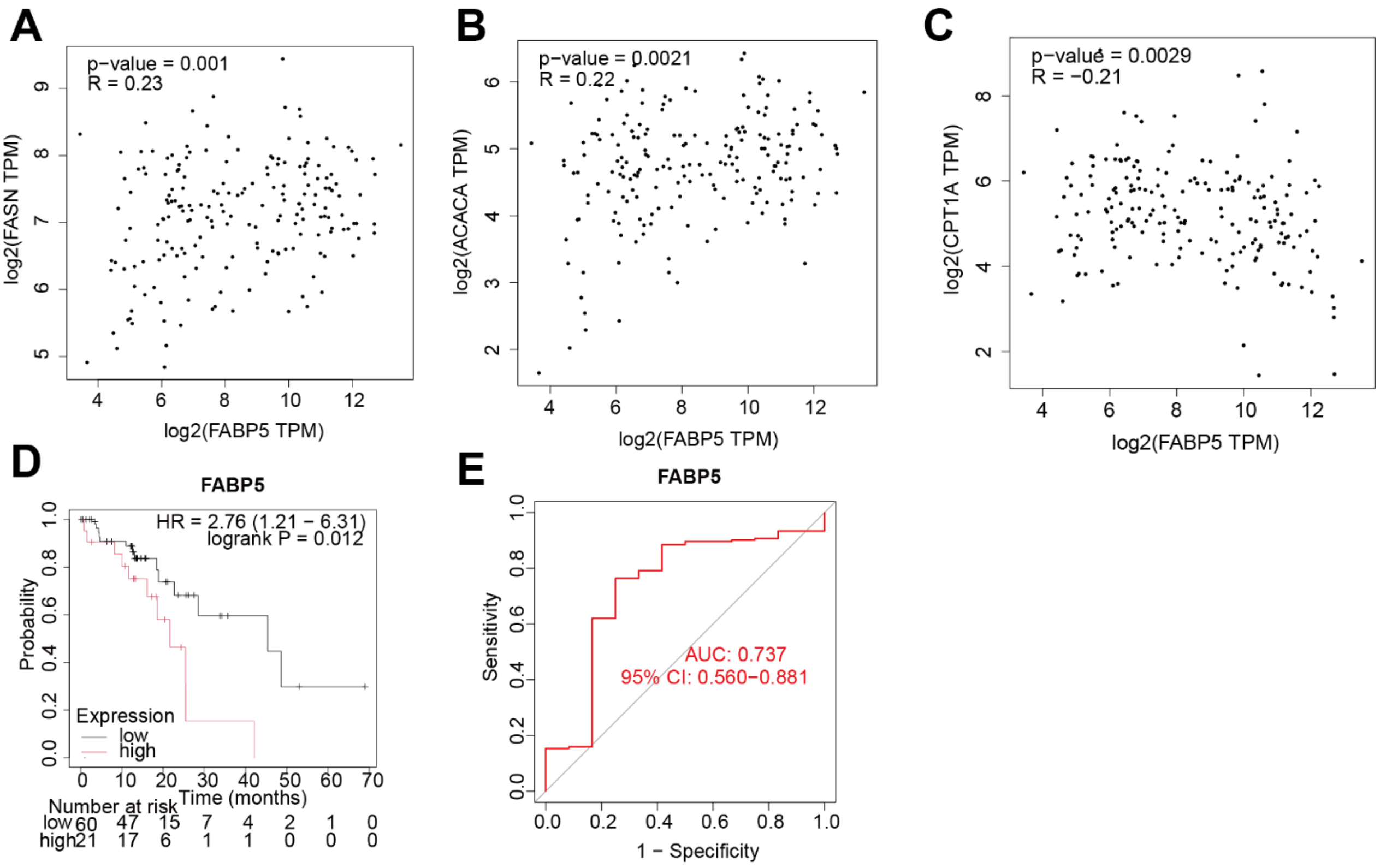

Supplementary Figure 4. FABP5 is positively associated with FASN, ACACA, CPT1A and the poor prognosis of ESCC patients. A-C The correlation of FABP5 expression with FASN (A), ACACA (B) and CPTIA (C) expression in ESCC patients was analysed based on the GEPIA profiling datasets. **D** Kaplan-Meier plotter analysis of ESCC patients with different FABP5 expression. HR, hazard ratio. **E** ROC curve of FABP5 expression in patients with ESCC obtained from TCGA.

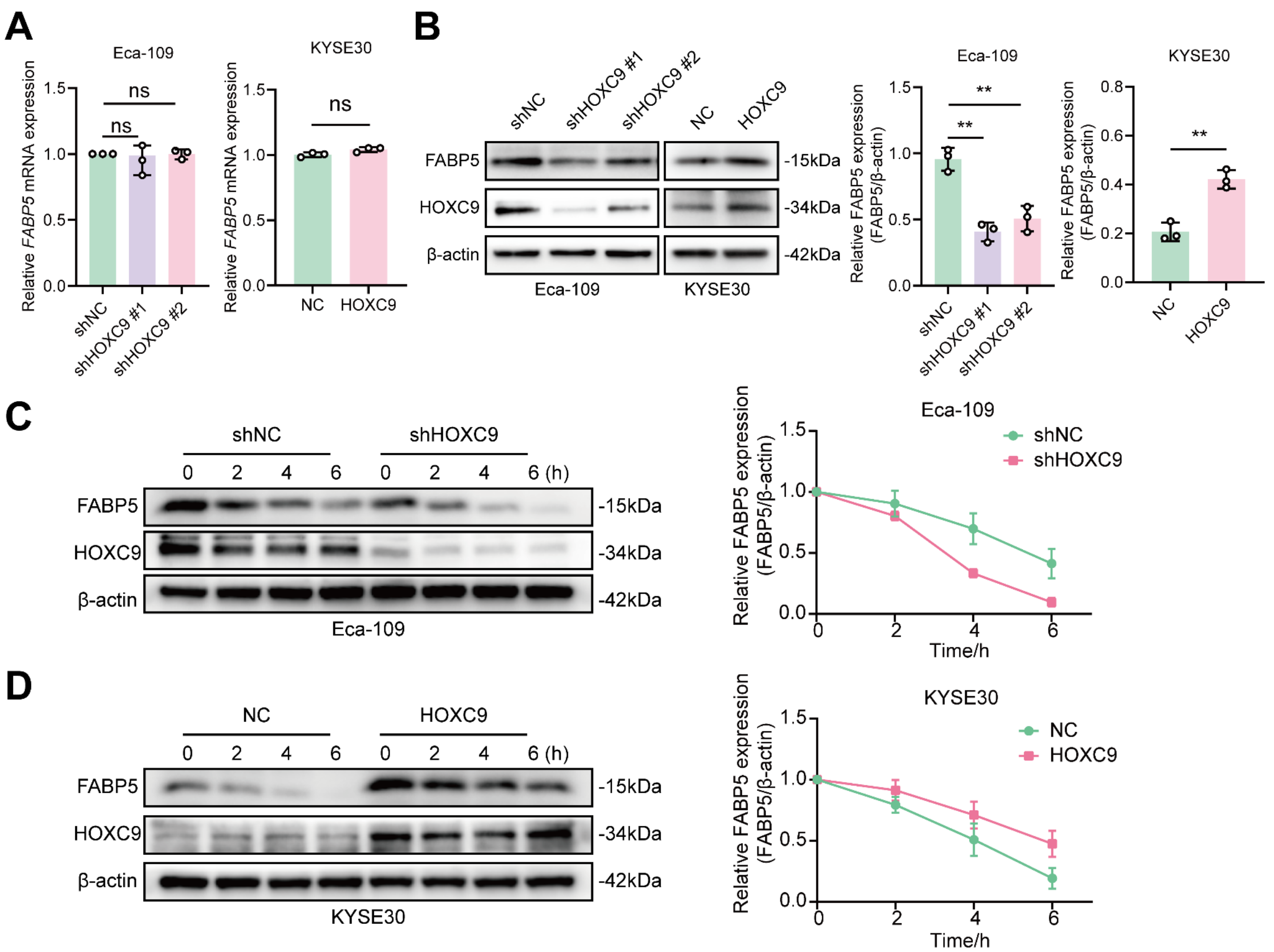

Supplementary Figure 5. HOXC9 promotes FABP5 protein expression by improving FABP5 protein stability. A-B FABP5 expression in Eca-109 (shNC, shHOXC9#1, shHOXC9#2) and KYSE30 (NC, HOXC9) cells was tested by RT-qPCR (A) and western blot (B). **C-D** FABP5 protein expression in Eca-109 (shNC, shHOXC9) and KYSE30 cells (NC, HOXC9) treated with cycloheximide (CHX, 400 µg/ml) for the indicated time was measured by western blot. All experiments were repeated three times. Data are shown as the mean ± SD and statistical significance was determined by one-way ANOVA in Eca-109 cells and unpaired t-test in KYSE30 cells for panels A and B. Ns, no significance; $**p < 0.01$.

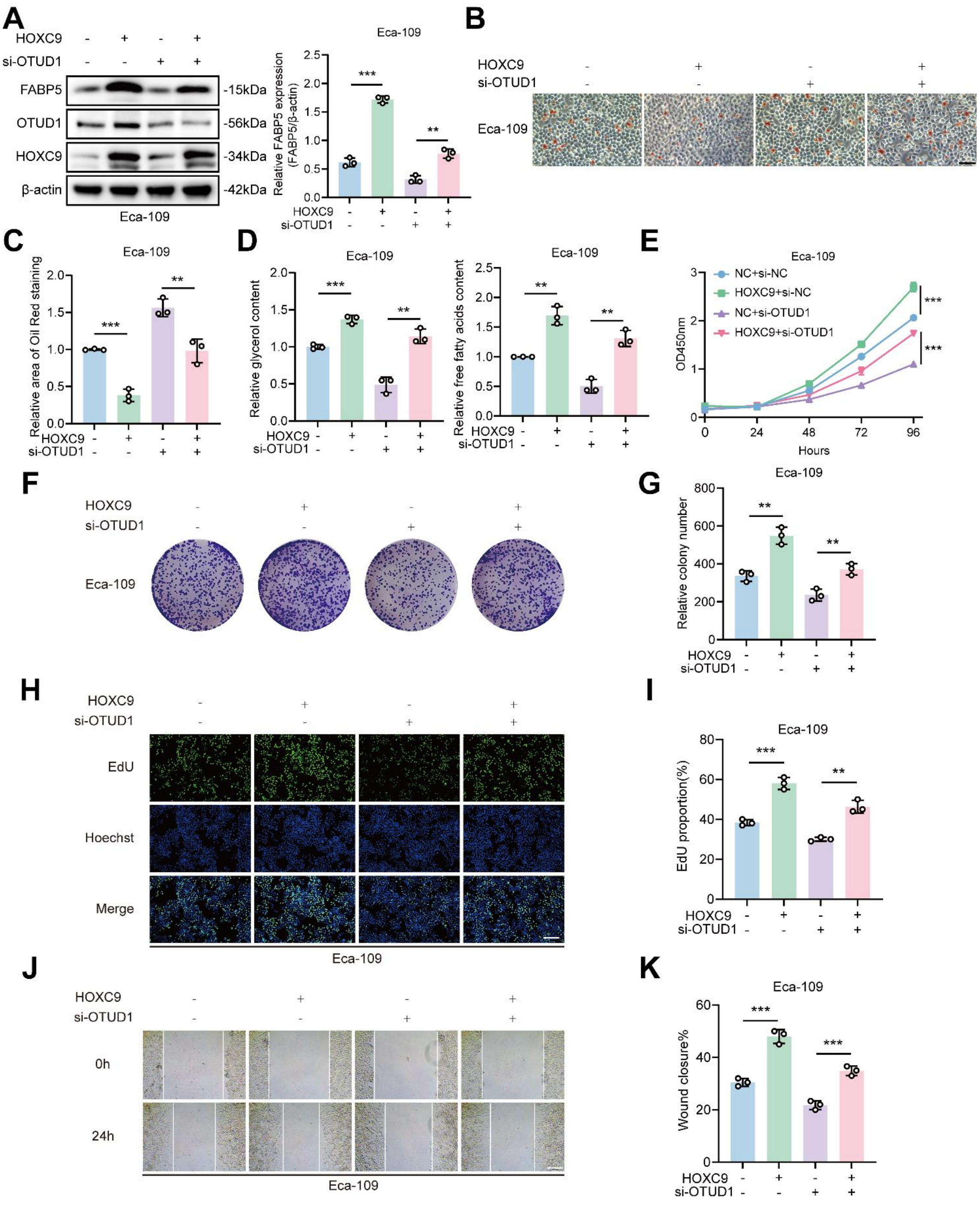

Supplementary Figure 6. Knockdown of OTUD1 inhibits HOXC9-mediated lipid metabolism, proliferation and migration in Eca-109 cells. Eca-109 cells (NC, HOXC9) were transfected with control siRNA (si-NC) and OTUD1 siRNA (si-OTUD1). **A** HOXC9, OTUD1 and FABP5 protein

expression in different groups of Eca-109 cells were detected by western blot. **B-C** Oil Red O staining in different groups of Eca-109 cells was shown. Scale bar, 100 μm. **D** Glycerol and free fatty acid levels in different groups of Eca-109 cells were determined. **E-I** The proliferation in different groups of Eca-109 cells was detected by CCK-8 assays (E), colony-formation assays (F-G) and EdU assays (H-I). Scale bar, 200 μm (EdU assays). **J-K** The migrative capacity in different groups of Eca-109 cells was detected by wound healing assays. Scale bar, 200 μm. All experiments were repeated three times. Data are shown as the mean ± SD and determined by two-way ANOVA. $^{**}p < 0.01$; $^{***}p < 0.001$.

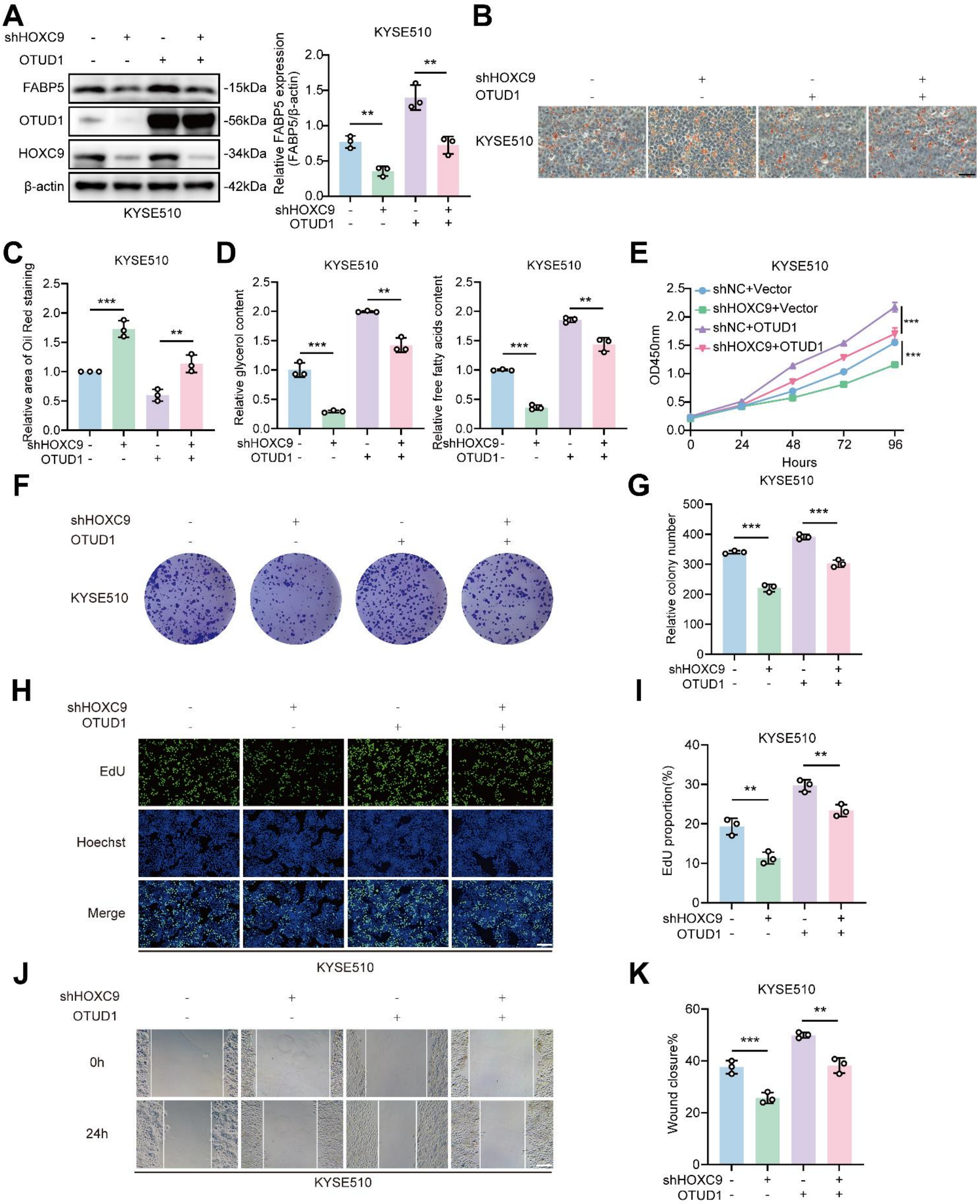

Supplementary Figure 7. Overexpression of OTUD1 rescues the reduced lipolysis level, proliferative and migrative ability in HOXC9-silencing KYSE510 cells. KYSE510 cells (shNC, shHOXC9) were transfected with empty plasmid (Vector) and OTUD1-overexpressing plasmid

(OTUD1). **A** HOXC9, OTUD1 and FABP5 protein expression in different groups of KYSE510 cells were detected by western blot. **B-C** Oil Red O staining in different groups of KYSE510 cells was shown. Scale bar, 100 μm. **D** Glycerol and free fatty acid levels in different groups of KYSE510 cells were measured. **E-I** The proliferation of KYSE510 cells in different groups was detected by CCK-8 assays (E), colony-formation assays (F-G) and EdU assays (H-I). Scale bar, 200 μm (EdU assays). **J-K** The migrative capacity of KYSE510 cells in different groups was detected by wound healing assays. Scale bar, 200 μm. All experiments were repeated three times. Data are shown as the mean ± SD and determined by two-way ANOVA. ***p* < 0.01; ****p* < 0.001.

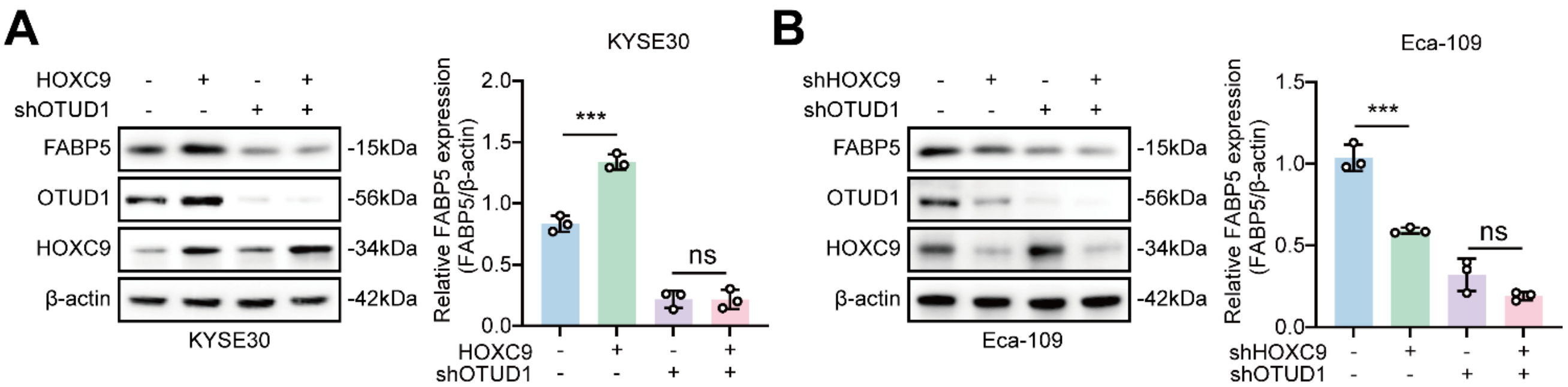

Supplementary Figure 8. Profound inhibition of OTUD1 blocks the regulatory effect of HOXC9 on FABP5. A KYSE30 (NC, HOXC9) were transfected with control lentivirus or OTUD1 shRNA lentivirus (shOTUD1). HOXC9, OTUD1 and FABP5 protein expression in different groups of KYSE30 cells was detected by western blot. **B** Eca-109 (shNC, shHOXC9) cells were transfected with control lentivirus or OTUD1 shRNA lentivirus (shOTUD1). HOXC9, OTUD1 and FABP5 protein expression in different groups of Eca-109 cells was detected by western blot. All experiments were repeated three times. Data are shown as the mean ± SD and determined by two-way ANOVA. Ns, no significance; ***$p < 0.001$.

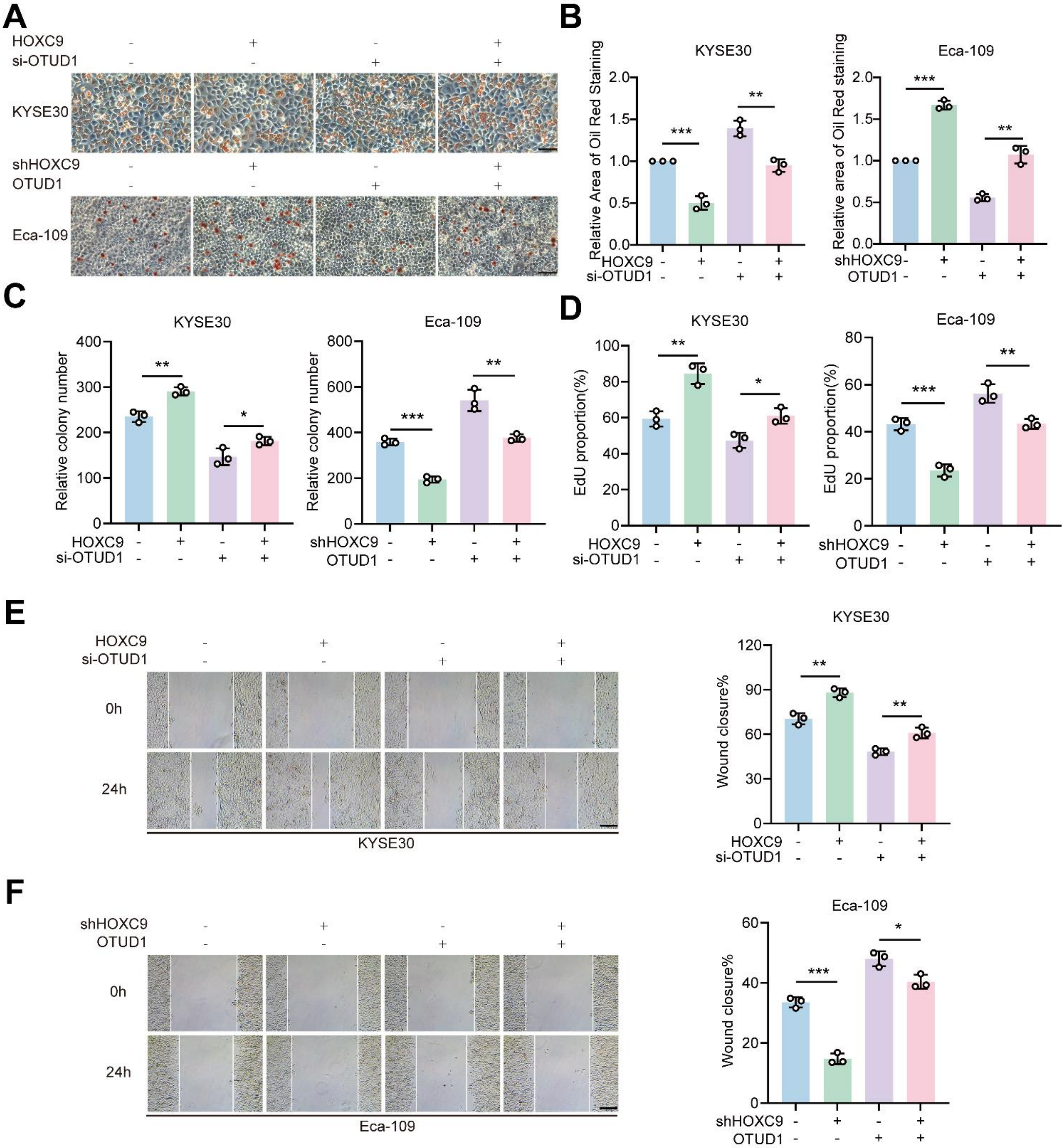

Supplementary Figure 9. HOXC9 promotes lipolysis, proliferation and migration of ESCC cells via OTUD1. KYSE30 (NC, HOXC9) cells were transfected with control siRNA (si-NC) and OTUD1 siRNA (si-OTUD1). Eca-109 (shNC, shHOXC9) cells were transfected with empty plasmid (Vector) and OTUD1-overexpressing plasmid (OTUD1). **A-B** Oil Red O staining of different groups of KYSE30 and Eca-109 cells were shown. Scale bar, 100 μm. **C-D** The proliferation of KYSE30 and

Eca-109 cells in different groups was detected by colony formation assays (C) and EdU assays (D). **E-F** The migration of KYSE30 and Eca-109 cells in different groups was detected by wound healing assays. Scale bar, 200 μm. All experiments were repeated three times. Data are shown as the mean ± SD and determined by two-way ANOVA. $*p < 0.05$; $**p < 0.01$; $***p < 0.001$.

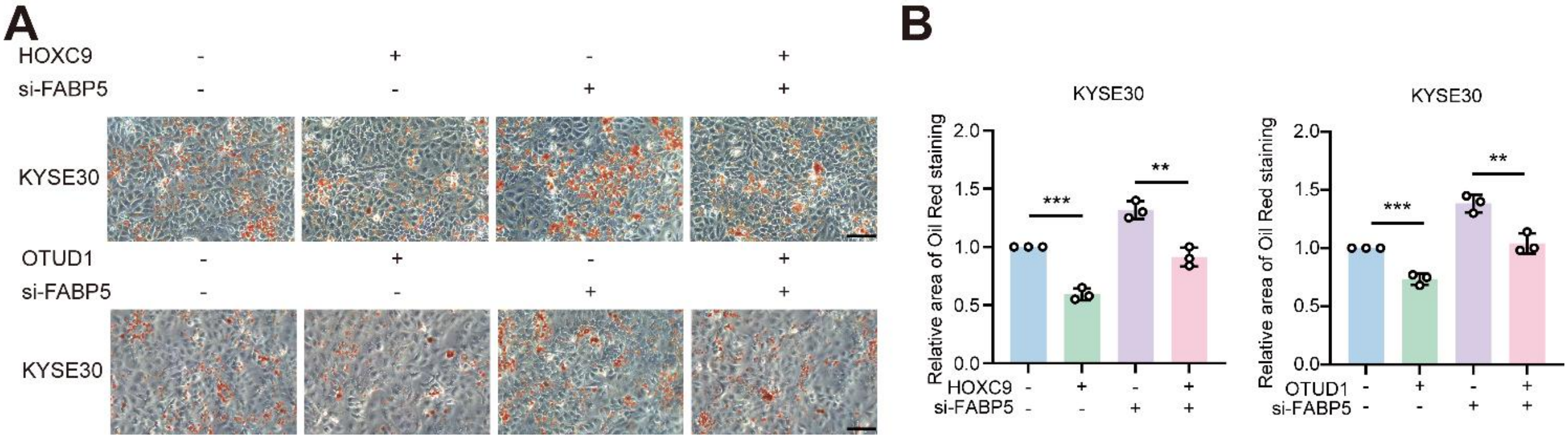

Supplementary Figure 10. FABP5 knockdown restrains the lipolysis-promoting effects of HOXC9 and OTUD1 in ESCC cells. A-B KYSE30 (NC and HOXC9, Vector and OTUD1) cells were transfected with control siRNA (si-NC) or FABP5 siRNA (si-FABP5). Oil Red O staining in different groups of KYSE30 cells was shown. Scale bar, 100 μm. All experiments were repeated three times. Data are shown as the mean ± SD and determined by two-way ANOVA. $^{**}p < 0.01$; $^{***}p < 0.001$.
