# Supplementary material for: HOXC9 accelerates esophageal squamous cell carcinoma progression via OTUD1-FABP5-mediated lipid metabolic reprogramming
Source: Cell Death Dis. 2026 Jun 30;17(1):809. doi: 10.1038/s41419-026-09013-4 (PMC13586250; doi:10.1038/s41419-026-09013-4)

Fig. 1E HOXC9

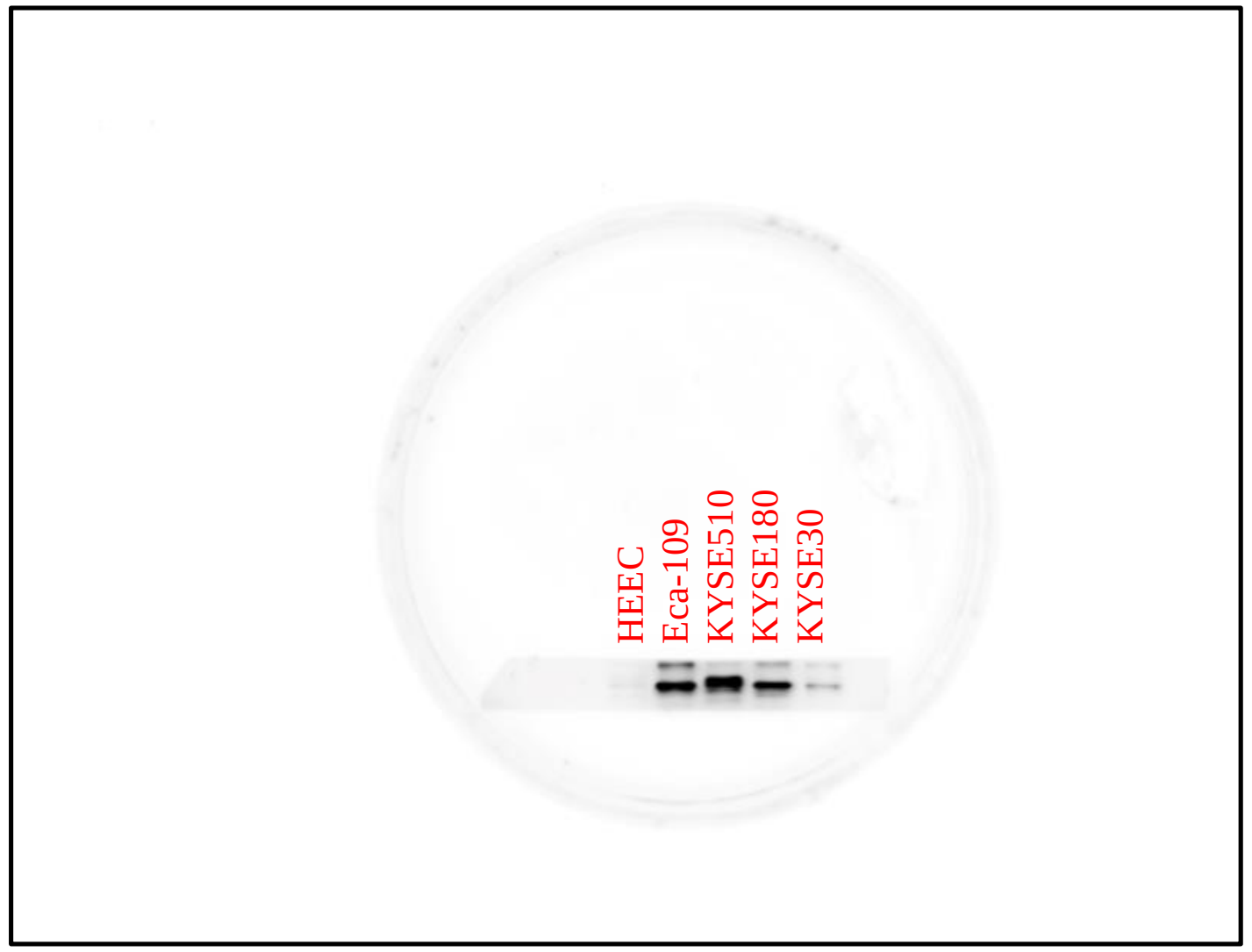

Fig. 1E β-Tubulin

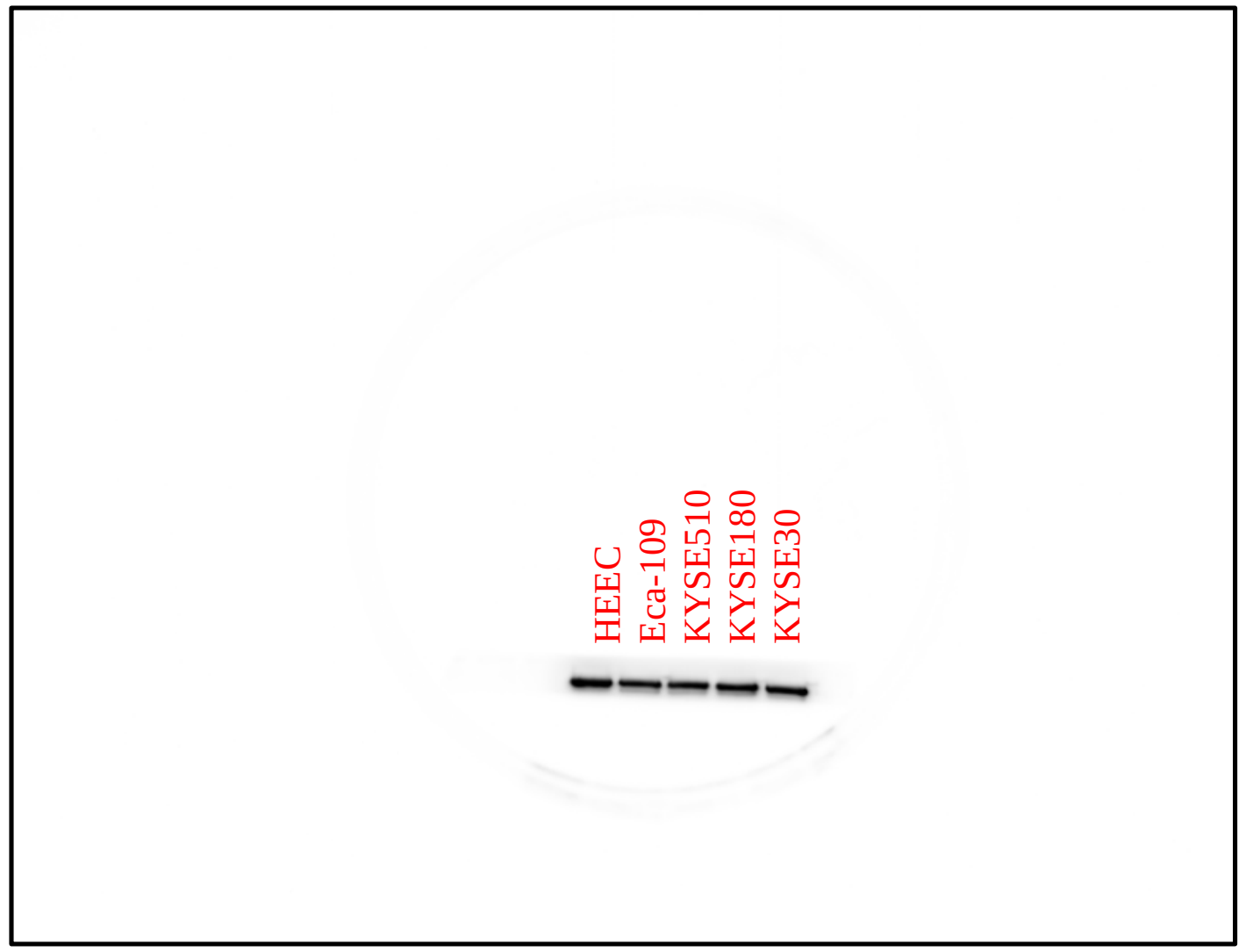

Fig. 2B β-Tubulin

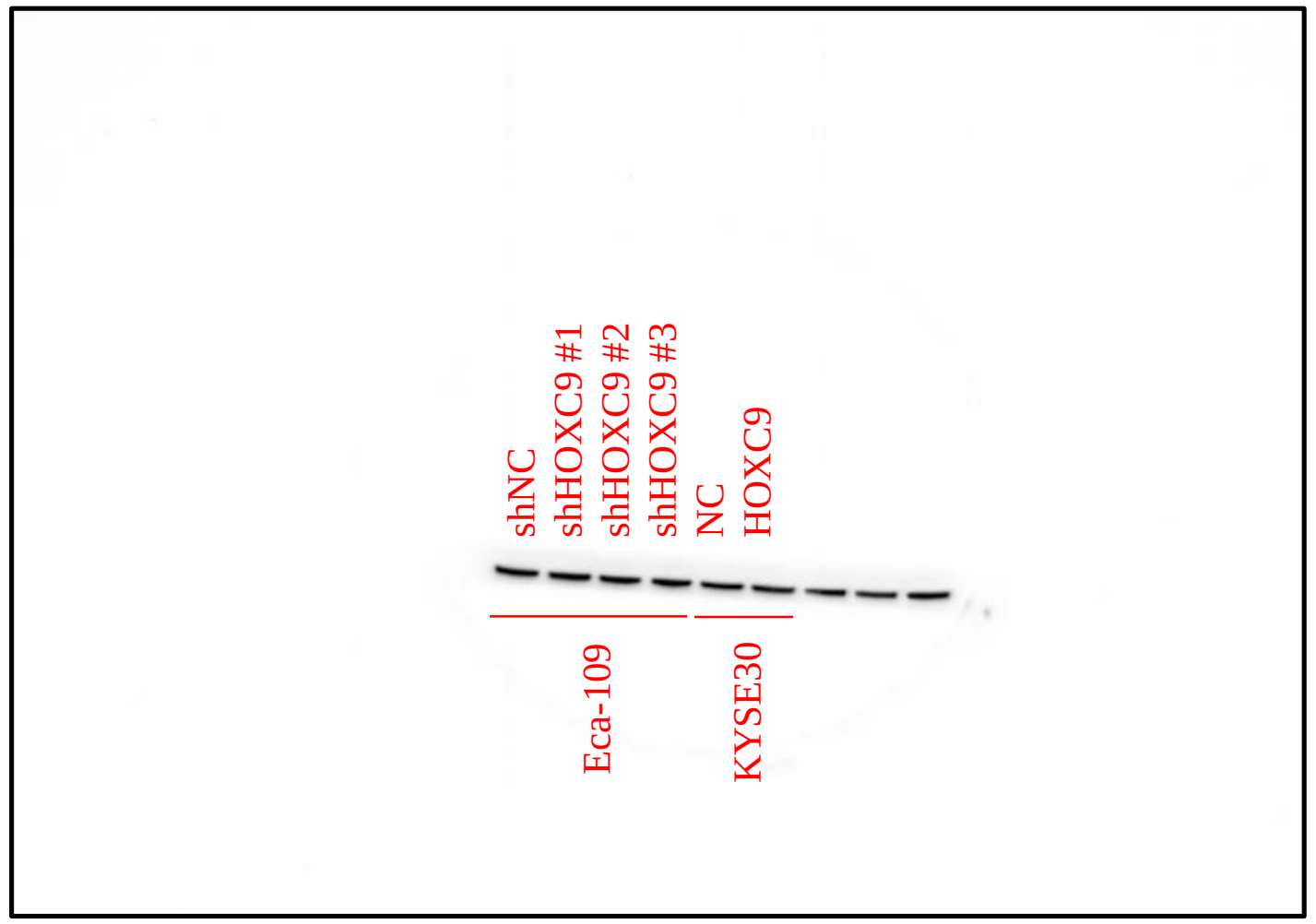

Fig. 2B HOXC9-low exposure

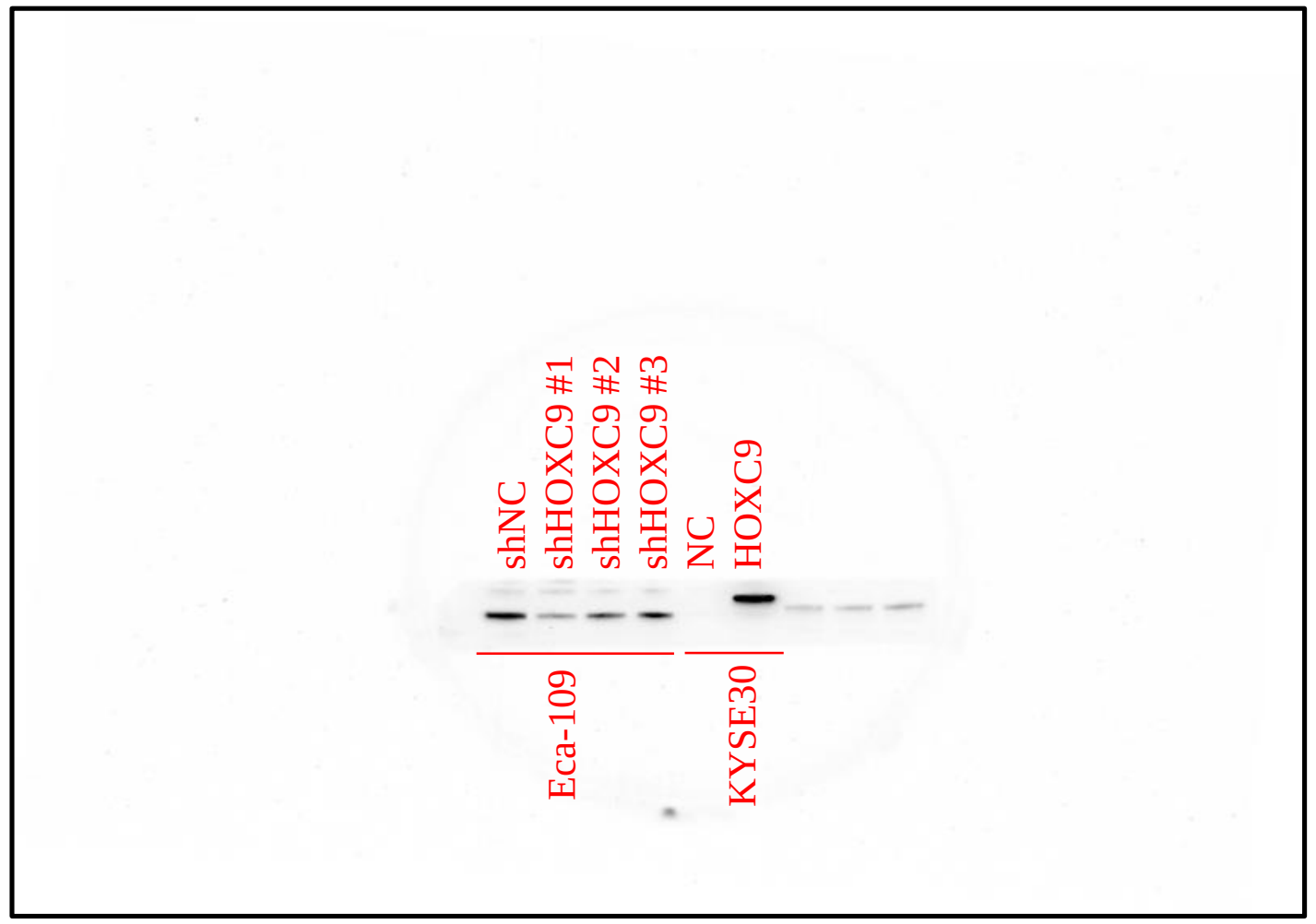

Fig. 2B HOXC9-high exposure

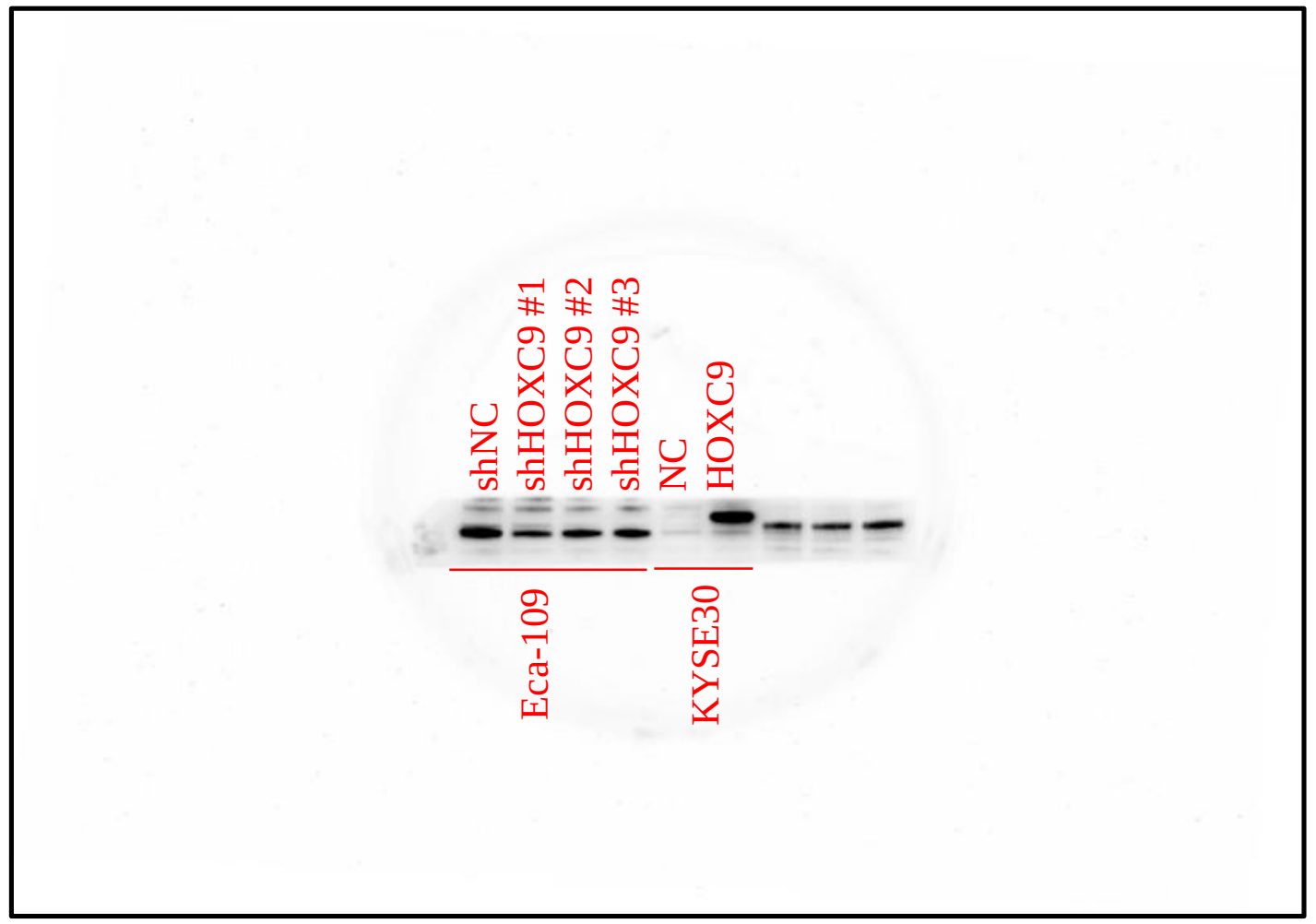

Fig. 2G β-actin

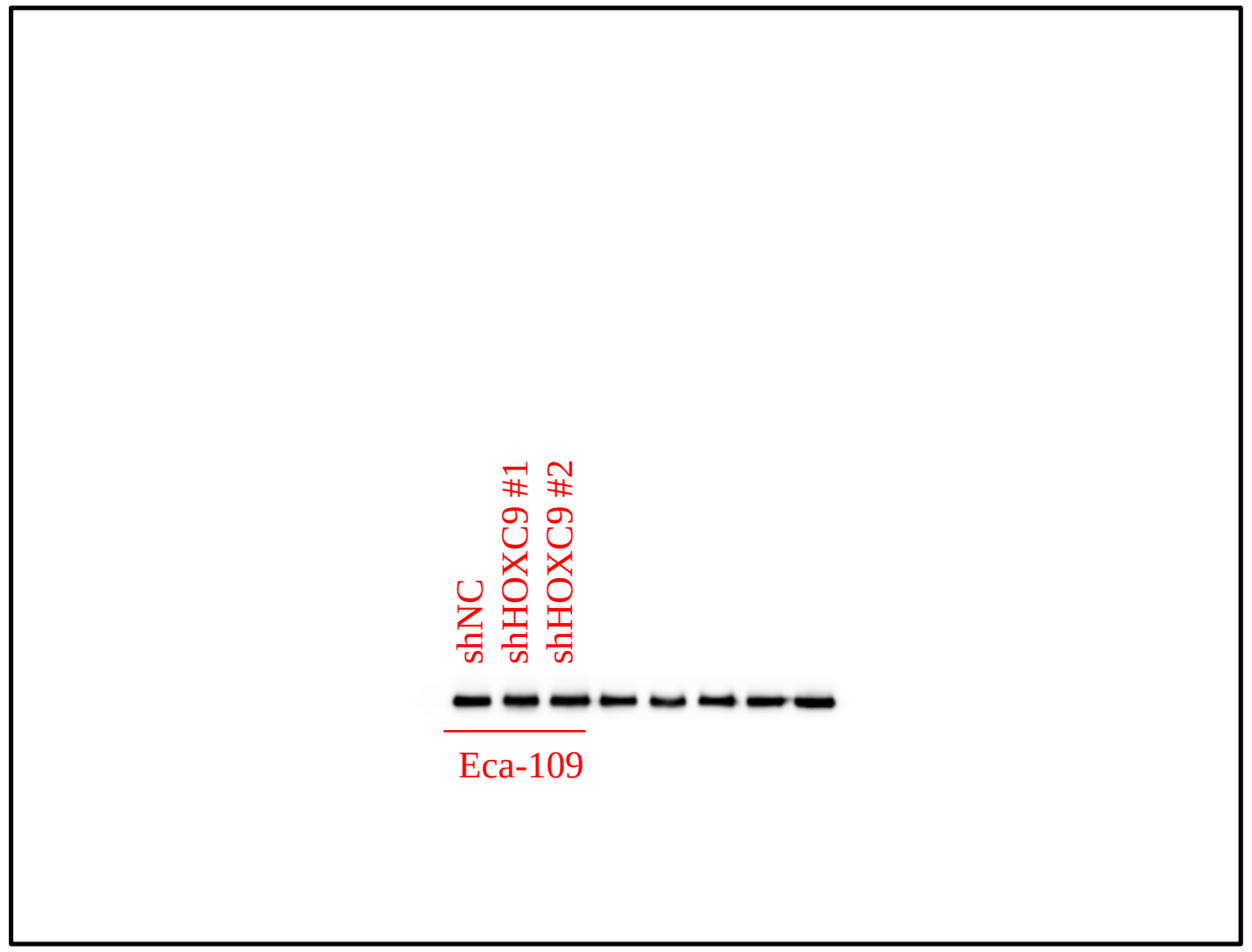

Fig. 2G Bax

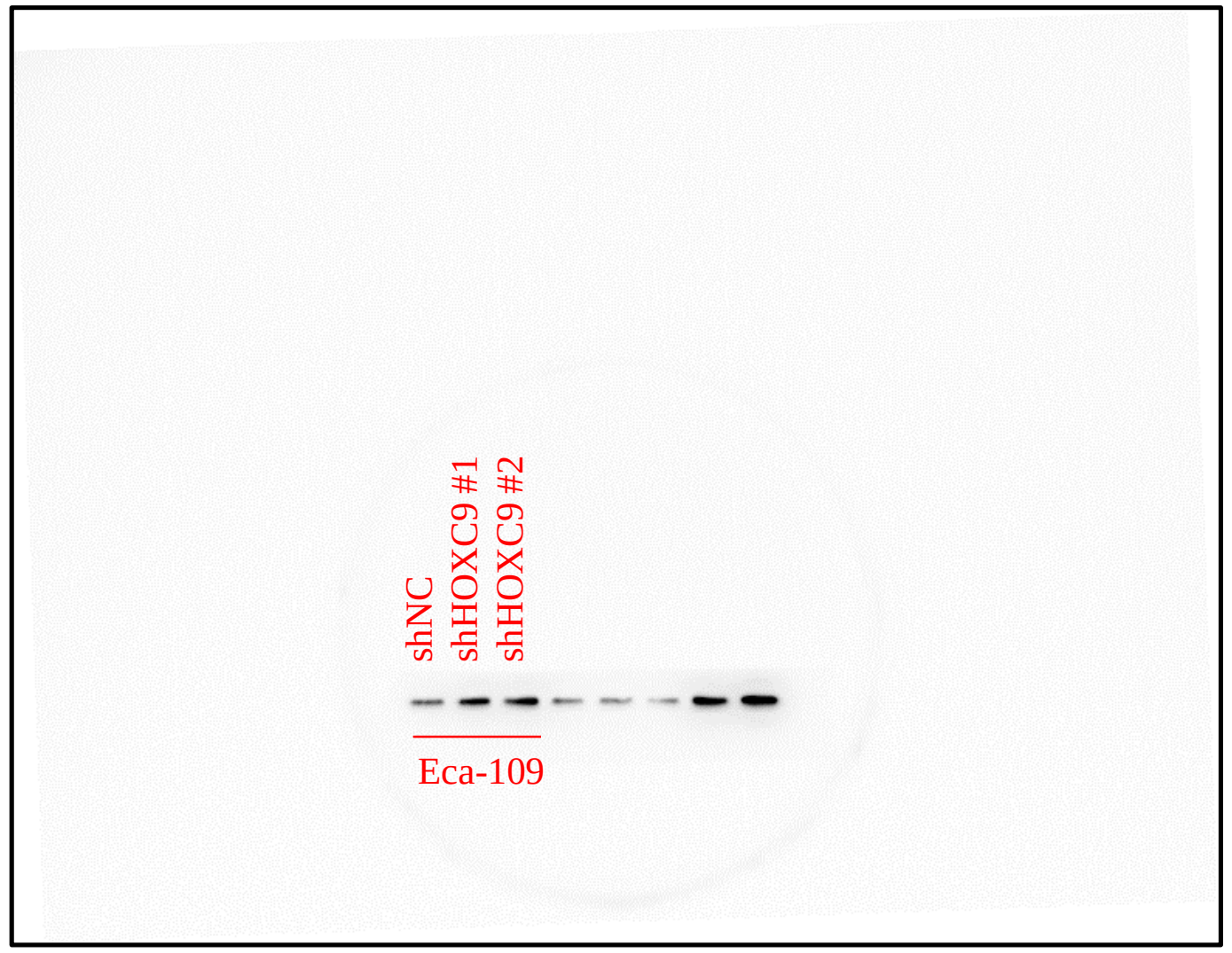

Fig. 2G β-actin

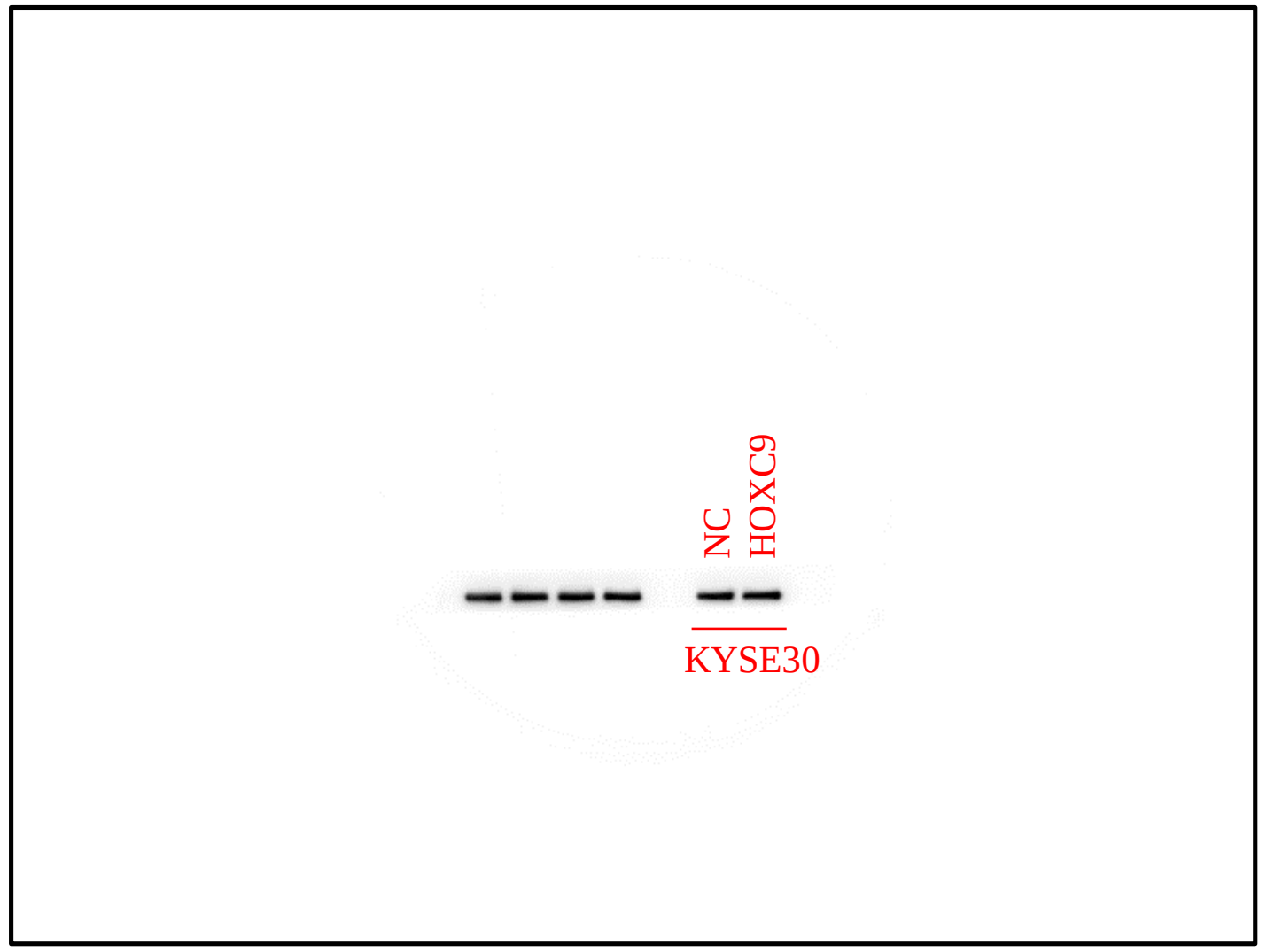

Fig. 2G Bax

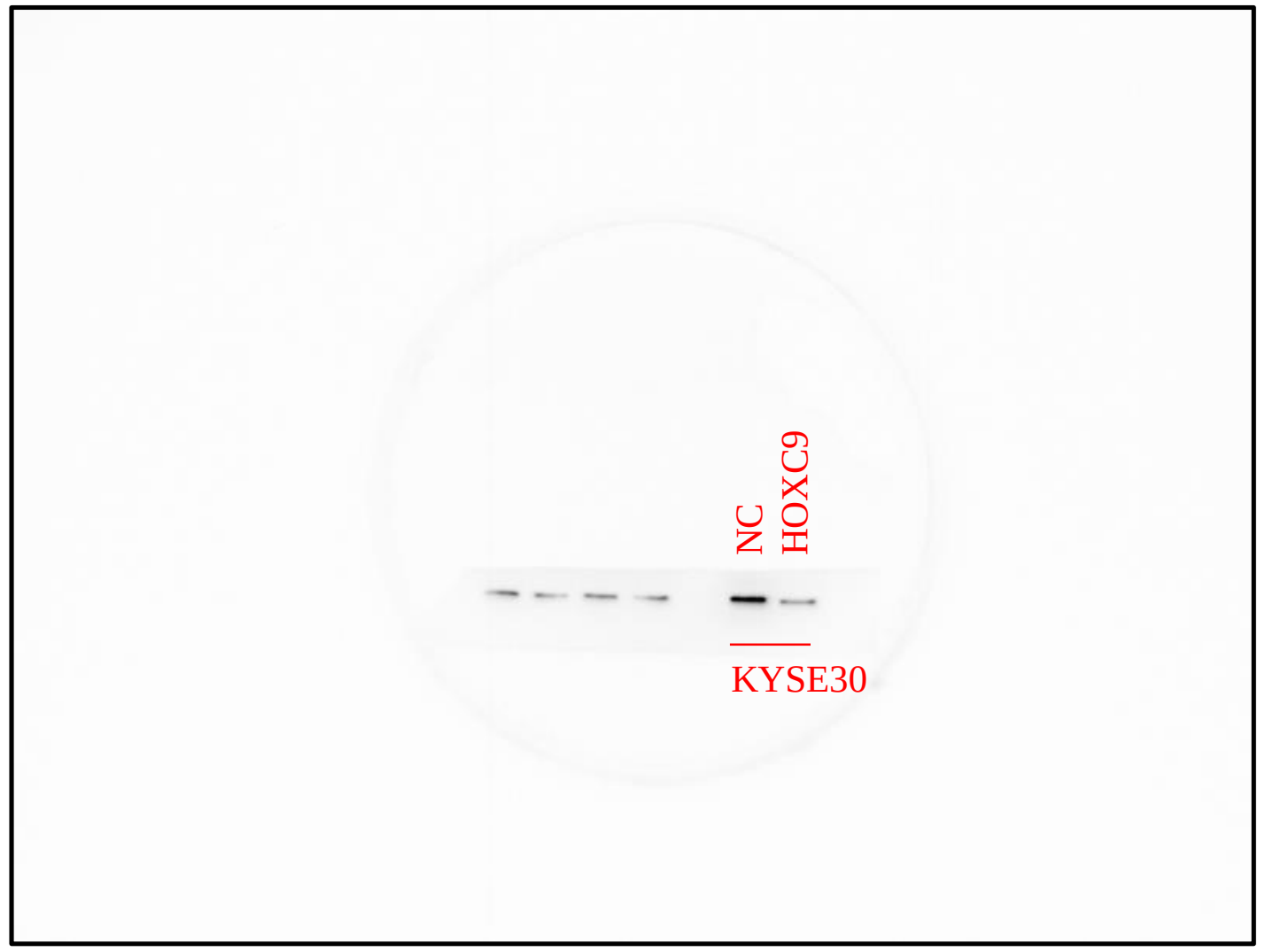

Fig. 2G Cyclin B1

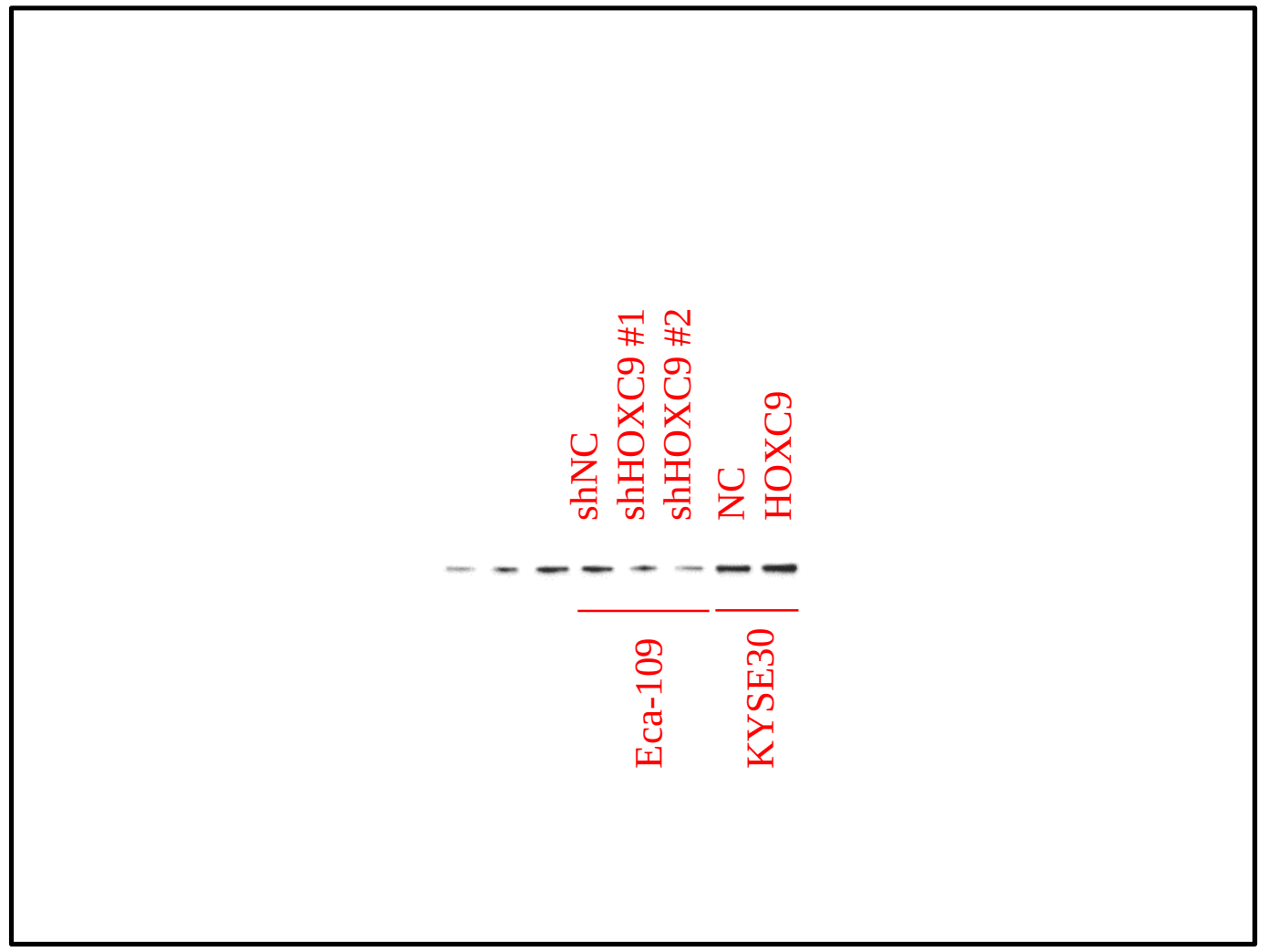

Fig. 4F β-actin

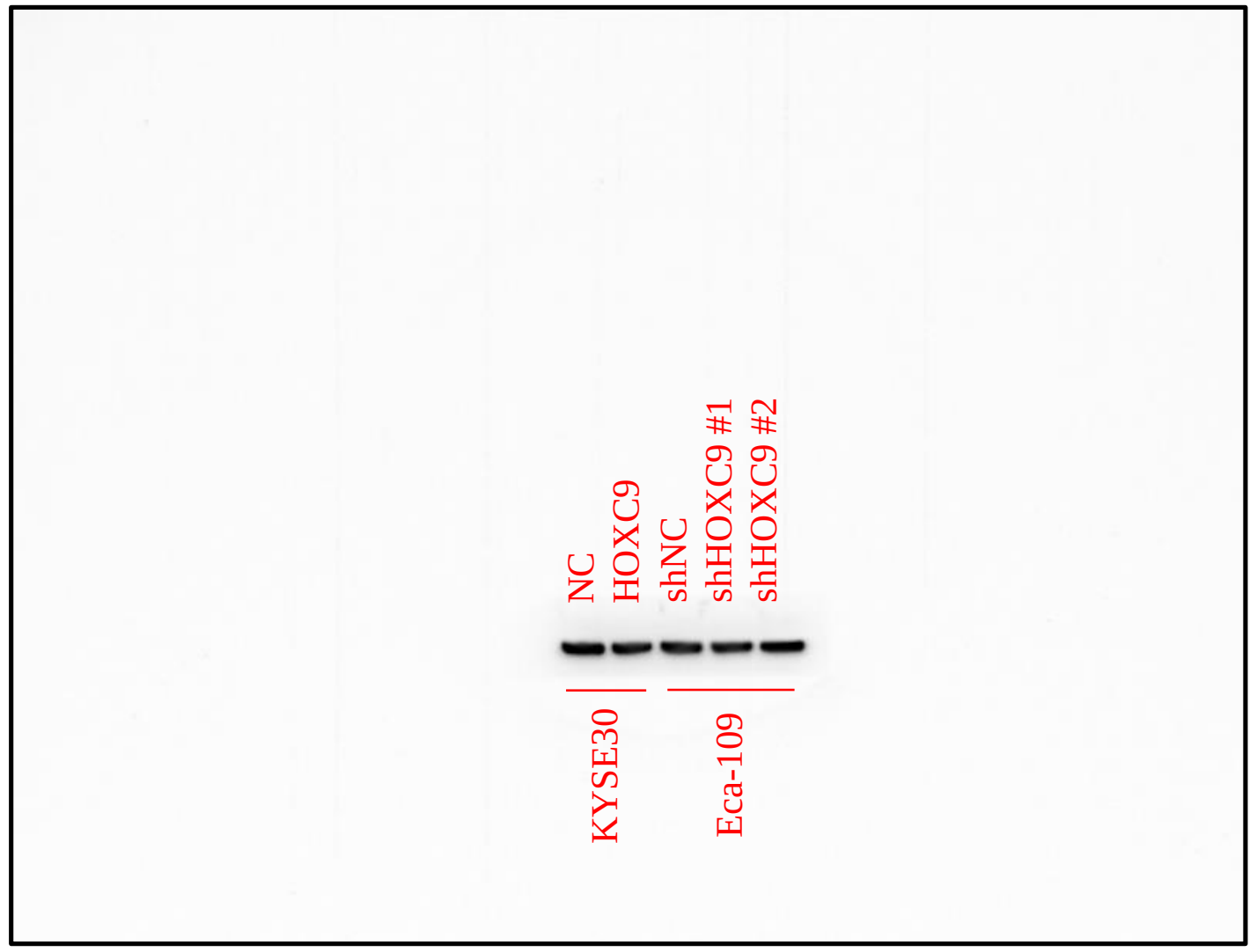

Fig. 4F HOXC9

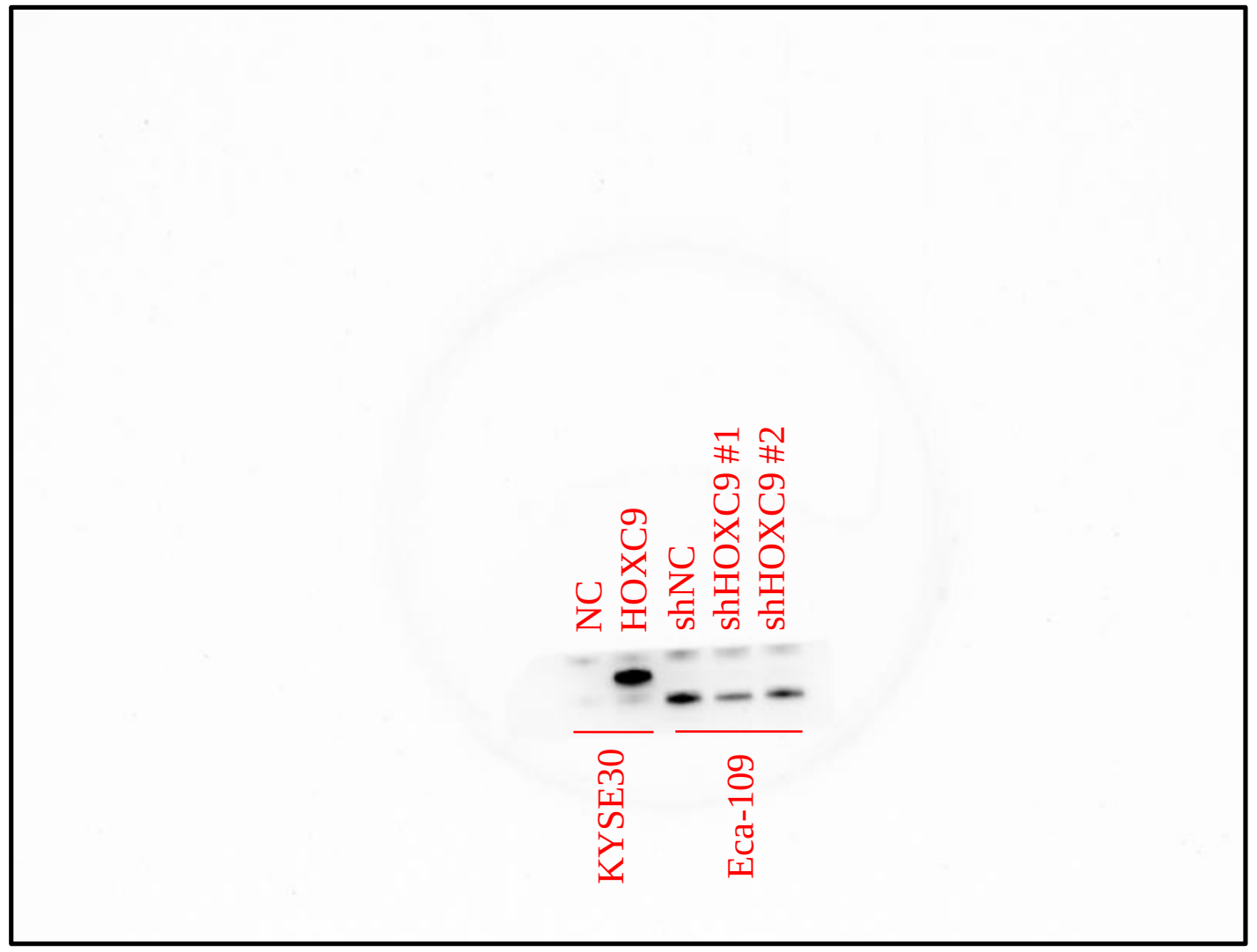

Fig. 4F OTUD1-low exposure

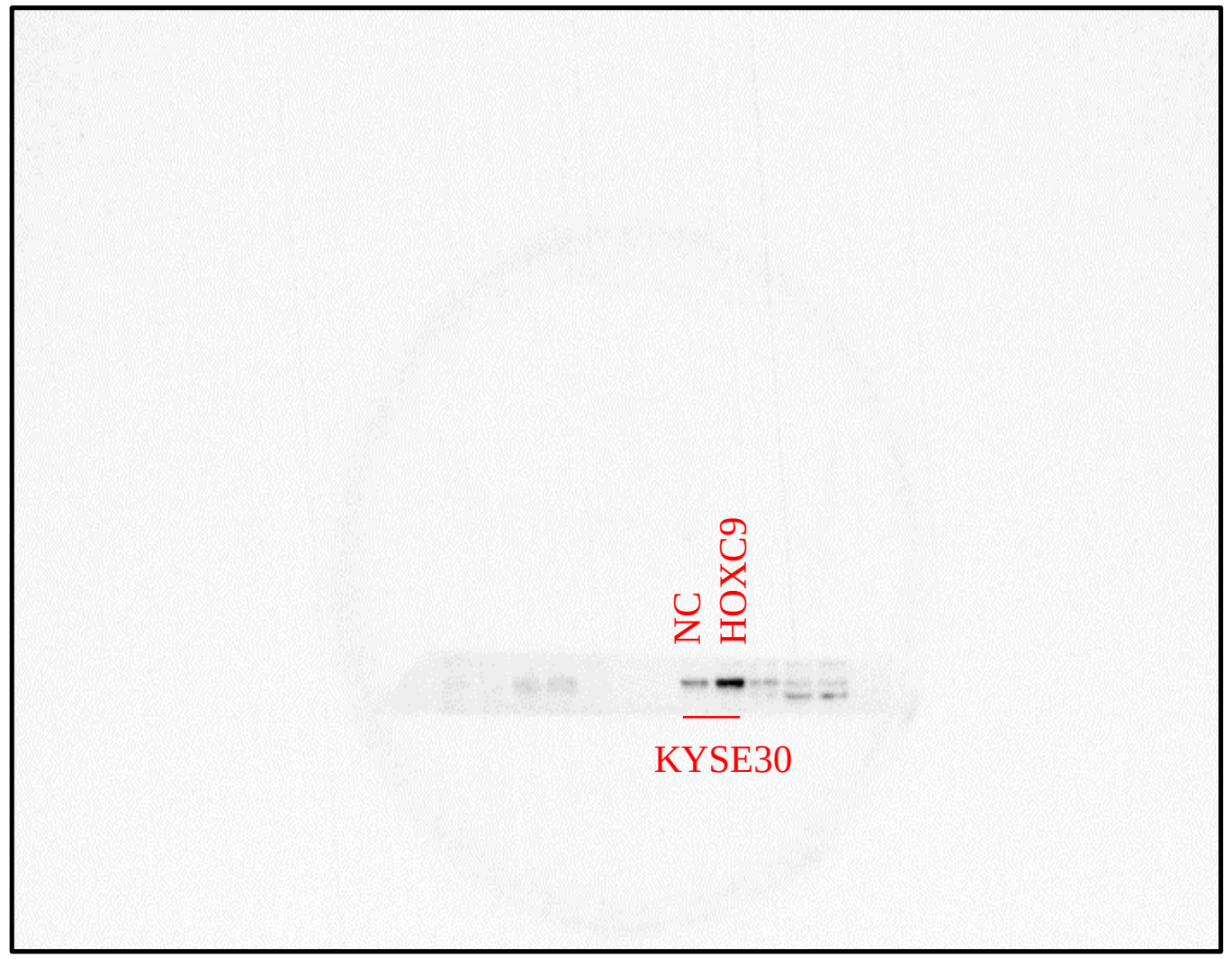

Fig. 4F OTUD1-high exposure

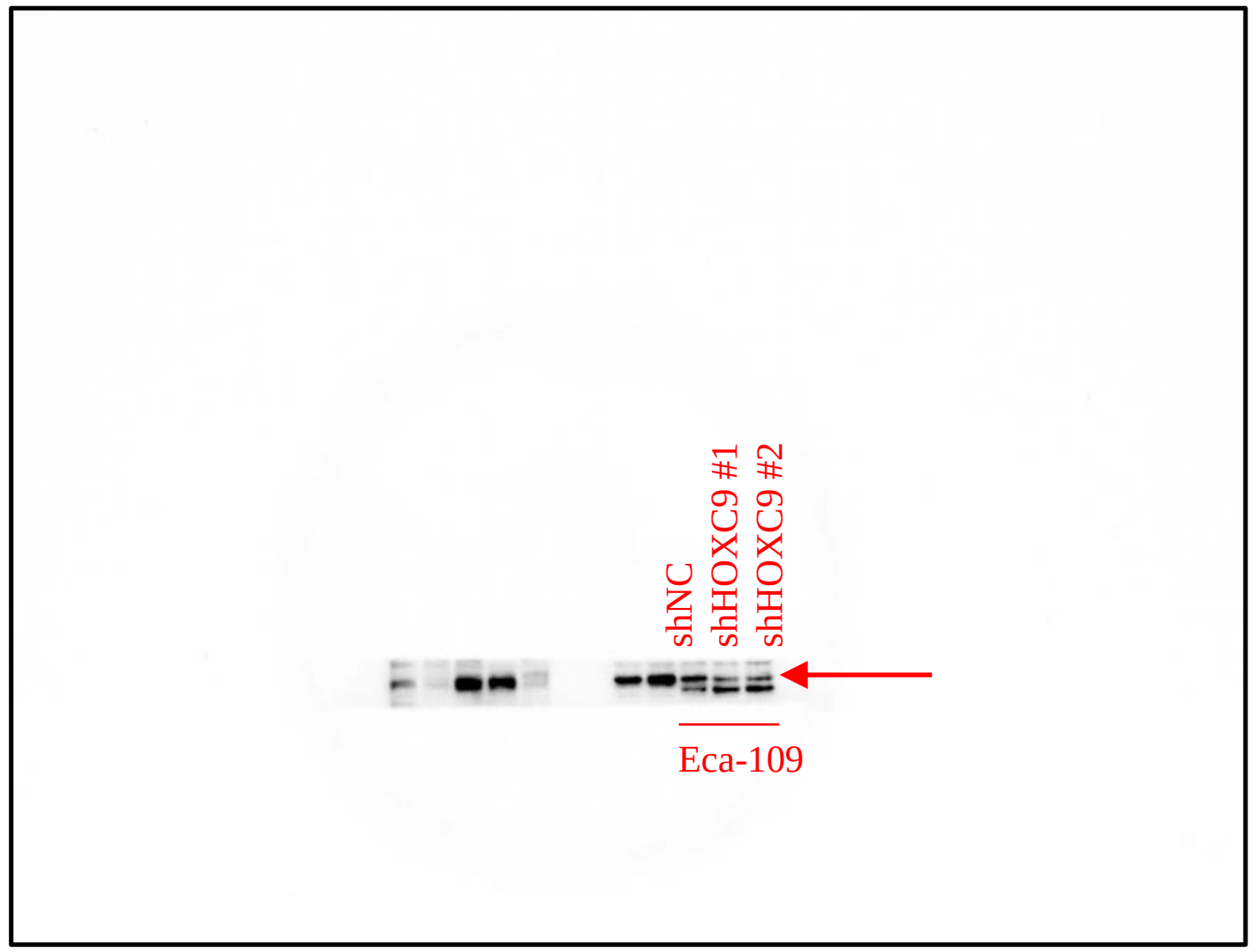

Fig. 5D OTUD1

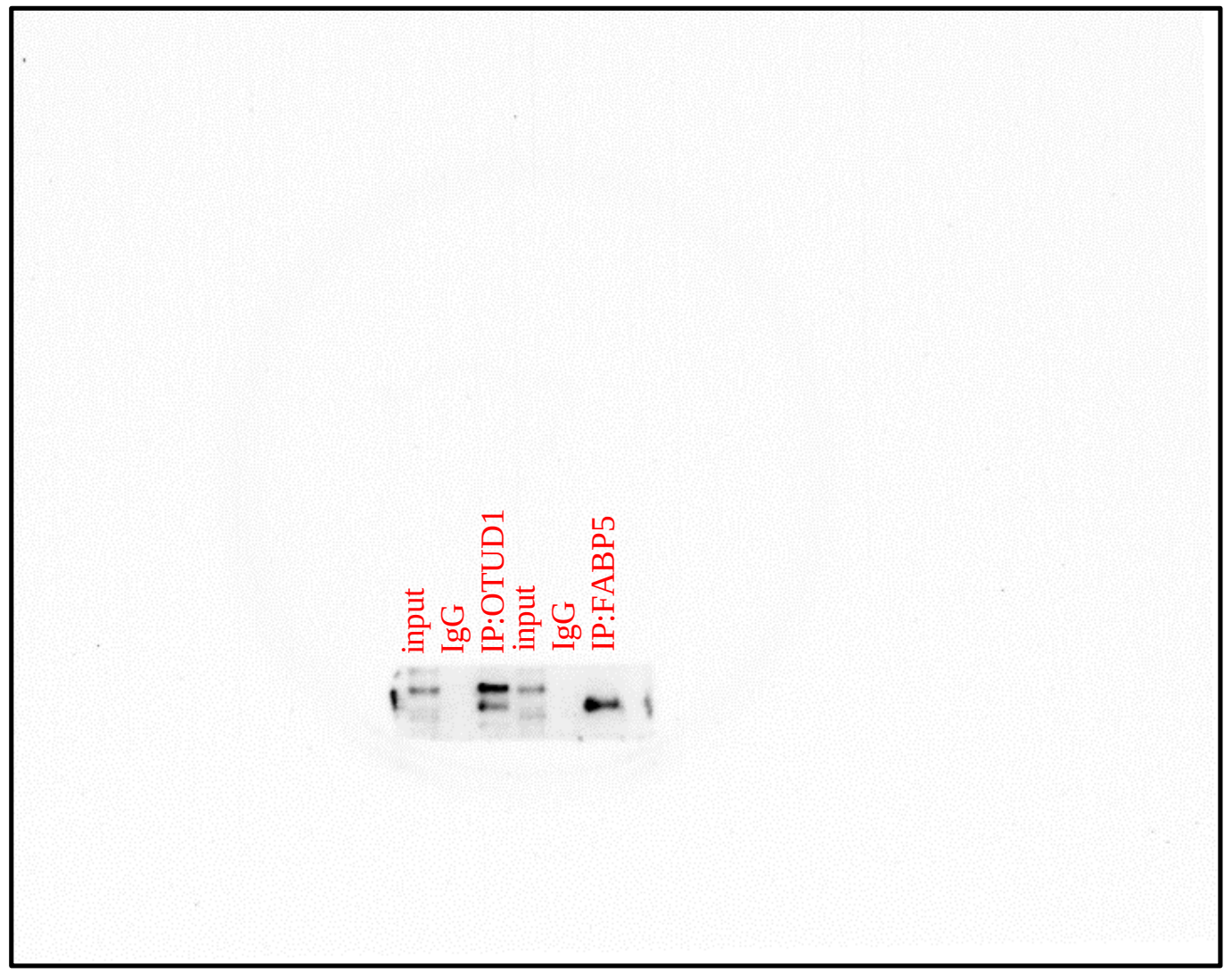

Fig. 5D FABP5-high exposure

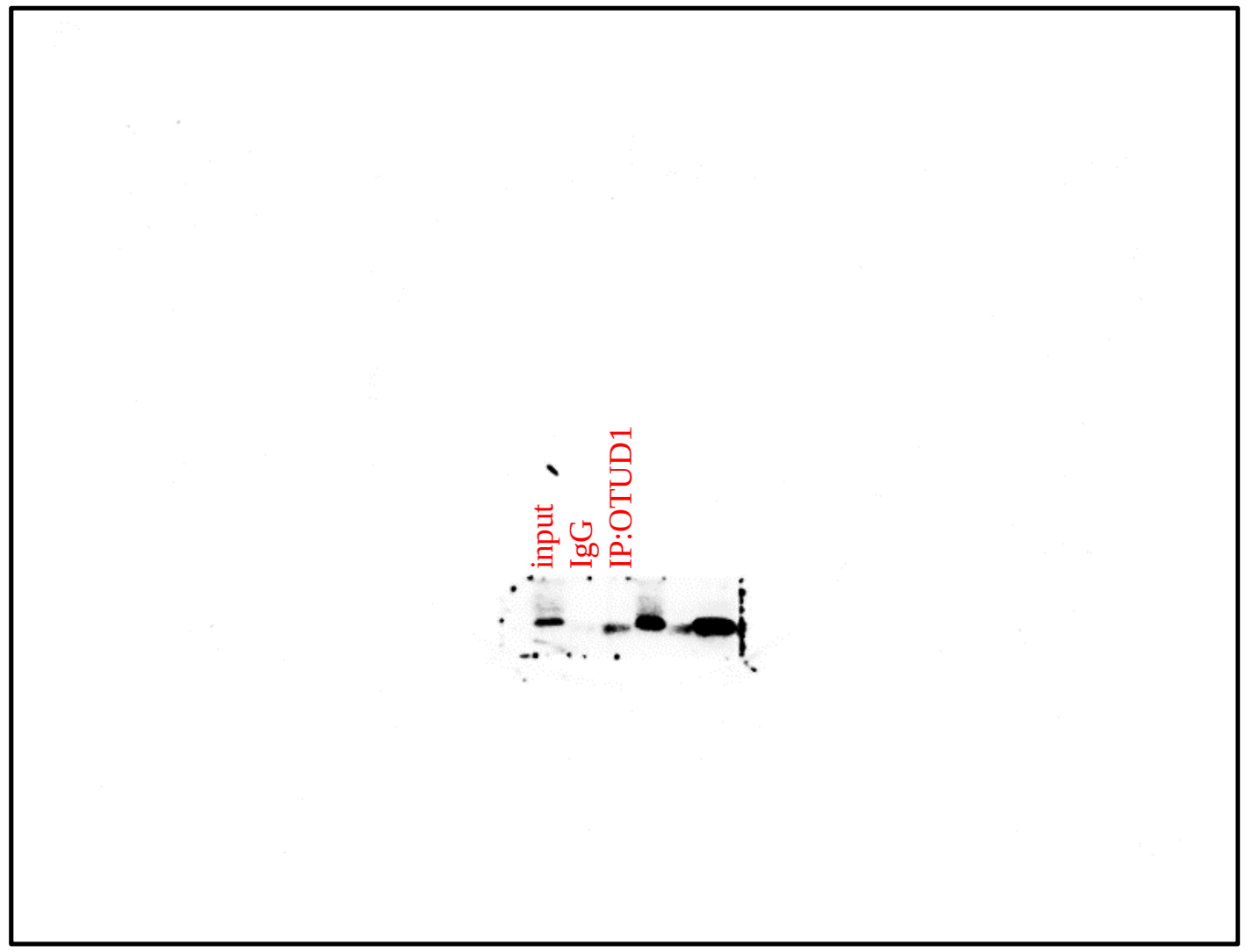

Fig. 5D FABP5-low exposure

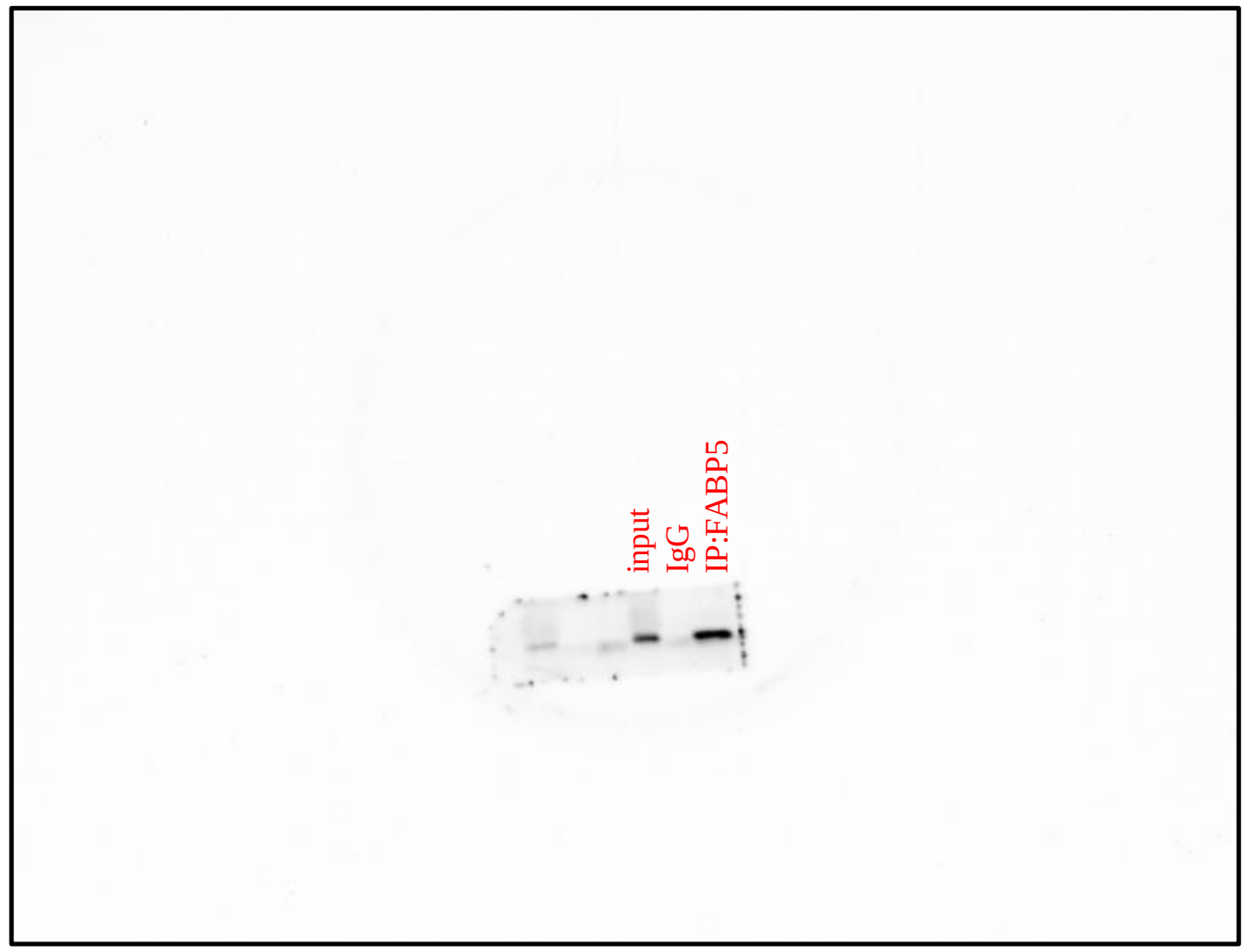

Fig. 5E β-actin

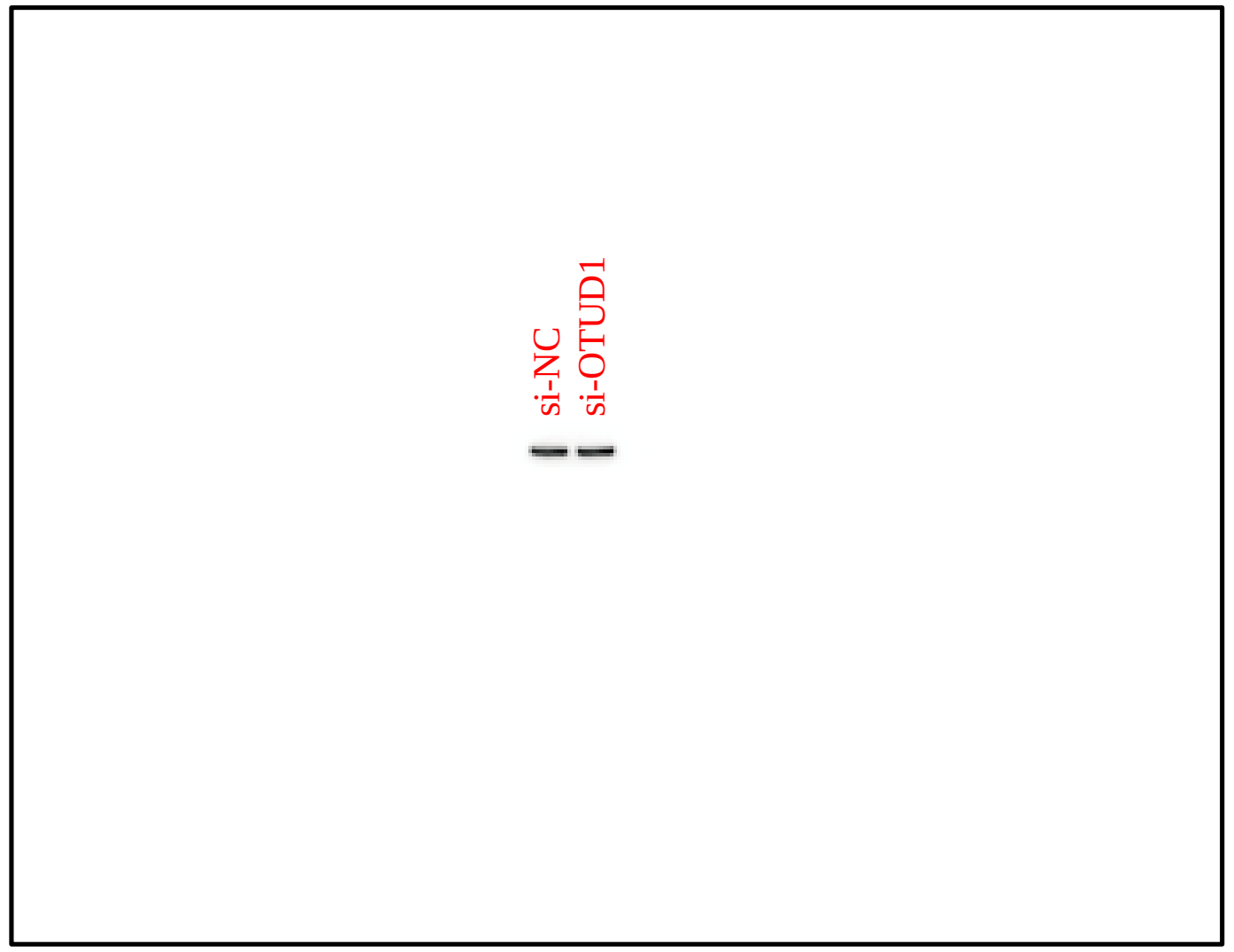

Fig. 5E OTUD1

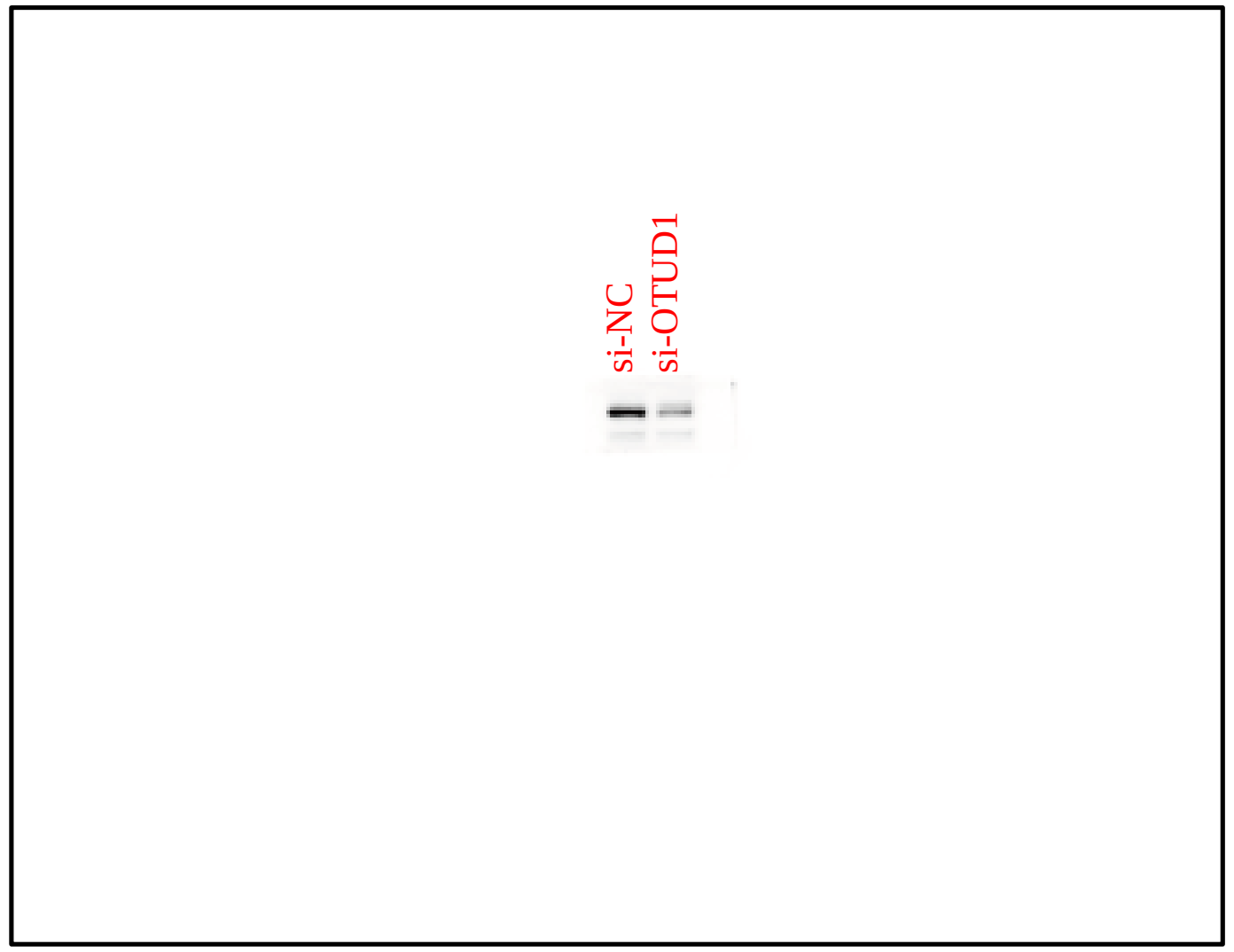

Fig. 5E FABP5

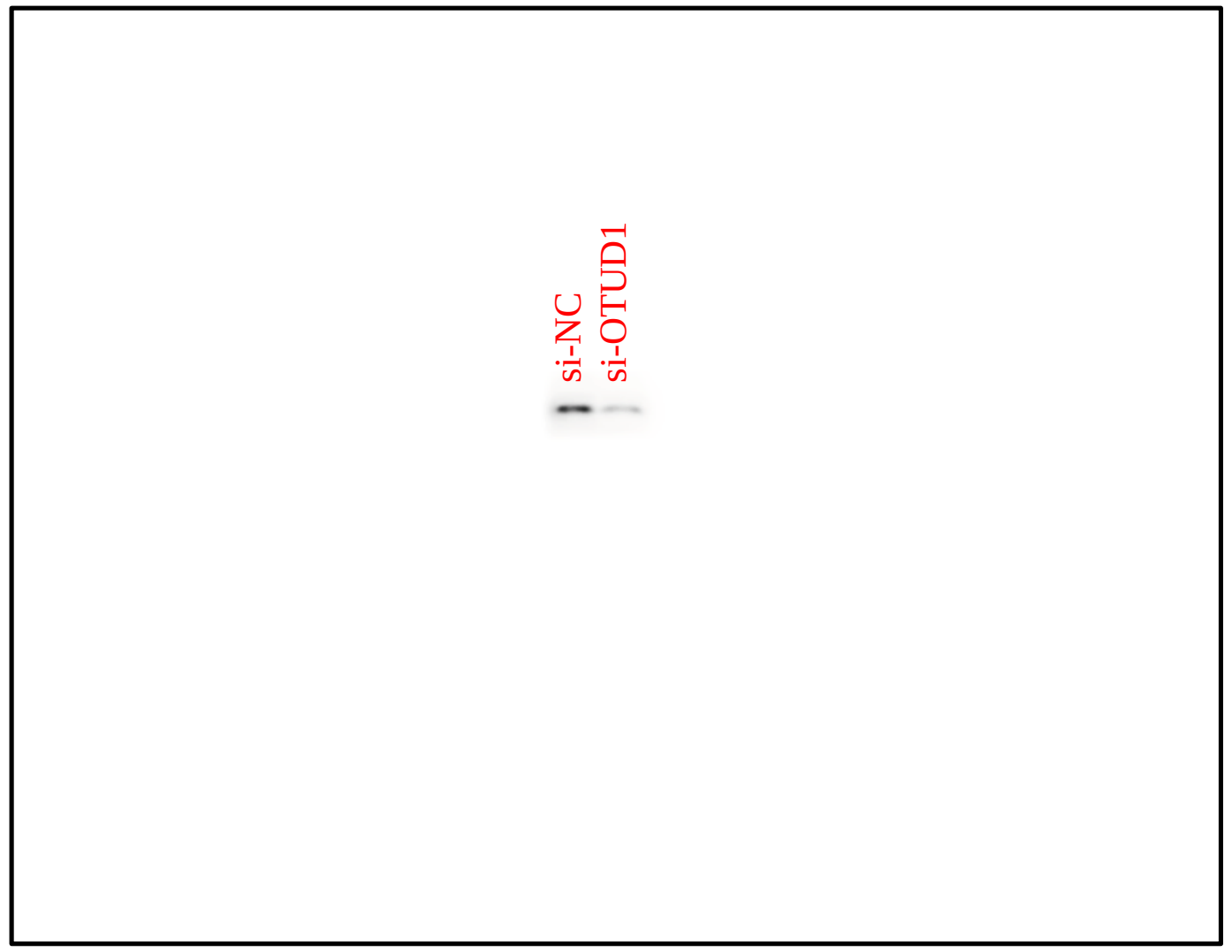

Fig. 5E β-actin

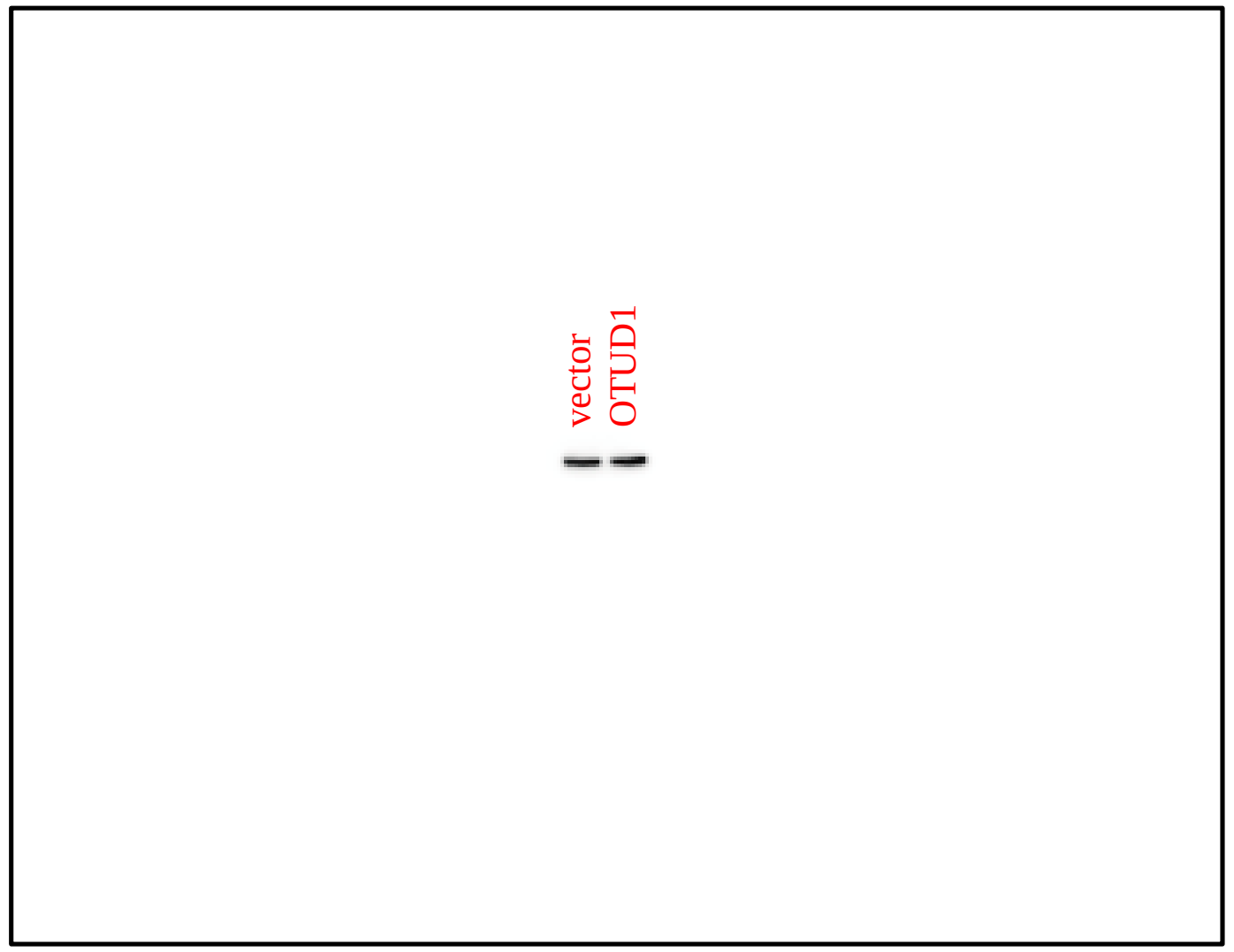

Fig. 5E OTUD1

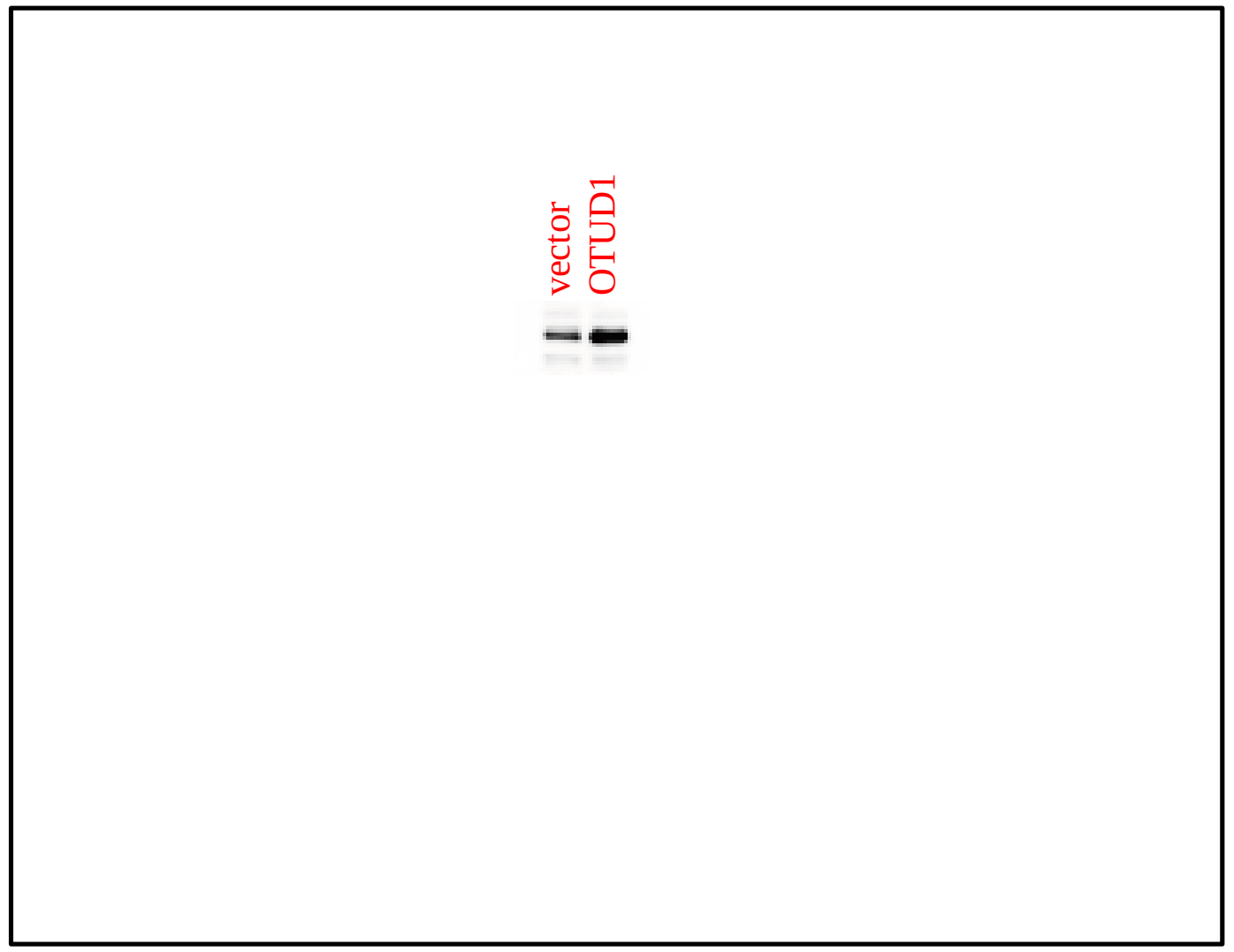

Fig. 5E FABP5

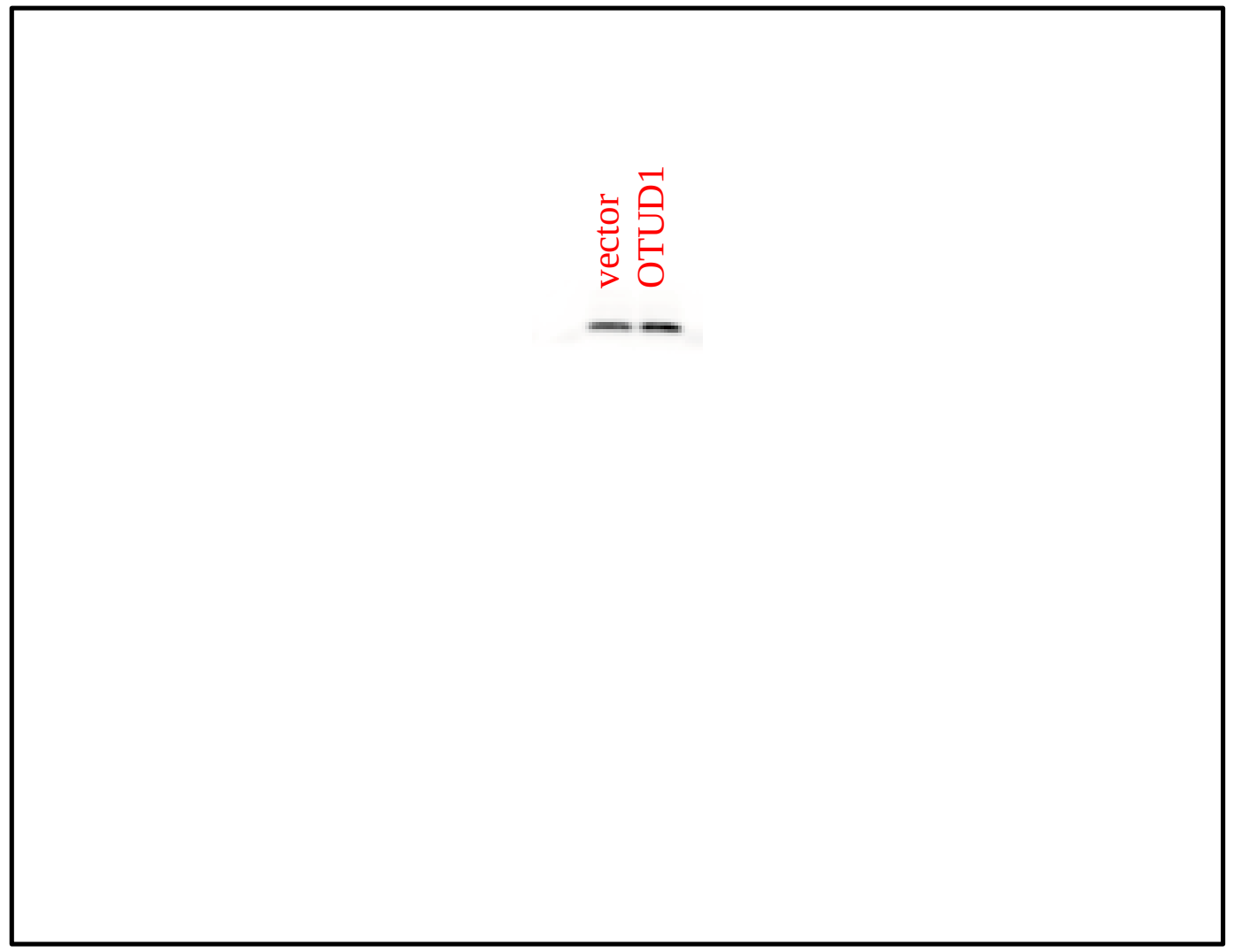

Fig. 5F β-actin

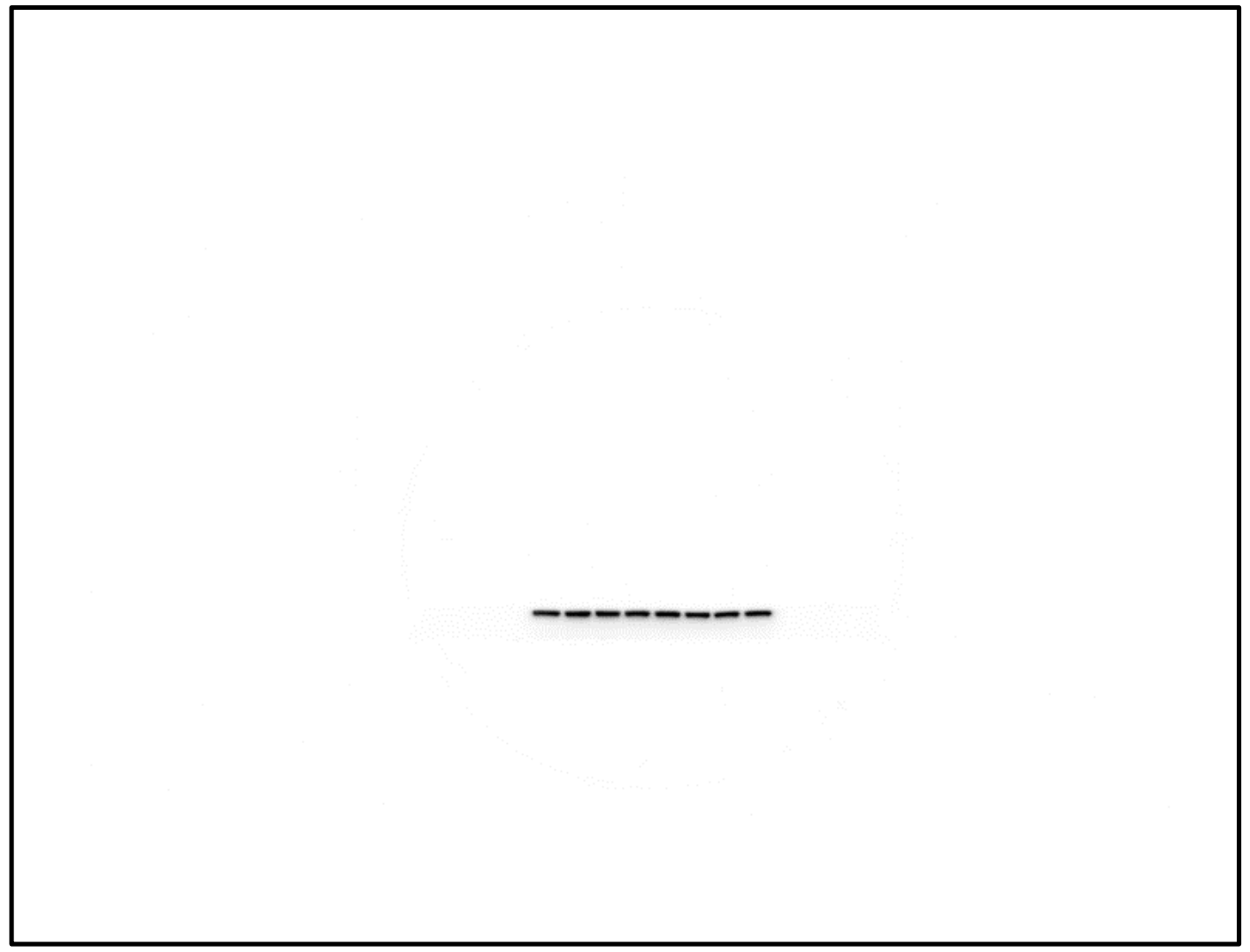

Fig. 5F OTUD1

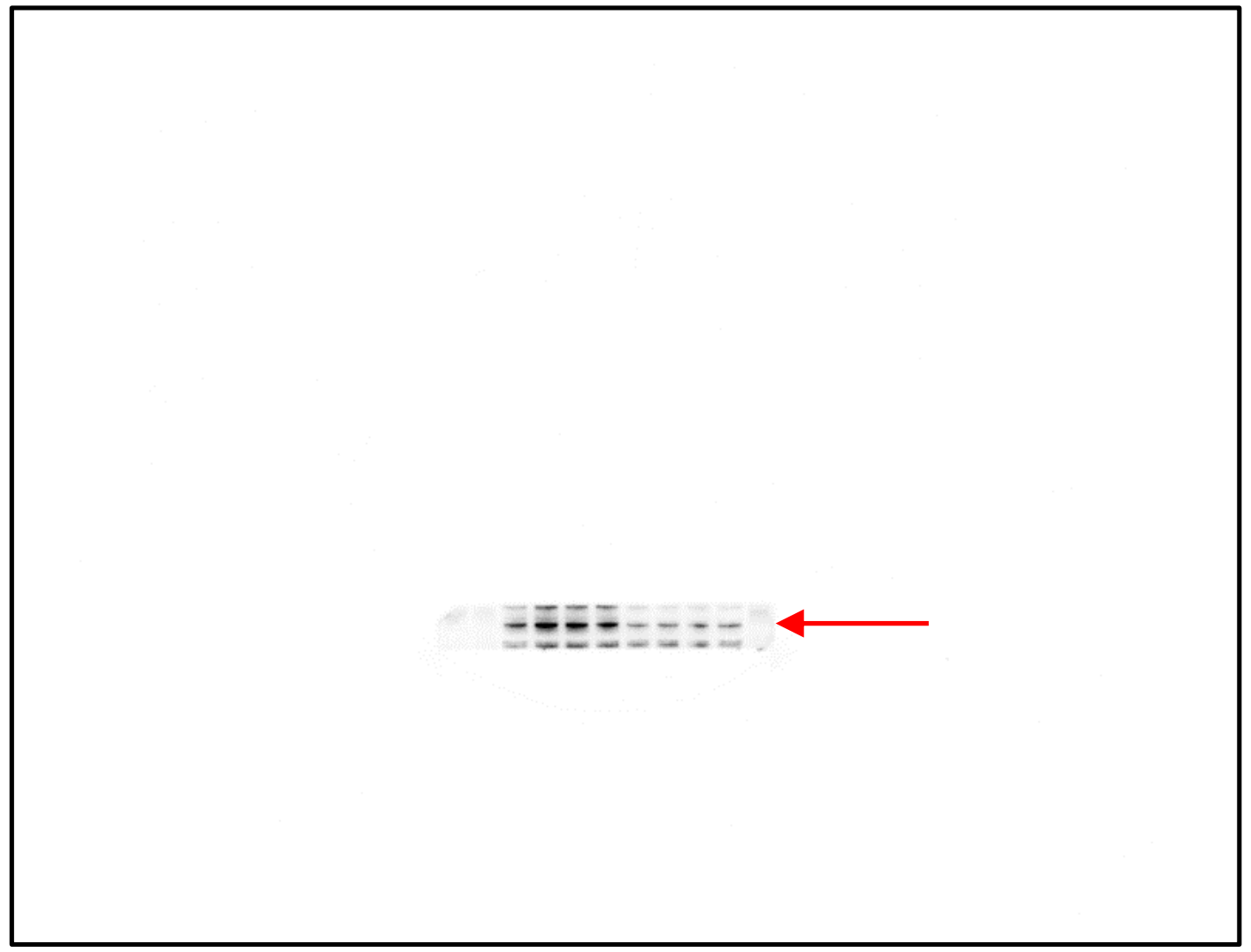

Fig. 5F FABP5

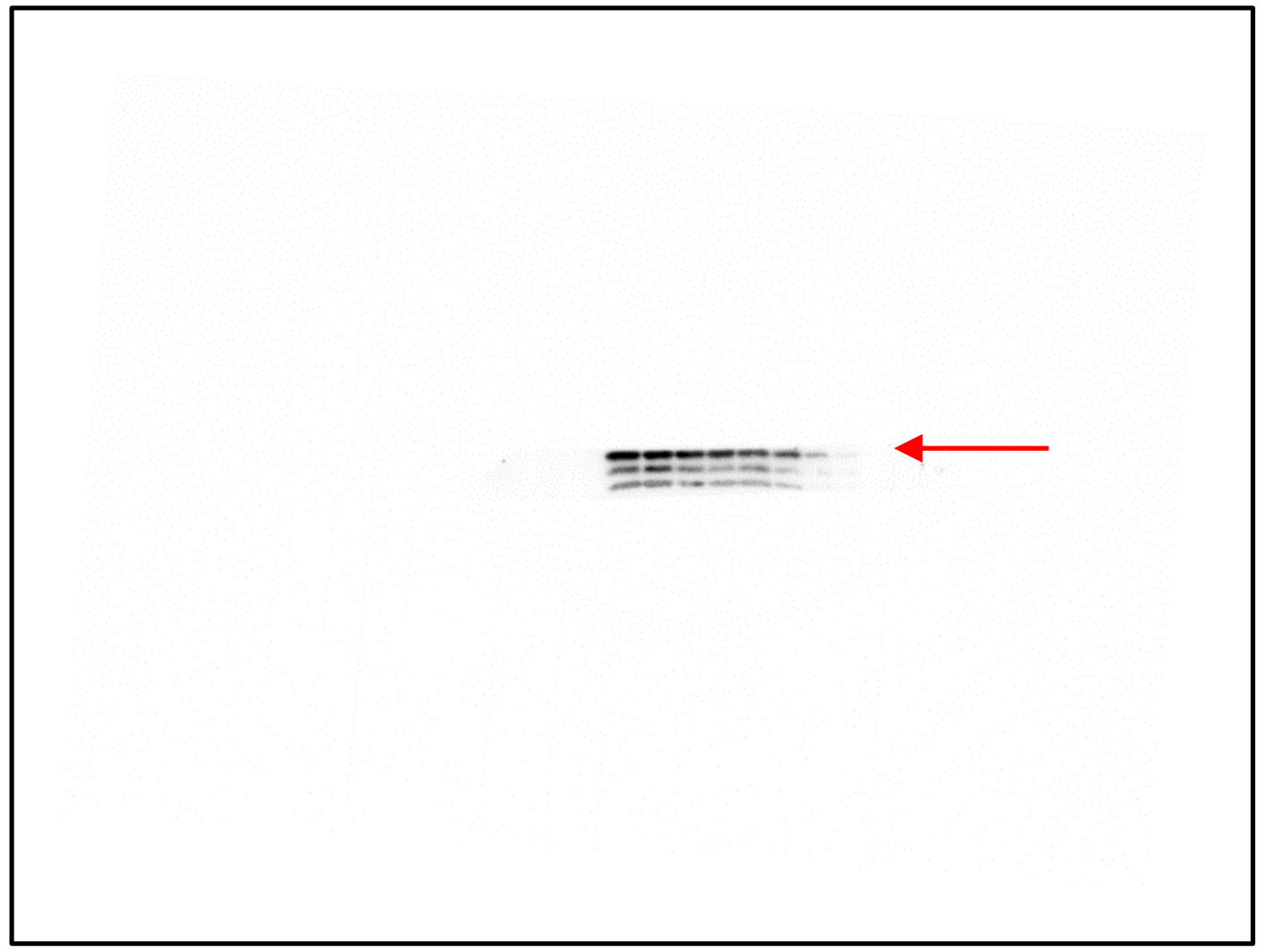

Fig. 5G β-actin

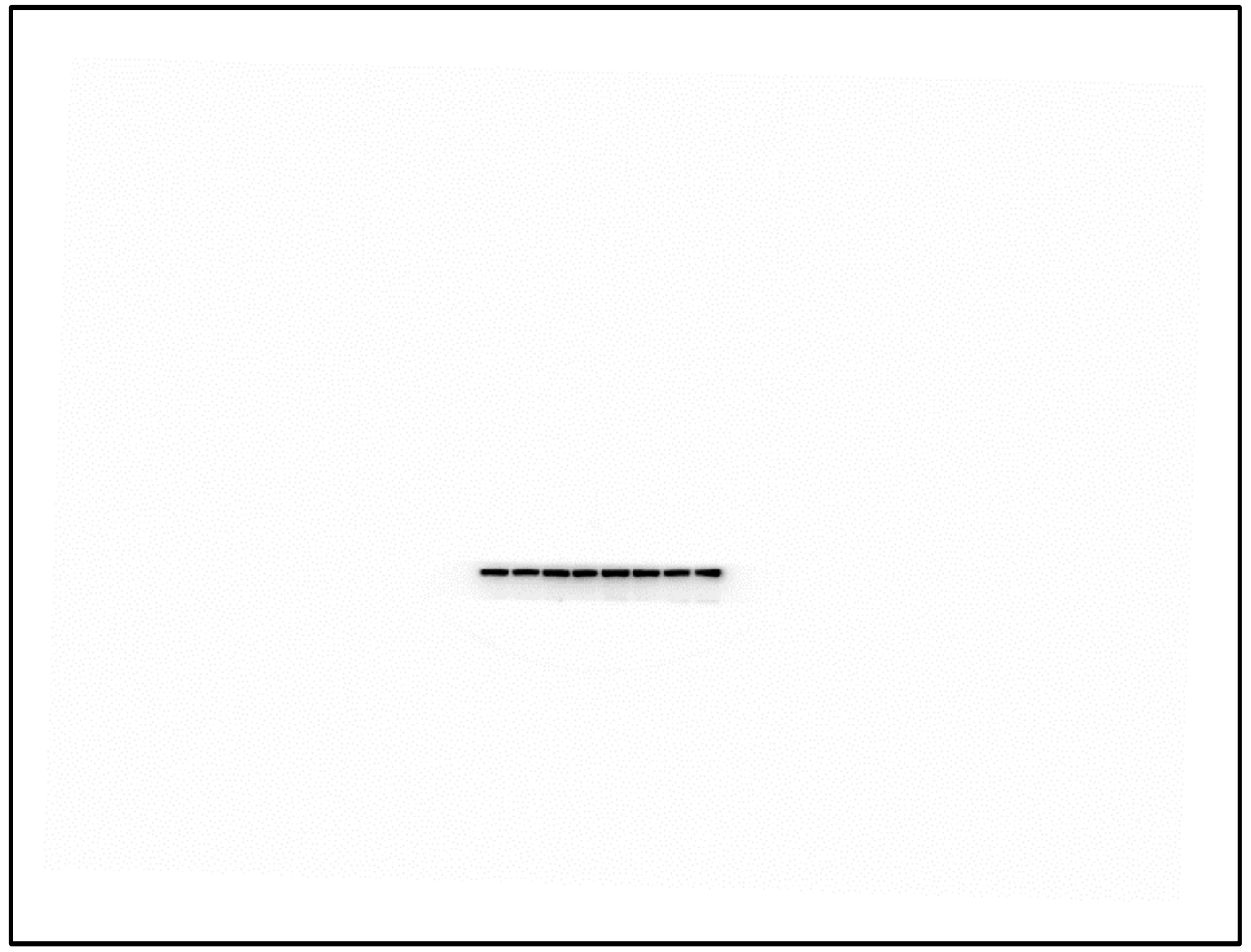

Fig. 5G OTUD1

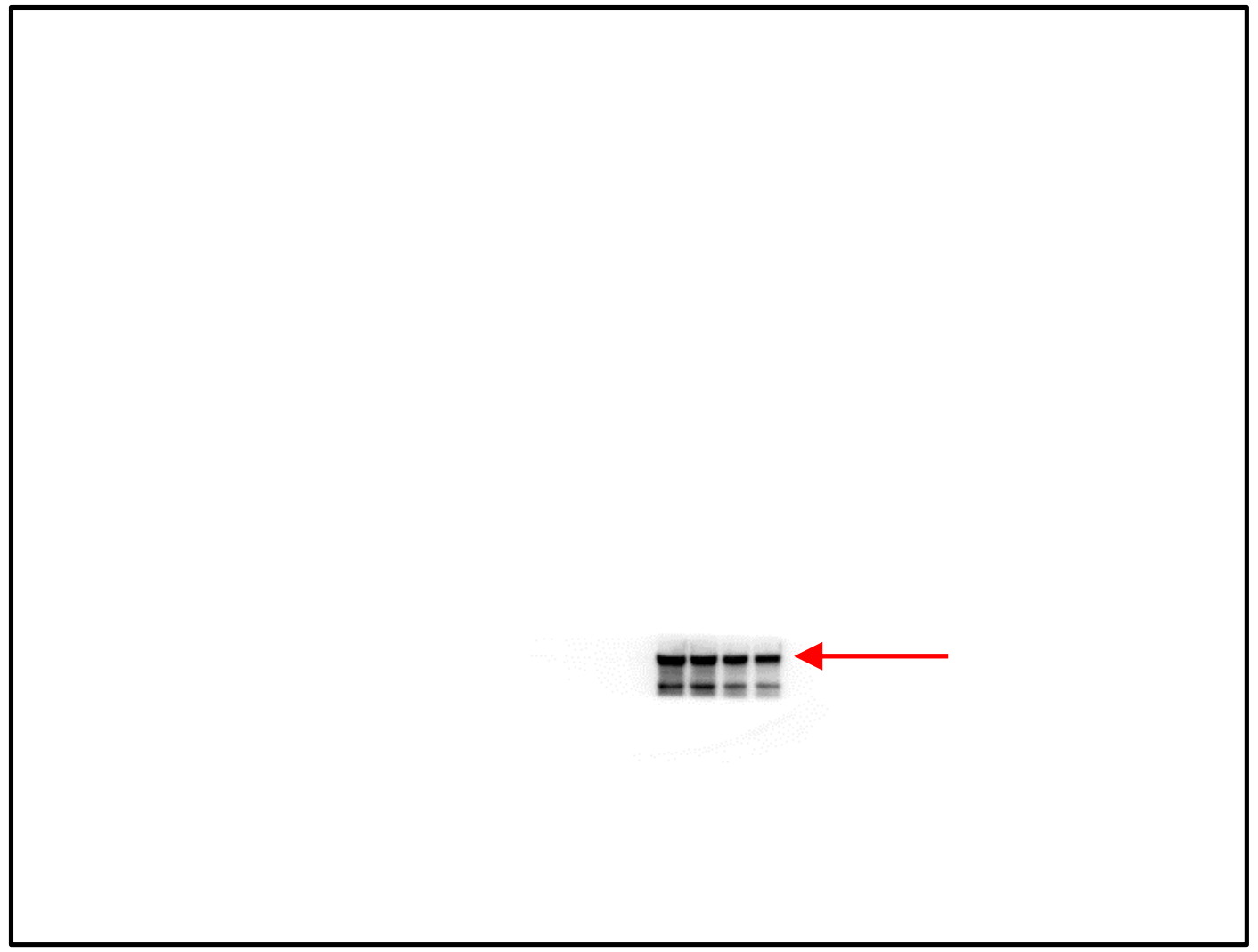

Fig. 5G FABP5

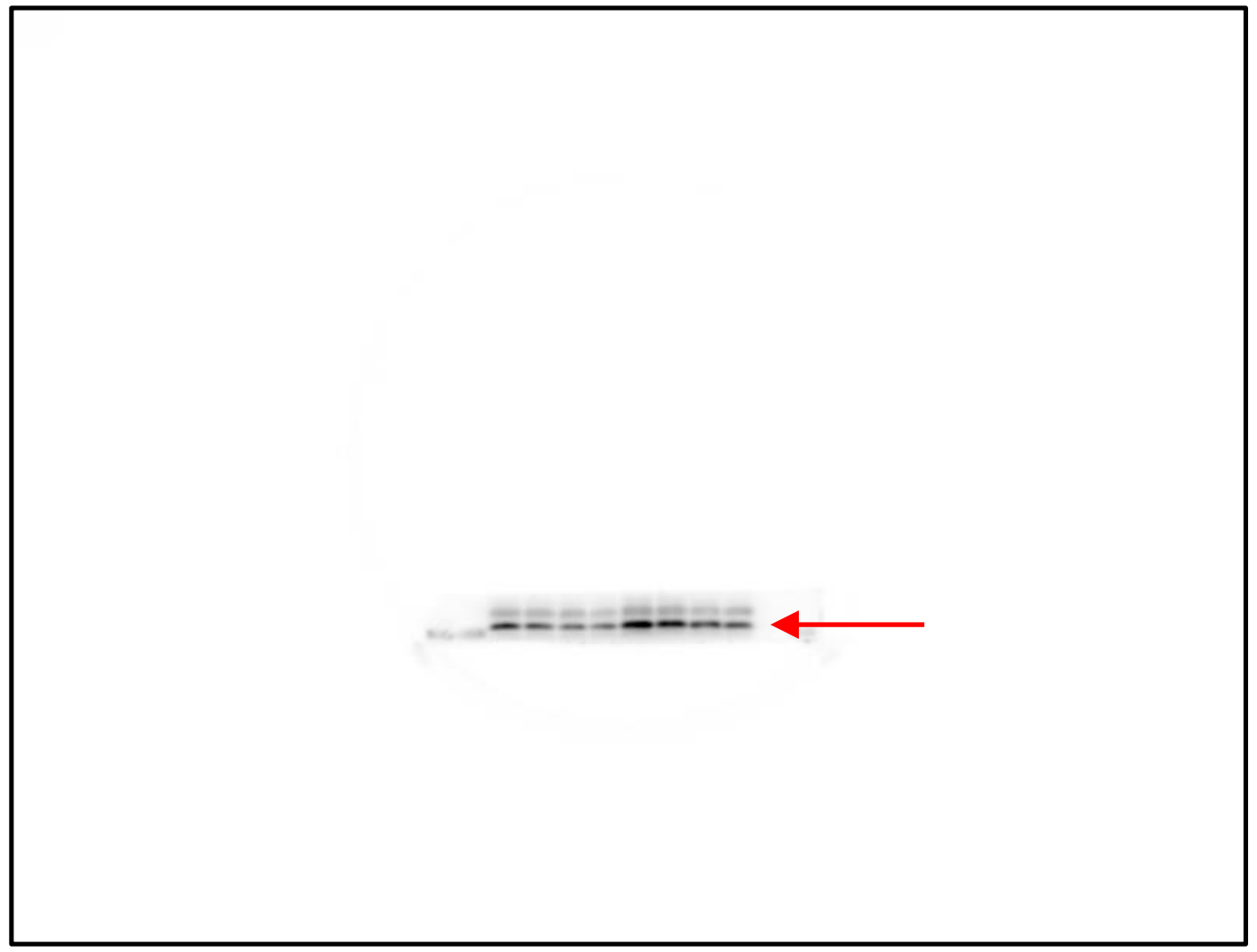

Fig. 5H β-actin

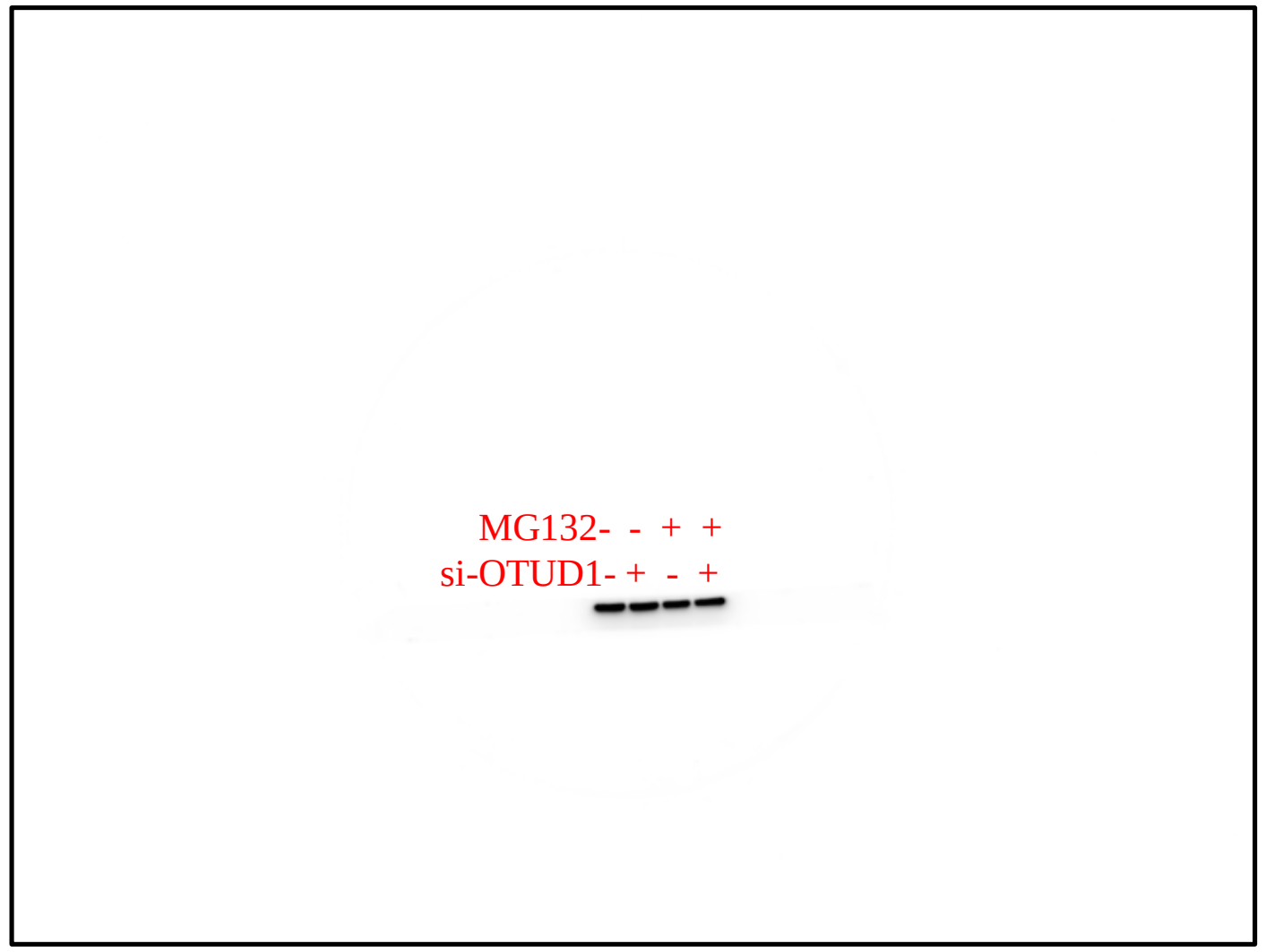

Fig. 5H OTUD1

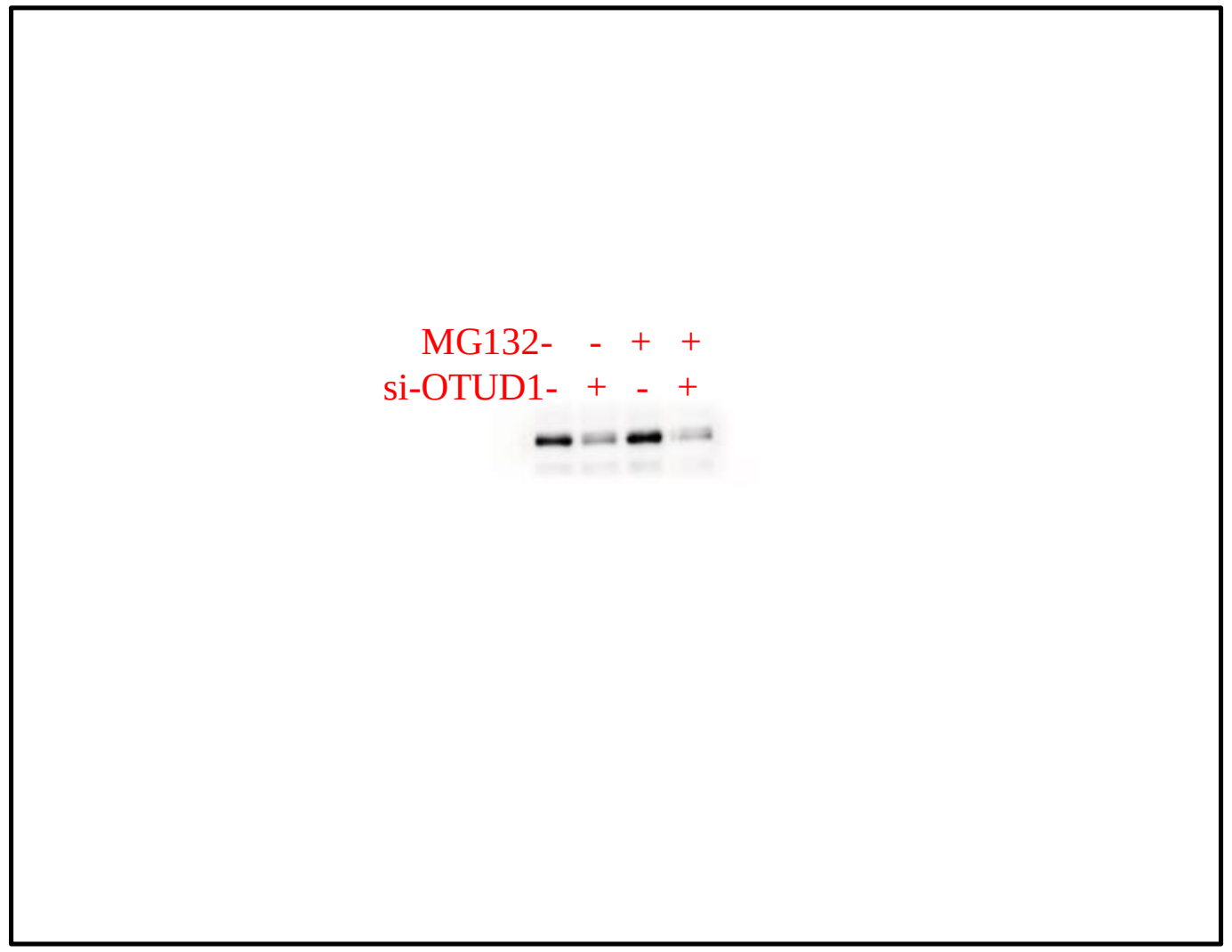

Fig. 5H FABP5

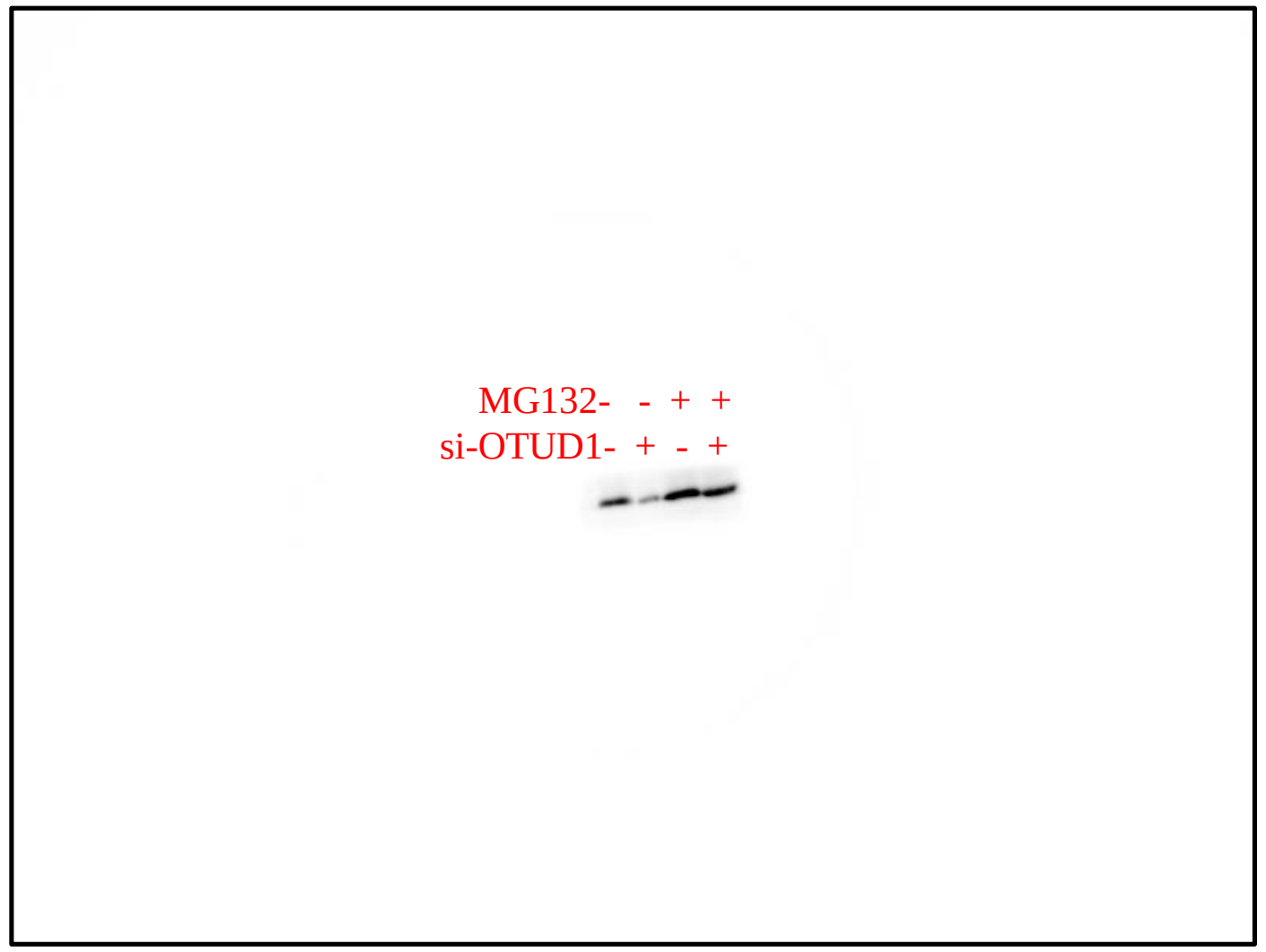

Fig. 5I β-actin

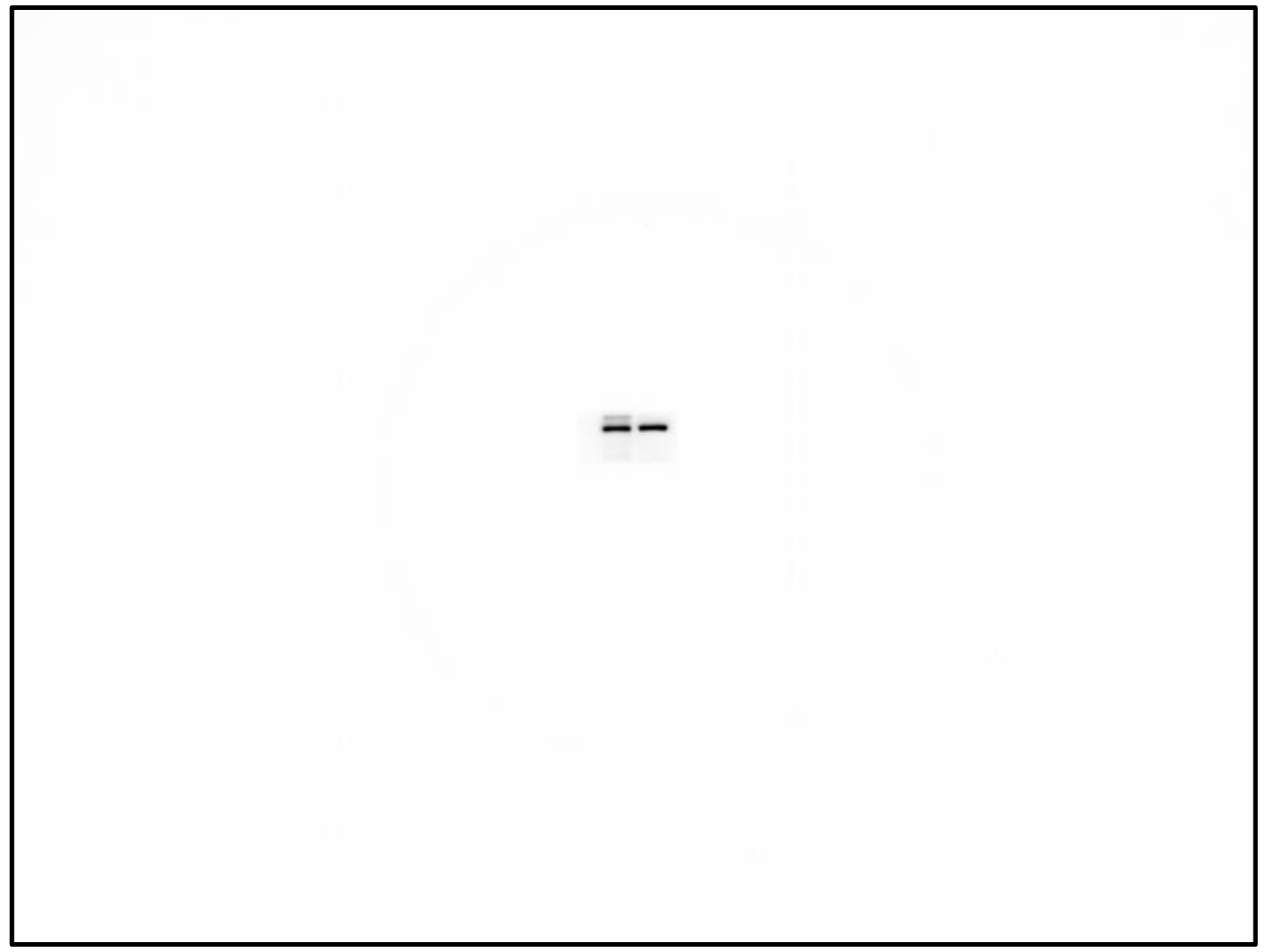

Fig. 5I OTUD1

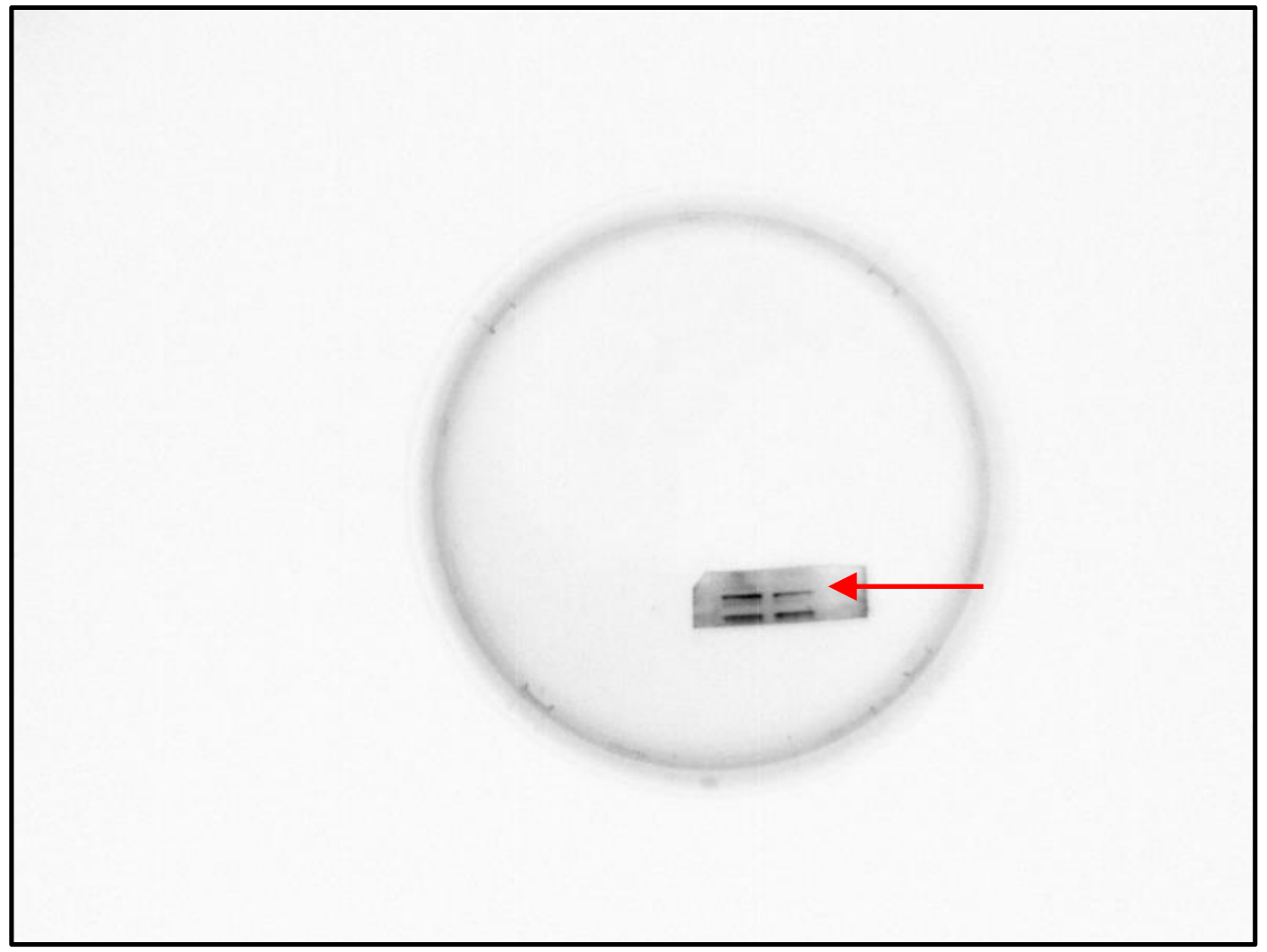

Fig. 5I FABP5-WCL

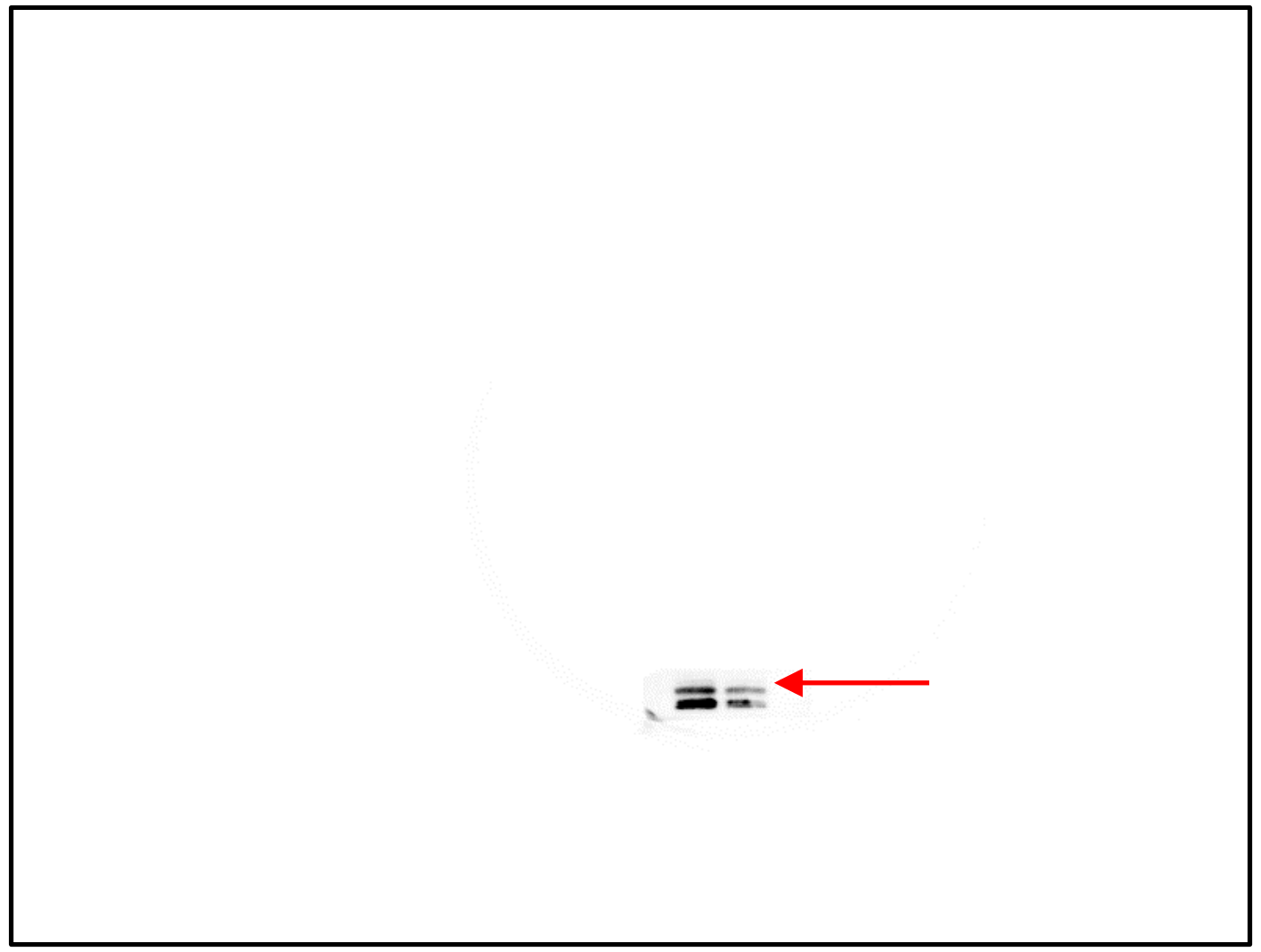

Fig. 5I FABP5-IP

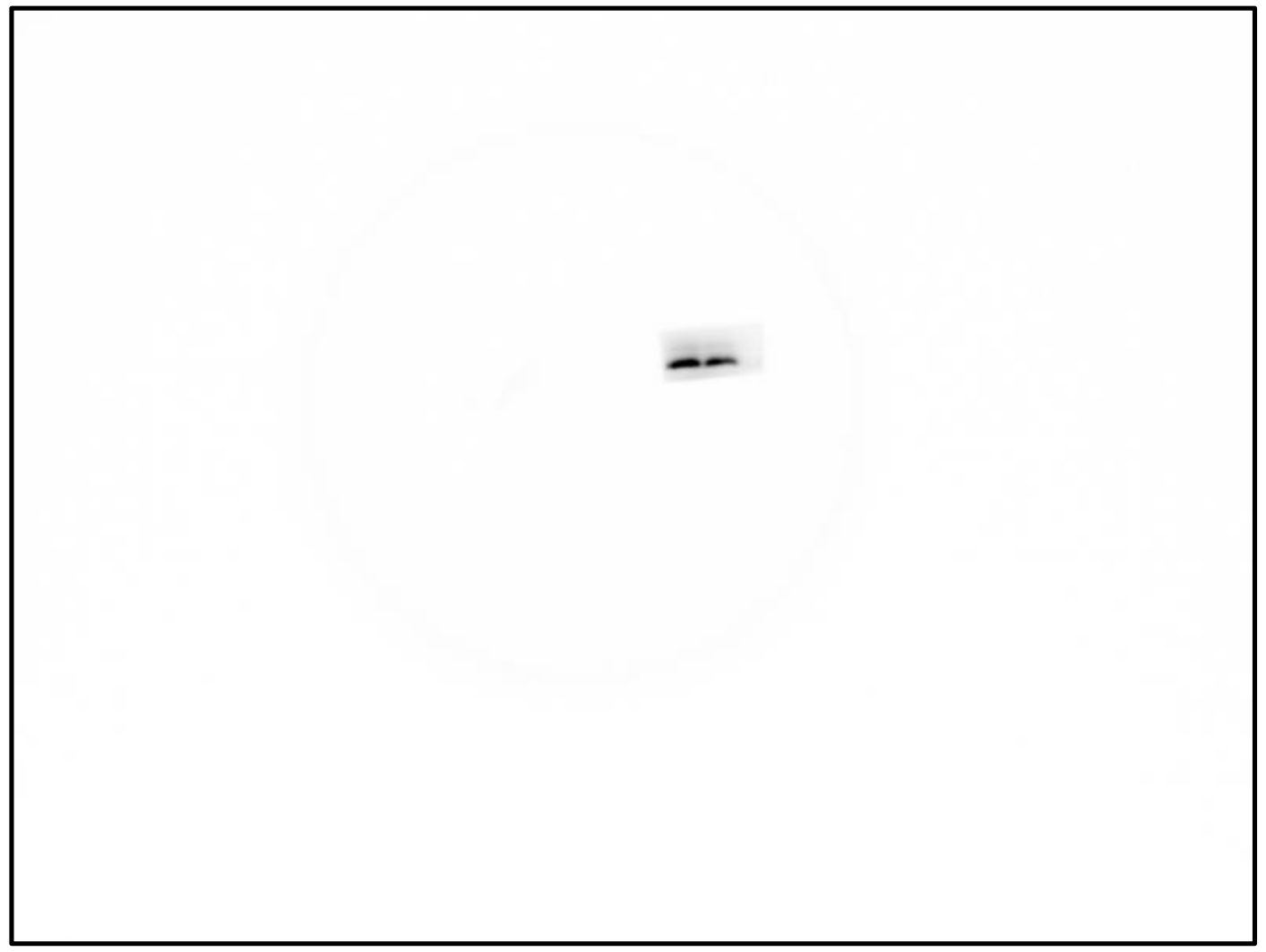

Fig. 5I UB-IP

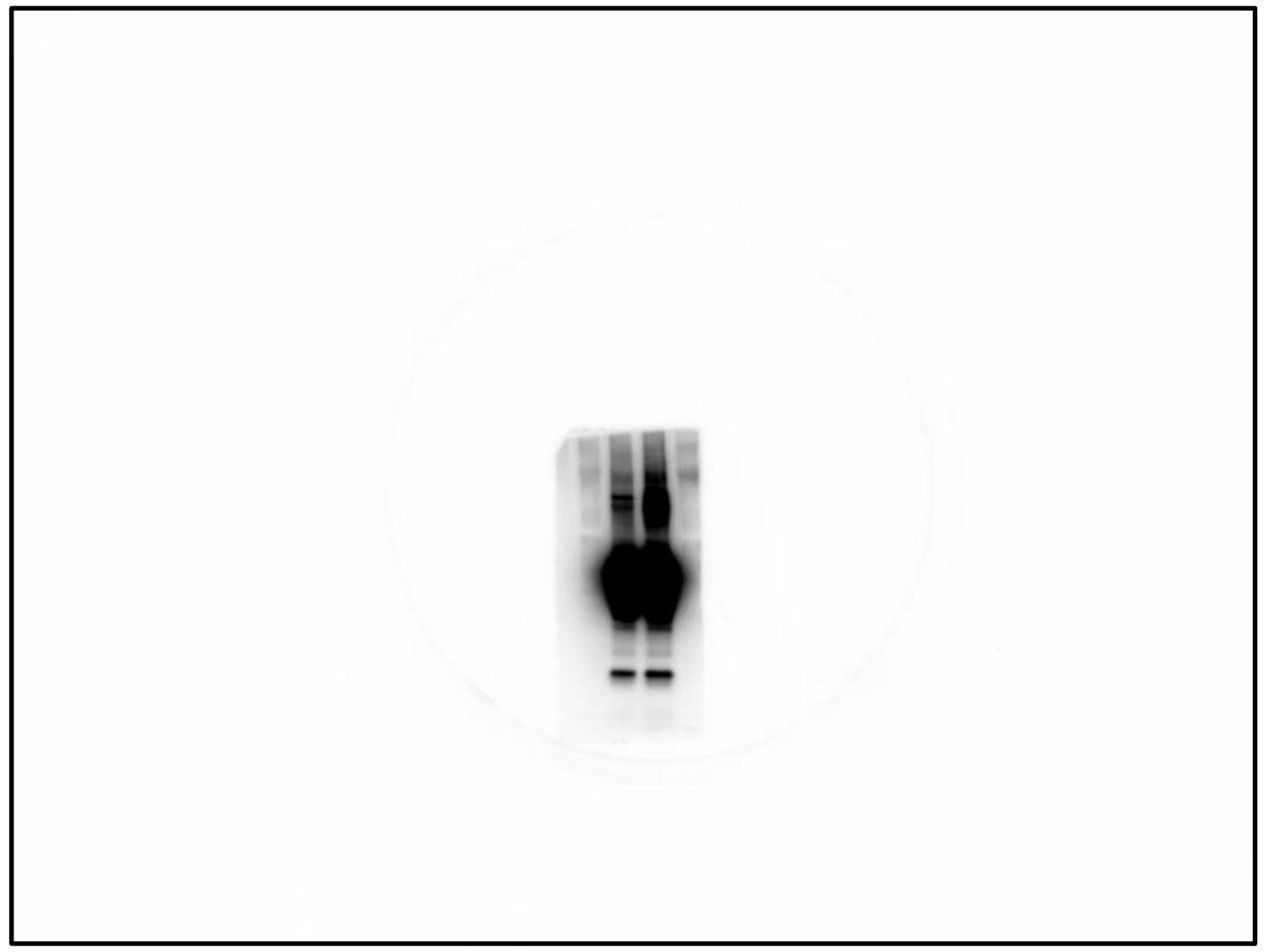

Fig. 6A β-actin

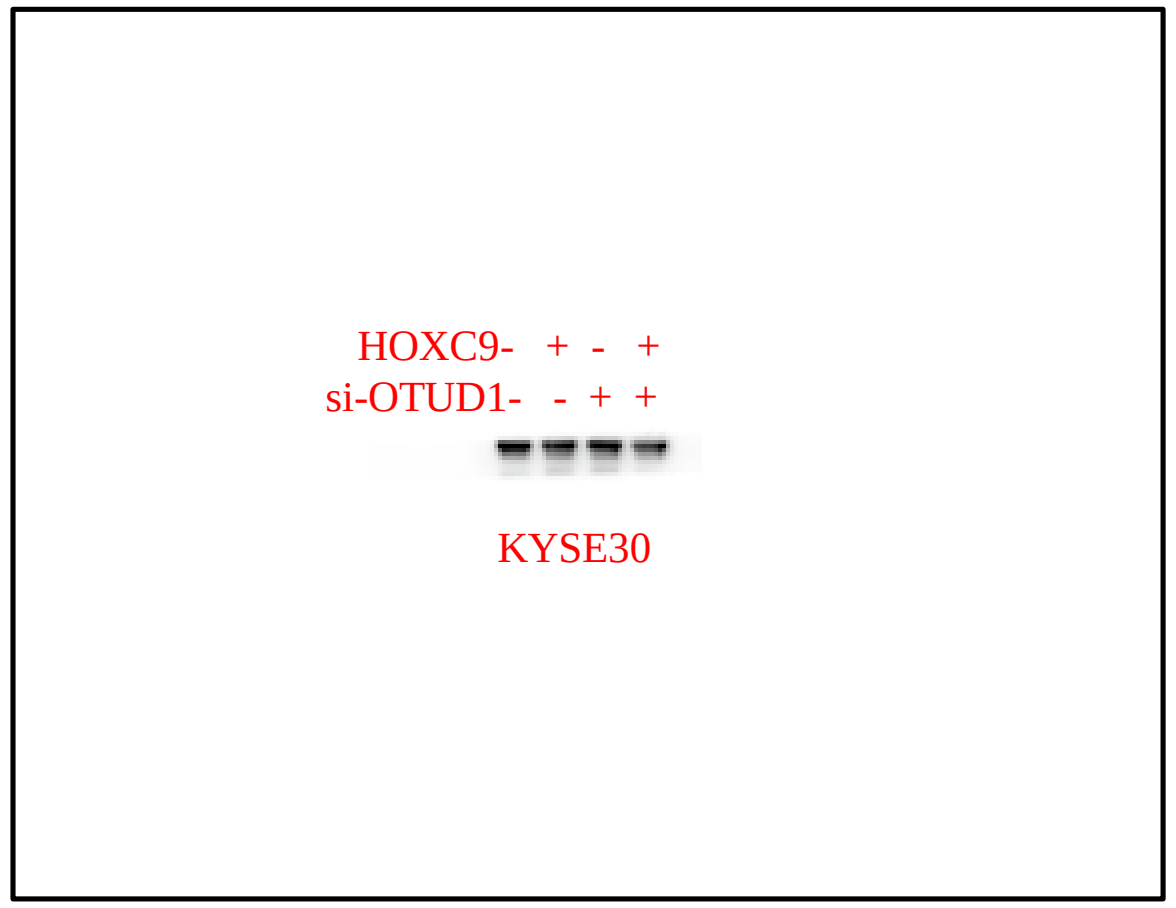

Fig. 6A HOXC9

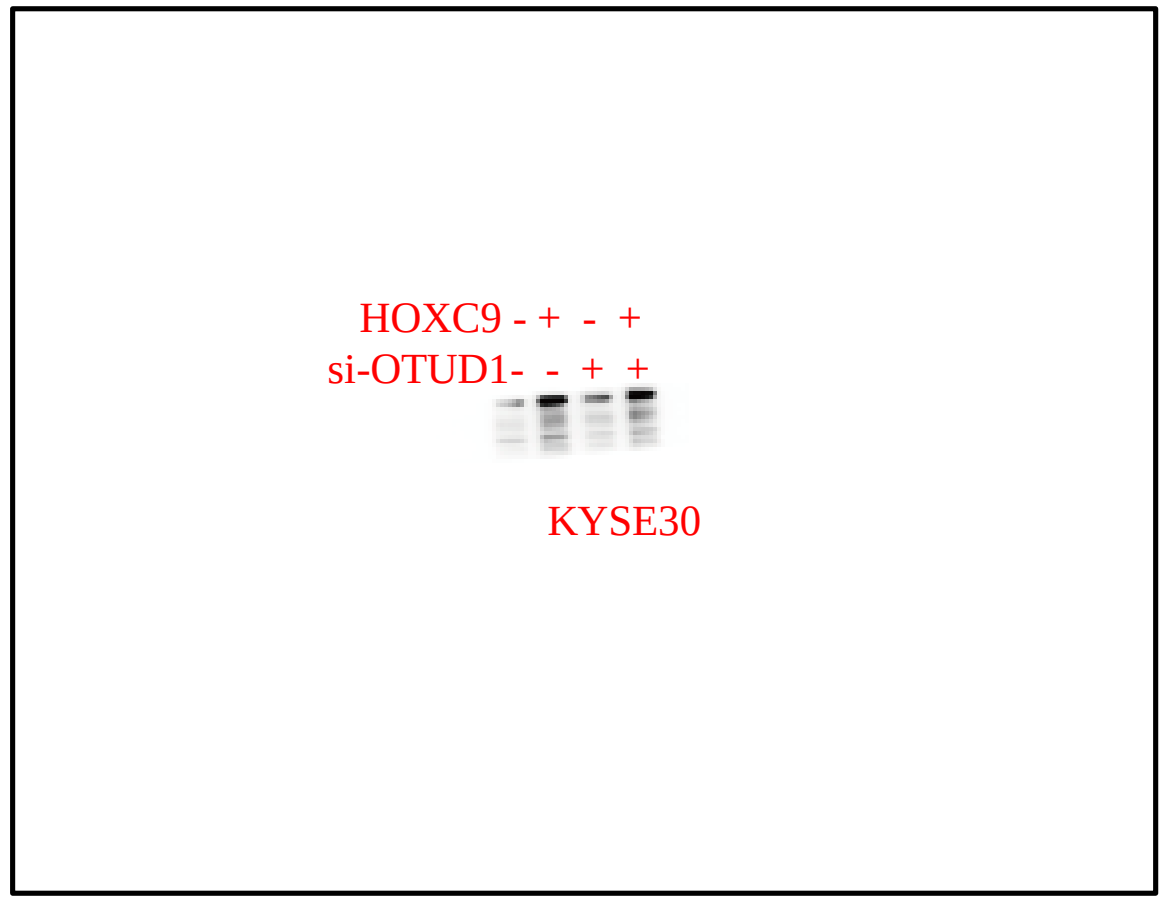

Fig. 6A OTUD1

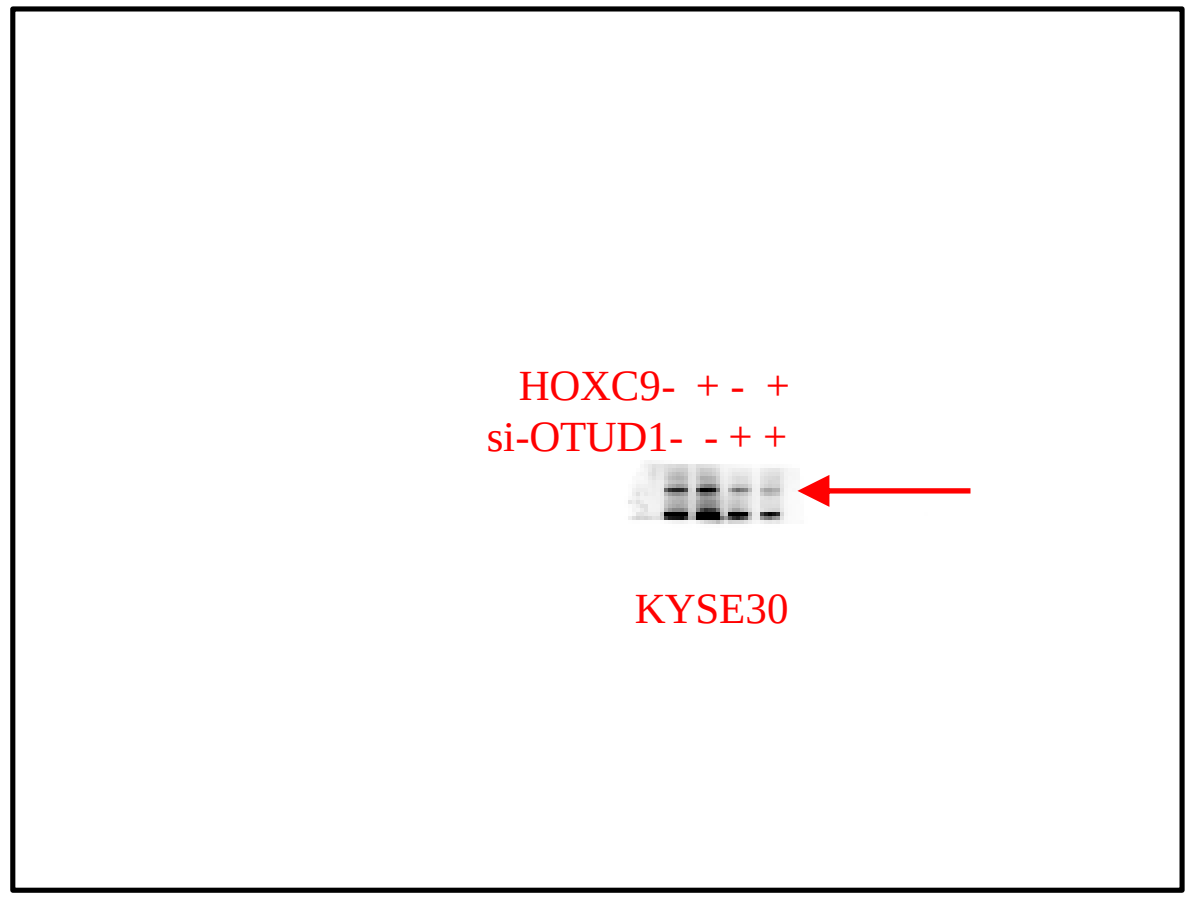

Fig. 6A FABP5

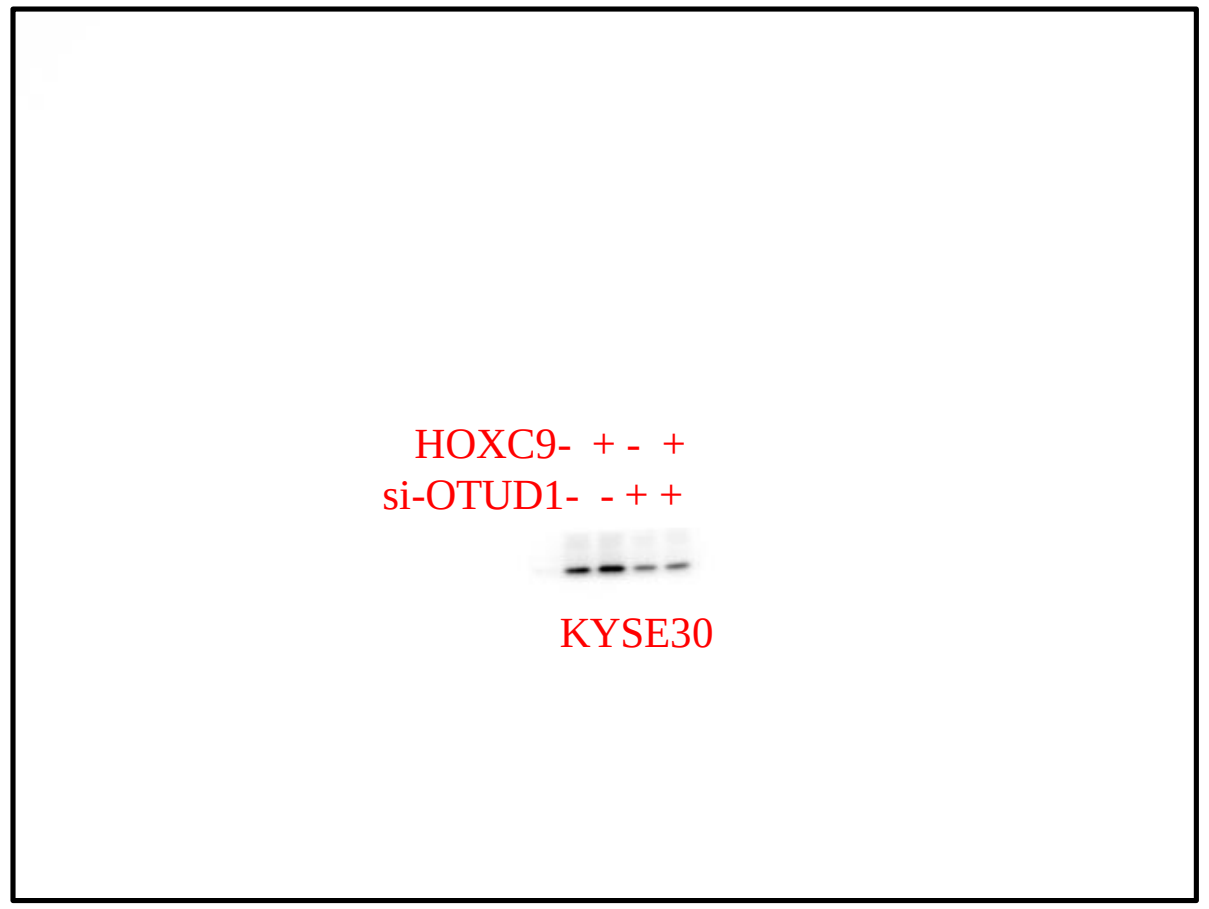

Fig. 6A β-actin

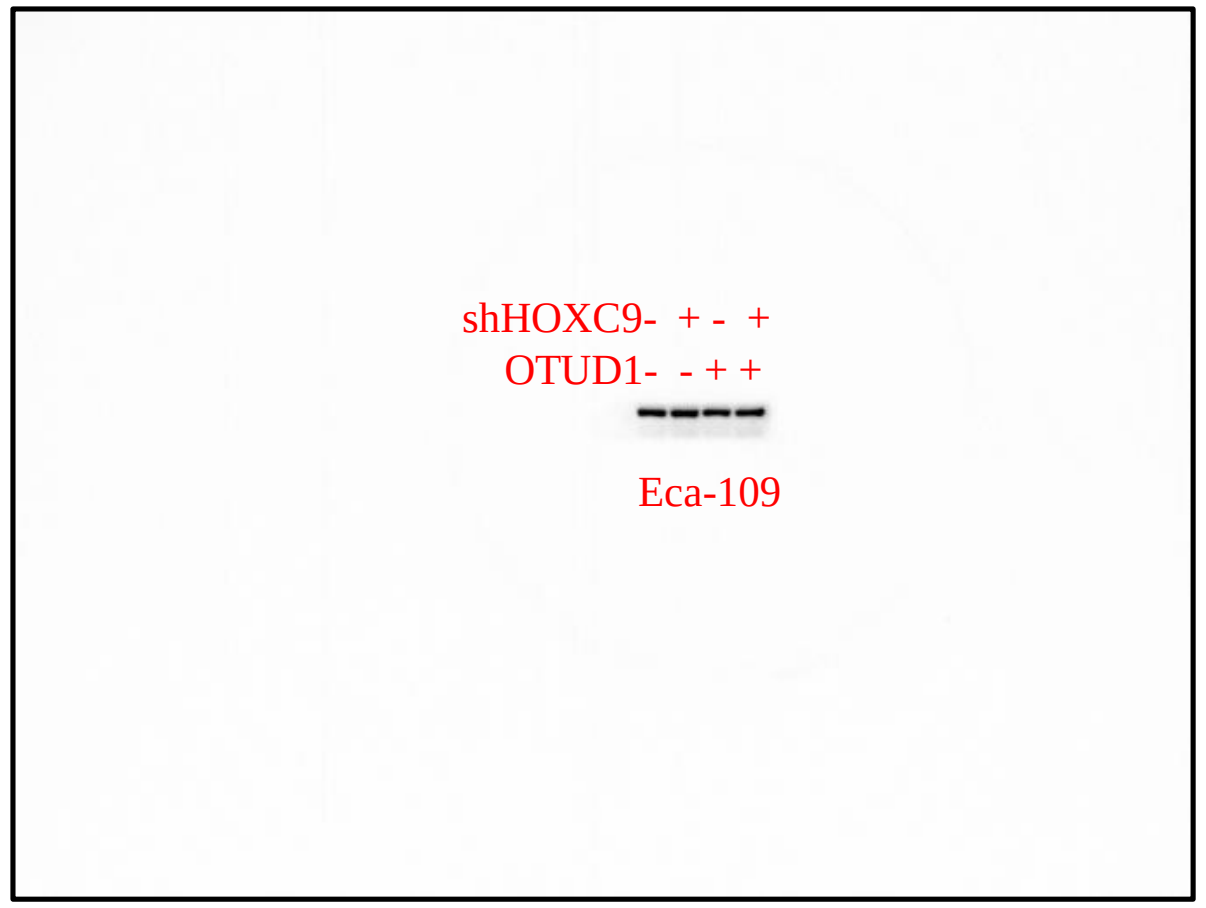

Fig. 6A HOXC9

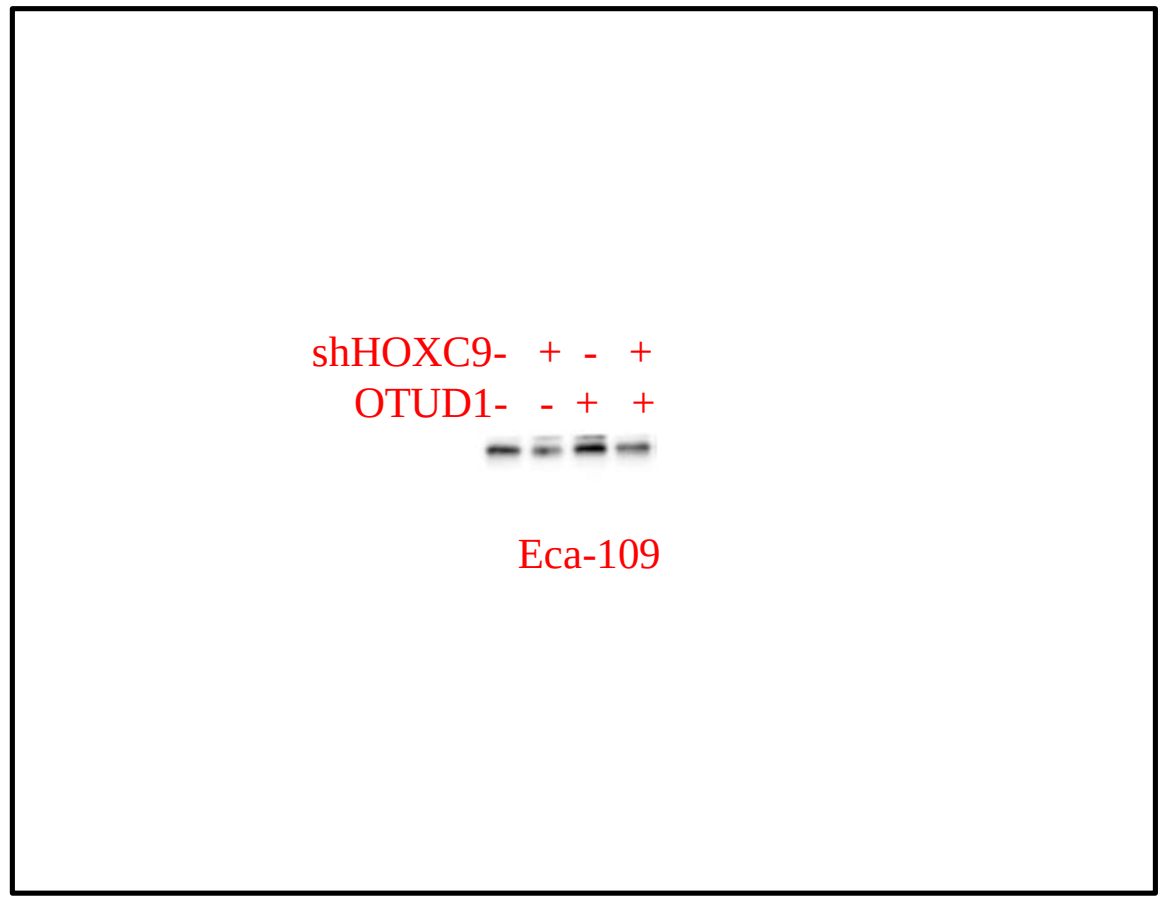

Fig. 6A OTUD1

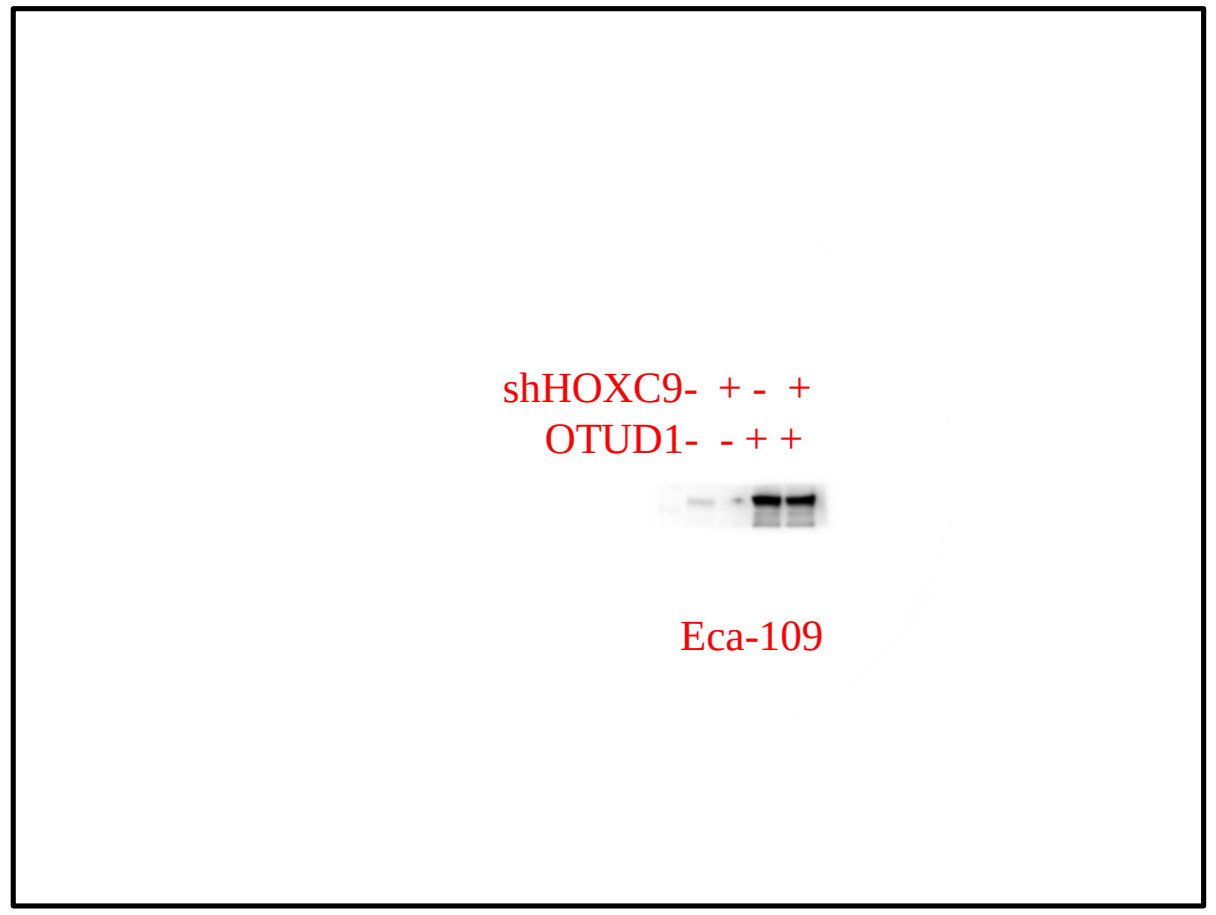

Fig. 6A FABP5

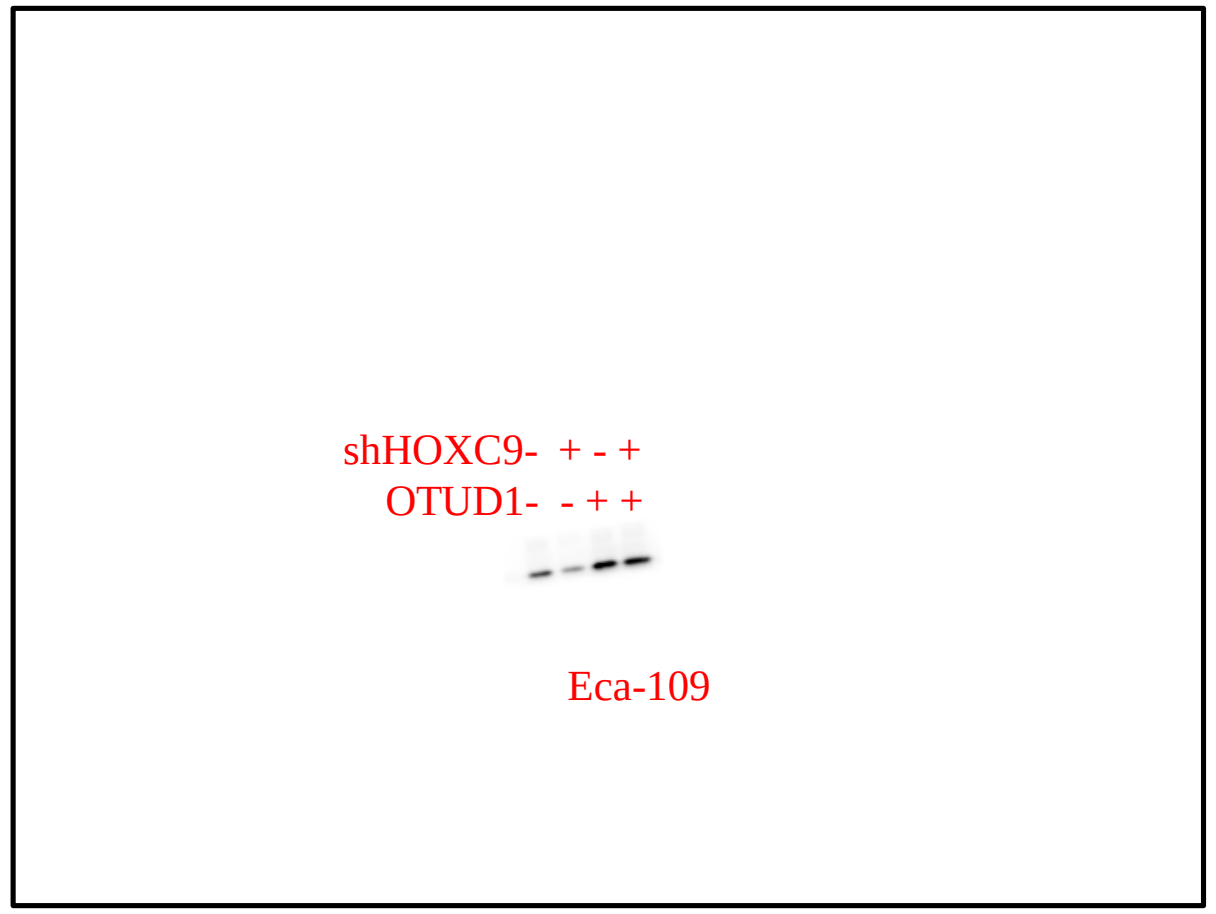

Fig. 6B WCL-β-actin

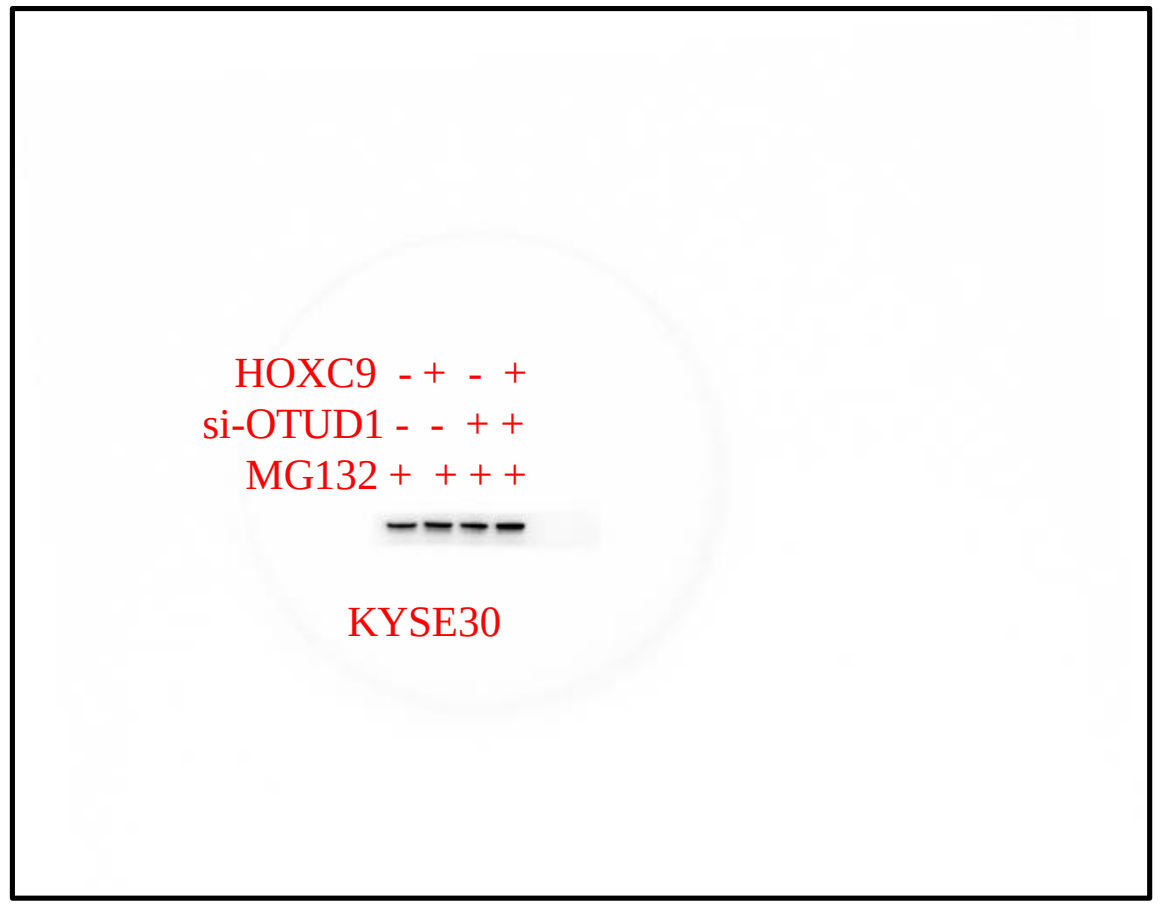

Fig. 6B WCL-HOXC9

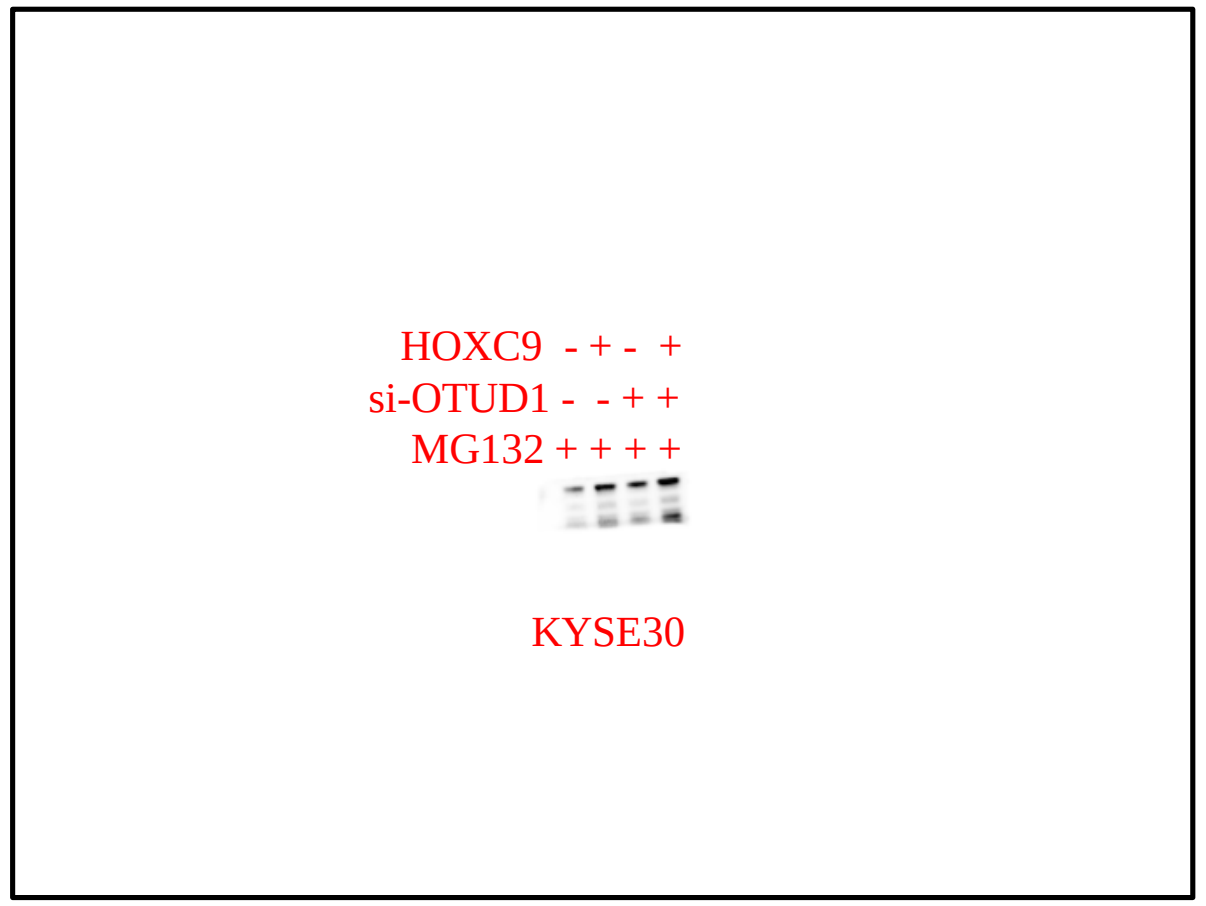

Fig. 6B WCL-OTUD1

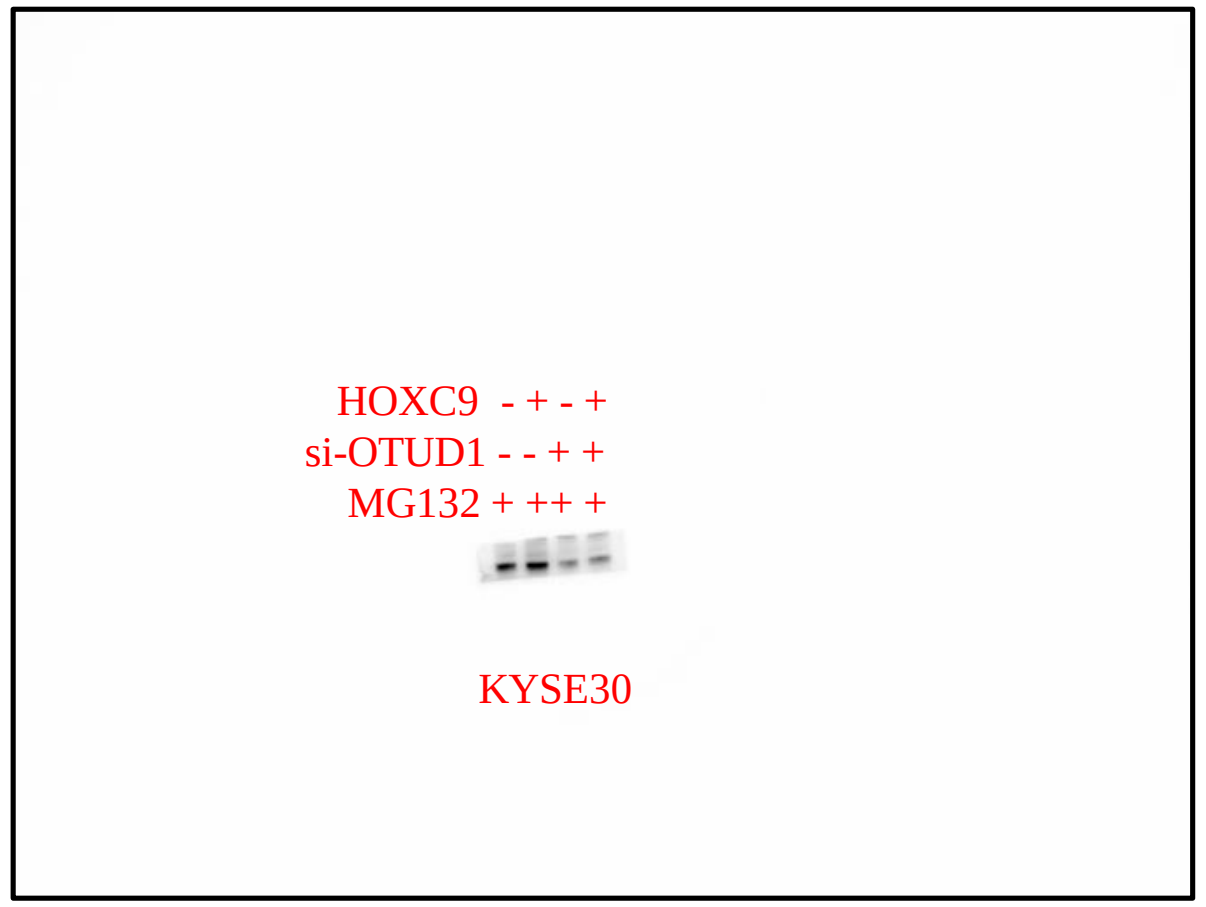

Fig. 6B WCL-FABP5

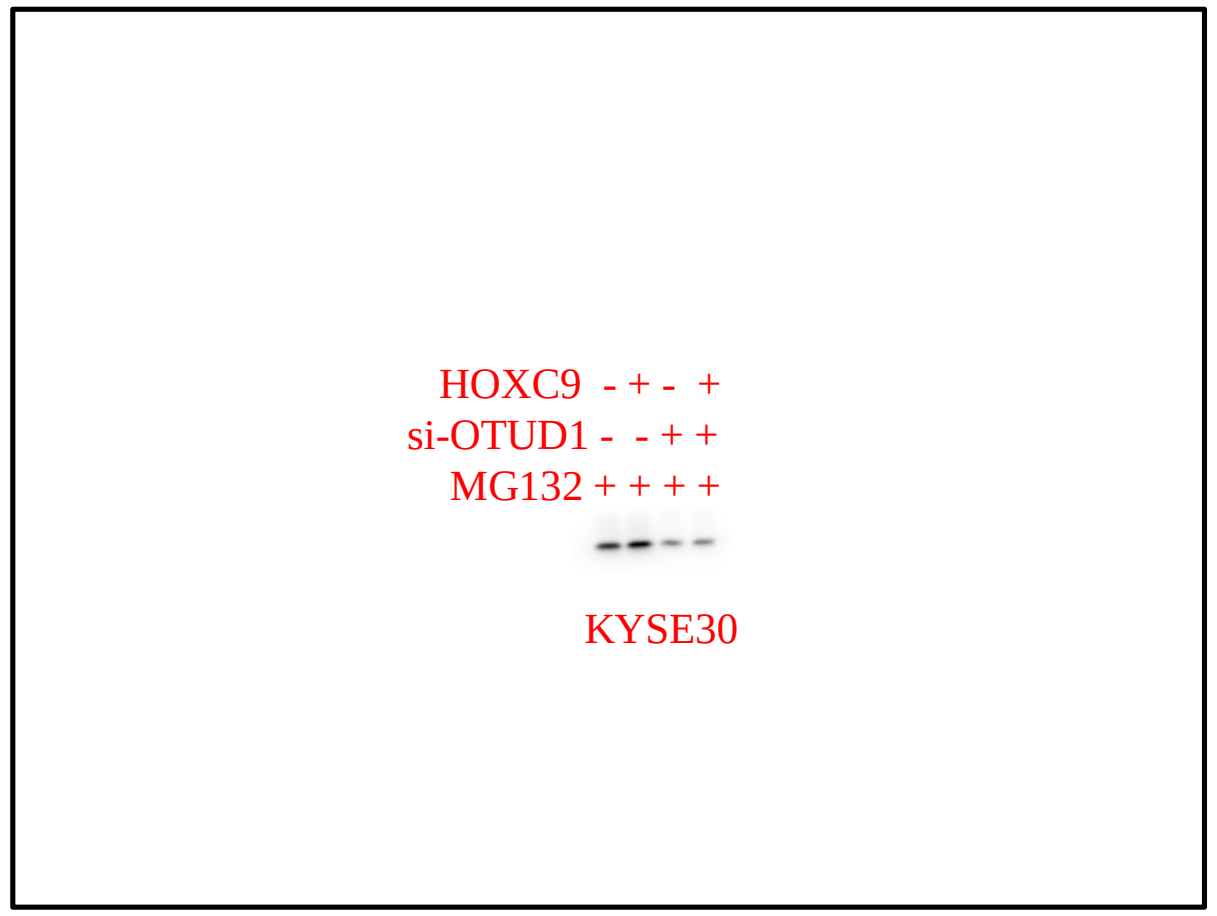

Fig. 6B WCL-UB

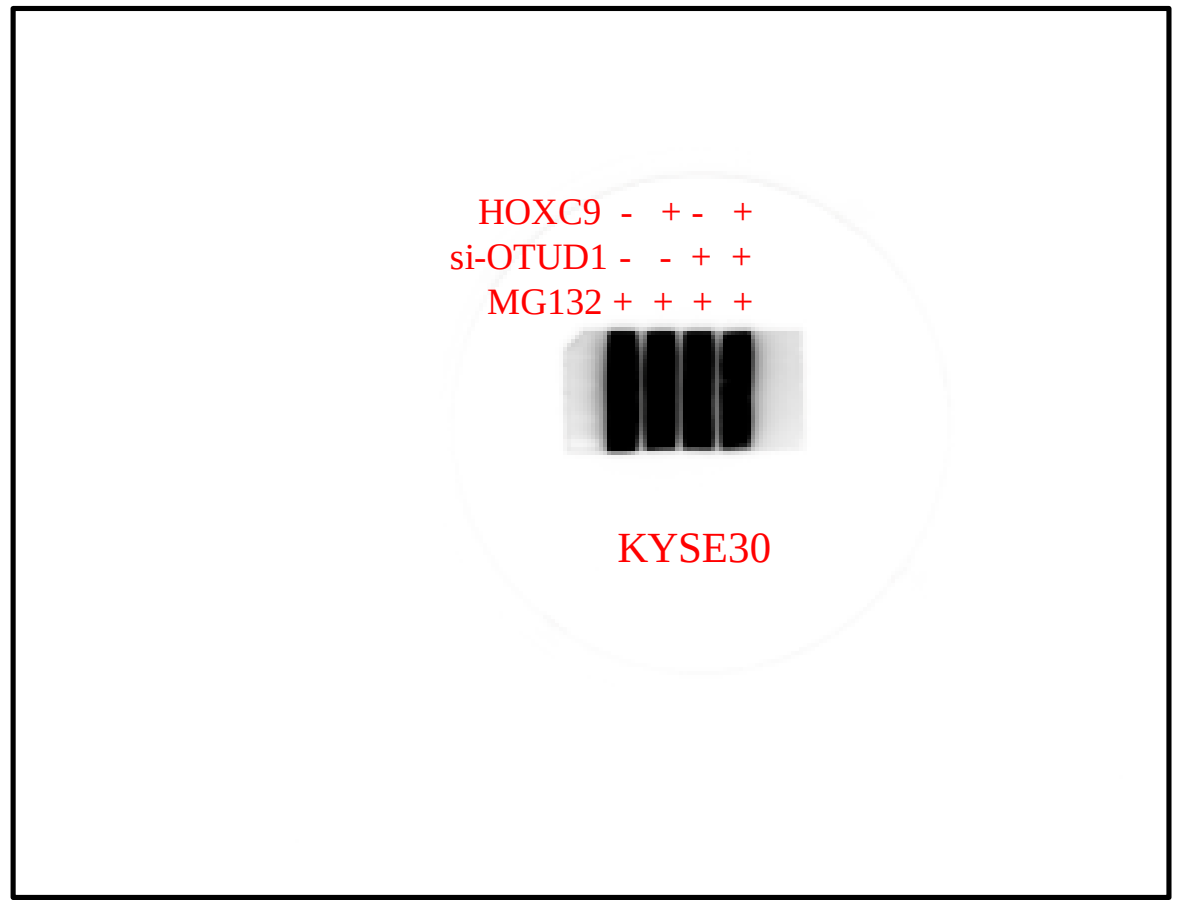

Fig. 6B IP-FABP5

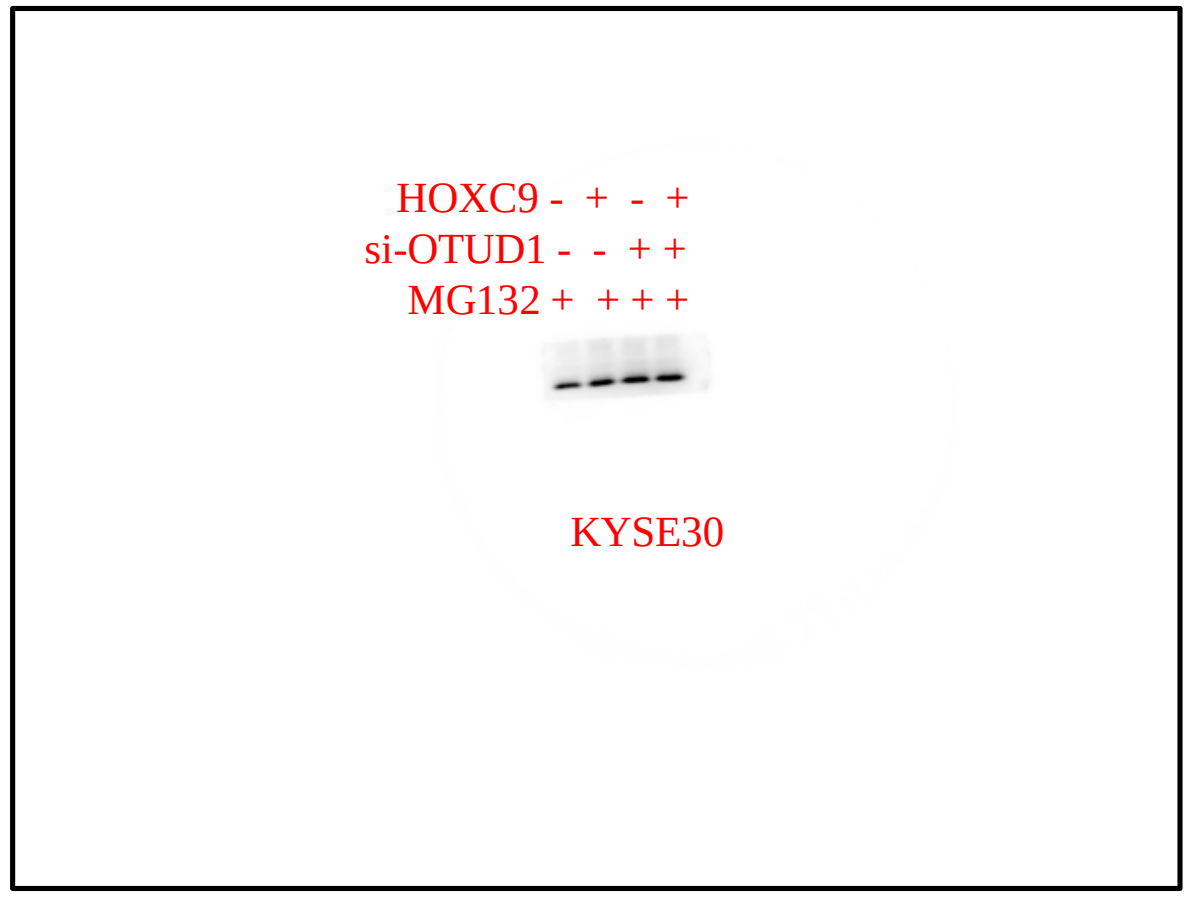

Fig. 6B IP-UB

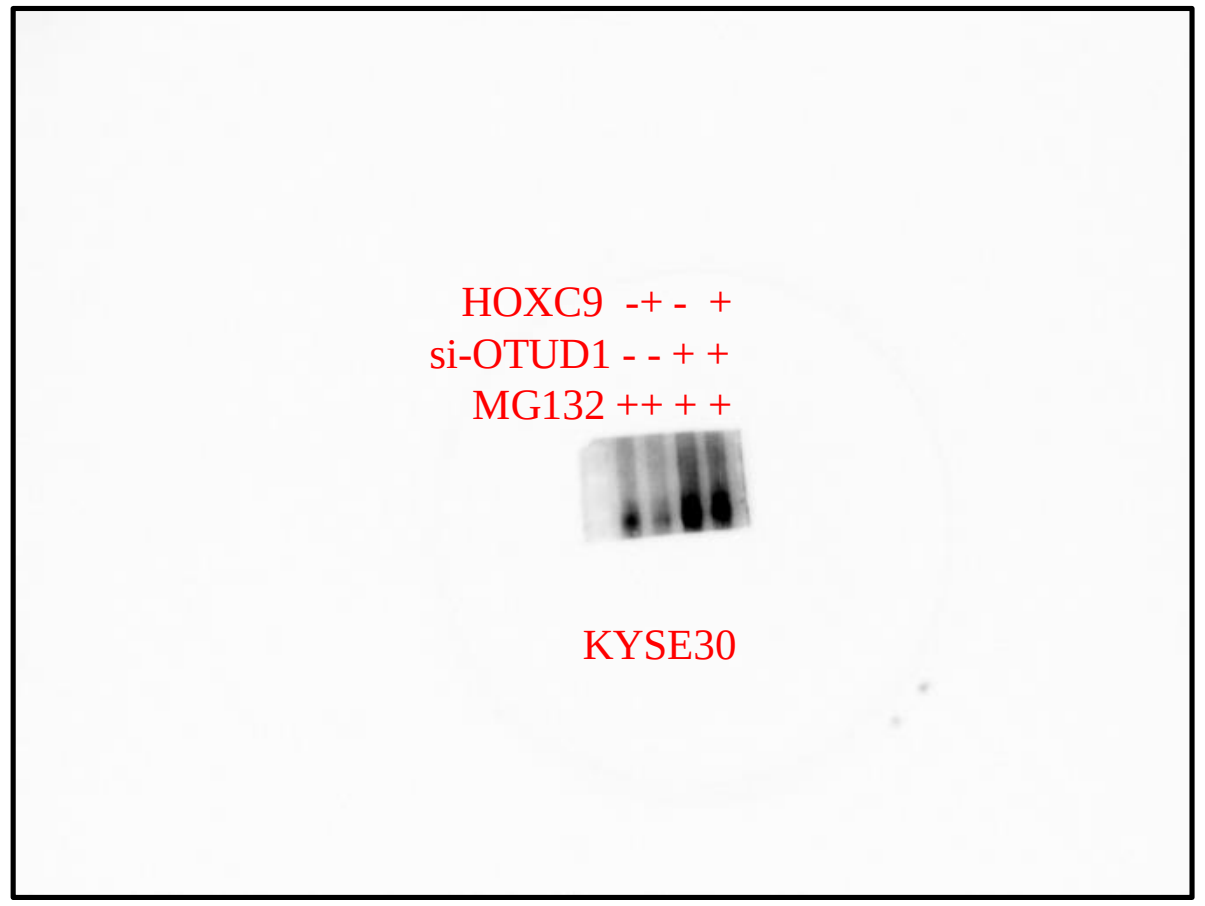

Fig. 6B WCL-β-actin

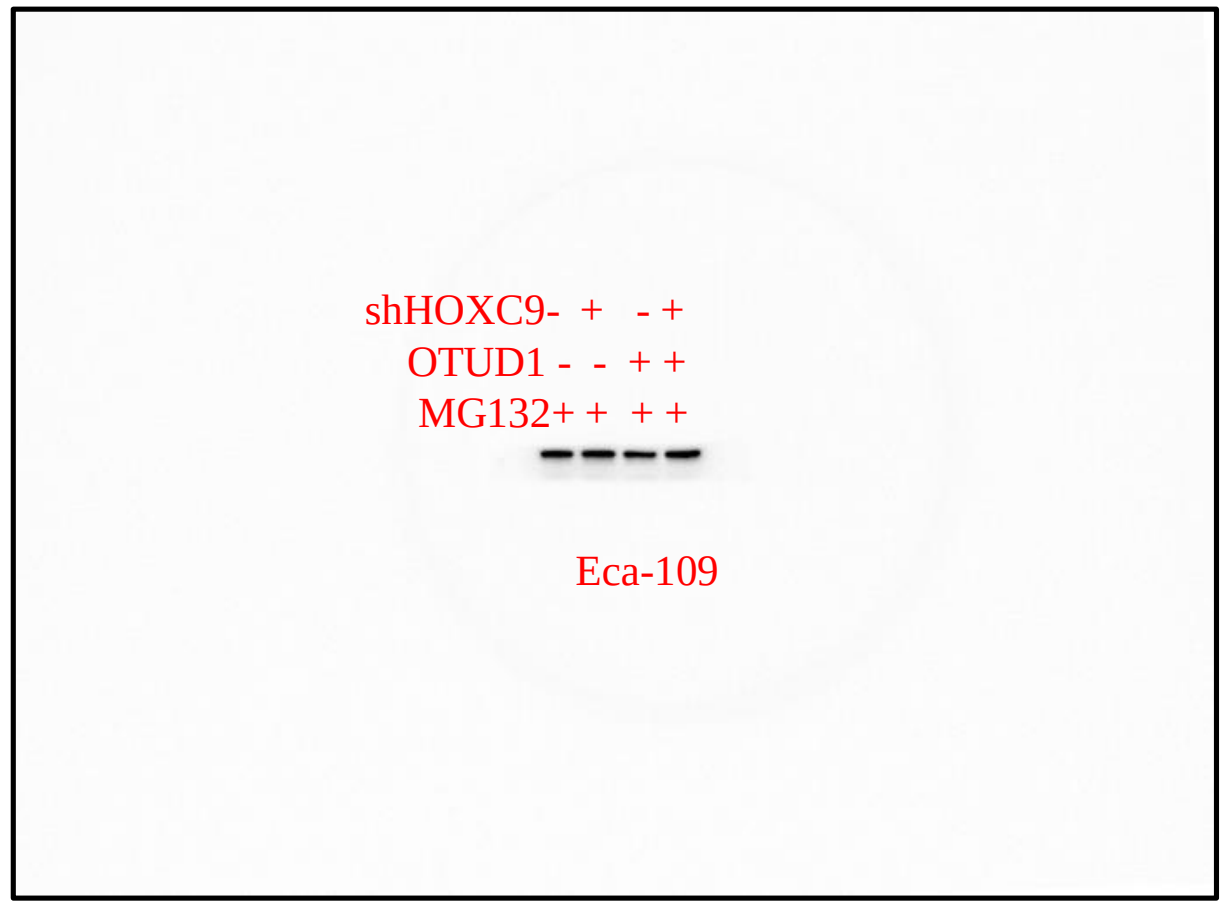

Fig. 6B WCL-HOXC9

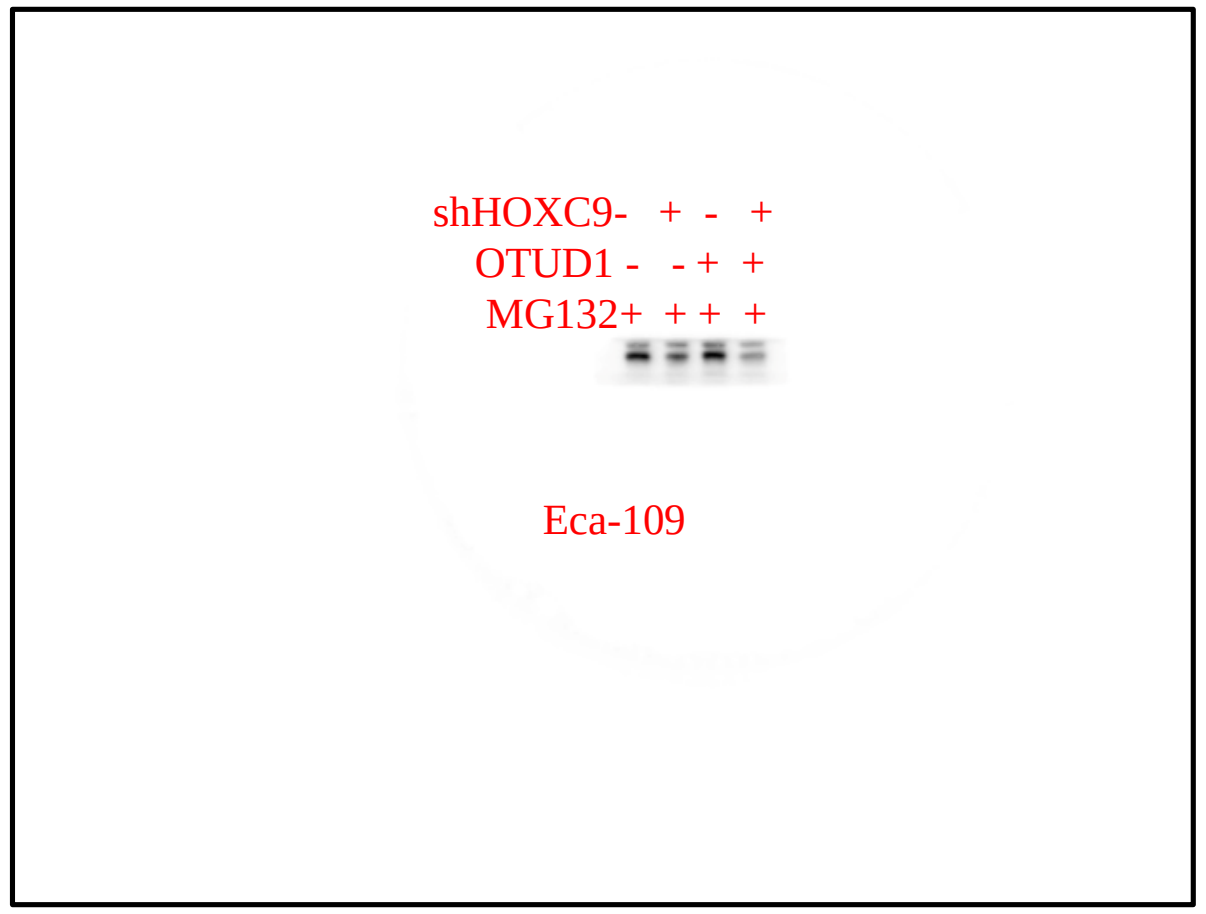

Fig. 6B WCL-OTUD1

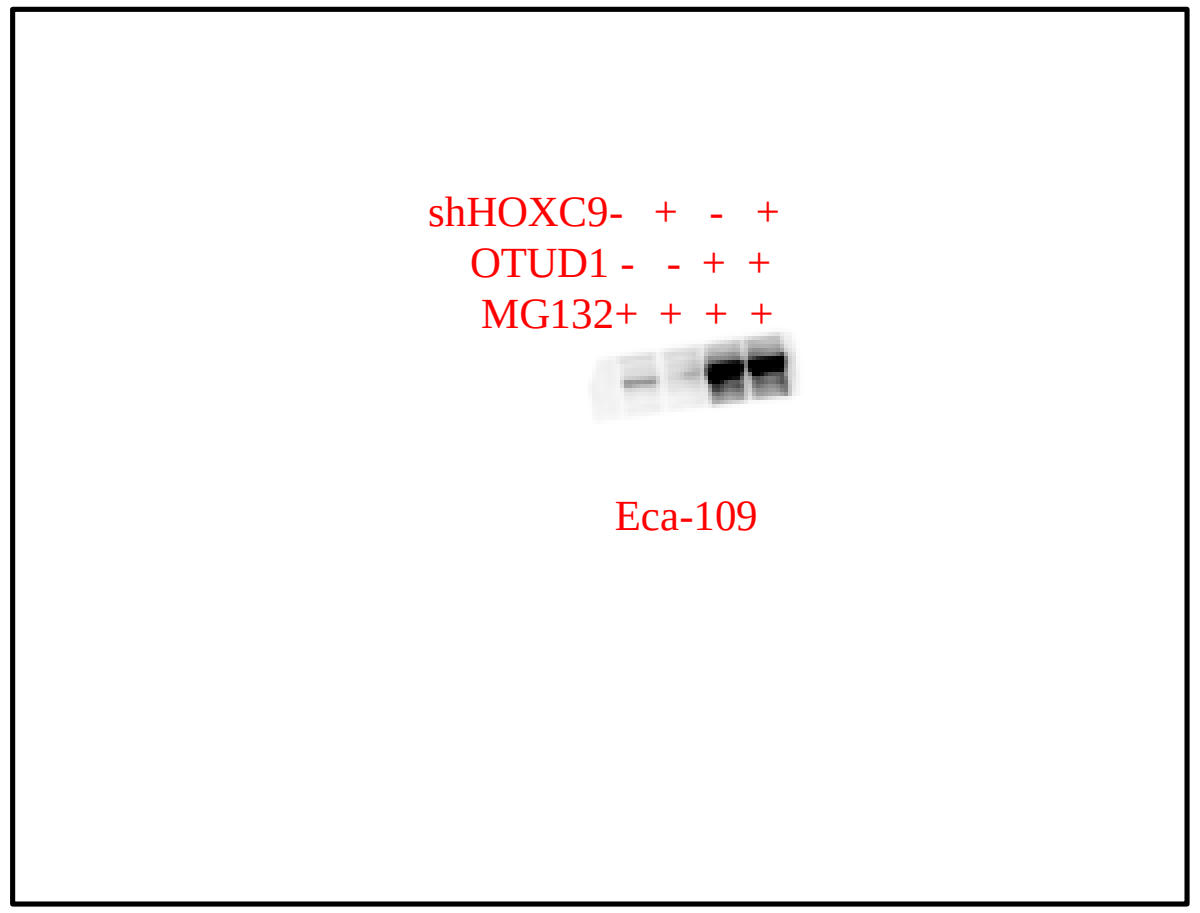

Fig. 6B WCL-FABP5

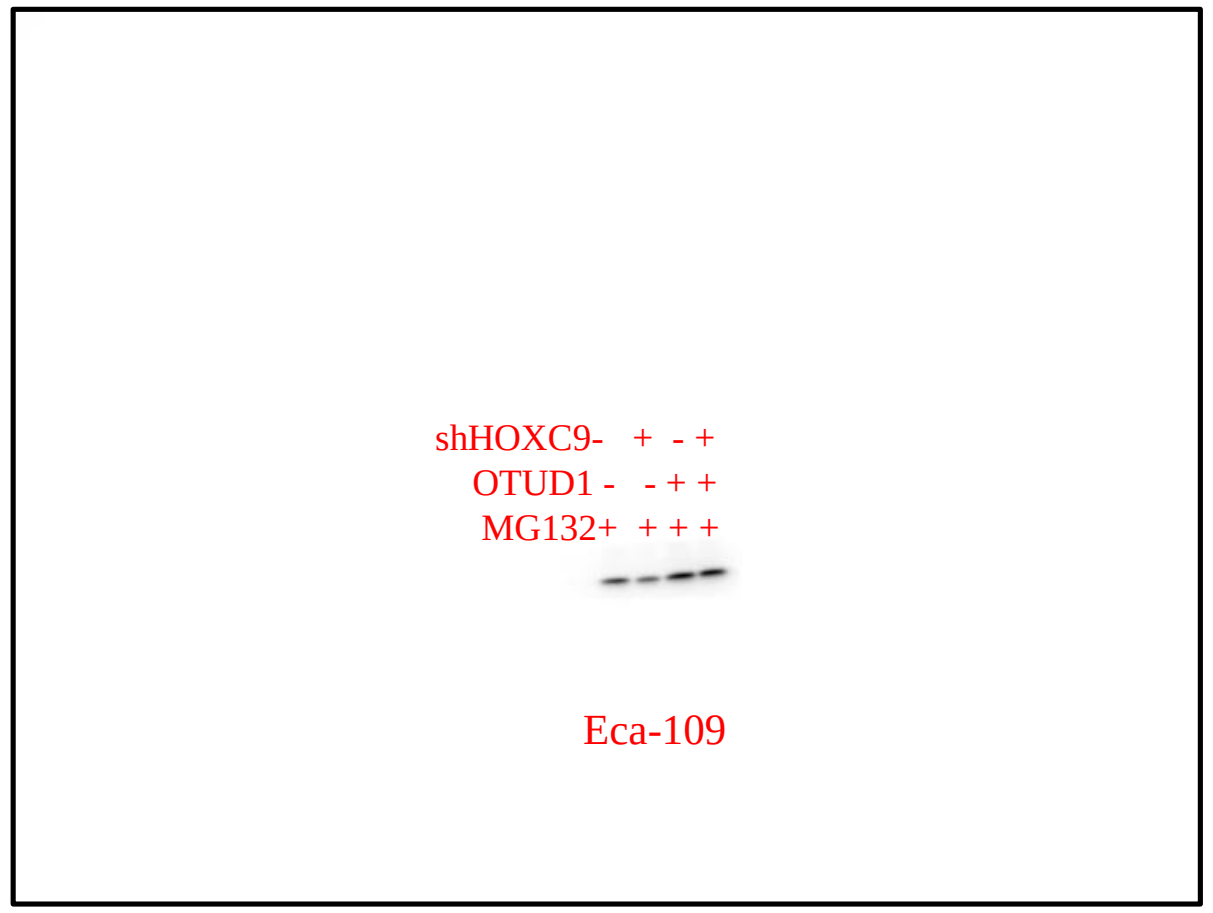

Fig. 6B WCL-UB

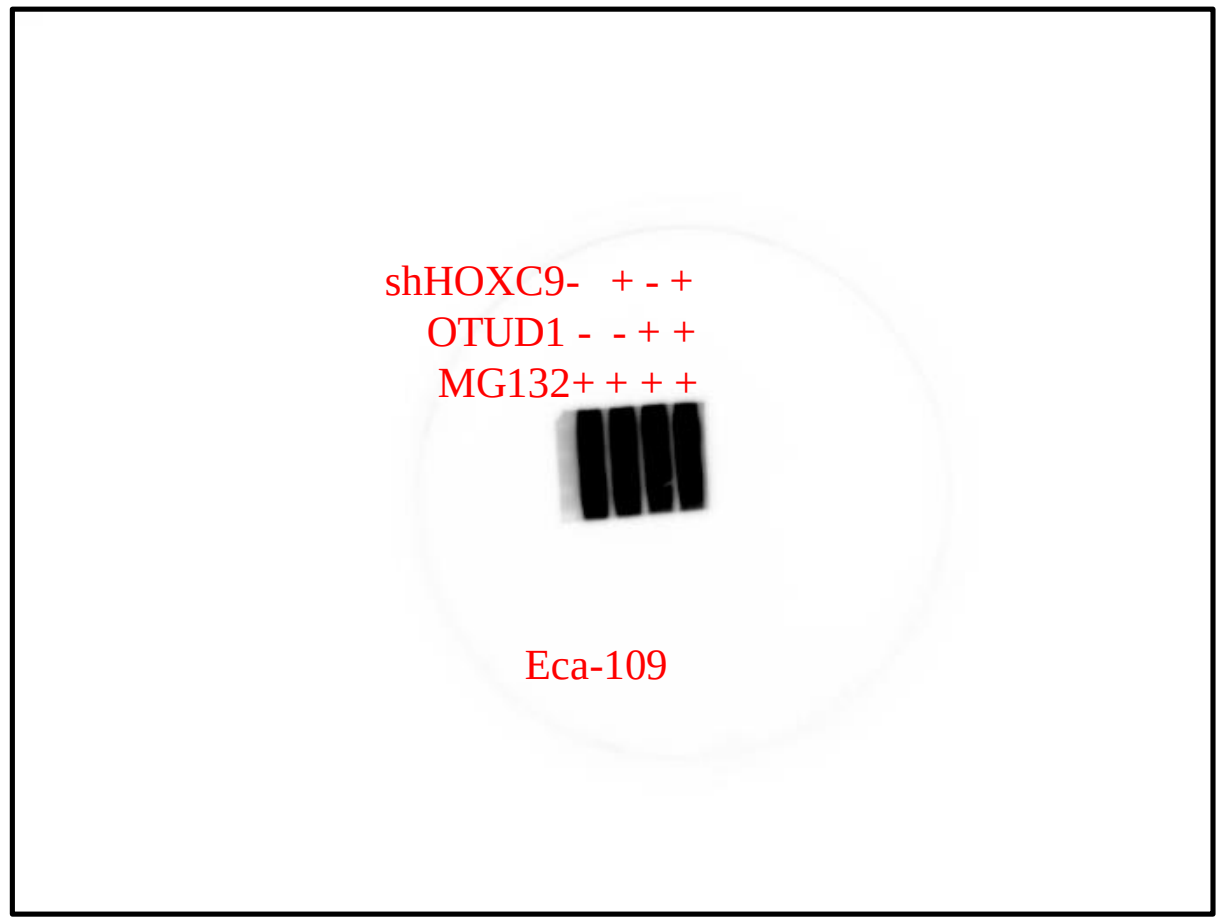

Fig. 6B IP-FABP5

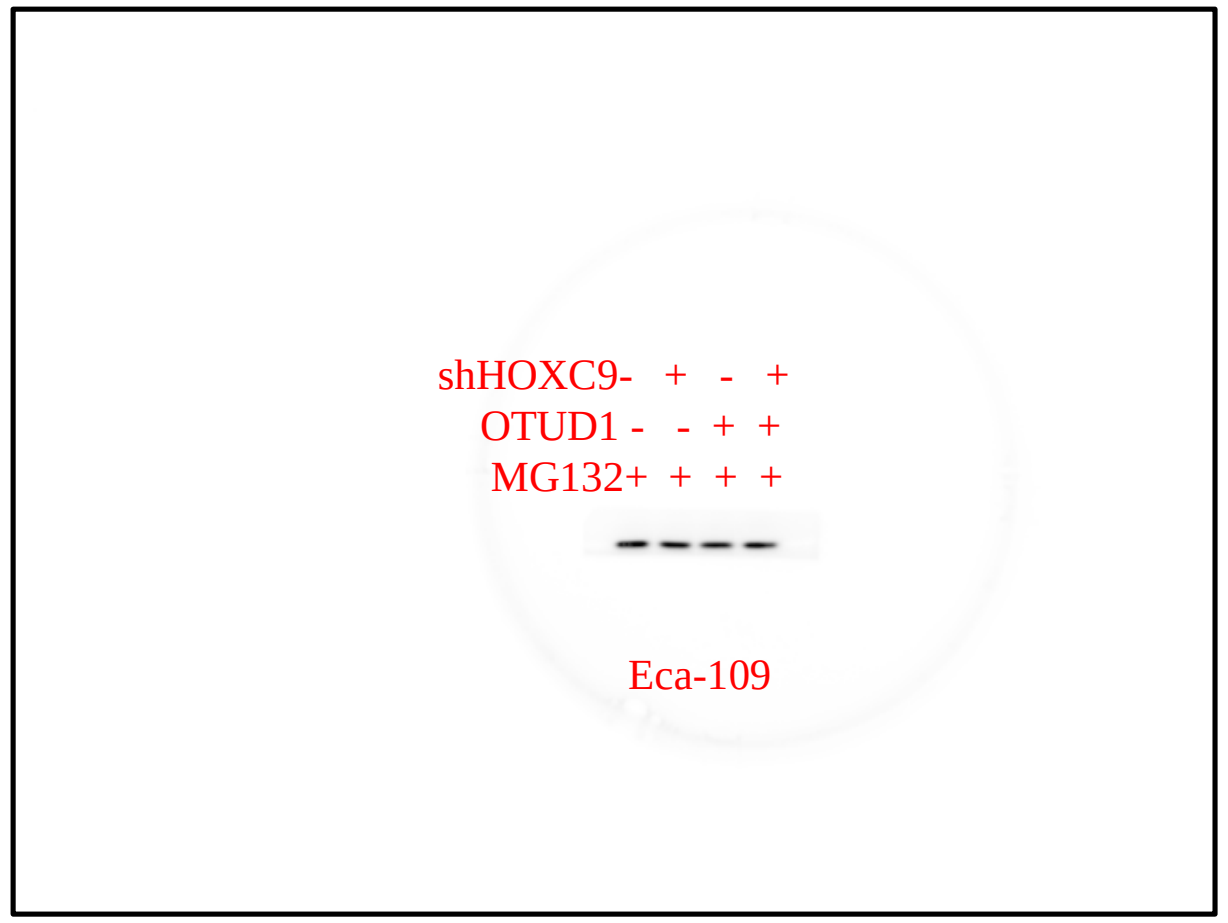

Fig. 6B IP-UB

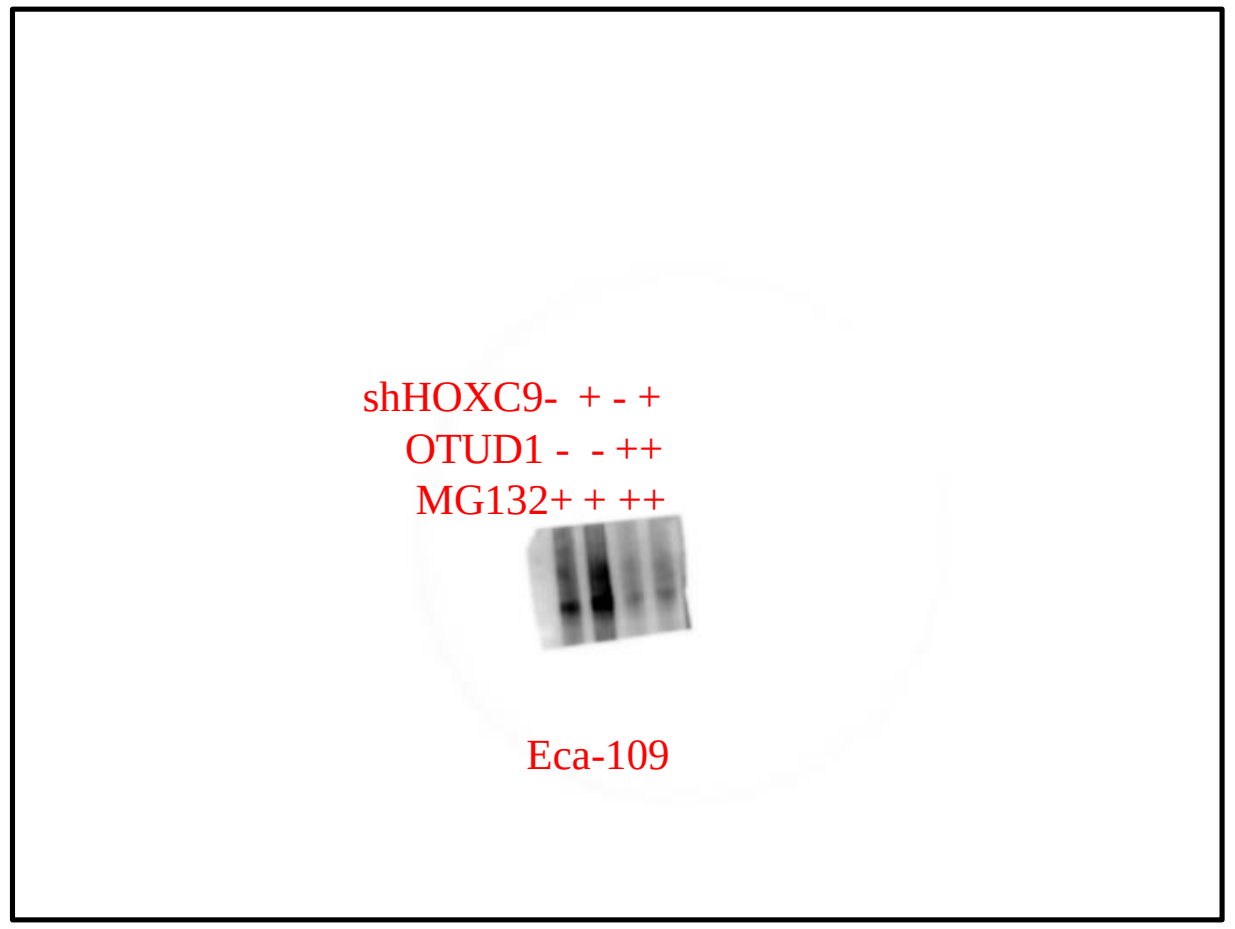

Fig. 7A β-actin

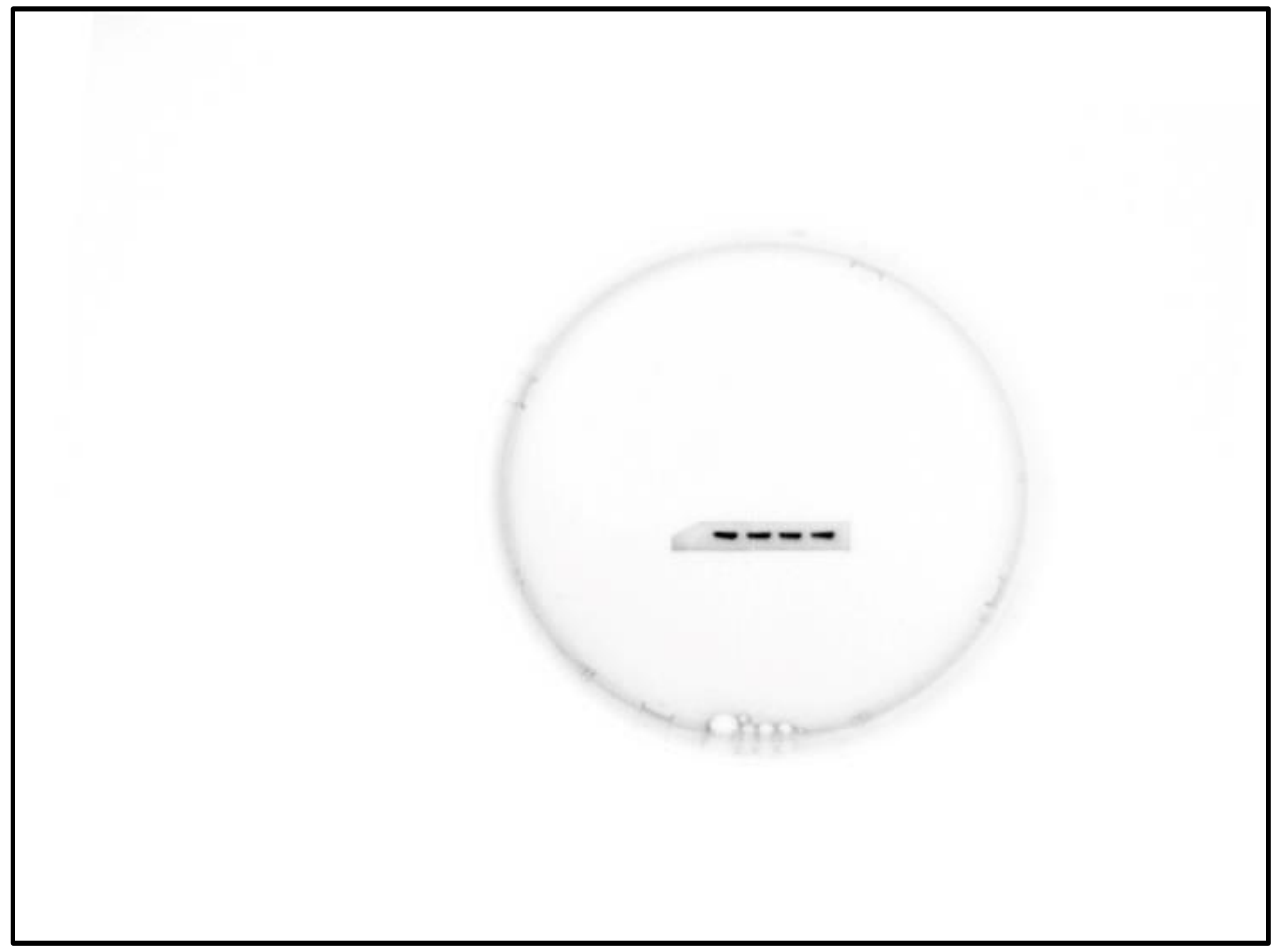

Fig. 7A HOXC9

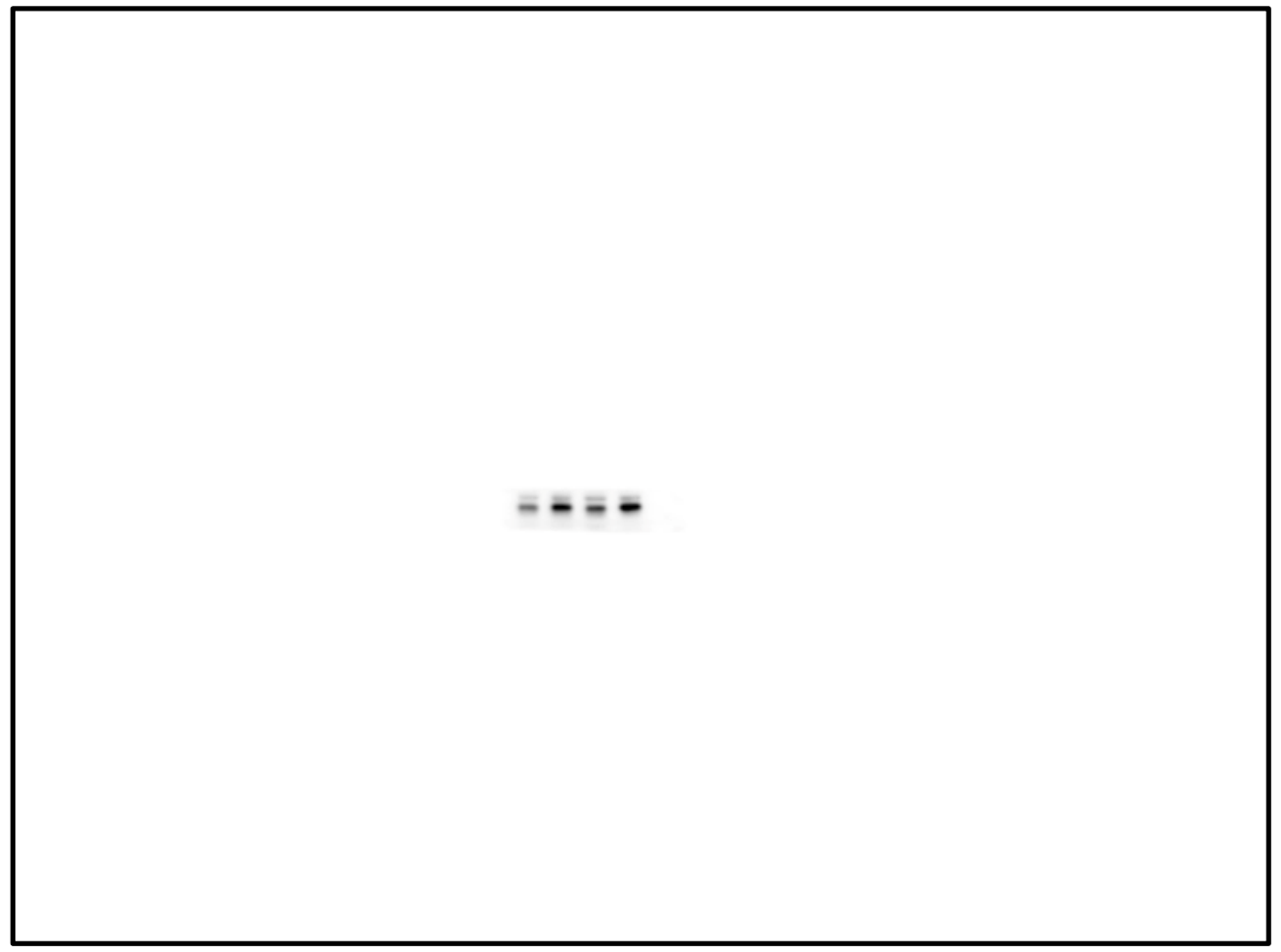

Fig. 7A FABP5

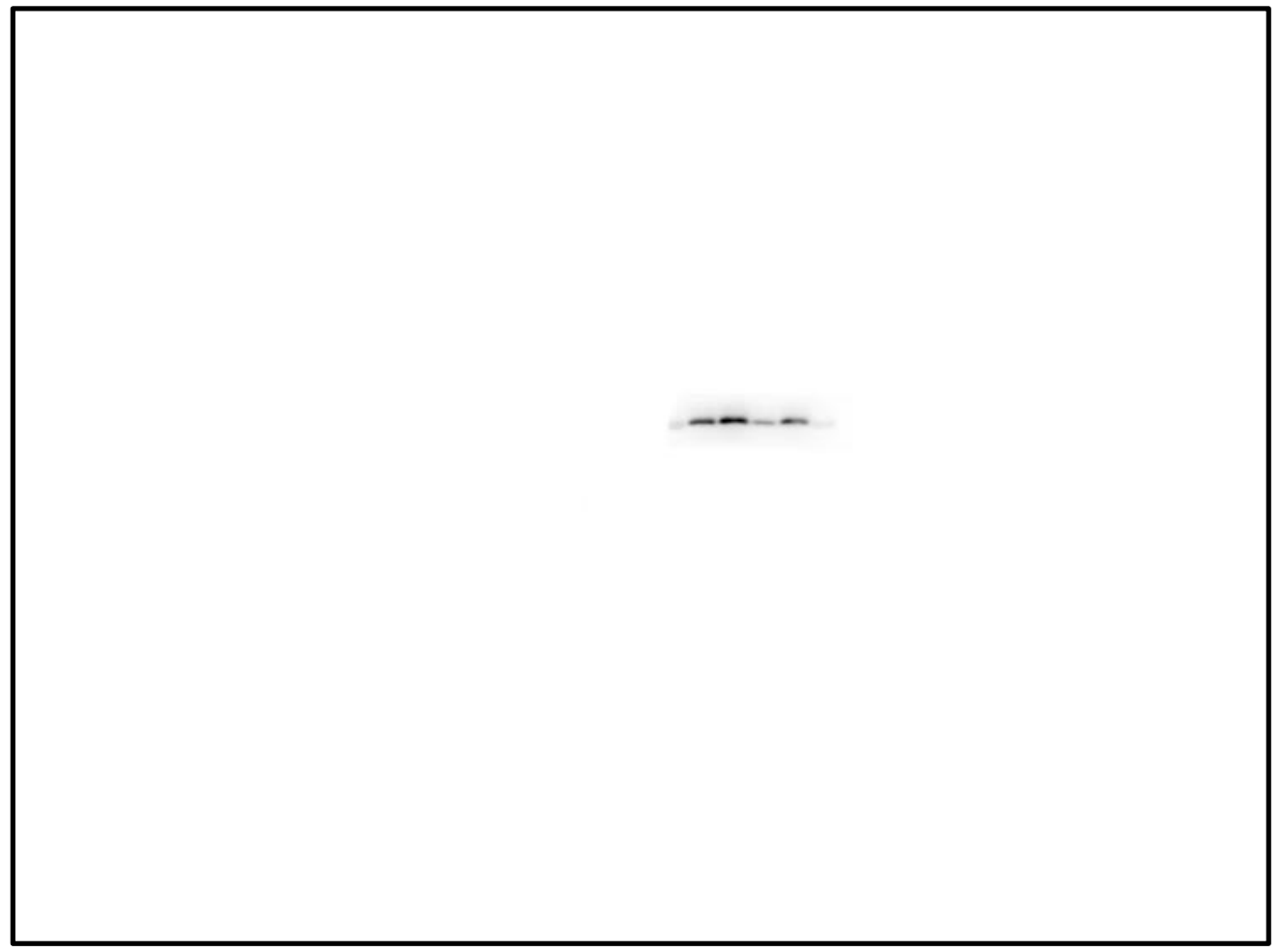

Fig. 7B β-actin

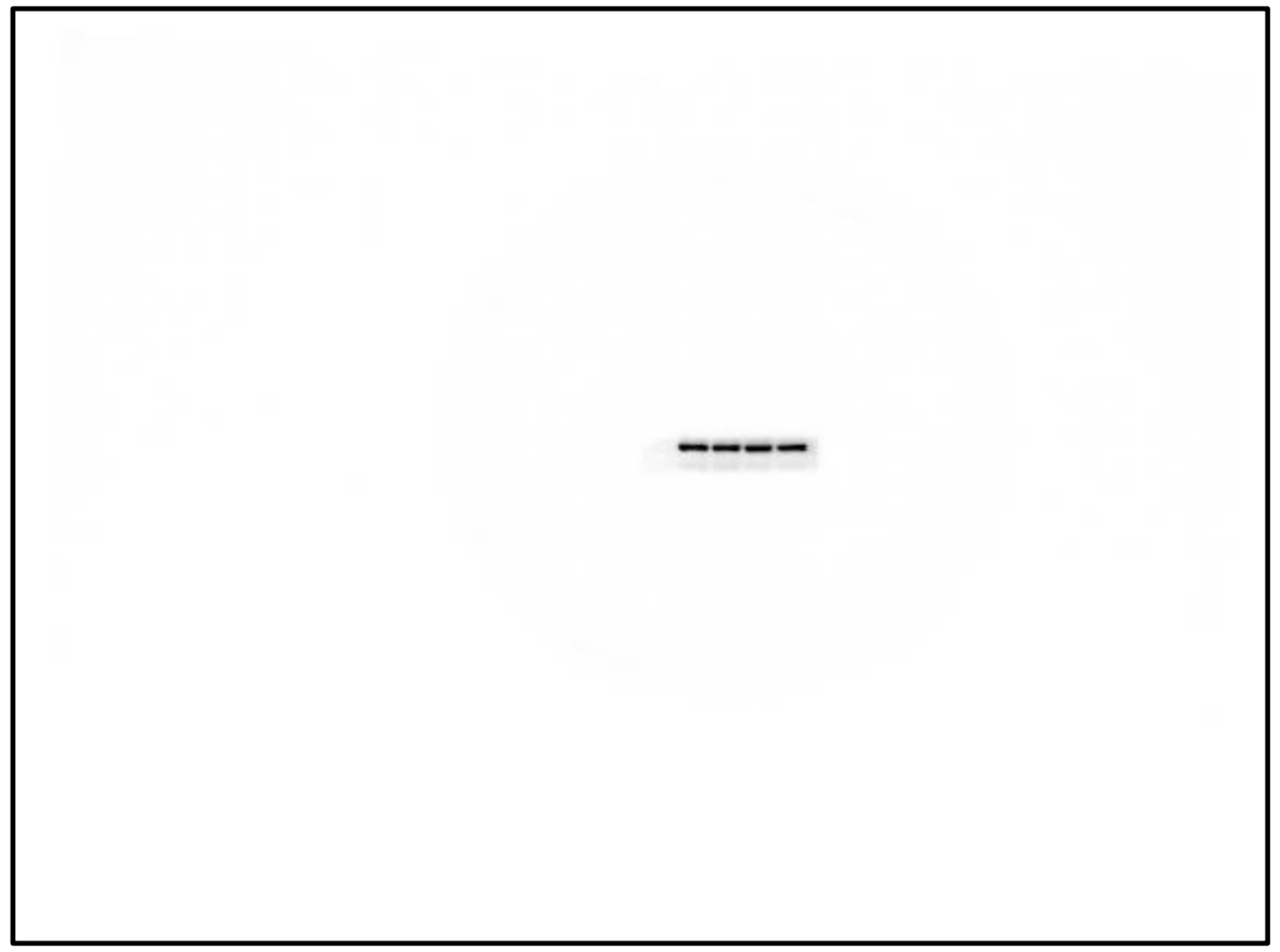

Fig. 7B OTUD1

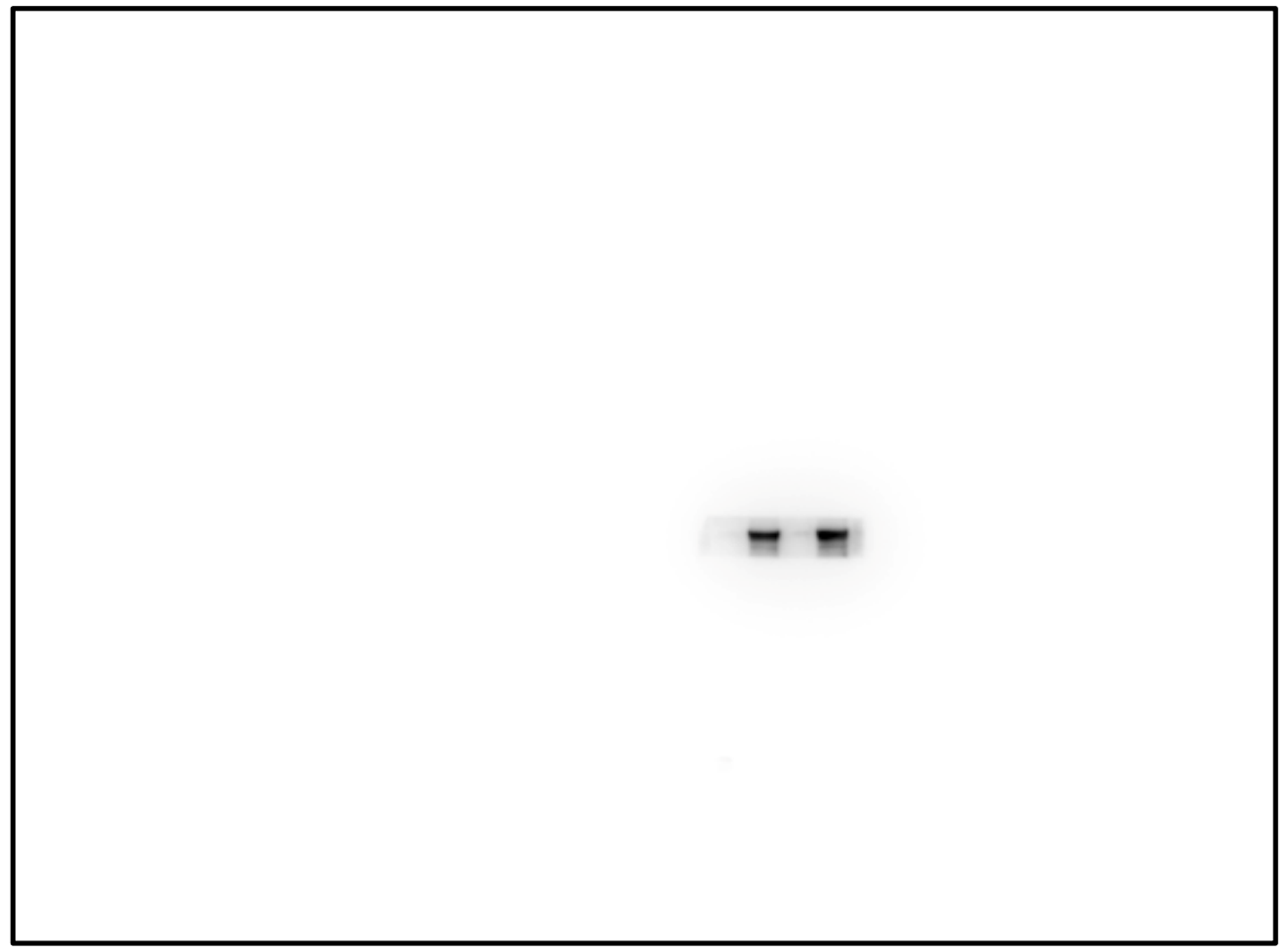

Fig. 7B FABP5

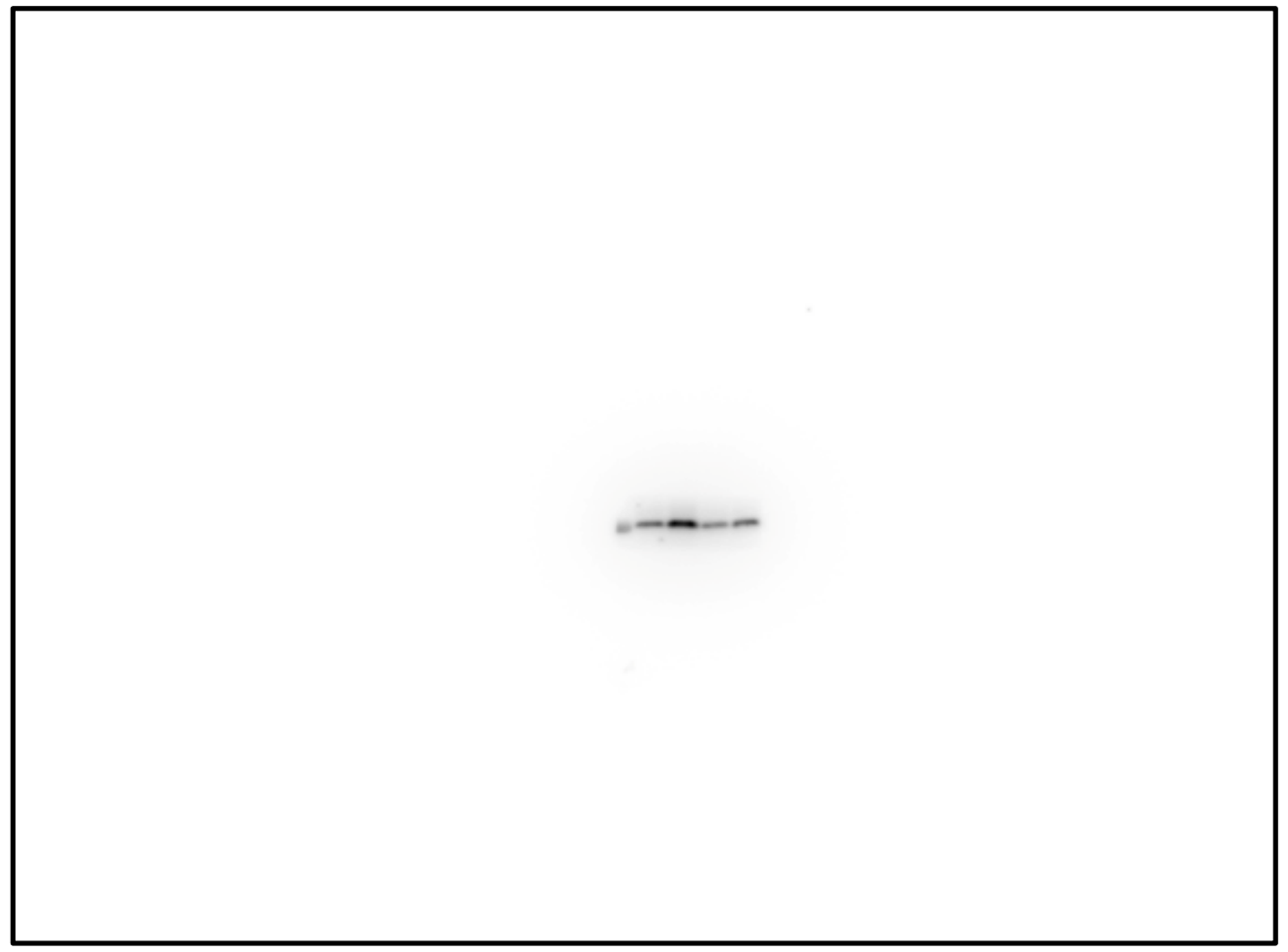

Fig. S1B -β-tubulin

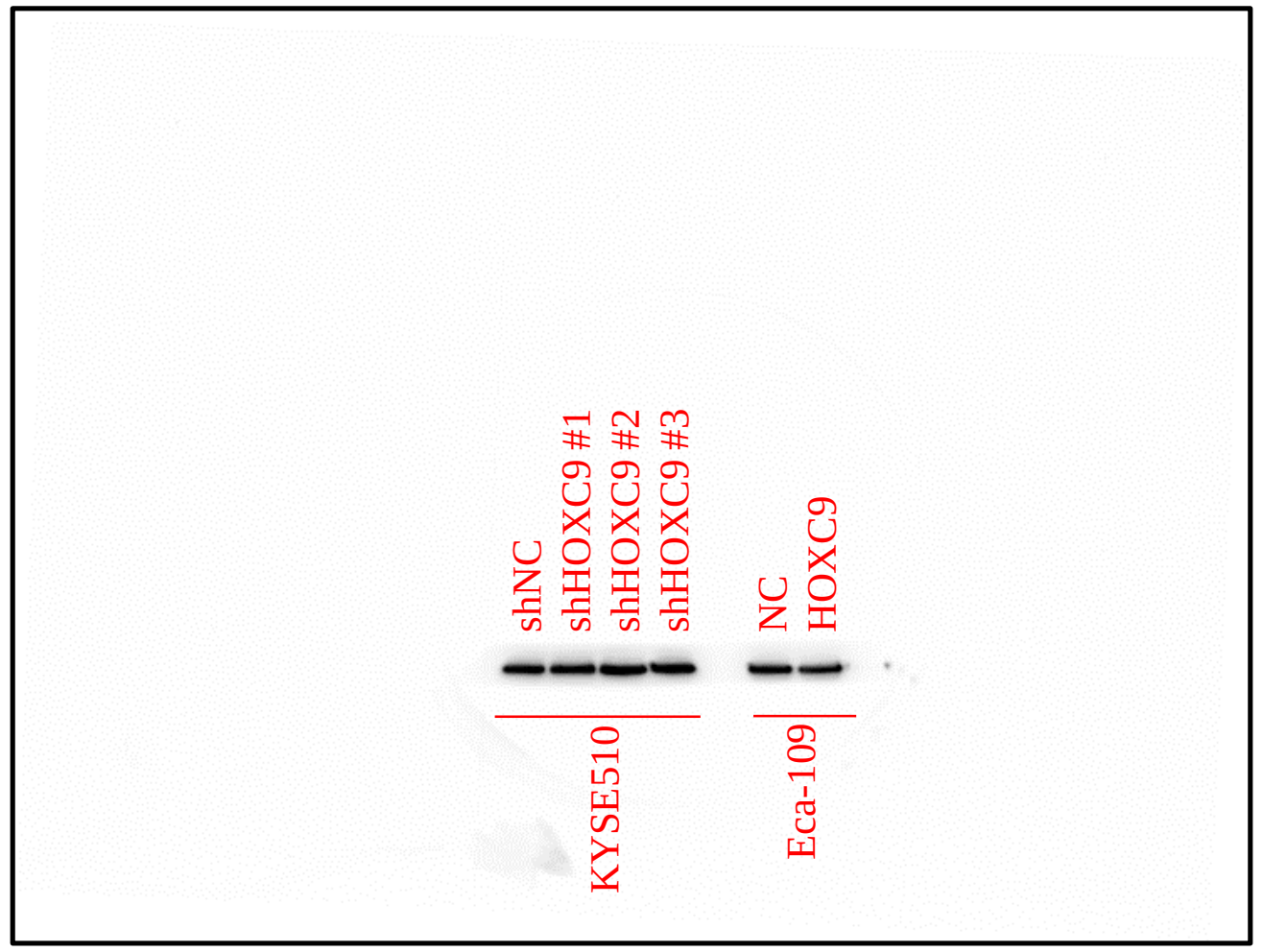

Fig. S1B HOXC9-low exposure

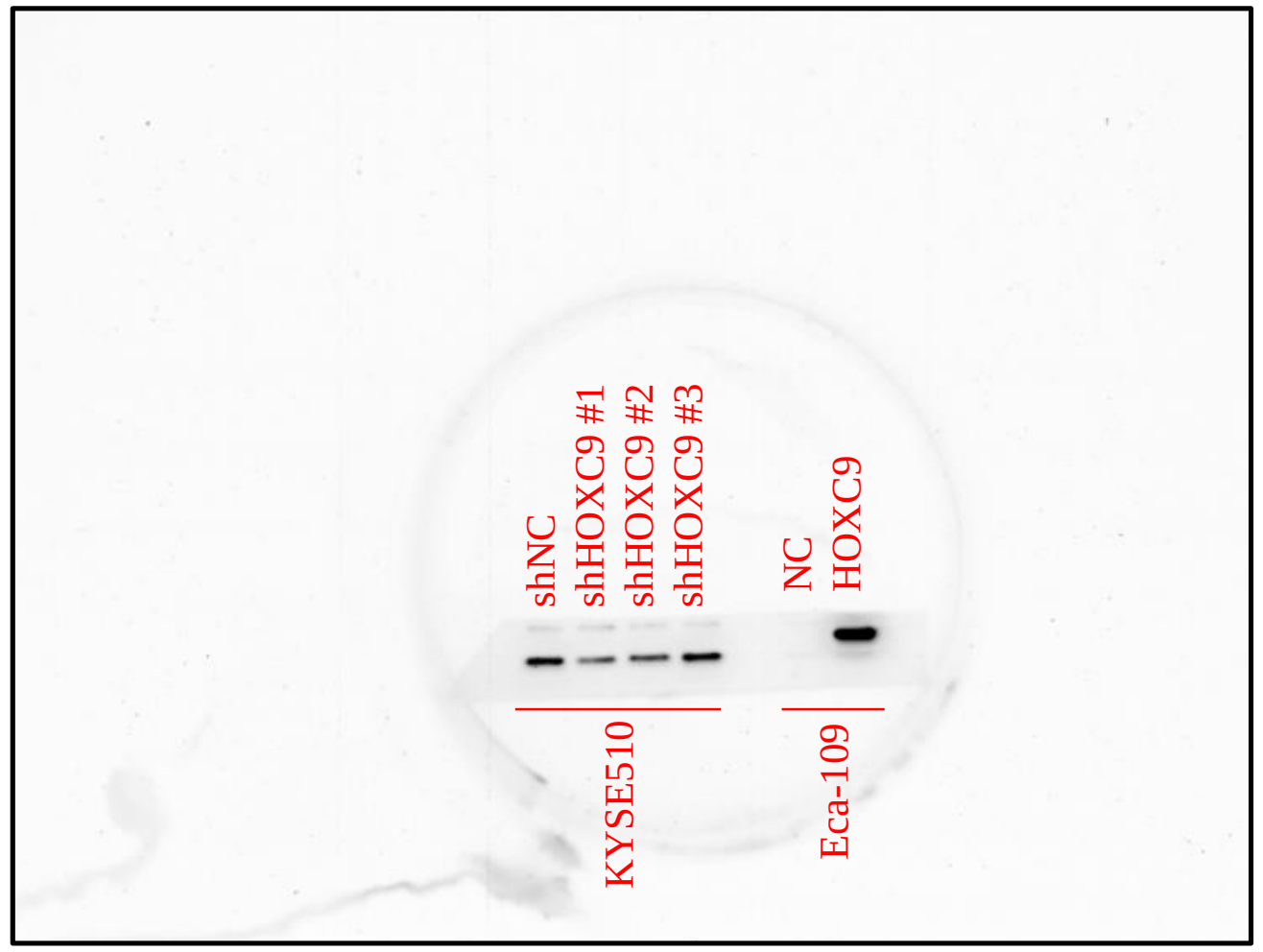

Fig. S1B HOXC9-high exposure

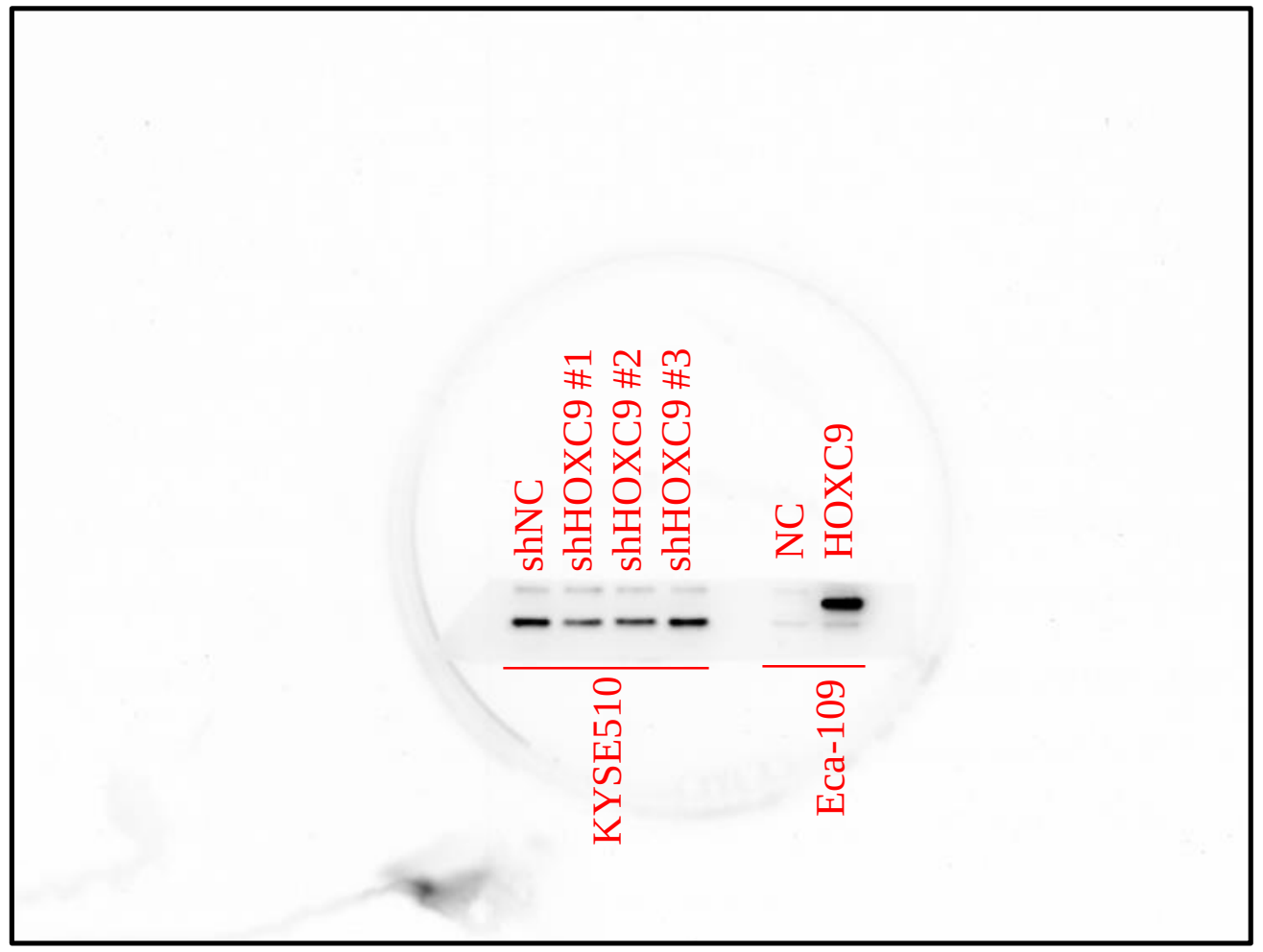

Fig. S3B β-actin

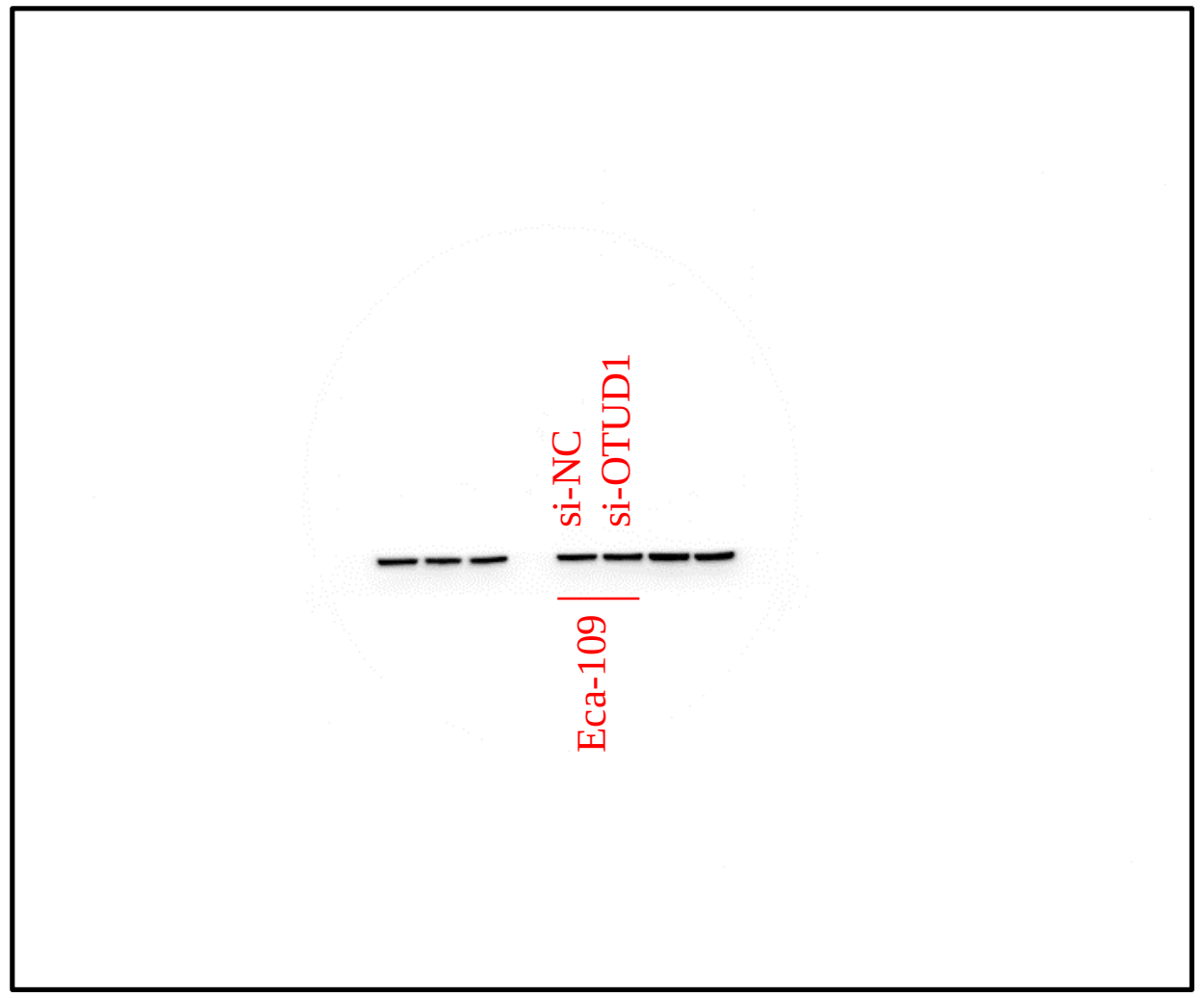

Fig. S3B OTUD1

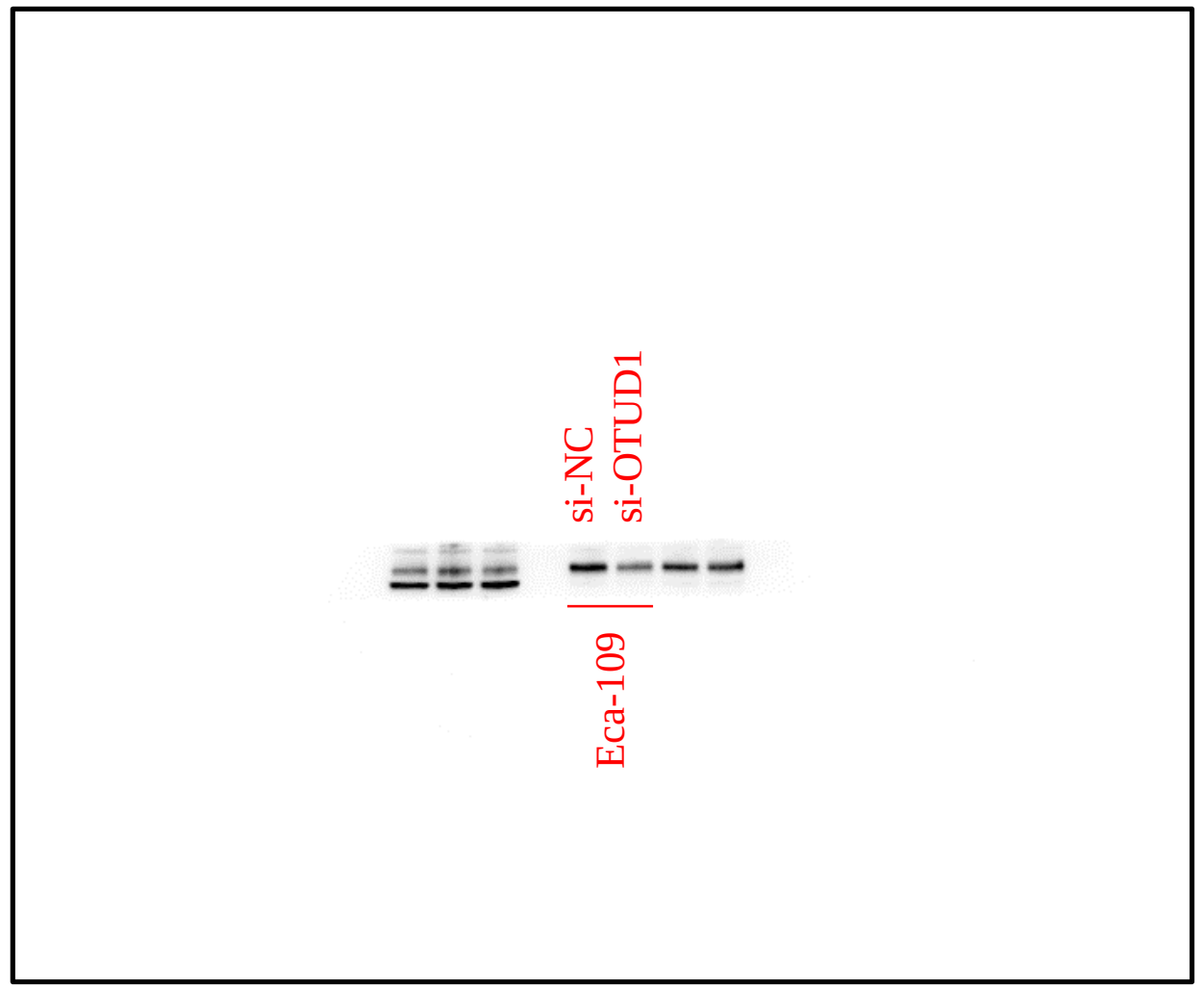

Fig. S3B β-actin

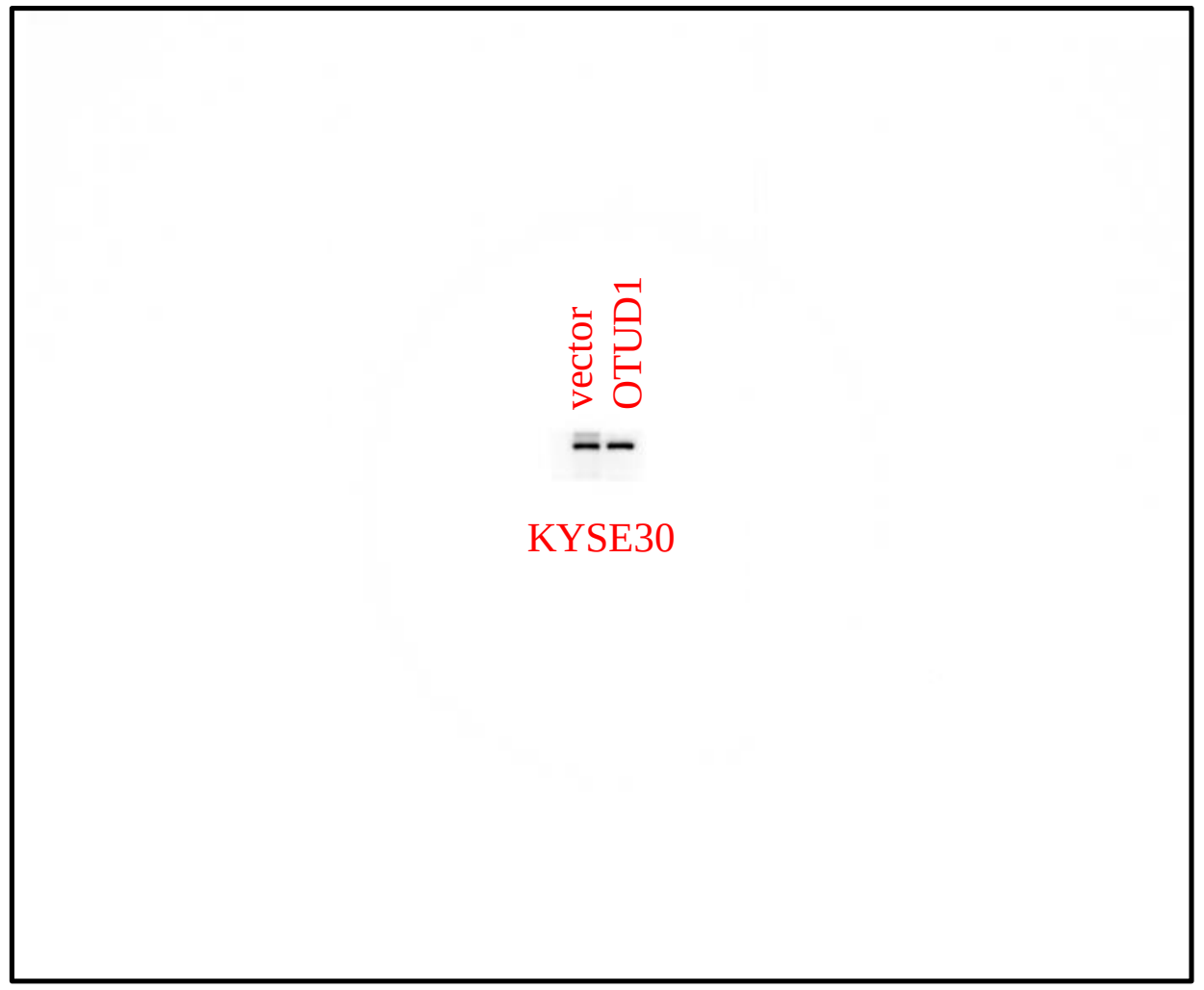

Fig. S3B OTUD1

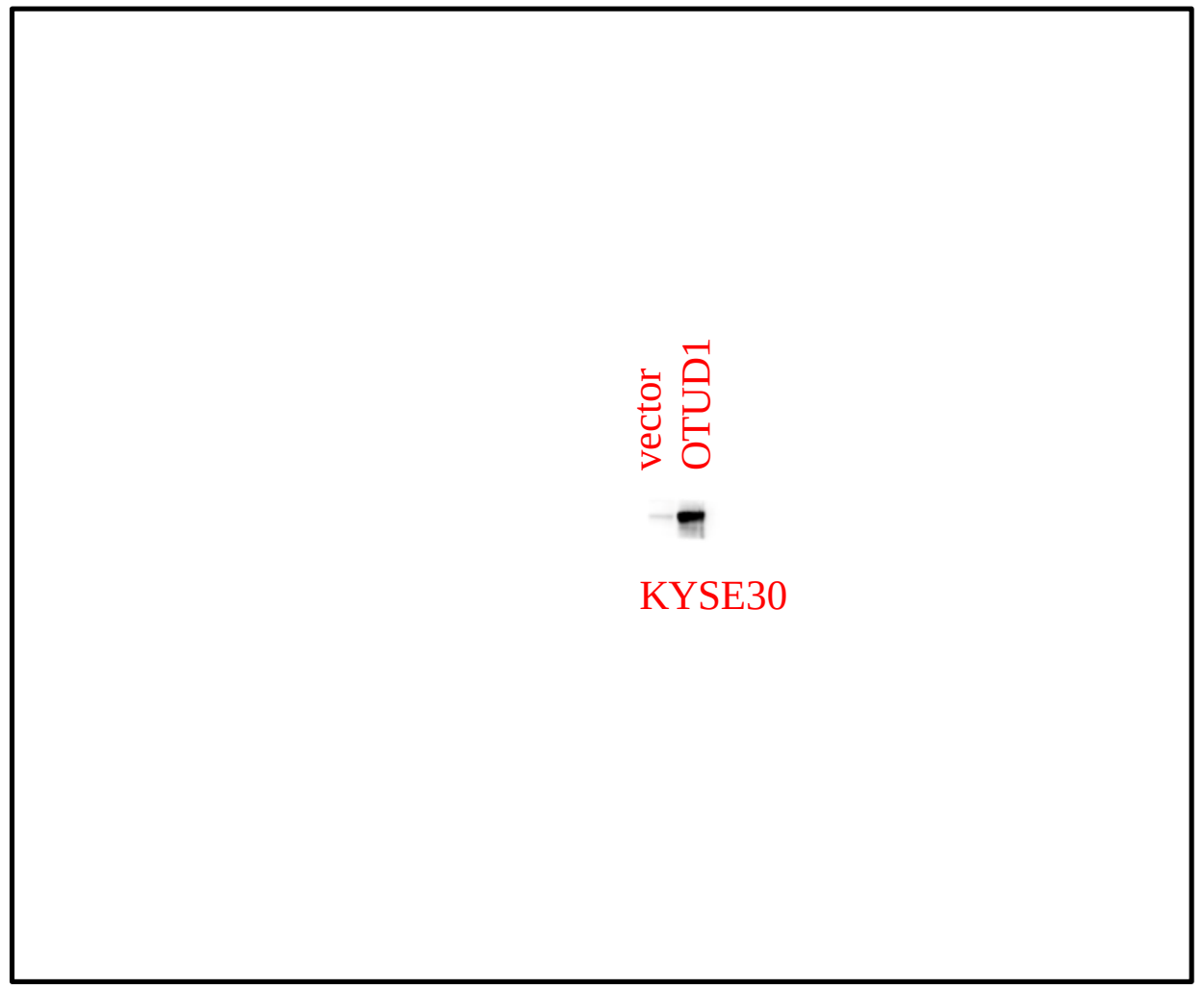

Fig. S5B β-actin

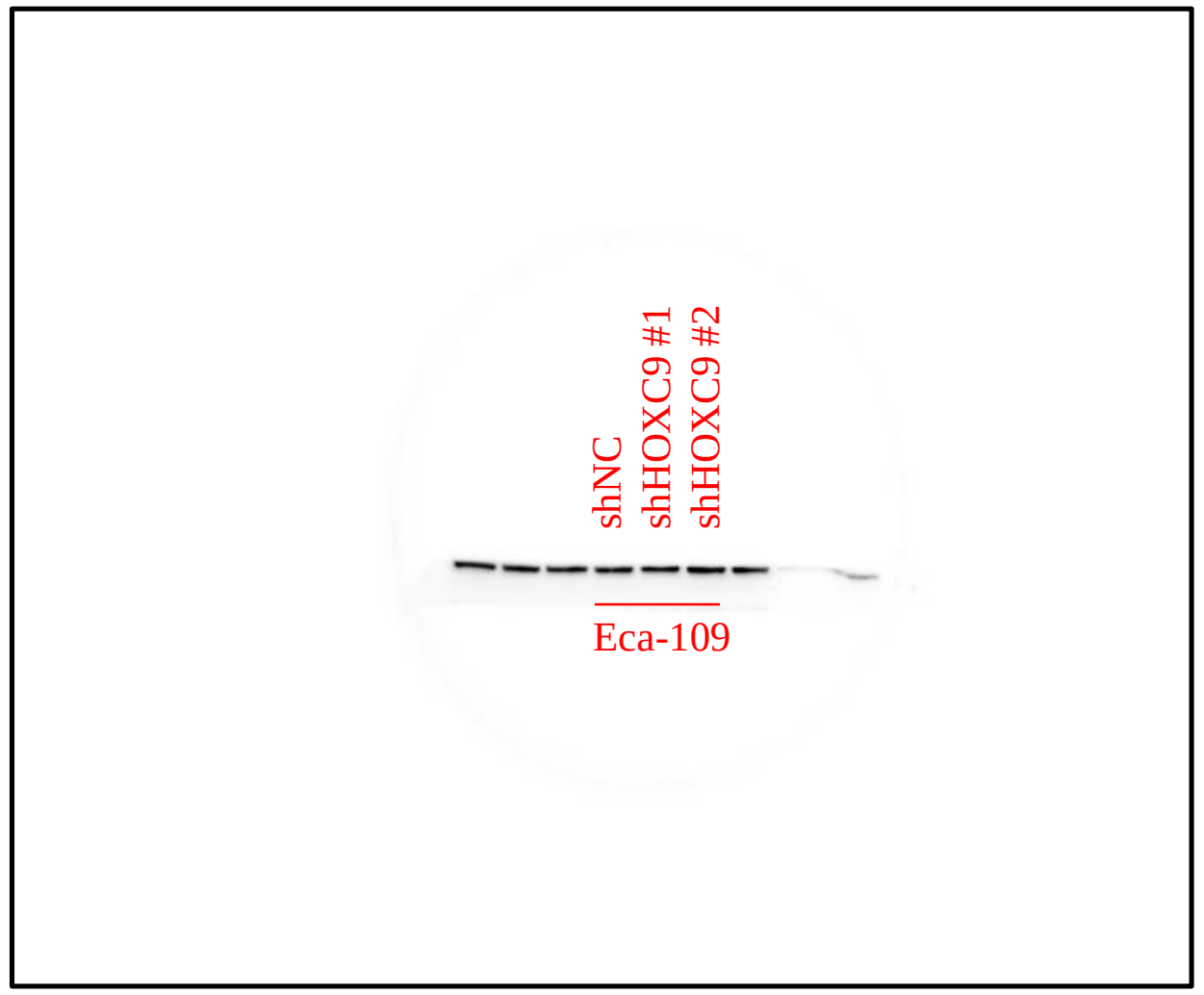

Fig. S5B HOXC9

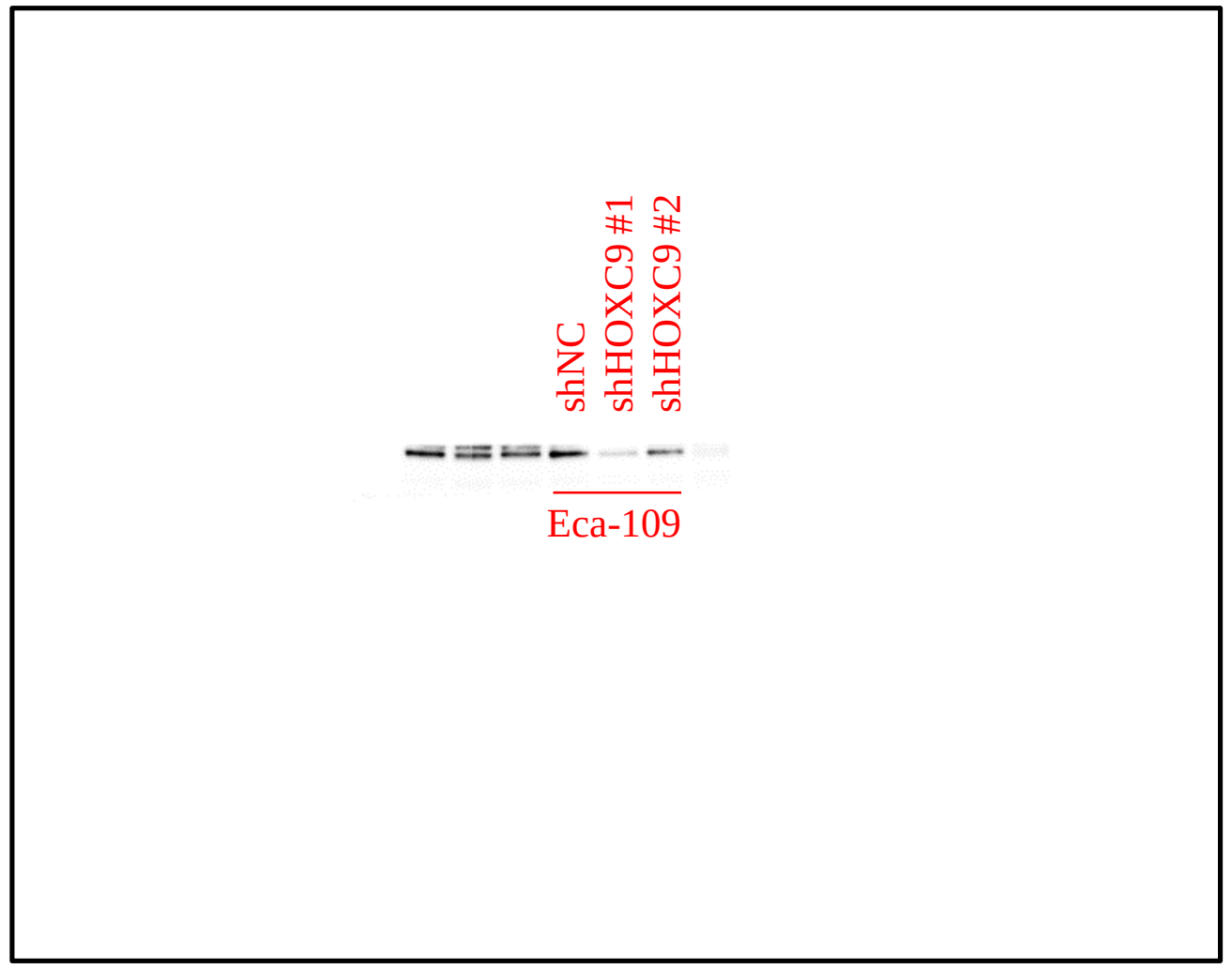

Fig. S5B FABP5

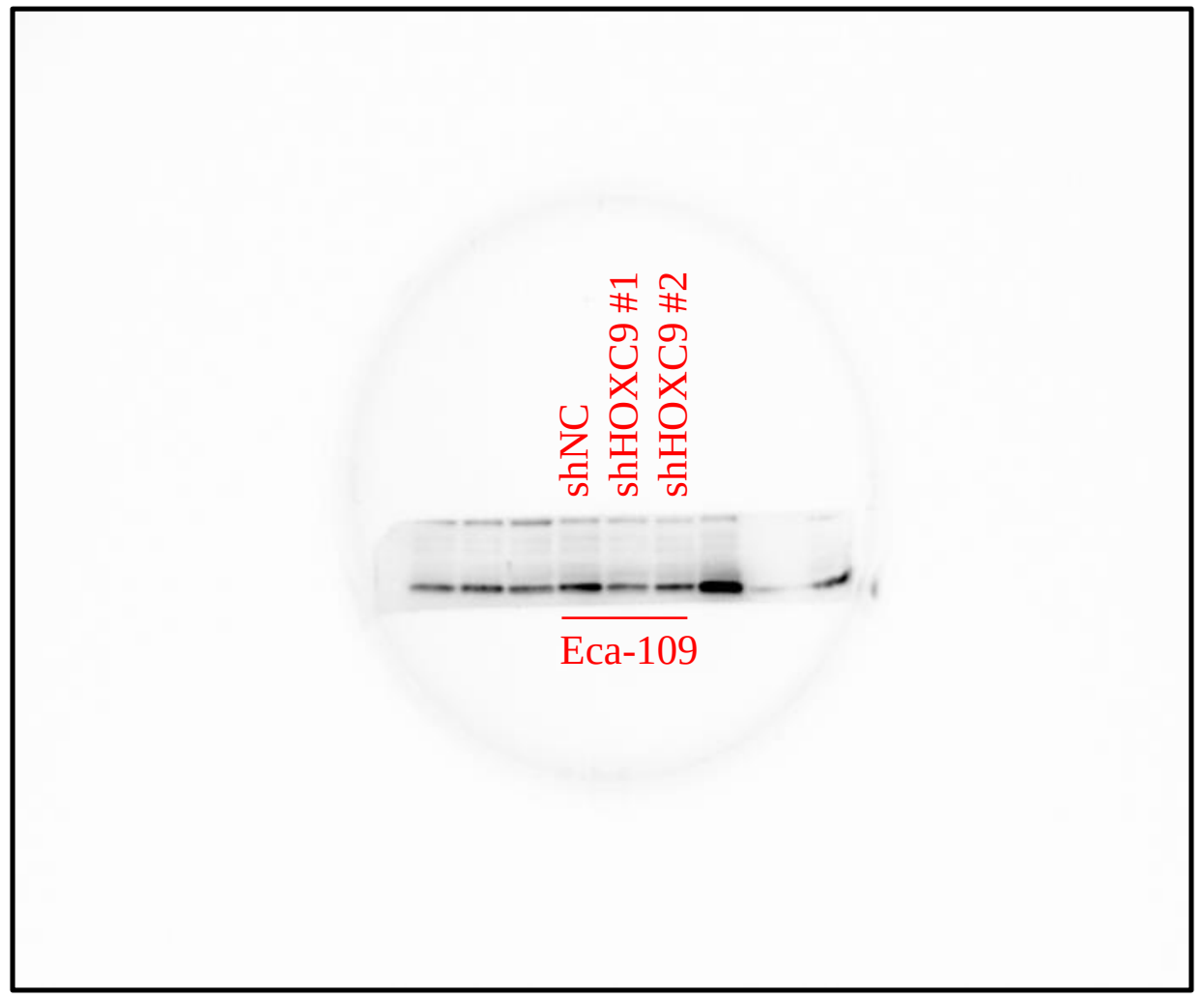

Fig. S5B β-actin

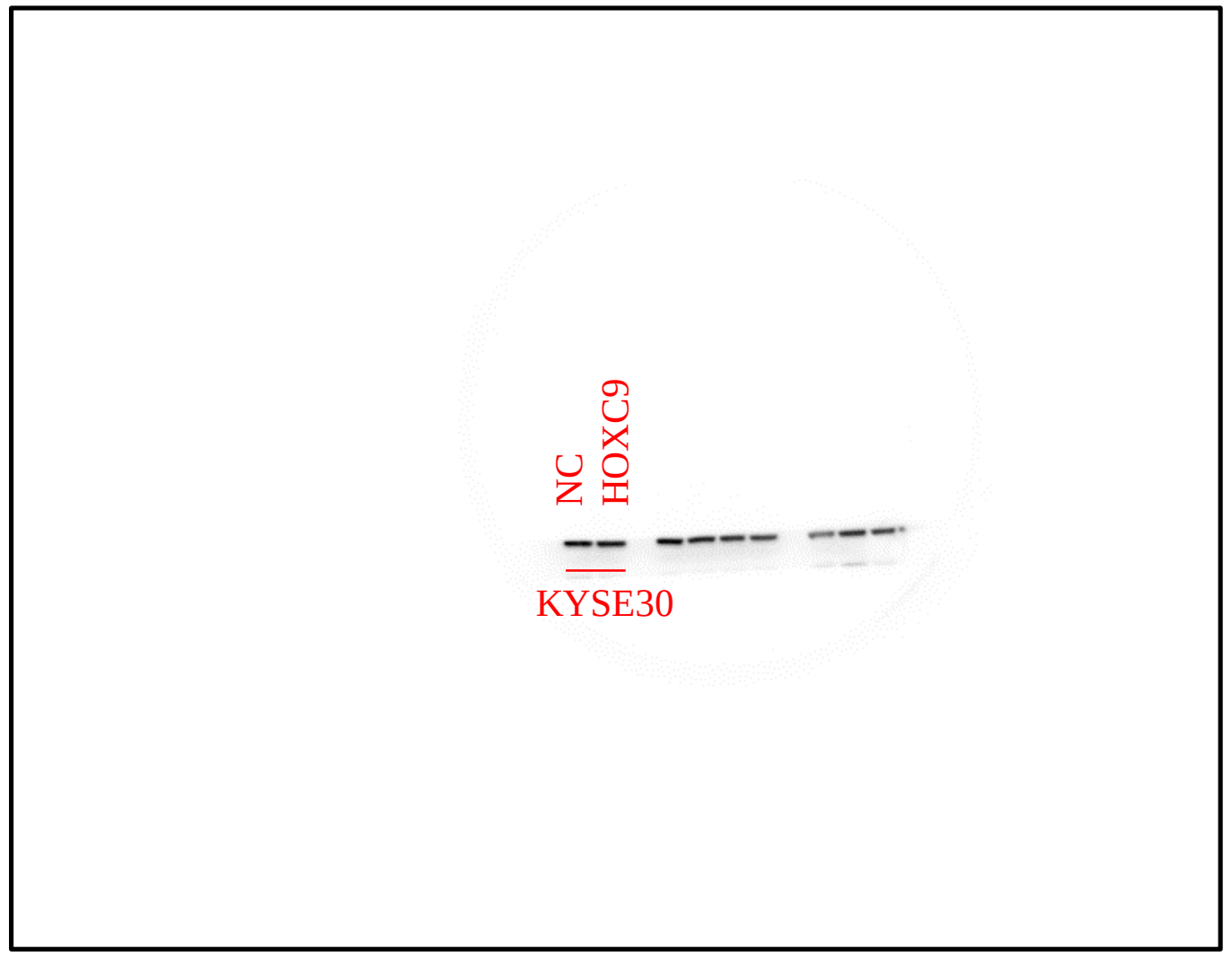

Fig. S5B HOXC9

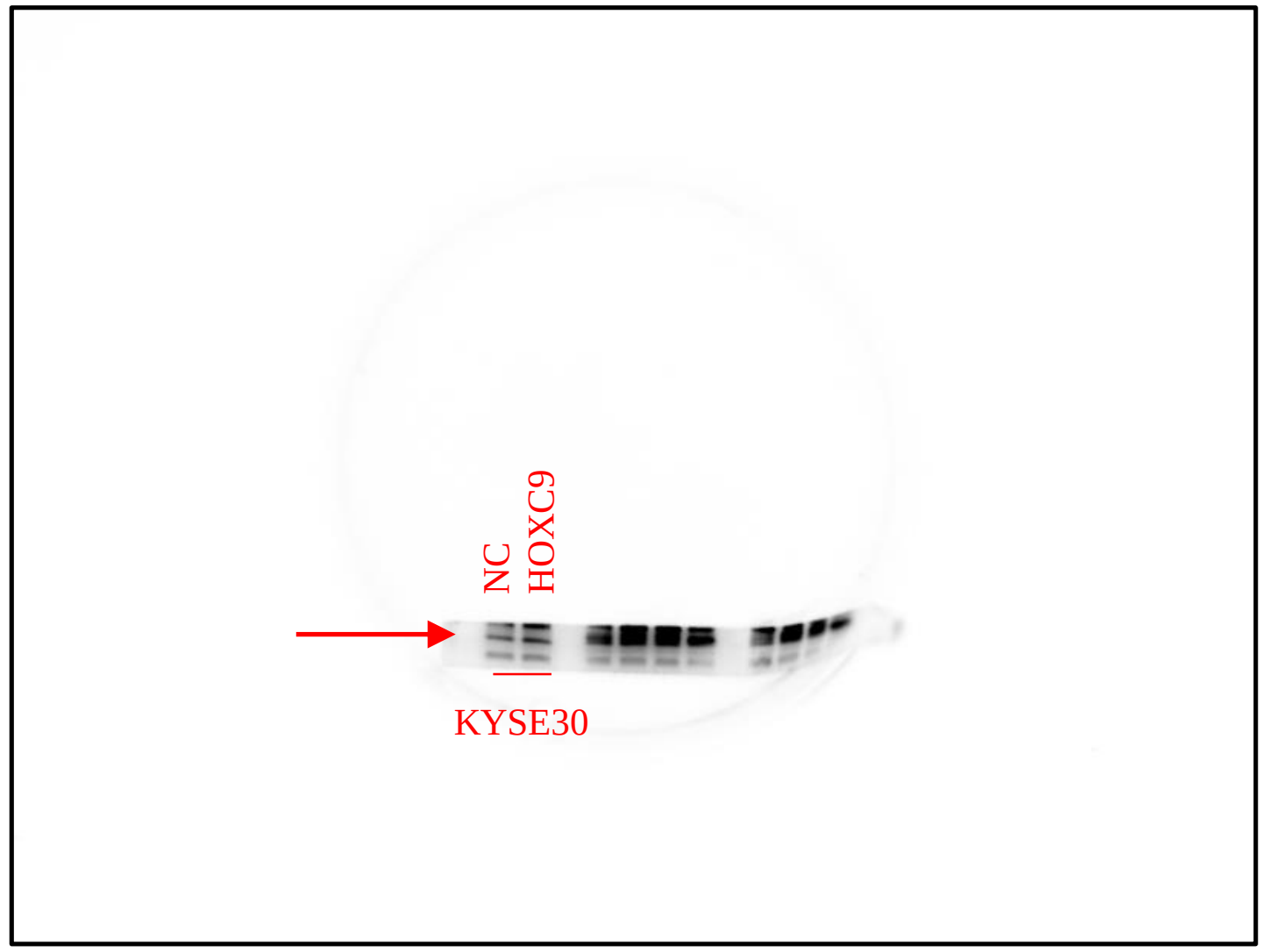

Fig. S5B FABP5

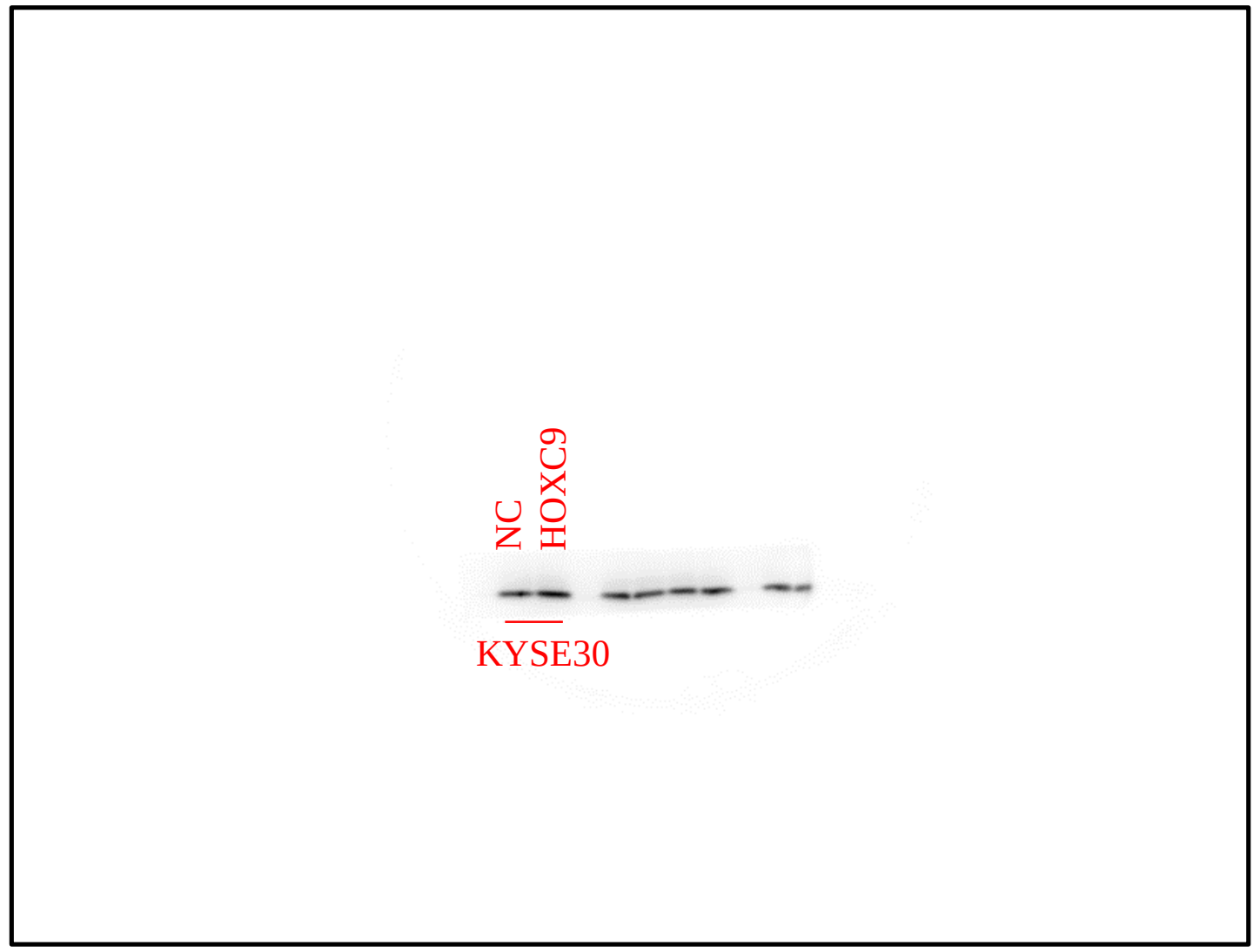

Fig. S5C β-actin

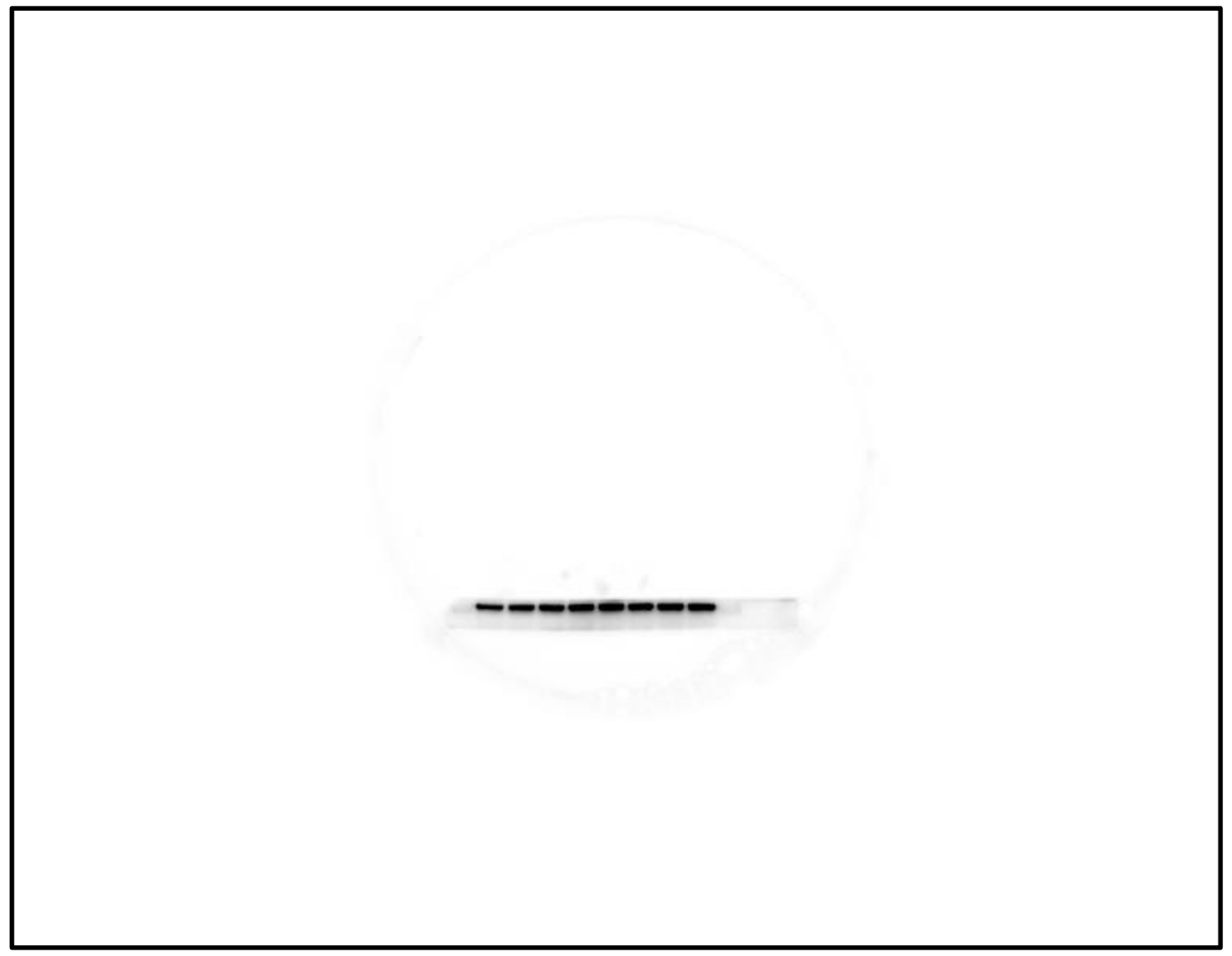

Fig. S5C HOXC9

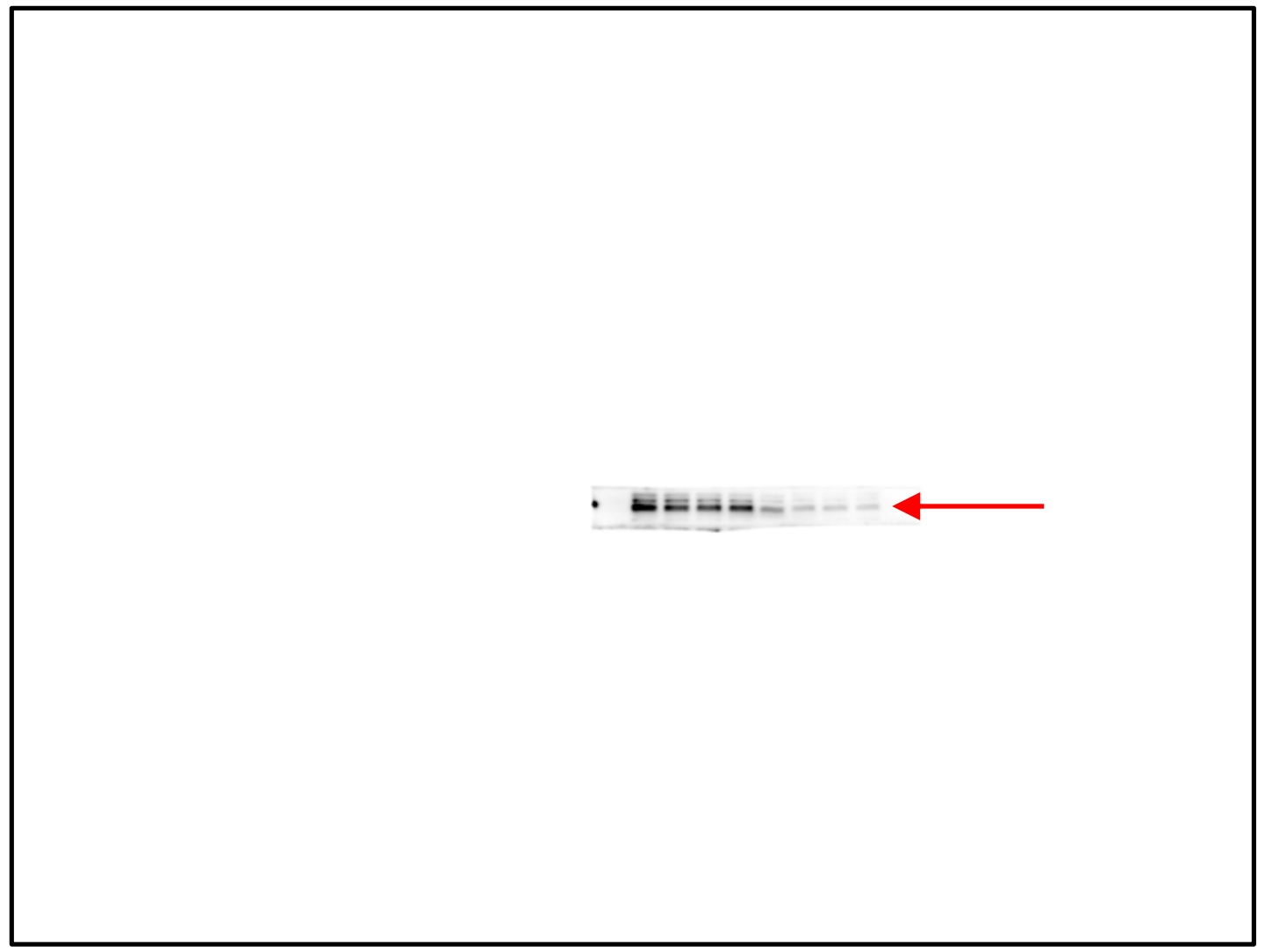

Fig. S5C FABP5

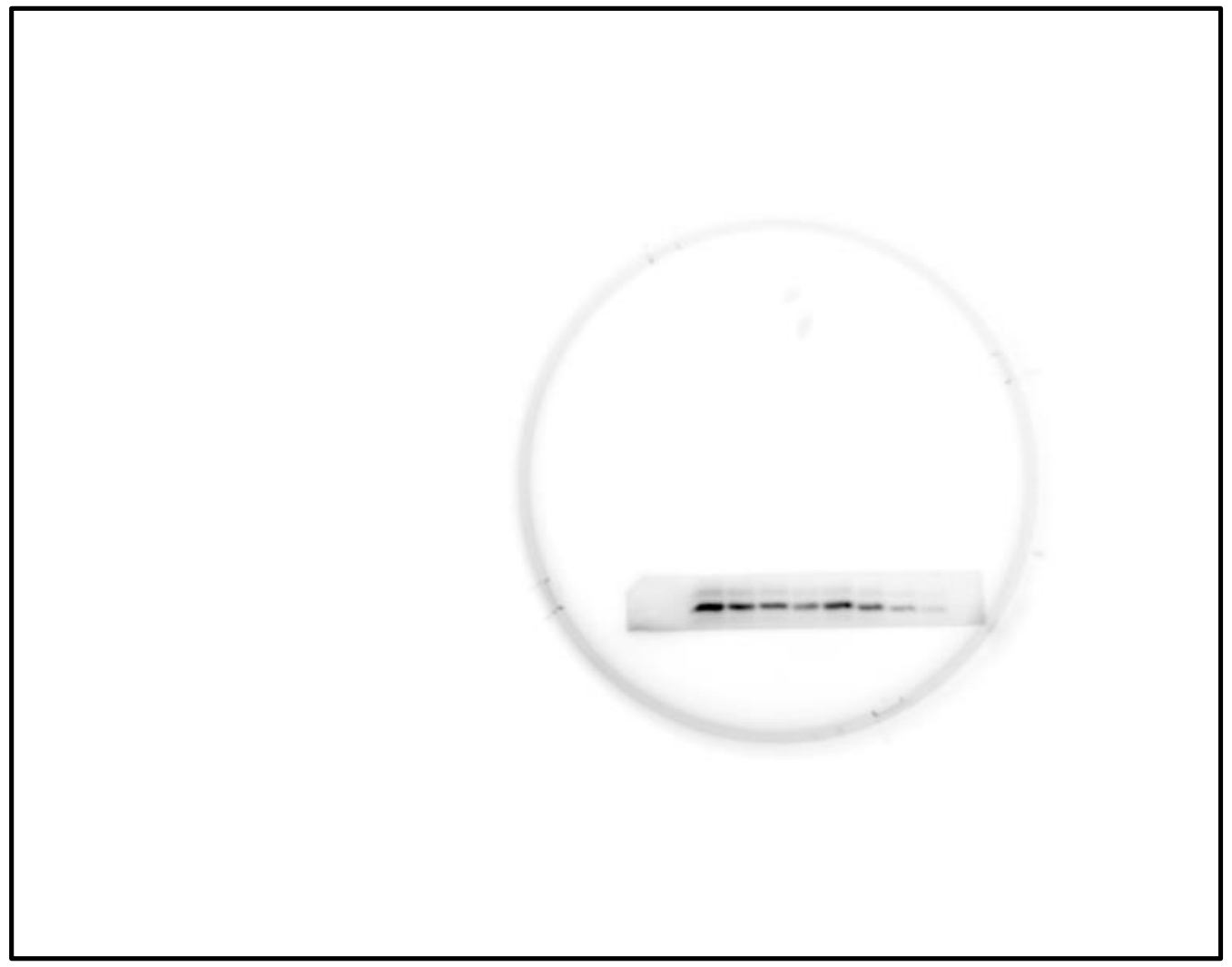

Fig. S5D β-actin

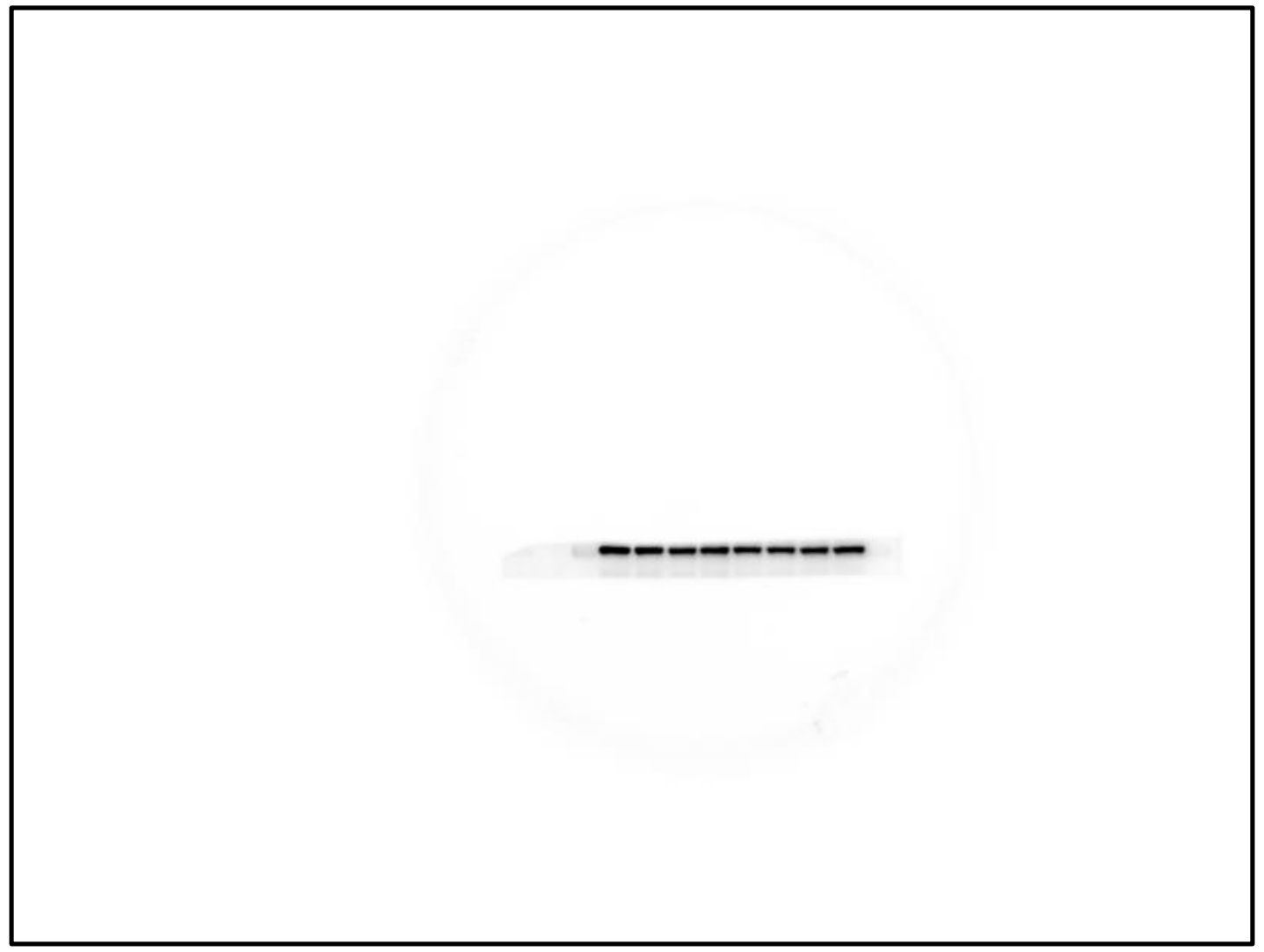

Fig. S5D HOXC9

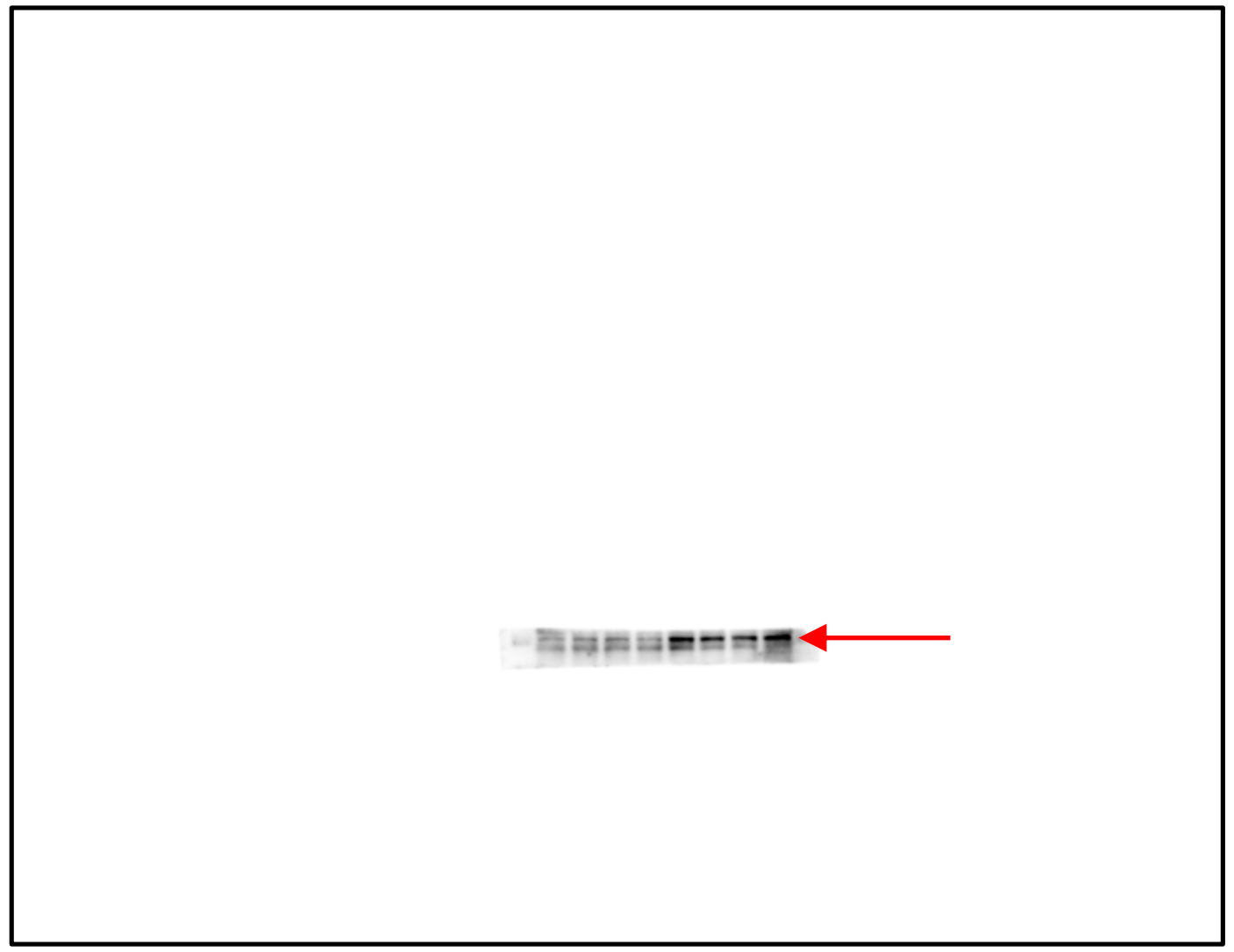

Fig. S5D FABP5

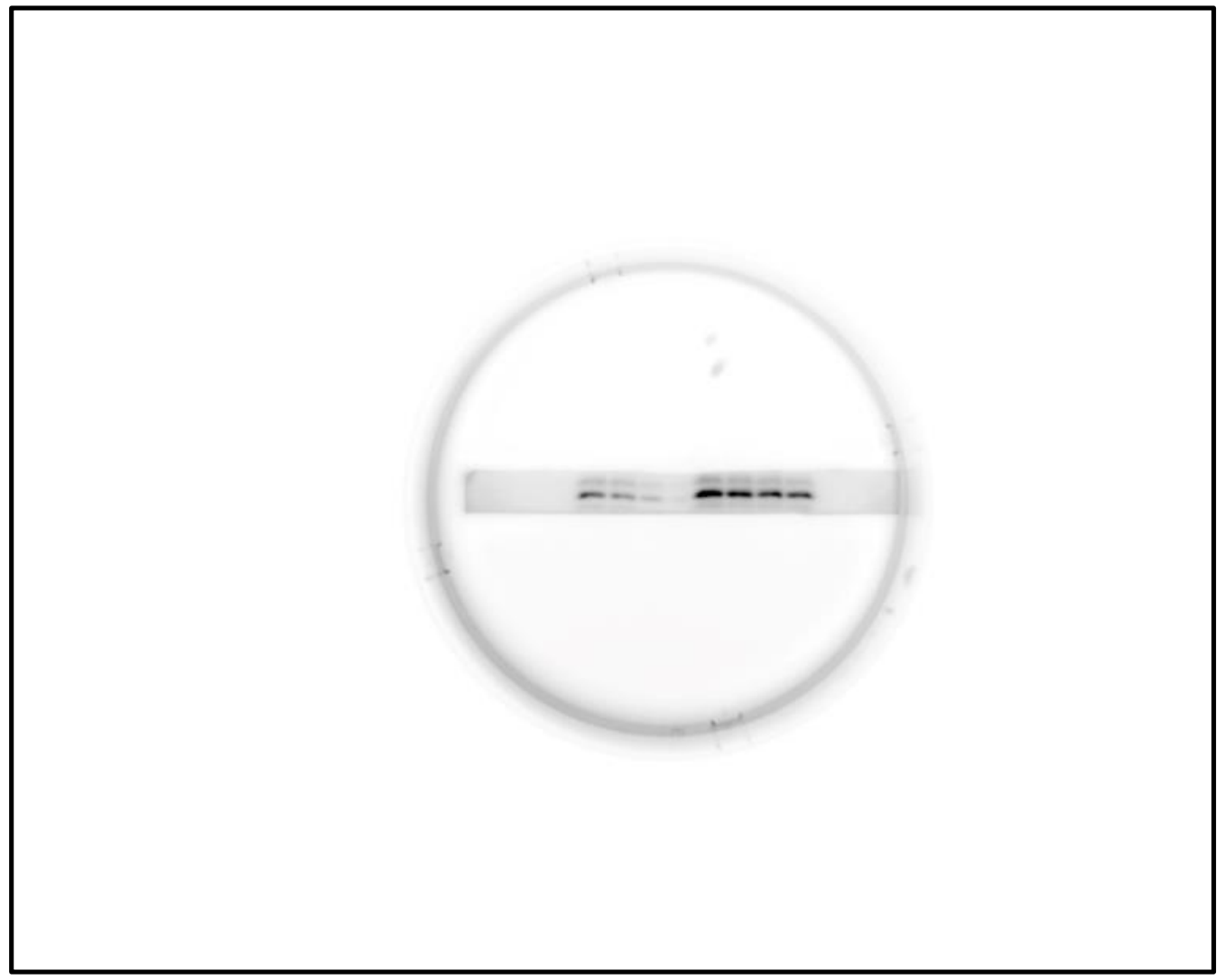

Fig. S6A β-actin

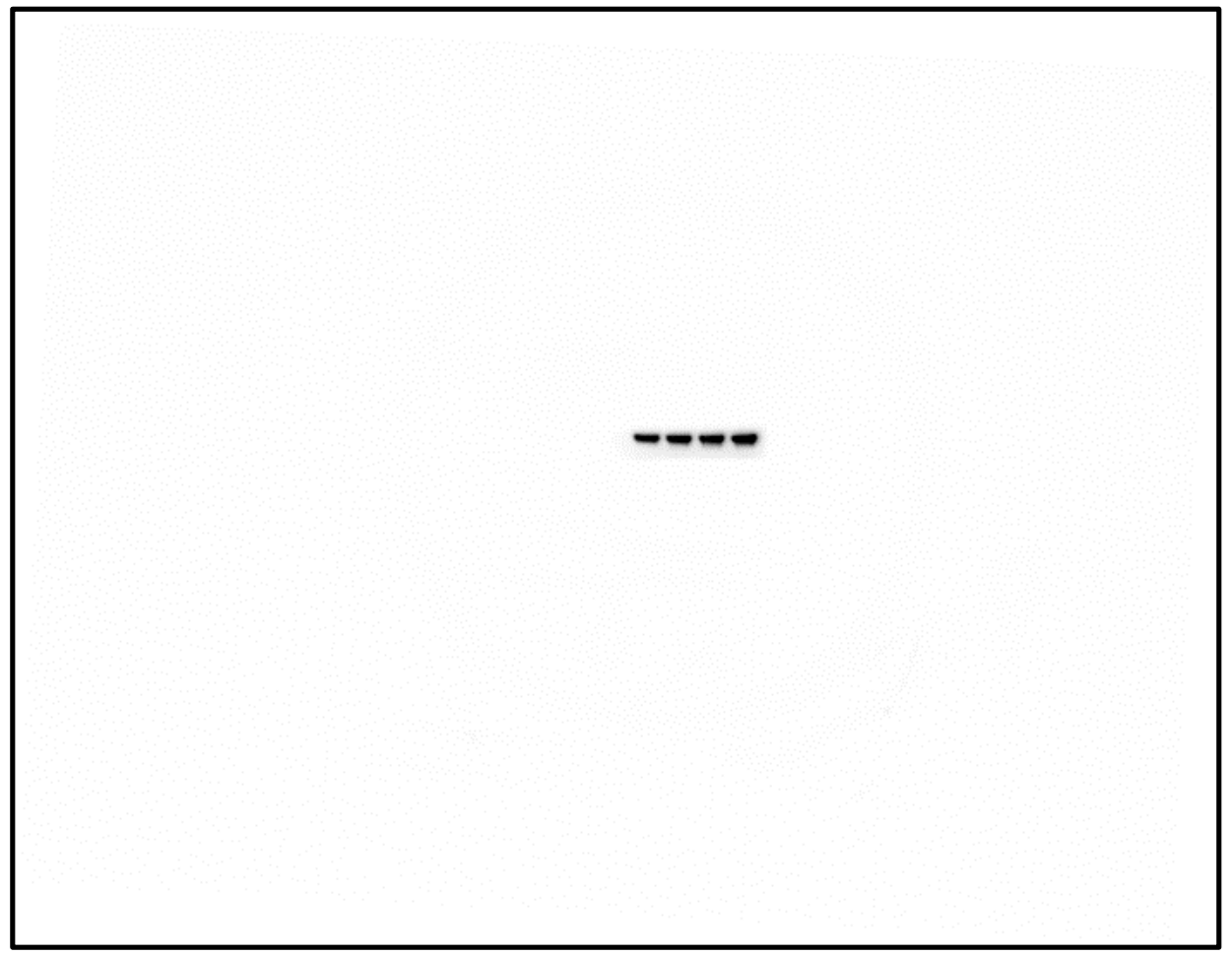

Fig. S6A HOXC9

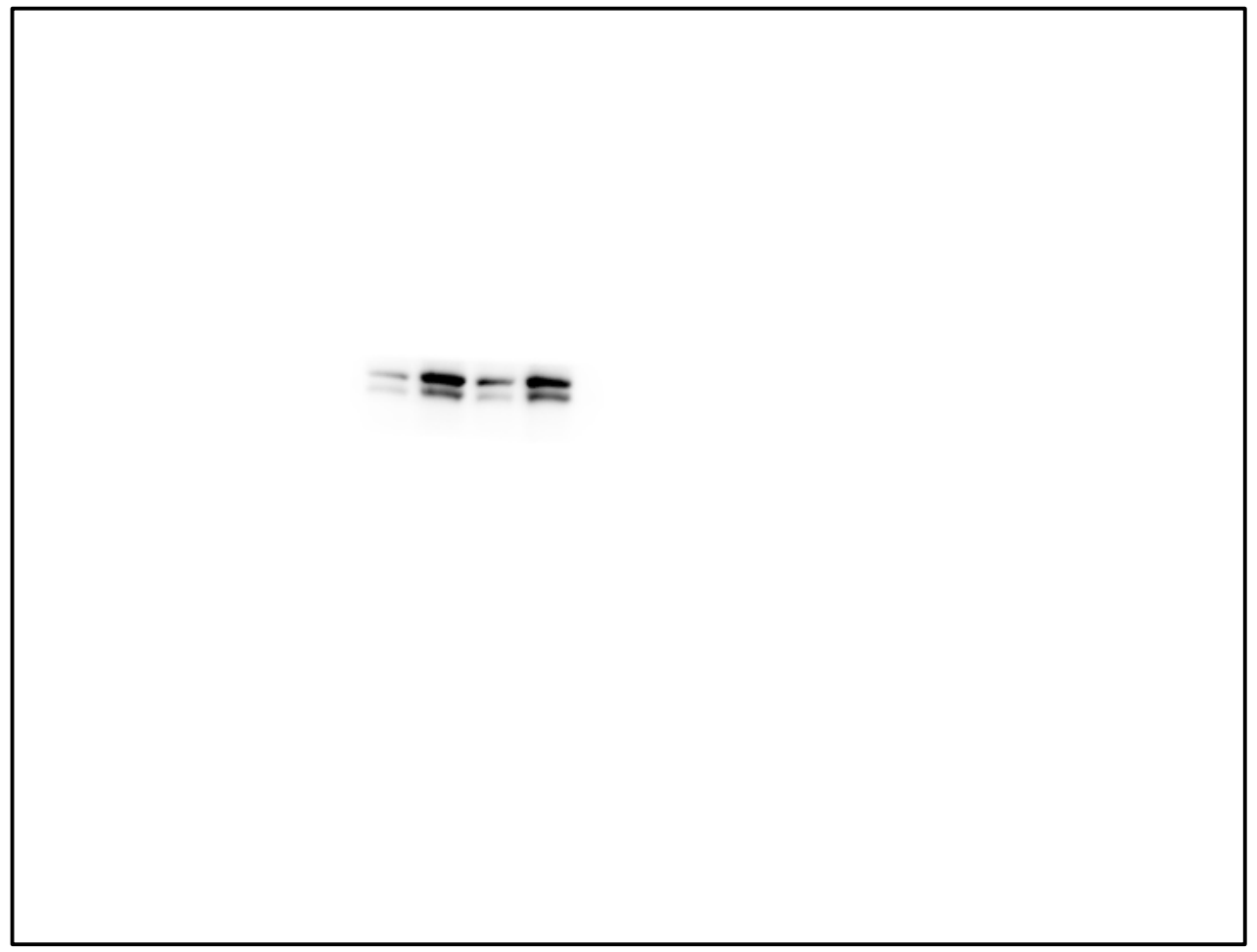

Fig. S6A OTUD1

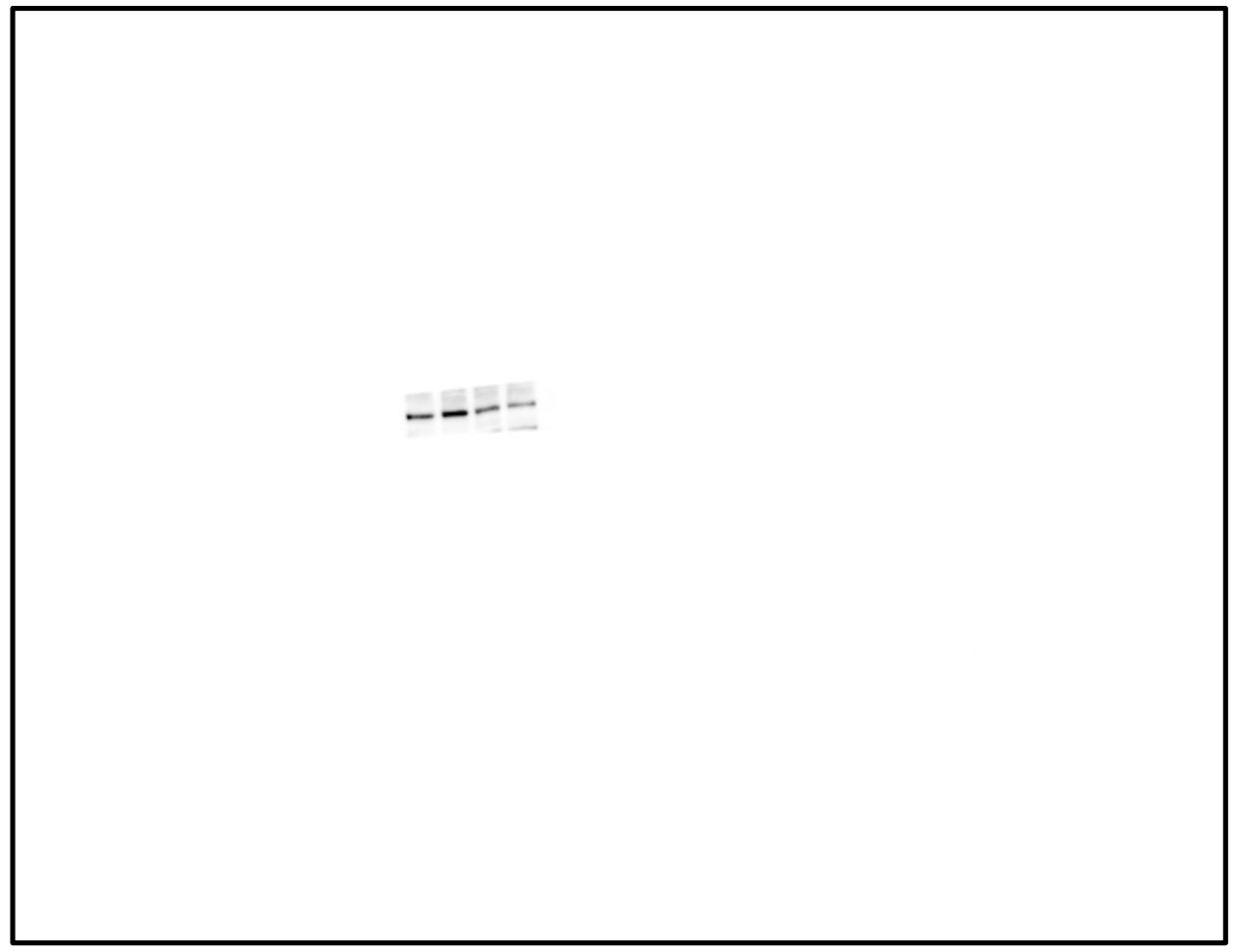

Fig. S6A FABP5

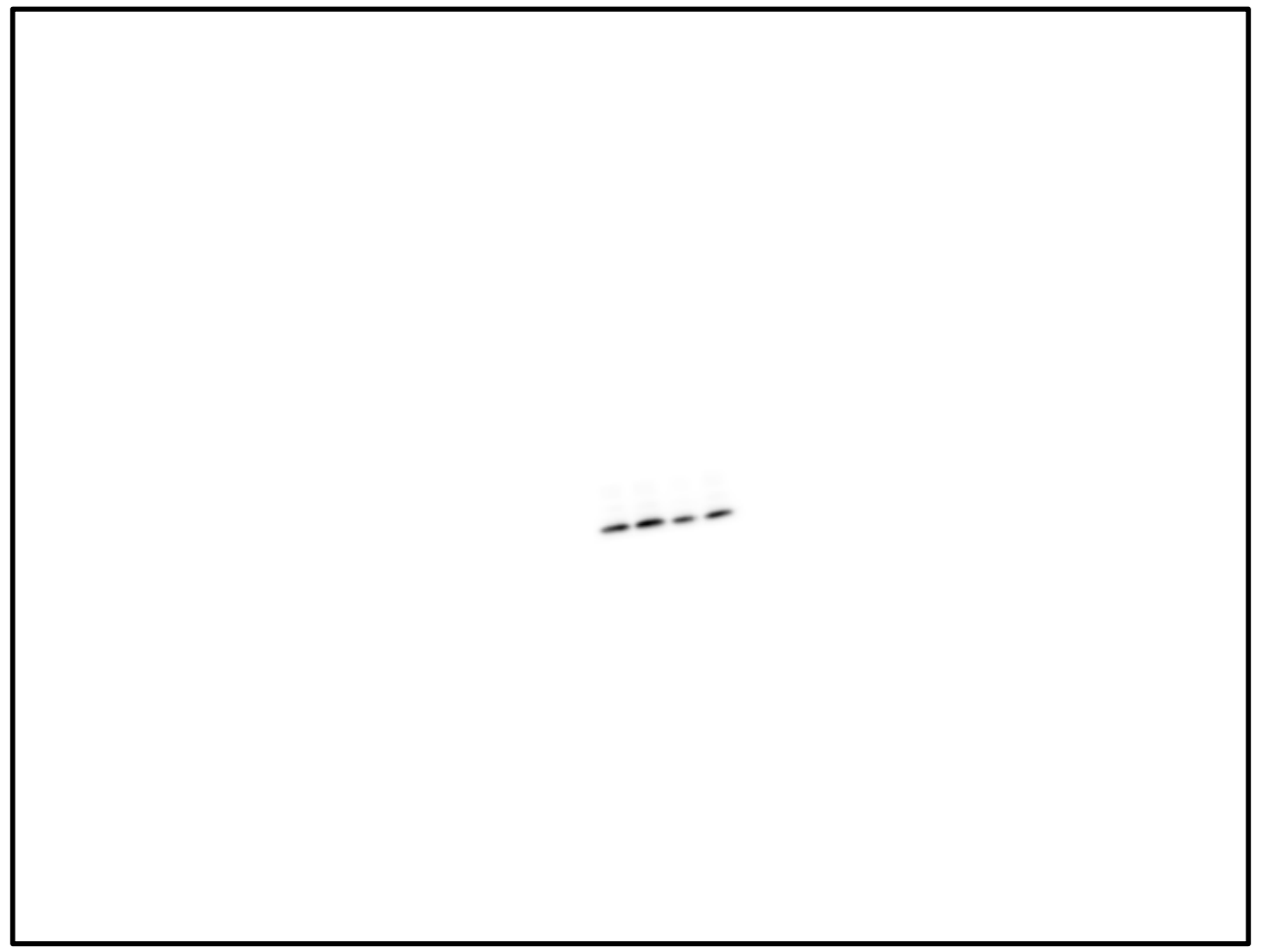

Fig. S7A β-actin

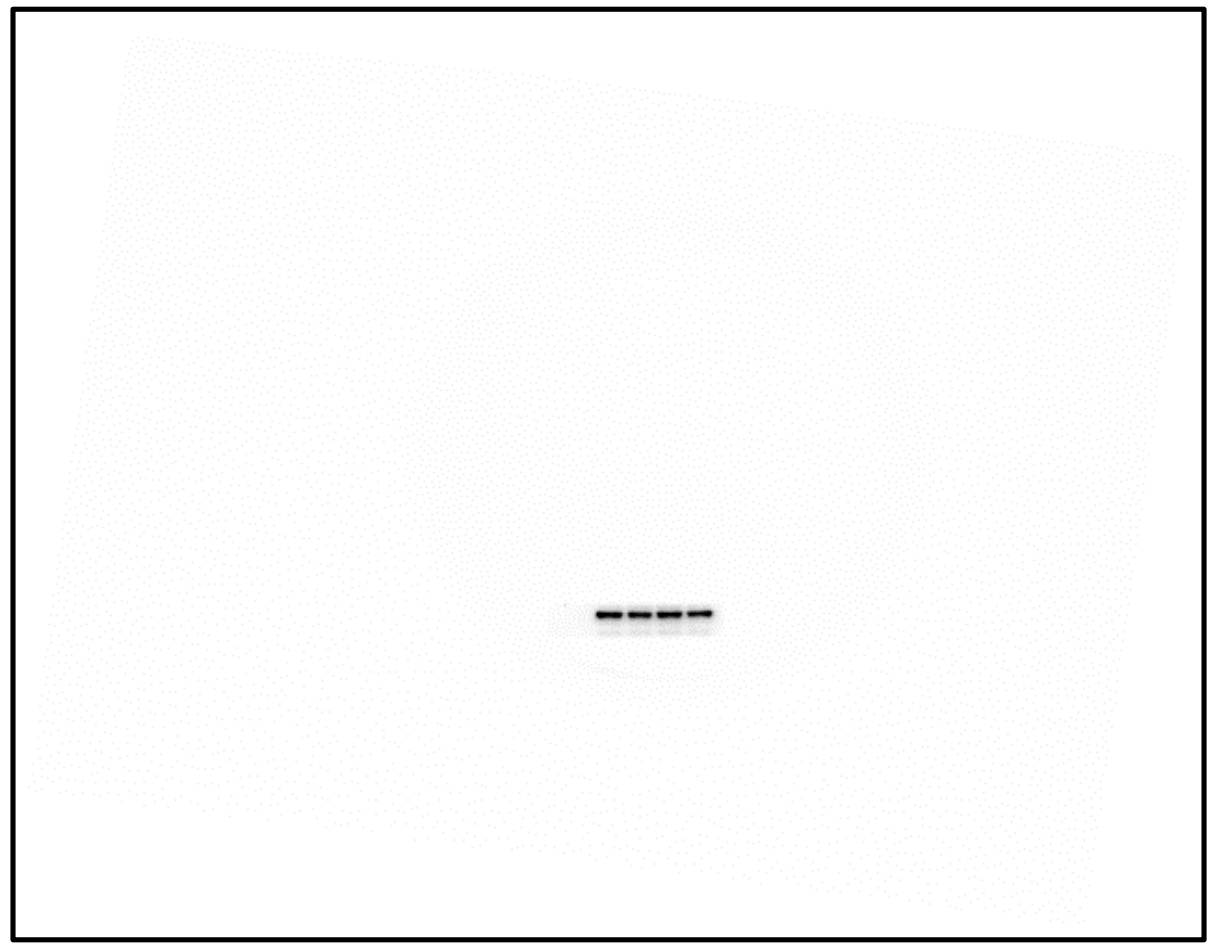

Fig. S7A HOXC9

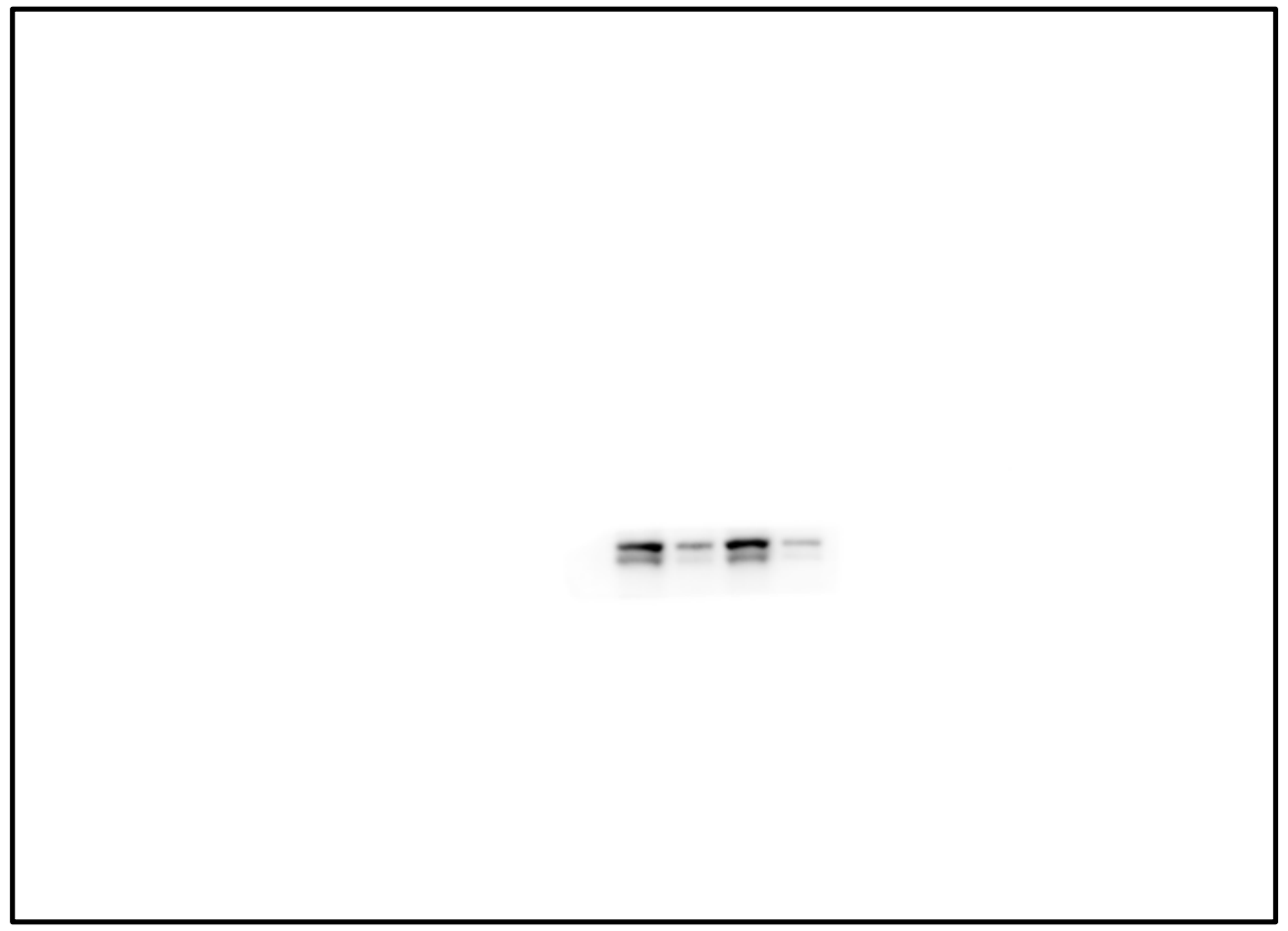

Fig. S7A OTUD1

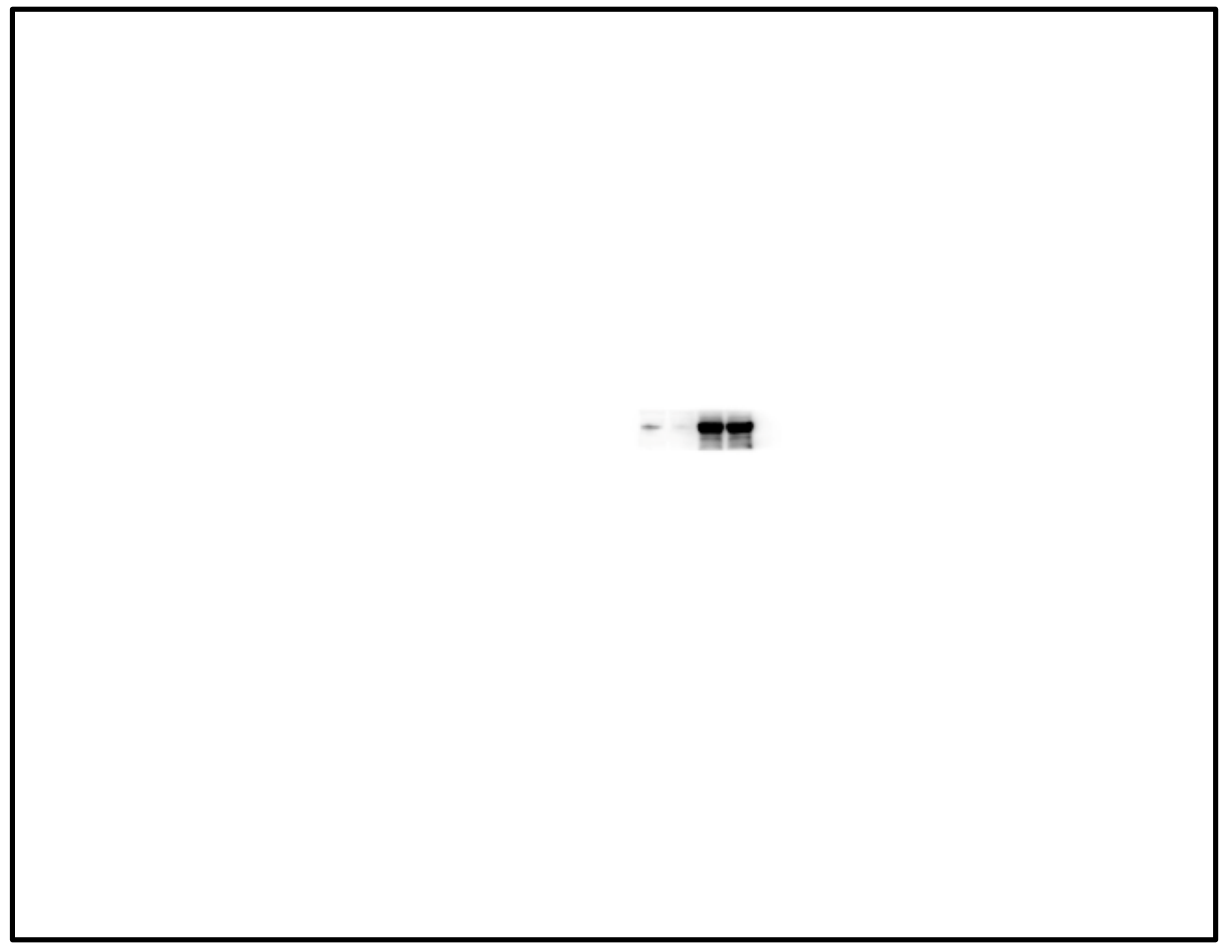

Fig. S7A FABP5

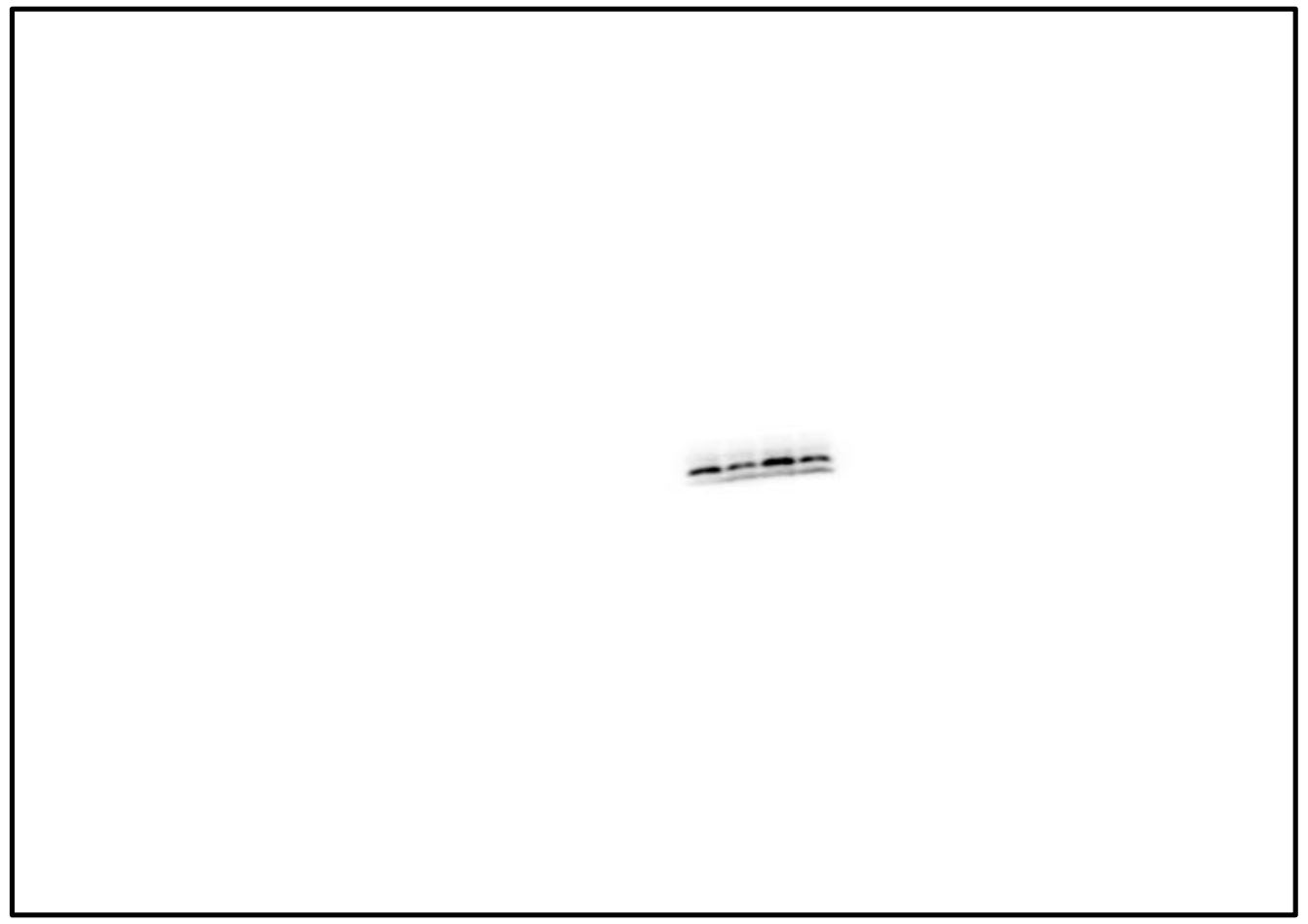

Supplement: Supplementary file 2 [file 41419_2026_9013_MOESM3_ESM.pdf]
