# Supplementary material for: HOXC9 accelerates esophageal squamous cell carcinoma progression via OTUD1-FABP5-mediated lipid metabolic reprogramming
Source: Cell Death Dis. 2026 Jun 30;17(1):809. doi: 10.1038/s41419-026-09013-4 (PMC13586250; doi:10.1038/s41419-026-09013-4)
Supplement: Supplementary file 3 [file 41419_2026_9013_MOESM2_ESM.docx]

**Table S1.** shRNAs and siRNAs used in the study.

| **shRNAs and siRNAs** | **Sequences (5'-3')** |
| --- | --- |
| shHOXC9 #1 | CTCATCTCTCACGACAATGAA |
| shHOXC9 #2 | CCGGGTTCTCAATCTCACCGA |
| shOTUD1 | CTGAATGTGAATATCCATTTA |
| si-HOXC9 | CAGGUACGAAUAGUCAAGA |
| si-OTUD1 | CGGACACUAUGAUGCUGUA |
| si-FABP5 | UGUACCCUGGGAGAGAAGU |

**Table S2**. Primers used in the study.

| **Primers** | **Sequences** |
| --- | --- |
| *HOXC9* | Forward 5'-ACTCGCTCATCTCTCACGACA-3' |
|  | Feverse 5'-GACGGAAAATCGCTACAGTCC-3' |
| *FABP5* | Forward 5'-ACATGAAGGAGCTAGGAGTGG-3' |
|  | Forward 5'-TCTCCCAGGGTACAAGAAAACTG-3' |
| *OTUD1* | Forward 5'-GACGAGAAGCTGGCCCTATAC-3' |
|  | Feverse 5'-TGGAATGATGTGGAATCGGTACT-3' |
| *OTUD1*-Promoter site 1 | Forward 5'-GCTTGGGGCGAGTTAGCAT-3' |
|  | Feverse 5'-GTAGCCCATGACCAGCAGAG-3' |
| *OTUD1*-Promoter site 2 | Forward 5'-CTAGCGGACTCACCCTTACC-3' |
|  | Feverse 5'-CTTCCTCAGCCTTGCCTTGG-3' |

**Table S3**. Antibodies used in the study.

| **Antibody** | **Manufacturer** | **Catalog#** | **Clone No.** | **Working dilution** |
| --- | --- | --- | --- | --- |
| HOXC9 | Santa Cruz Biotechnology | sc-81100 | HOXCA6E6 | WB: 1:500  IHC: 1:50 |
| OTUD1 | Proteintech | 29921-1-AP | Polyclonal | WB: 1:4000  IHC: 1:250 |
| FABP5 | Proteintech | 12348-1-AP | Polyclonal | WB: 1:3000  IHC: 1:250 |
| Ubiquitin | Proteintech | 80992-1-RR | 6H6 | WB: 1:10000 |
| Bax | Proteintech | 50599-2-Ig | Polyclonal | WB:1:20000 |
| Cyclin B1 | Abways | CY5378 | Polyclonal | WB:1:2000 |
| β-actin | Abways | AB0035 | Polyclonal | WB:1:10000 |
| β-tubulin | Abmart | M20005 | 10B1 | WB:1:5000 |
| HRP-conjugated Goat Anti-Rabbit IgG(H+L) | Proteintech | SA00001-2 | Polyclonal | WB:1:10000 |
| HRP-conjugated Goat Anti-Mouse IgG(H+L) | Proteintech | SA00001-1 | Polyclonal | WB:1:10000 |
| Mouse IgG | Proteintech | B900620 | Polyclonal | ChIP: 1 µg per 4×10⁶ cells |
| Rabbit IgG | Proteintech | 30000-0-AP | Polyclonal | IP: 1 µg per 500 µg total protein |
